# Arrhythmia mechanisms and spontaneous calcium release: Bi-directional coupling between re-entrant and focal excitation

**DOI:** 10.1371/journal.pcbi.1007260

**Published:** 2019-08-08

**Authors:** Michael A. Colman

**Affiliations:** School of Biomedical Sciences, University of Leeds, Leeds, United Kingdom; University of Washington, UNITED STATES

## Abstract

Spontaneous sub-cellular calcium release events (SCRE) are conjectured to promote rapid arrhythmias associated with conditions such as heart failure and atrial fibrillation: they can underlie the emergence of spontaneous action potentials in single cells which can lead to arrhythmogenic triggers in tissue. The multi-scale mechanisms of the development of SCRE into arrhythmia triggers, and their dynamic interaction with the tissue substrate, remain elusive; rigorous and simultaneous study of dynamics from the nanometre to the centimetre scale is a major challenge. The aim of this study was to develop a computational approach to overcome this challenge and study potential bi-directional coupling between sub-cellular and tissue-scale arrhythmia phenomena. A framework comprising a hierarchy of computational models was developed, which includes detailed single-cell models describing spatio-temporal calcium dynamics in 3D, efficient non-spatial cell models, and both idealised and realistic tissue models. A phenomenological approach was implemented to reproduce SCRE morphology and variability in the efficient cell models, comprising the definition of analytical Spontaneous Release Functions (SRF) whose parameters may be randomly sampled from appropriate distributions in order to match either the 3D cell models or experimental data. Pro-arrhythmogenic pacing protocols were applied to initiate re-entry and promote calcium overload, leading to the emergence of SCRE. The SRF accurately reproduced the dynamics of SCRE and its dependence on environment variables under multiple different conditions. Sustained re-entrant excitation promoted calcium overload, and led to the emergence of focal excitations after termination. A purely functional mechanism of re-entry and focal activity localisation was demonstrated, related to the unexcited spiral wave core. In conclusion, a novel approach has been developed to dynamically model SCRE at the tissue scale, which facilitates novel, detailed multi-scale mechanistic analysis. It was revealed that complex re-entrant excitation patterns and SCRE may be bi-directionally coupled, promoting novel mechanisms of arrhythmia perpetuation.

## Author summary

A loss of the regular rhythm of the beating heart, called arrhythmia, can inhibit its pumping function and even lead to sudden death. Understanding the processes by which normal rhythm is interrupted presents a major yet critical research problem. One challenge is the inherent multi-scale dependence of the electrical activity of the heart: behaviour at the microscopic scales (single proteins) can propagate to the macroscopic (whole-heart). Simultaneously studying phenomena at both of these scales is difficult, if not impossible, to perform experimentally. Developing mathematical models of the heart in order to perform variable and controlled simulations of its electrical activity provides the possibility to undertake such multi-scale analysis. However, new approaches are required to simulate the potential role of “spontaneous calcium release”, which occurs at the sub-cellular scale, in triggering arrhythmia events at the whole-heart scale. This study develops an approach to perform such simulations and applies it to study the long-term interactions of these sub-cellular triggers with the complex electrical activity of the most dangerous arrhythmias–tachycardia and fibrillation. The new mechanistic insight has implications for both diagnosis and treatment of the associated disorders.

## Introduction

Cardiovascular disease is one of the major healthcare problems faced by the developed world, with increasing prevalence associated with aging populations [1–3]. Improved understanding of the mechanisms underlying cardiac arrhythmias, a major component of cardiovascular diseases’ impact on morbidity and mortality, is vital to the effort to improve both quality and duration of life. Rapid arrhythmias, such as tachycardia and fibrillation, are associated with disordered and incomplete contraction, reducing cardiac output and potentially leading to sudden cardiac death. The underlying rapid and irregular electrical activation of cardiac tissue may be mediated by abnormal spontaneous pacing (focal ectopic activity; “arrhythmia triggers”), self-perpetuating re-entrant excitation (“arrhythmia substrate”), or a complex interplay between both mechanisms (trigger-substrate interactions) [4,5]. Management of these arrhythmias is typically challenging, often requiring invasive procedures such as implanted defibrillators or catheter ablation; even these interventions have limited success rates [6,7]. Understanding of the mechanisms underlying the genesis, perpetuation and recurrence of rapid arrhythmias will ultimately lead to the development of improved treatment strategies.

Whereas multiple experimental and simulation studies have investigated the tissue substrate for the emergence and perpetuation of re-entrant excitation [8,9], the multi-scale mechanisms of arrhythmia triggers, and their dynamic interaction with the tissue substrate, remain elusive [10]. Malfunction of the intracellular calcium (Ca^2+^) handling system has been implicated in the development of rapid arrhythmias, linking sub-cellular spontaneous Ca^2+^ release events (SCRE) to pro-arrhythmic triggers in single cell [11–15]. However, translation of these cellular data to assess the mechanisms and importance of SCRE in tissue-scale arrhythmia is a significant challenge [10], namely because of the dependence of SCRE on stochastic fluctuations at the sub-cellular, nanometre scale which must propagate to the whole-organ, centimetre scale. These complex multi-scale mechanisms are discussed in detail in previous reviews [11,12,16], and are briefly summarised below.

A feedback mechanism emerging from the structure-function relationships underlying cardiac cellular excitation-contraction coupling presents a potential pro-arrhythmogenic pathway for the propagation of random state-transitions at the microscopic scale (sub-cellular) to the macroscopic (whole-organ): (i) spontaneous opening of the ryanodine receptors (RyRs), which control release of Ca^2+^ from the Sarcoplasmic Reticulum (SR) into the intracellular space, can trigger spontaneous Ca^2+^ sparks in restricted nanodomains called dyads; (ii) the sub-cellular spatial distribution of dyads presents a substrate for the propagation of Ca^2+^ sparks as a whole-cell event; (iii) this spontaneous whole-cell Ca^2+^ transient can lead to cellular delayed-after-depolarisations (DADs) and triggered action potentials (TA) through activation of the sodium-calcium exchanger (NCX); (iv) TA may propagate in tissue as ectopic focal excitation, potentially leading to the initiation of transient or sustained arrhythmia. The dynamics of Ca^2+^ homeostasis, also strongly dependent on the refilling of the SR through the SR-Ca^2+^ pump (SERCA), is critical for the emergence of SCRE: cardiac cells typically exhibit a minimum SR-Ca^2+^ load threshold above which whole-cell SCRE occur. The cytosolic Ca^2+^ concentration and kinetics of the RyRs (which may be more prone to spontaneous release in disease [14,17]) also strongly affect SCRE dynamics and vulnerability, which can be reflected in adaptations of this SR-Ca^2+^ load dependence (wherein lower thresholds correspond to larger vulnerability to SCRE).

Computational modelling provides a viable approach for detailed simultaneous multi-scale evaluation of cardiac arrhythmia mechanisms. Nevertheless, simulating SCRE in tissue-scale models is non-trivial due to the contrasting computational requirements of models at these different scales: single-cell models capable of reproducing SCRE are computationally intensive and unsuitable for simulation of the thousands or millions of coupled cells comprising tissue models appropriate for studying arrhythmia mechanisms. New approaches must therefore be developed to realise this goal. Only a few previous studies have attempted such modelling, for example investigating: (i) the minimum tissue substrate for the emergence of focal excitations resulting from non-stochastic DADs [18]; (ii) the emergence of focal excitation from stochastic SCRE [19–23] and its potential interaction with extracellular matrix remodelling [24]; (iii) SCRE as a mechanism for both triggered activity and conduction block [25]; and (iv) the potential complex considerations for pharmacological action on both triggers and substrate [22].

In a previous study, a phenomenological approach was introduced to model the synchronisation of cellular triggers in tissue [22]. The present study aims to refine this approach, and extend it to allow dynamic modelling of trigger-substrate interactions. The novel approach, comprising a hierarchy of computational models, is applied to: (i) directly translate behaviour observed in detailed cell models to the tissue-scale and (ii) study the dynamic interactions of SCRE and re-entrant excitation. The approach is also generalised to allow fully controllable investigations and integrate limited experimental data.

## Methods

This section first outlines the computational framework and the hierarchy of cell and tissue models of which it comprises. Then, analysis of stochastic SCRE observed using the spatial single cell models is discussed in relation to the development of analytical waveform functions which approximate SCRE in the non-spatial cell- and multi-dimensional tissue-models, termed Spontaneous Release Functions (SRF). Multiple implementations are presented in which the distributions from which waveform parameters are sampled are determined: (i) by directly controlled user inputs (“**Direct Control**” model); (ii) dynamically to reproduce the behaviour of the spatial cell model under multiple conditions (“**Dynamic Fit”** model); or (iii) dynamically based on user-controlled inputs (“**General Dynamic**” model). Finally, simulation protocols for studying different arrhythmia dynamics are described.

### The computational framework

**Fig 1:**
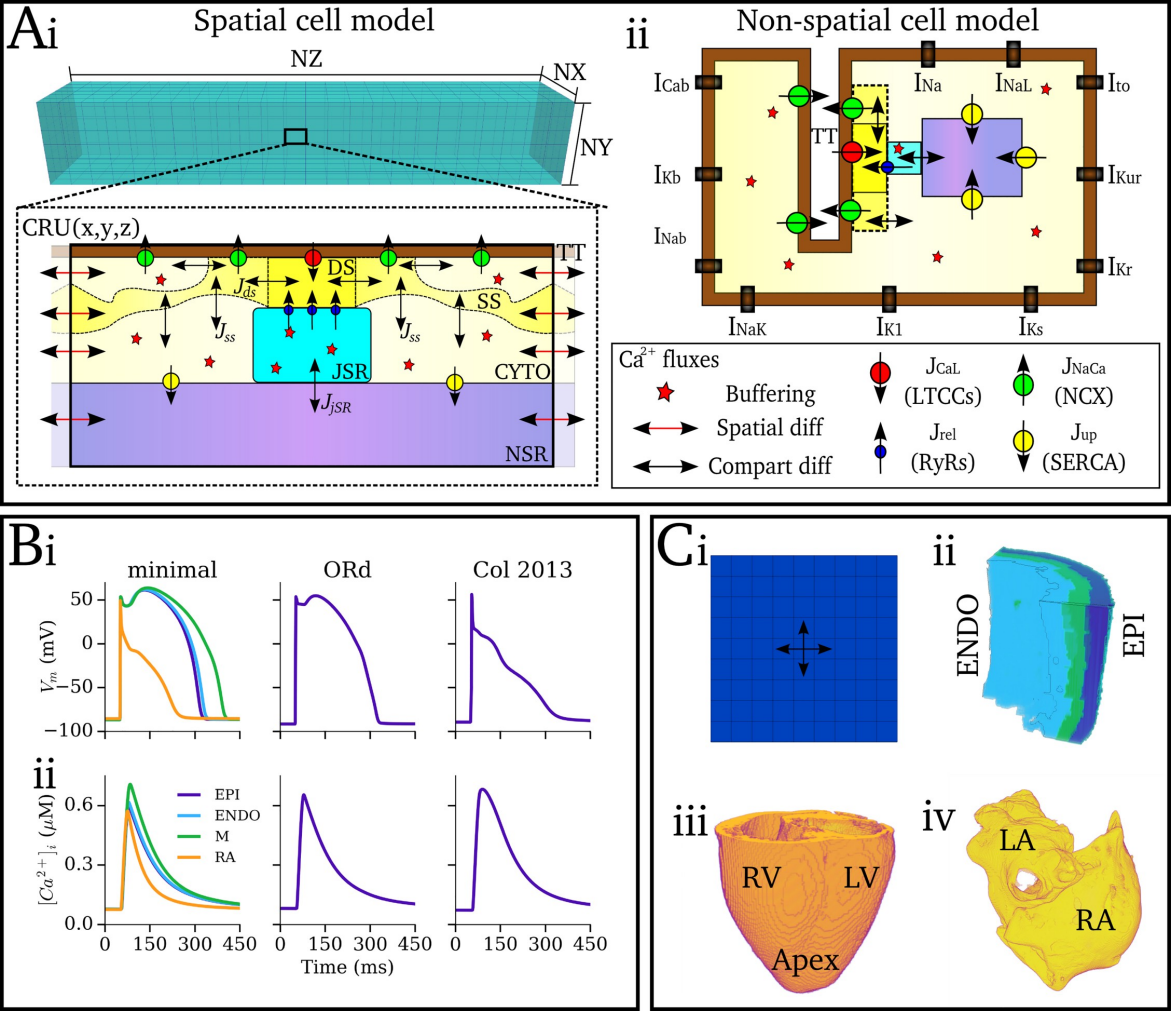
Components of the multi-scale computational framework. A–Schematic of single cell Ca^2+^ handling and ion current models. (i)– 3D, microscopic Ca^2+^ handling model, illustrating the 3D grid of calcium release units (CRUs; upper panel) and the compartments and Ca^2+^ fluxes within a single CRU (lower panel). Labelled are the dyadic cleft space (DS), sub-space (SS), bulk cytosolic space (CYTO), network and junctional SR spaces (NSR, JSR), and a T-tubule (TT); fluxes through the LTCCs (*J*_CaL_), RyRs (*J*_rel_), NCX (*J*_NaCa_) and SERCA (*J*_up_) are illustrated according to the key; double-headed black arrows indicate transfer between compartments; double-headed red arrows indicate diffusion between neighbouring CRUs. (ii)– 0D, non-spatial cell model, illustrating the same fluxes as in (i) but without inter-CRU diffusion. Global ion currents are illustrated along the membrane (which apply to both models). B–Whole-cell voltage (i) and calcium transient (ii) of the different ion-current models used in the present study, showing the hybrid-minimal model (left; minimal), O’Hara et al., human ventricular model [26] (middle; ORd) and Colman et al., human atrial model [27] (right; Col 2013). C–Tissue models, showing schematic of a 2D sheet model (i), and the 3D anatomical reconstructions: (ii)—human ventricular wedge [28]; (iii) whole canine ventricle [29]; and (iv) whole human atria [27,30–32].

The developed framework consists of a hierarchy of computational models (Fig 1):

1. The microscopic or 3D spatio-temporal cell model is the baseline model for the framework. It accounts for spatially distributed dyads, Ca^2+^ diffusion within the cell, and stochastic state transitions in the L-type Ca^2+^ channel (LTCC) and RyR models (Fig 1Ai and 1B). This model was used to study SCRE at the cellular scale.
2. The deterministic, 0D or non-spatial cell model is derived from the microscopic model. It does not contain a distributed Ca^2+^ handling structure or account for stochastic state transitions (Fig 1Aii and 1B). This manuscript focuses on the derivation and introduction of analytical Spontaneous Release Functions (SRF) into 0D models to reproduce behaviour from the 3D spatio-temporal cell model.
3. The Tissue model refers to coupled 0D cell models in either idealised 2D sheets or 3D reconstructions of cardiac anatomy (Fig 1C).

#### The microscopic, 3D spatio-temporal Ca^2+^ handling model

The microscopic model, a simplification of a previously presented and structurally detailed model [33], consists of an idealised 3D intracellular Ca^2+^ system coupled to a point-source *V*_m_ and ion-current model (Fig 1Ai and 1B). The model is capable of reproducing normal and abnormal Ca^2+^ dynamics [33] and captures the general properties of experimentally observed SCRE [11,14,34,35] in-line with the most recent modelling studies [19,25,36]. The intracellular model incorporates a 3D grid of CRUs, each containing five compartments (three intracellular compartments comprising the bulk-cytoplasm, sub-space, and restricted dyadic cleft, as well as the network and junctional SR spaces; Fig 1Ai). The bulk cytosolic, sub-space and network SR compartments are coupled to neighbouring CRUs. Each dyad contains ~10 LTCCs and ~100 RyRs (varies between models and can be heterogeneous within a cell), described by stochastic differential equations [33]. Full details and model equations are provided in the S1 Text (Model Description). Most relevant for this study is the reaction for the dyadic cleft, *Φ*_*ds*_, which contains intracellular Ca^2+^ release, *J*_rel_. For a single dyad *n*, *J*_rel_ is described by:

$${}^{n}J{}_{rel}={}^{n}N{}_{RyR\_O}.g_{RyR}.{}^{n}v{}_{ds}^{-1}\left({}^{n}\left[Ca^{2+}\right]{}_{jSR}-{}^{n}\left[Ca^{2+}\right]{}_{ds}\right)$$ (1)

Where *g*_RyR_ is the maximal flux factor for Ca^2+^ release, ^n^*v*_ds_ is the volume of the dyadic cleft space, and ^n^*N*_RyR_O_ is the summed number of open RyR channels in dyad *n*, determined through the stochastic Monte-Carlo method. *N*_RyR_O_ is therefore the primary variable which describes the dynamics of SCRE, and thus the variable used to integrate spatial and non-spatial cell models.

#### The deterministic, 0D model

The deterministic model structure is identical to a single CRU of the microscopic model and of the same form as the majority of contemporary cardiac cell models based on the Hogkin-Huxley approach (Fig 1Aii). Both the RyR and LTCC models are described by ordinary differential equations and solved through the deterministic forward-Euler method. *N*_RyR_O_ now represents the whole-cell average of the equivalent dyad variable in the 3D cell model. Modifications to the RyR kinetics model were required due to the poor recapitulation of whole cell Ca^2+^-induced-Ca^2+^-release (CICR) using deterministic approaches–see S1 Text (Model Description) for details.

#### Action potential and tissue models

For the purpose of demonstrating the general potential of the developed framework for integration with contemporary cell models, multiple ionic models describing the non-Ca^2+^ dependent membrane currents were integrated with the Ca^2+^ handling system (Fig 1B): the model was integrated with simplified versions of the O’Hara et al., 2011 human ventricular AP model [26] and Colman et al., 2013 human atrial AP model [27,37]. Furthermore, a hybrid-minimal model (comprising of a minimal setup suitable for coupling with physiological Ca^2+^ currents) was developed which describes human AP morphology in the three transmural cell types of the ventricles as well as the atrial myocardium. This cell-type heterogeneity pertained only to differences in the ionic model and not the intracellular Ca^2+^ handling model. The minimal model is loosely based on a combination of the two biophysically detailed models, but with fewer components not corresponding directly to specific physiological ion currents, and designed with the motivation for full controllability of AP morphology.

Idealised 2D sheet models consist of a 2D array of coupled cells in isotropic medium (Fig 1Ci), including both homogeneous sheets of either the ventricular epicardial layer or the right atrial wall, and a model of the transmural heterogeneity in the ventricular wall (simple ratio of 1:1:1 ENDO:M:EPI cells). 3D models consist of a wedge reconstruction of the human ventricular wall (Fig 1Cii, [28]), a reconstruction of the whole canine ventricle [29] (Fig 1Ciii), and a reconstruction of the whole human atria [27,31] (Fig 1Civ). Further details of the tissue models are presented in S1 Text (Model Description); all tissue models were electrically homogeneous except for the 2D ventricular transmural sheet and human ventricular wedge models, which implemented ENDO, M and EPI cells.

### Pro-arrhythmic conditions

To induce prominent full-cell release events through different cellular conditions, representative (but non-specific) models were included for isoprenaline (ISO, sympathetic response which enhances CICR) and two types of pro-SCRE general disease remodelling mimicking features observed in conditions such as AF and HF (e.g., [14,38]): (i) SERCA was up-regulated and NCX was down-regulated (R_SERCA/NCX_); (ii) the SR-Ca^2+^ threshold for release was lowered through increased inter-CRU coupling (R_CRU-CRU_). R_SERCA/NCX_ also involved remodelling of the ion currents, including a reduction of *I*_K1_, to provide a different AP environment coupled to the Ca^2+^ handling model. Details are provided in S1 Text (Model Description). The purpose of these models, which are not biophysically detailed or representative of specific regulation or diseased conditions, was to induce pro-arrhythmic behaviour which is not observed under control conditions, and test and demonstrate the ability of the 0D approximations to accurately capture variable cellular conditions and translate these to the tissue-scale.

### Partial Ca^2+^-clamp protocol

**Fig 2:**
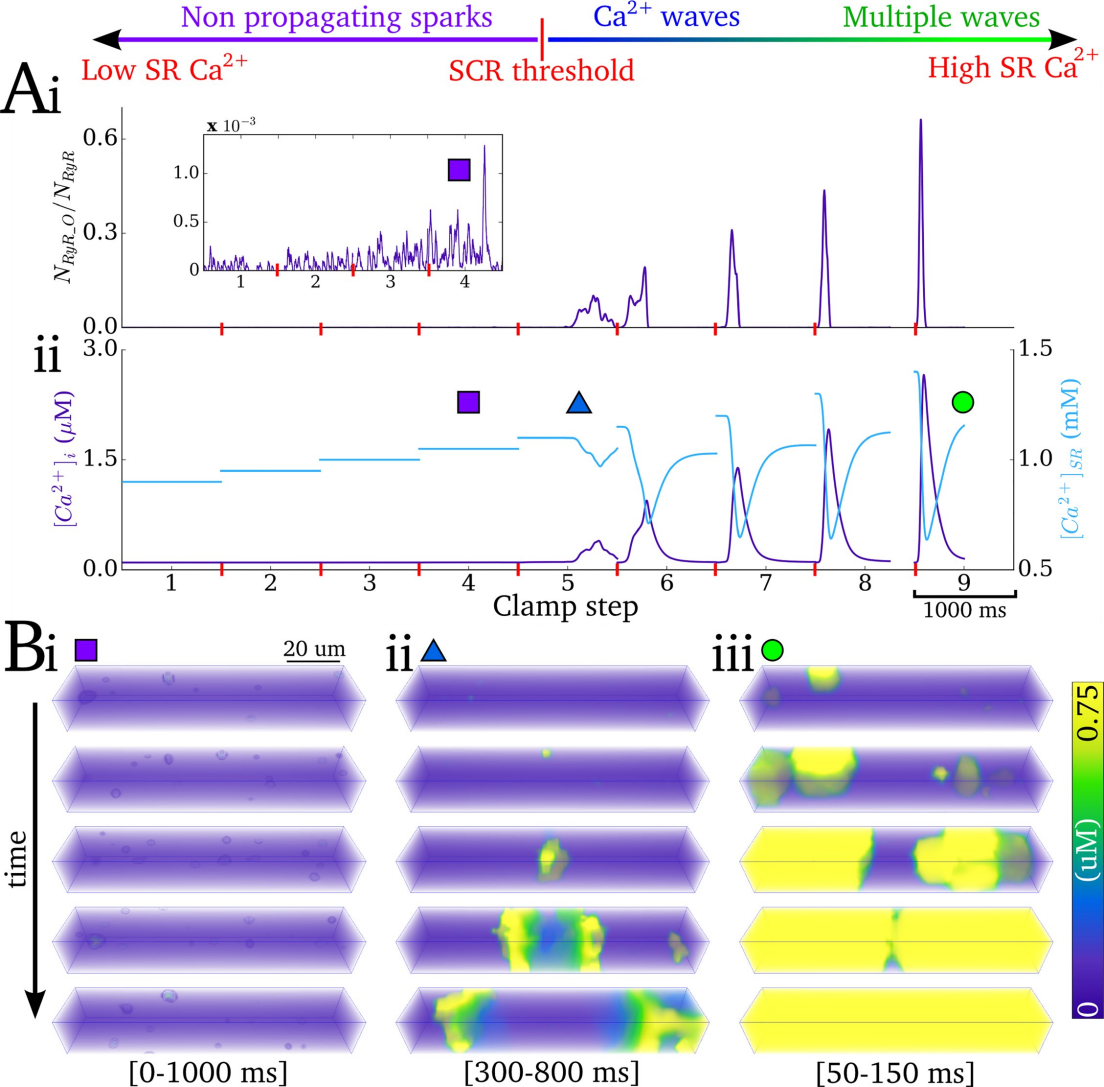
Illustration of Ca^2+^ clamp protocol. A–Ca^2+^ clamp protocol illustrated for 9 steps of SR-Ca^2+^, showing traces for: (i) proportion open RyR; (ii) intracellular- (purple) and SR- (blue) Ca^2+^ concentration. B–Snapshots of the spatio-temporal Ca^2+^ dynamics at different SR-Ca^2+^ concentrations, showing: (i) non-propagating sparks; (ii) slow Ca^2+^ wave; (iii) multiple and rapid Ca^2+^ waves; the time range for the snapshots is shown in the square brackets. The data shown are clipped to the first of the two seconds associated with each clamp step in order to clearly visualise the waveforms.

A partial Ca^2+^-clamp ladder protocol (Fig 2) was implemented to both derive and validate the SRF in the 0D model: At each step (total duration 2s), the intracellular- and SR-Ca^2+^ concentrations were initially clamped to specified values, with the SR-Ca^2+^ clamped concentration incrementing for each successive step (Fig 2Aii). When spontaneous intracellular release occurs, the Ca^2+^ concentrations were allowed to dynamically evolve (clamp constraint removed). The membrane potential was allowed to evolve during this protocol, and the conductances of *I*_Na_ and *I*_CaL_ were set to zero to prevent excitation and resulting interruption of the SCRE by CICR. This protocol illustrates the variety of SCRE and their underlying spatio-temporal dynamics which must be captured in the 0D model (Fig 2B; S1 Video).

### Derivation of the Spontaneous Release Functions (SRF)

**Fig 3:**
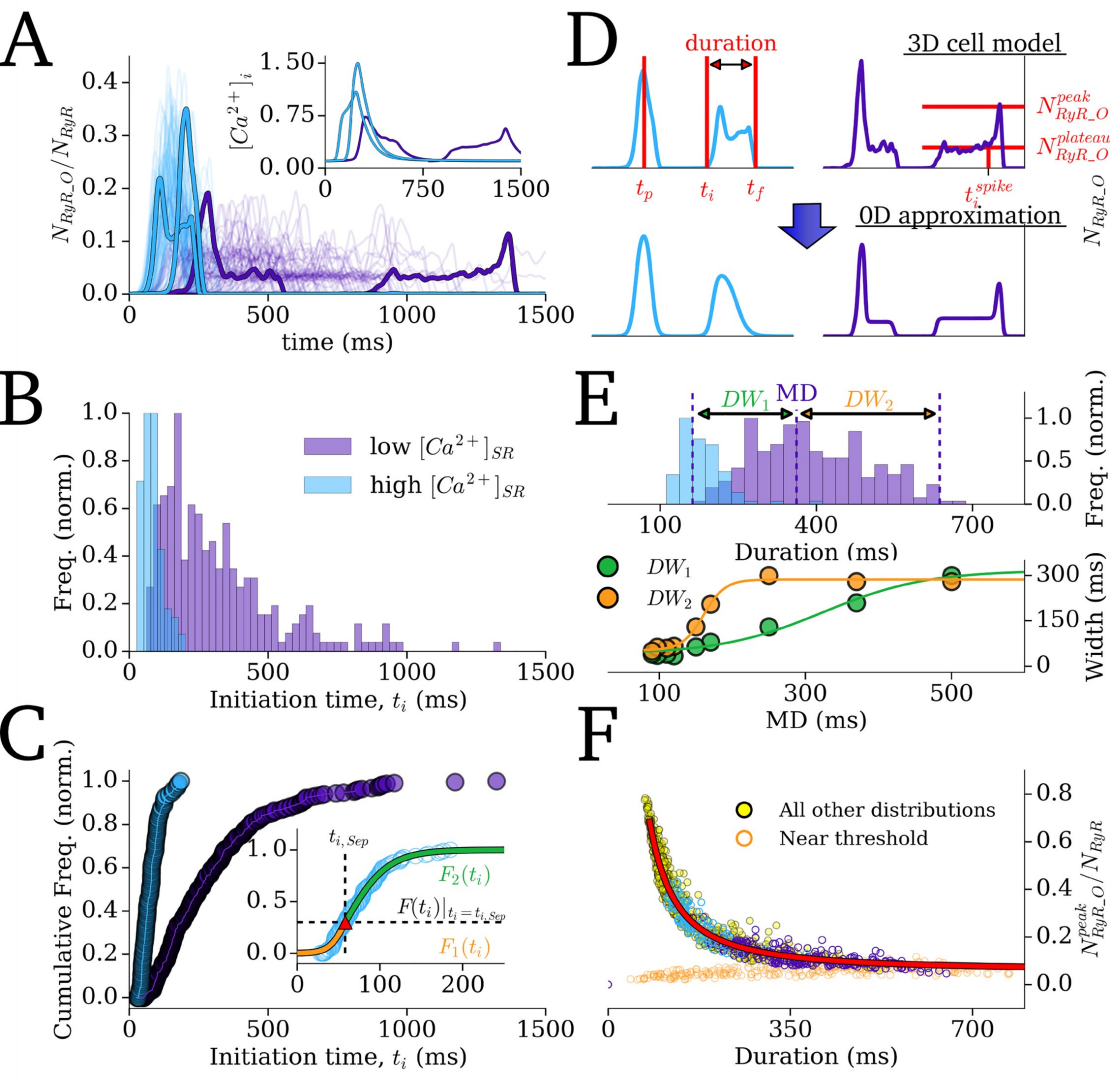
Derivation of the Spontaneous Release Functions. A–Traces of open RyR (*N*_RyR_O_/*N*_RyR_) associated with 250 simulations of SCRE at different SR-Ca^2+^ concentrations (low = 1125 mM, purple; and high = 1200 mM, blue; 100 traces for each condition shown) in the 3D cell model. Each trace represents an individual simulation, and two simulations are highlighted for each of the two SR-Ca^2+^ values (note the two blue traces overlap, but are separate simulations); A(inset)–spontaneous Ca^2+^ transients which correspond to the four highlighted RyR traces. B–Histogram describing the initiation time for SCRE and C–the corresponding cumulative frequency plots, associated with 250 simulations of each condition. Inset–example of fitting the cumulative frequency with two sigmoidal functions (*F*_1_(t_i_) and *F*_2_(t_i_), orange and green), separated at a specific point (*t*_i_Sep_, *F*(_ti_)|_ti = ti_Sep_, red triangular marker). D–Examples of two types of waveform, corresponding to those highlighted in A (upper panel) and the SRF which approximate them (lower panel). Labelled are the parameters which fully describe the waveforms: the initiation time (*t*_i_), peak time (*t*_p_), final time (*t*_f_), initiation time of spike during plateau (*t*_i_^spike^), peak open RyR (*N*_RyR_O_^peak^), plateau open RyR (*N*_RyR_O_^plateau^). E–Histogram illustrating the distribution of RyR waveform duration for the two conditions, with the median duration (*MD*) and width of distribution either side of the median (*DW*_1_, *DW*_2_) labelled for Distribution 1 (upper panel); relationship between the distribution widths and median (lower panel, points–data; lines–fit by Eqs (18 and 19)). F–Correlation between peak of open RyR and the duration, shown for the two conditions featured in the Figure (purple, blue) and all other simulations (yellow). Low amplitude SCRE occurring near the threshold SR-Ca^2+^ are shown in orange. Fit by Eq (12) is shown by the red line.

The phenomenological approach involved the development of SRF which describe whole-cell RyR dynamics associated with SCRE observed in the 3D cell model, based on an extension of previous work [21,22]. These functions are waveforms which approximate the range of morphologies for the time series of the whole-cell *N*_RyR_O_/*N*_RyR_ observed during SCRE (Fig 3A).

The concept of these SRF was introduced in a previous work [22] in the context of a statically defined model (corresponding to the Direct Control approach, below). This required the parameters describing the SRF to be defined by the user for specific simulations and therefore is not suitable for investigating natural cellular responses to different conditions or coupled dynamic simulations. Here, the functions themselves are refined, a method to derive the function parameters dynamically from model variables is introduced, leading to behaviour directly in-line with the 3D cell models, and the approaches are generalised to be fully controllable and suitable for parameterisation to experimental data. For completeness and context, the SRF are described here in full.

#### The Spontaneous Release Functions (SRF)

Results from the Ca^2+^ clamp ladder protocol across all SR-Ca^2+^ values were used to derive the analytical formulations of the SRF describing the variability in spontaneous release *N*_RyR_O_ waveforms. Comparison of SCRE from 250 simulations at low and high SR-Ca^2+^ illustrates the range of waveforms observed (Fig 3A) and the corresponding probability density function and cumulative frequency of initiation times, *t*_i_ (Fig 3B and 3C).

The *N*_RyR_O_ waveforms can be grouped into two primary types: spike-like associated with short, large-amplitude release, and plateau-like associated with long, small-amplitude release (Fig 3D). For the spike-like morphology, the waveform can be well approximated with the simple function:

$$N_{RyR\_O}=N_{RyR\_O}^{peak}{\left[\left(1+e^{-\left(t-t_{1}\right)/k_{1}}\right)\left(1+e^{-\left(t-t_{2}\right)/k_{2}}\right)\right]}^{-1}$$ (2)

$$t_{1}=t_{i}+0.5\left(t_{p}-t_{i}\right)$$ (3)

$$t_{2}=t_{p}+0.5\left(t_{f}-t_{p}\right)$$ (4)

$$k_{1}=0.1689(t_{p}-t_{i})+0.00255$$ (5)

$$k_{2}=0.1689\left(t_{f}-t_{p}\right)+0.00255$$ (6)

where *t*_*i*_ is the initiation time of the SCRE, *t*_f_ is the end time (duration, *λ*, thus = *t*_f_-*t*_i_), *t*_p_ is the time of the peak of the waveform and *N*_RyR_O_^peak^ is the peak of open proportion RyR (Fig 3D). The constants in Eqs (5 and 6) were obtained from best fits to the waveforms observed. The function for the plateau-like waveform (corresponding to durations longer than 300 ms) is derived from the same parameters:

$$\begin{array}{l} N_{RyR\_O}=\\ \;N_{{}_{RyR\_O}}^{plateau}{\left[\left(1+e^{-\left(t-(t_{i}+17.5)\right)/5.946}\right)\left(1+e^{\left(t-(t_{f}-17.5)\right)/5.946}\right)\right]}^{-1}+\\ \;\left(N_{{}_{RyR\_O}}^{peak}-N_{{}_{RyR\_O}}^{plateau}\right){\left[\left(1+e^{-\left(t-(t_{p}-25)\right)/5.946}\right)\left(1+e^{\left(t-(t_{p}+17.5)\right)/5.946}\right)\right]}^{-1} \end{array}$$ (7)

Where *N*_RyR_O_^plateau^ is the amplitude of the plateau (Fig 3D). This equation assumes the same form for the spike occurring within the plateau, with its upstroke time being 50 ms and its decay time 35ms; *t*_i_^spike^ (Fig 3D) therefore corresponds to *t*_p_-50 (and its half maximal activation time *t*_p_-25). The constants were similarly obtained from the best fits to the waveforms observed.

The waveform is therefore completely described by four or five parameters (for spike-like and plateau-like waveforms, respectively): (1) initiation time, *t*_i_; (2) duration (*λ = t*_f_-*t*_i_); (3) peak time, *t*_p_; and (4–5) amplitude (*N*_RyR_O_^peak^; *N*_RyR_O_^plateau^). In order to maintain physiological waveforms and randomly sample the parameter values from appropriate distributions, the nature of stochastic variation of these four parameters must be examined, as is discussed below.

#### Parameter distributions and inverse functions

(1)—*t*_i_: The probability density functions for the initiation time associated with each SR-Ca^2+^ value exhibit skewed distributions (Fig 3B). The cumulative frequency (Fig 3C) was well approximated by the use of two simple sigmoidal functions (Fig 3C-inset), maintaining the desire for restraint in the number of parameters and allowing simple and intuitive controllability:

$$\begin{array}{l} F(t_{i})=\\ \;F_{1}\left(t_{i}\right)=\left(2CF_{t_{i,Sep}}\right){\left(1+e^{-\left(t_{i}-t_{i,sep}\right)/k_{F_{1}}}\right)}^{-1}\\ \;F_{2}\left(t_{i}\right)=\left(2\left(1-CF_{t_{i,Sep}}\right)\right){\left(1+e^{-\left(t_{i}-t_{i,sep}\right)/k_{F_{2}}}\right)}^{-1}-1+2CF_{t_{i,Sep}} \end{array}\}\begin{array}{c} t_{i}<t_{i,Sep}\\ t_{i}\geq t_{i,Sep} \end{array}$$ (8)

**Fig 4:**
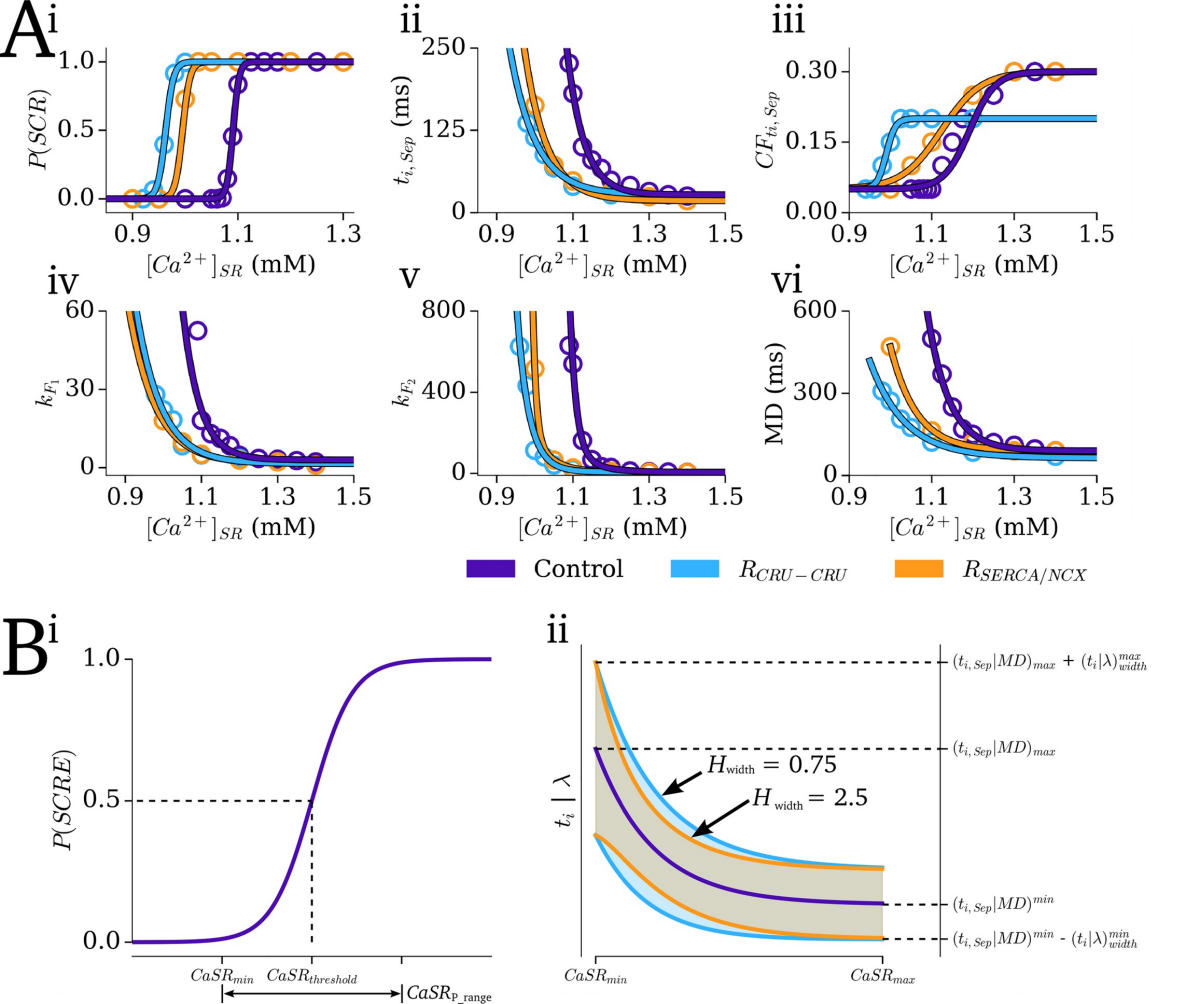
SR-Ca^2+^ dependence of SRF distribution parameters. A–The Dynamic Fit SRF parameters: summary data (points) and the fit from the relevant functions (lines) for the three Ca^2+^ handling conditions (purple–control; blue–CRU-CRU coupling enhancement; orange–SERCA upregulation and NCX downregulation model) against SR-Ca^2+^ for: i) probability of whole-cell SCRE; ii) initiation time corresponding to the separation point, *t*_i_; iii) the normalised cumulative frequency at this point, *CF*_ti,Sep_ = *F*(_ti_)|_ti = ti,Sep_; iv) the *k* parameter for *F*_1_(t_i_) and (v) for *F*_2_(t_i_); (vi) the median duration, *MD*. B–The General Dynamic SRF parameters: i) Illustration of the curve for probability of SCRE and its relation to the user defined parameters (*CaSR*_thresdhold_, *CaSR*_P_range_) and derived parameters (*CaSR*_min_). ii) Illustration of the function form describing *t*_i,Sep_ and median duration (*MD*) (purple line) and its relation to *CaSR*_min_, *CaSR*_max_, *t*_i,Sep_^min^, *t*_i,Sep_^max^, *MD*^min^, *MD*^max^, and how the range of the distributions varies with SR-Ca^2+^ (shaded regions) at two different non-linearity factors (*H*_width_ = 0.75, blue; = 2.5, orange).

The distribution for *t*_i_ is therefore determined by four parameters: the initiation time corresponding to the point where the functions are separated (*t*_*i*,*Sep*_); the cumulative frequency at this point (*CF*_ti,Sep_ = *F*(*t*_i_)|_ti = ti,Sep_), and the gradient parameter of each function (*k*_F1_, *k*_F2_—corresponding to the width of the distribution either side of *t*_i,Sep_; Fig 4C-inset.)

(2)–*λ*: The distributions for the duration are also non-normal, and well approximated by two sigmoidal functions describing the cumulative frequency for half of the data either side of the median duration (*MD*; Fig 3E):

$$\begin{array}{l} F(MD)=\\ \;F_{D1}(MD)={\left(1+e^{-(\lambda -MD)/0.261DW_{1}}\right)}^{-1}\\ \;F_{D2}(MD)={\left(1+e^{-(\lambda -MD)/0.261DW_{2}}\right)}^{-1} \end{array}\}\begin{array}{c} \lambda <MD\\ \lambda \geq MD \end{array}$$ (9)

Where the widths (*DW*_1_, *DW*_2_, in ms) are a function of the *MD* (Fig 3E), given by:

$$DW_{1}=A_{DW1}{\left(1+e^{-\left(MD-a_{DW1}\right)/k_{DW1}}\right)}^{-1}+DW_{1}^{\min}$$ (10)

$$DW_{2}=A_{DW2}{\left(1+e^{-\left(MD-a_{DW2}\right)/k_{DW2}}\right)}^{-1}+DW_{2}^{\min}$$ (11)

Default parameters, fit to the waveforms observed in the control model, are given in S1 Text (Model Description). The duration distribution for each model condition (control and remodelling models) is therefore completely described by one variable, the median, ***MD***, and the above model-specific parameters. Note that the widths (***DW***_**1**_**, *DW***_**2**_) could also be specified directly for complete control over the variability in duration.

(3)—*t*_p_: The timing of the peak varies approximately evenly within the duration of the wavefrom, occurring between 25 ms after the initiation (*t*_i_) and 52 ms before the final time (*t*_f_).

(4)—*N*_RyR_O_^peak^; *N*_RyR_O_^plateau^: The expectation value of the amplitude correlates strongly with duration, λ (Fig 3F):

$$\langle N_{RyR\_O}^{peak}\rangle =692.99\lambda^{-1.6}+0.059$$ (12)

$$\langle N_{RyR\_O}^{plateau}\rangle =31.09{\left(0.01\lambda \right)}^{-7.39}+0.034\}if\;\lambda >300ms$$ (13)

With uniform variation around these expectation values. Note that it would not be appropriate to define the amplitude independently from the duration, due to the correlation between these two parameters corresponding to the total amount of Ca^2+^ released.

With this setup, therefore, all parameters of the waveform are derived from two primary waveform properties: the initiation time, *t*_i_, and the duration, *λ*, which also determine the peak time and amplitude. The distributions describing the variability of these properties are entirely described by 5–7 parameters (*t*_i_ = *f*(*t*_i_sep_, *CF*_ti_sep_, *k*_F1_, *k*_F2_); *λ* = *f*(*MD*, *DW*_1_, *DW*_2_) where *DW*_1_, *DW*_2_ = *f*(*MD*) or manually specified). Producing a single SRF waveform therefore requires only that *t*_*i*_ and *λ* are sampled from the relevant distributions, by passing a random number between 0 and 1 into the corresponding inverse functions:

Initiation time, *t*_i_; inverse function of Eq (8):

$$\begin{array}{l} t_{i}=\\ \;-k_{F_{1}}.\ln\left(\frac{2CF_{t_{i,Sep}}}{rand}-1\right)+t_{i\_sep}\\ \;-k_{F_{2}}.\ln\left(\frac{2\left(1-CF_{t_{i,Se[}}\right)}{rand+1-2CF_{t_{i,Sep}}}-1\right)+t_{i\_sep} \end{array}\}\begin{array}{c} rand<CF_{t_{i,Sep}}\\ rand\geq CF_{t_{i,Sep}} \end{array}$$ (14)

Duration, *λ*; inverse function of Eq (9):

$$\begin{array}{l} \lambda =\\ \;0.261DW_{1}.\ln\left(rand^{-1}-1\right)+MD\\ \;0.261DW_{2}.\ln\left(rand^{-1}-1\right)+MD \end{array}\}\begin{array}{c} rand<0.5\\ rand\geq 0.5 \end{array}$$ (15)

And the derived variables, *t*_p_ and *N*_RyR_O_^peak^, *N*_RyR_O_^plateau^, can be randomly sampled from uniform distributions:

$$t_{p}=25+rand\left(\lambda -52\right)+t_{i}$$ (16)

$$N_{RyR\_O}^{peak}=\langle N_{RyR\_O}^{peak}\rangle +0.05(rand-0.5)$$ (17)

$$N_{RyR\_O}^{plateau}=\left(1+0.25(rand-0.5)\right)\langle N_{RyR\_O}^{plateau}\rangle$$ (18)

The different implementations of the SRF therefore pertain to different methods to define the distributions from which the SRF parameters will be sampled, as well as the probability of release, *P(SCR)*.

#### Sampling the parameters: The direct control SRF model

For the simplest implementation of SCRE in 0D models, the user “Direct Control” model, SRF variability and morphology can therefore be described simply by explicitly defining the 5–7 parameters which describe the *t*_*i*_ and *λ* distributions as desired (for example, to fit a single dataset, or for controlled investigation), and then sampling from the resulting inverse functions on a beat-by-beat basis. The next section describes an approach to derive these parameters dynamically based on relevant Ca^2+^ handling environment variables.

#### Sampling the parameters: The dynamic fit SRF model

The Dynamic Fit SRF model was derived through correlation of the parameters defining the *t*_i_ and *λ* distributions with the primary environment variable controlling SCRE, the SR-Ca^2+^ concentration (Fig 4A; S1 Text (Model Description)). Relation to this single variable was chosen for practicality and simplicity of the resulting equations. Other factors which affect SCRE dynamics (e.g. RyR dysfunction, remodelling of SERCA/NCX, changes in diastolic Ca^2+^) can then be indirectly accounted for by their impact on the SR-Ca^2+^ dependence; using these simple functions, the coefficients which fit the data from the two remodelling conditions (which both directly affected SCRE dynamics) could also be obtained (Fig 4A; S1 Text (Model Description)). Note that both remodelling models reduce the SR-Ca^2+^ threshold for SCRE compared to control. For R_CRU-CRU_, this is a direct result of the reduced time constant of sub-space coupling; for R_SERCA/NCX_, it is primarily the reduced Ca^2+^ efflux due to the lower expression of NCX which causes this shift, by more easily allowing spontaneous Ca^2+^ sparks to propagate to neighbouring CRUs.

#### Sampling the parameters: The general dynamic SRF model

Whereas the Dynamic Fit SRF model described above ensures congruence between the 3D and 0D models, a General Dynamic SRF model was also derived which provides full control over the distributions determining the dynamic behaviour. The approach therefore involved defining the functions which correlate SR-Ca^2+^ with the SRF input parameters (*P*(SCR), *t*_i_sep_, *CF*_ti,Sep_, *k*_F1_, *k*_F1_, *MD*) based on intuitive controllable parameters (Fig 4B): (i)—The threshold for SCRE (*CaSR*_threshold_); (ii)—The SR-Ca^2+^ range over which *P*(SCR) varies from 0 to 1 (*CaSR*_P_range_); (iii)—The maximal SR-Ca^2+^ above which SCRE distributions converge (*CaSR*_max_ > *CaSR*_threshold_ + *CaSR*_P_range_); (iv)—The minimum and maximum *t*_i,Sep_ and *MD* (*t*_i,Sep_^min^, *t*_i,Sep_^max^, *MD*^min^, *MD*^max^); (v)—The *t*_i_ and *λ* distribution widths at these extremes (*t*_i,width_^min^, *t*_i,width_^max^, *λ*_width_^min^, *λ*_width_^max^); And (vi)—the non-linearity of width variance (*H*_width_); *CF*_ti,Sep_ was set to 0.4. Equations are given in S1 Text (Model Description).

#### Implementation with the 0D cell model

**Fig 5:**
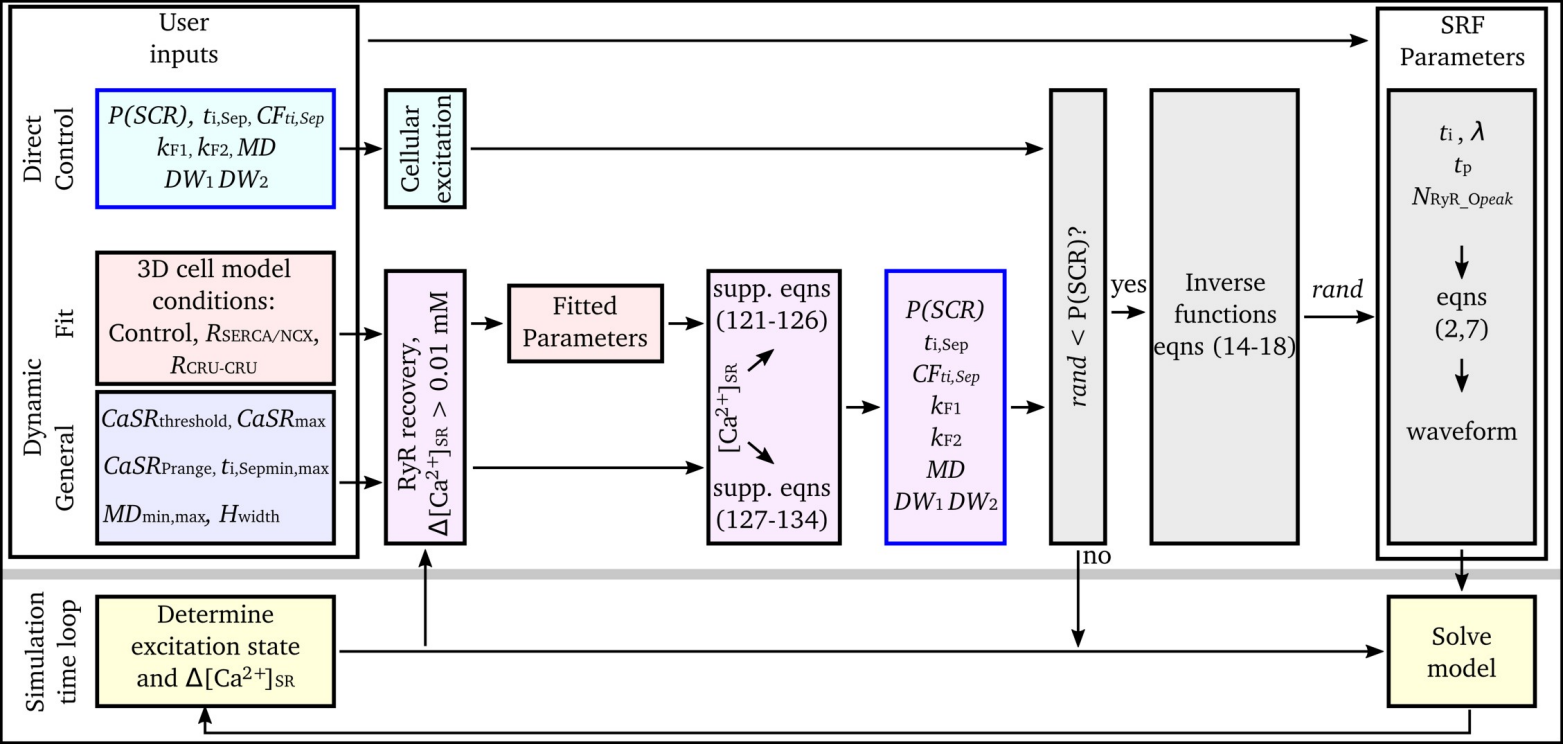
Implementation algorithm. Schematic of the algorithms used to integrate the SRF with the non-spatial cell models for the three implementations. Note that the Δ[Ca^2+^]_SR_ clause is only calculated if the SRF parameters have been set but SCRE has not yet been initiated, and RyR recovery is only calculated if the model has undergone an AP.

These SRF were implemented within the 0D cell models using a simple algorithm (Fig 5): the input parameters (*P*(SCR), *t*_i,Sep_, *CF*_ti,Sep_, *k*_F1_, *k*_F2_, *MD*; see previous section: Parameter distributions) are defined at a certain time (see below) and then the waveform parameters (*t*_i_, and *λ*, which in turn define *t*_p_ and *N*_RyR_O_^peak^; see previous section: Parameter distributions) are randomly sampled from the associated inverse functions (Eqs 14–18 above). When the SRF have been initiated (i.e., *t*_i_ < *t* < *t*_i_ + *λ* and *N*_RyR_O_^SRF^ is > 0), the *N*_RyR_O_ in the cell model is set to *N*_RyR_O_^SRF^ and the model thus evolves as if the equivalent SCRE was occurring in the 3D cell model.

For the simplest implementation of the SRF, the Direct Control model, this calculation is determined at the time of cellular excitation, setting *t*_i_ and *λ* based on the distributions defined by user input parameters (*P*(SCR), *t*_i,Sep_, *CF*_ti_sep_, *k*_F1_, *k*_F2_, *MD*) and five random numbers input into the corresponding inverse functions. The model will set SRF parameters based on these single distributions with every cellular excitation (note that the parameters may give an SCRE timing later than the next stimulated excitation, in which case it will not occur and be reset on the next excitation).

For the Dynamic Fit and General Dynamic models, the calculation is performed multiple times, dynamically determined during the simulation: Initially, after the cell model has undergone an AP and the RyR availability has recovered above a set threshold (inactivation state-occupancy reaches below 0.2), the SR-Ca^2+^ is input to first define the probability of release, *P(SCR)*, from S1 Text (Model Description)–equations (121; 128), and then the remaining input parameters are defined from the SR-Ca^2+^ according to the appropriate functions [S1 Text (Model Description)–equations (122–126; 127–134)]. Once these parameters have been calculated, if the SR-Ca^2+^ concentration changes more than by a predefined value (0.01 mM) before the SCRE has been initiated, then the parameters are recalculated based on this new SR-Ca^2+^. SRF parameters are not calculated during excitation (i.e., when the RyR availability is low) but recalculated upon RyR recovery, to impose that the SRF do not interrupt deterministic CICR.

### Simulation protocols

#### Rapid pacing protocol

To study the emergence of triggered activity in single cell and tissue, a rapid pacing protocol was applied to promote SR-Ca^2+^ loading and the emergence of whole-cell SCRE: The models were paced to stable state under varying basic cycle lengths (BCL = 200–600 ms); the state variables were saved at the stable values and used as initial conditions to efficiently run any number of simulations of a short pacing period followed by a quiescent (non-applied pacing) period, within which the statistics of SCRE can be analysed. This protocol was applied to the 3D and 0D single cell models (implementing the Dynamic Fit SRF approach) to compare behaviour between these models and translate cell-type and model-condition differences to the tissue-scale.

#### Assessing the relationship between SR-Ca^2+^ and focal excitation

The General Dynamic SRF implementation was used to assess the relationship between SR-Ca^2+^ and focal excitation in tissue, allowing simulation of both homogeneous and heterogeneous media with respect to SCRE properties (electrical heterogeneity, pertaining to the different cell-types, was not included in this analysis). A baseline SRF model was set with an SR-Ca^2+^ threshold for SCRE of 1.10 mM. Four alternative models were introduced with this SR-Ca^2+^ threshold varied by ± 0.05 and 0.1 mM. All other parameters are either identical or set relative to the threshold (S1 Text (Model Description)). Note that at a specific SR-Ca^2+^ concentration, implementations with a lower SR-Ca^2+^ threshold exhibit larger amplitude and more rapidly timed SCRE than those with a higher SR-Ca^2+^ threshold. A small amount of heterogeneity was first introduced by randomly assigning 60% of the cells to the baseline, and 10% to each of the four other distributions; a larger amount of heterogeneity was introduced by randomly assigning 20% of the cells to each of the five distributions. Note that this heterogeneity is designed to represent normal inter-cellular variability, rather than heterogeneous patches associated with disease remodelling, and is therefore implemented to preserve the mean for direct comparison to the homogeneous case.

#### Studying unidirectional conduction block

Two potential mechanisms of SCRE-induced unidirectional excitation conduction block, which presents the possibility to degenerate into re-entry, have been shown in the studies of Liu et al., 2015 [25] and Campos et al., 2017 [20]: a well-established mechanism, in which focal excitation (in this instance as a result of SCRE) interacts with heterogeneity in refractory period and leads to asymmetric propagation patterns. And a novel mechanism by which DADs in combination with sodium channelopathy led to heterogeneous inactivation of the fast sodium current and conduction block. To demonstrate the capability of the SRF implementation to reproduce these dynamics, and to provide independent verification of the results in those previous studies, two protocols were used: (i) DAD mediated conduction block was simulated in a homogeneous 2D sheet of the epicardial layer of the ventricle in combination with impaired sodium current (conductance of *I*_Na_ scaled by 30%; 5 mV shift of the inactivation steady-state). These conditions were combined with SRF parameters which were below the threshold for focal excitation, but in which significant DADs were observed; (ii) Conduction block due to focal excitation and repolarisation heterogeneity was simulated in a 2D sheet model of the transmural cell-types in the ventricular wall (simple 1:1:1 ratio of EPI, M and ENDO cells), with the pre-pacing stimulus applied uniformly to the edge of the ENDO region; a small region was set to reduced *I*_K1_ to promote localized excitation. The Direct Control implementation was used to impose early-timed and controllable SCRE.

#### Re-entry and SCRE interactions

To study the long-term interactions of re-entry and SCRE, it was required to initiate a transient period of re-entrant excitation, and then for this to terminate to allow the emergence of SCRE. Re-entrant excitation was initiated using two different methods: the phase-distribution method [39,40], and cross-field stimulation. In general, a reduction in the conduction velocity (***D*** reduced by up to half) and/or shortening of the AP (increase in *I*_Kr_ and/or *I*_Ks_) was also employed to shorten the wavelength and promote sustained excitation. Different modifications were required by different cell models and conditions in order to lead to this complex excitation; it should be noted, however, that the aim of these studies was to determine how re-entry may promote SCRE, rather than to study the dynamics of re-entry itself. In many cases, re-entry self-terminated within 20s, representing natural self-termination of a transient re-entrant event. In other cases, a block of *I*_Kr_ or an increase in ***D*** (back to control values) was necessary to terminate re-entry which would otherwise sustain–the required termination mechanism also depended highly on the underlying cell model and conditions, and was not the focus of the simulations. These simulations were combined with the General Dynamic SRF model to investigate the possible influence of re-entry under different vulnerabilities to SCRE: simulations associated with multiple re-entrant conditions were analysed under multiple SRF parameter combinations. A rapid regular pacing protocol was also applied to match SR-Ca^2+^ loading as observed in different re-entrant cases, to study the potential differences between behaviour emerging from the two different loading mechanisms under comparable environmental conditions.

## Results

In this section is first described validation of the Dynamic Fit SRF approach for the three Ca^2+^ system conditions before application of the framework, in order to: (i) demonstrate the mechanism of stochastically mediated SCRE-induced ectopic beats; (ii) examine the complexities underlying the SR-Ca^2+^ dependence of focal excitation; (iii) demonstrate two independent mechanisms of conduction block associated with SCRE; and (iv) study the potential multi-scale, pro-arrhythmic interactions between SCRE and re-entrant excitation.

### Validation of the dynamic fit spontaneous release functions

The 0D model implementing the Dynamic Fit SRF model was first validated by comparison of whole-cell SCRE under Ca^2+^ clamp conditions with a second set of simulations (i.e., not those on which the model was derived) of the 3D cell model, for the control and remodelling conditions (S2 Text (Validation and Results)). These simulations highlight the strong agreement for waveform morphology and its variation, the distributions and their summary properties, and the differences between the control and remodelled conditions.

**Fig 6:**
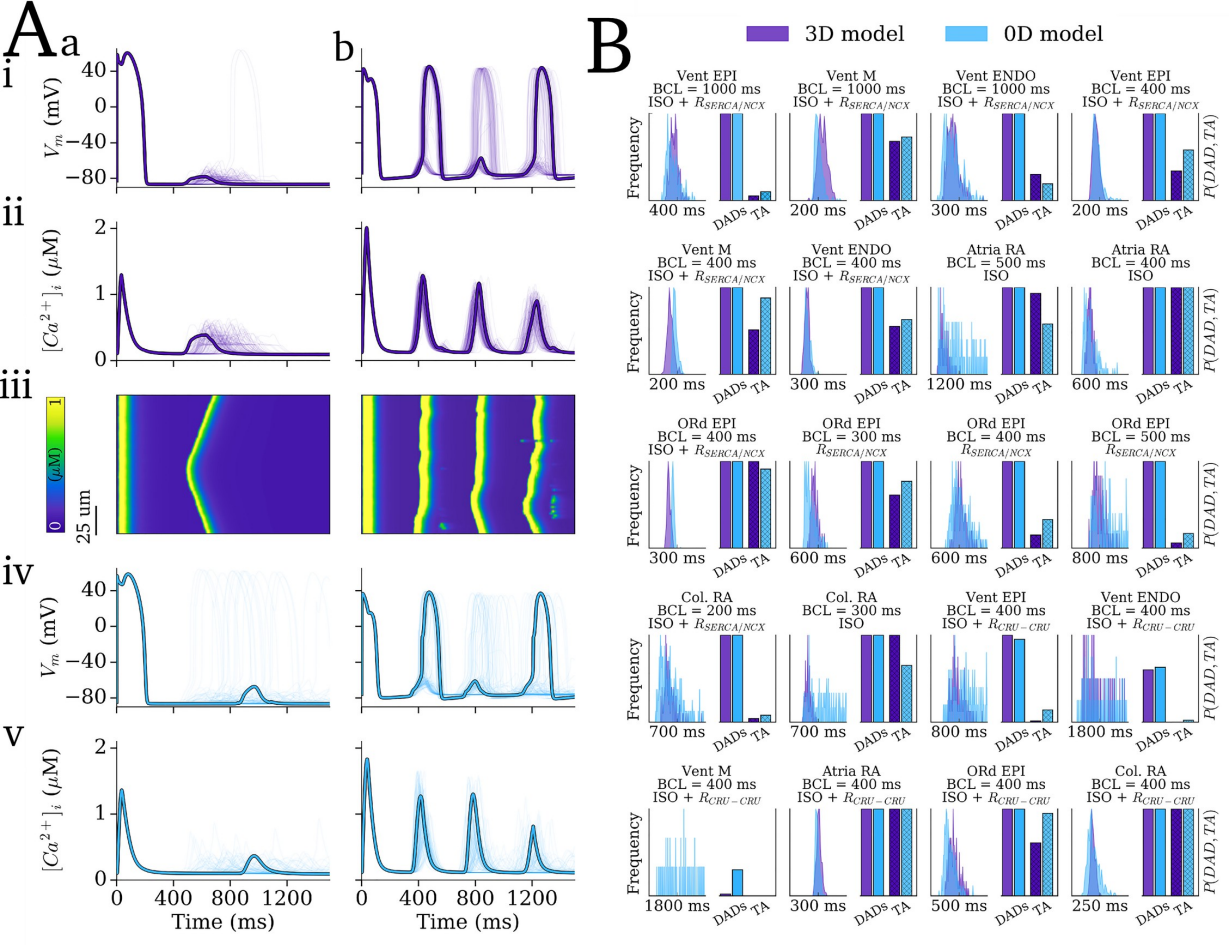
Validation of the SRF under dynamic pacing conditions. Results of 250 simulations for 20 different pacing conditions in which notable SCRE occurred. A– 100 examples (with one highlighted) of SCRE occurring in the 3D model (purple, i-iii) and 0D model (blue, iv-v) for two different conditions which resulted in SR-Ca^2+^ close to threshold (a, corresponding to Vent EPI, BCL = 400 ms, *R*_CRU-CRU_) and above it (b, corresponding to Vent ENDO, BCL = 400 ms, ISO + *R*_SERCA/NCX_). The linescans in (iii) correspond to the highlighted trace in (i-ii). Final paced beat and subsequent quiescent period is shown. B–Histograms of SCRE initiation time (left of each panel) and incidence of DADs and TA (bars, right of each panel) for the 20 different conditions (panel titles correspond to cell model, pre-pacing BCL and pro-SCRE conditions); the x-axis label for the histogram plots refers to the total range over which the plot is shown, rather than absolute values. Col. RA refers to the simplified Colman et al. 2013 [27] human atrial model; ORd refers to the simplified O’Hara et al. [26] human ventricular cell model; all other labels refer to the cell-type used in the hybrid minimal model presented in this study.

Secondly, the 0D and 3D models were compared across the range of ionic models (see Methods: Action Potential and Tissue Models) and remodelling/ISO conditions (see Methods: Pro-arrhythmic conditions). Examples of dynamics emerging in conditions close to the SR-Ca^2+^ threshold (Fig 6Aa) and high above it (Fig 6Ab) show good agreement between the 3D and 0D models (compare Fig 6Ai-ii with 6Aiv-v), importantly capturing the key features and differences between the conditions. The case for low SR-Ca^2+^ (Fig 6Aa) was intentionally selected as one of those in which the match was the poorest, in order to fully illustrate the quality of the approximation in an upfront manner; even here, the match is reasonable, and the main features of the behaviour are preserved (DADs with some TA; wide initiation time distribution). The distributions of initiation time and the probability of triggered APs across all conditions tested which resulted in notable SCRE also show good agreement (Fig 6B), confirming the ability of the 0D models to dynamically reproduce the SCRE of the 3D cell models and capture cellular and condition dependent behaviour. Note that the differences between the cell-types and conditions is not of primary interest, but, rather, it is the match between 3D and 0D models, including the reproduction of these differences, which is of interest, with a key feature being the probability of TA or DADs.

### Emergence of focal excitation and translation of model differences

The potential for the models to simulate SCRE at the tissue and organ scale was illustrated through pacing tissue models under an equivalent rapid pacing–quiescent protocol to the single cells (see Methods: Simulation Protocols). Under the right conditions (i.e., significant SR-Ca^2+^ loading and thus large-scale release events) a triggered action potential emerged from a single focus and propagated throughout the tissue, observed in multiple tissue models (S2 Text (Validation and Results)). Multi-focal activations were also observed.

**Fig 7:**
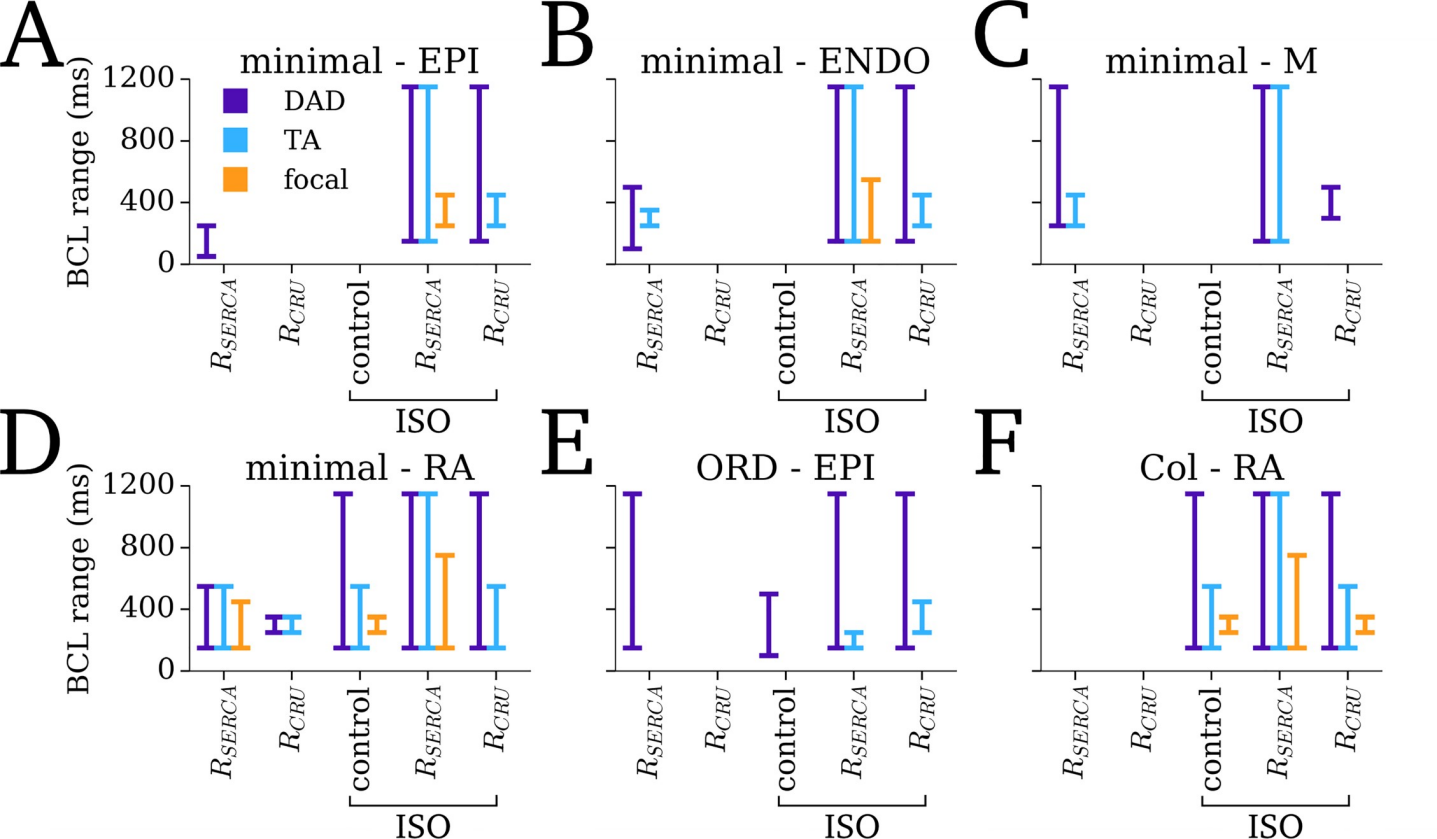
The emergence of SCRE at the tissue scale in different models. The BCL range over which activity corresponding to single cell DADs (purple) and TA (blue) and tissue focal excitation (orange) is shown for each of the different cell models/regions (A-F) under different conditions (*x-axis* labels–remodelling, and control and remodelling + ISO). The BCL range for which at least one SCRE/TA/focal-excitation occurs is indicated by the extent of the lines. Note that no significant SCRE was observed for control conditions for any model, and so this condition has not been included in the figure.

The different SCRE dynamics observed across the range of 3D single cells models under pro-SCRE conditions (Fig 6B) was accentuated in tissue, wherein focal excitations only emerged under conditions which resulted in significant TA (Fig 7). Note also the important impact of reduced *I*_K1_, present in the atrial cell models and *R*_SERCA_NCX_ remodelling conditions, on allowing the emergence of tissue focal excitation.

### Mechanisms of synchronisation of focal excitation

**Fig 8:**
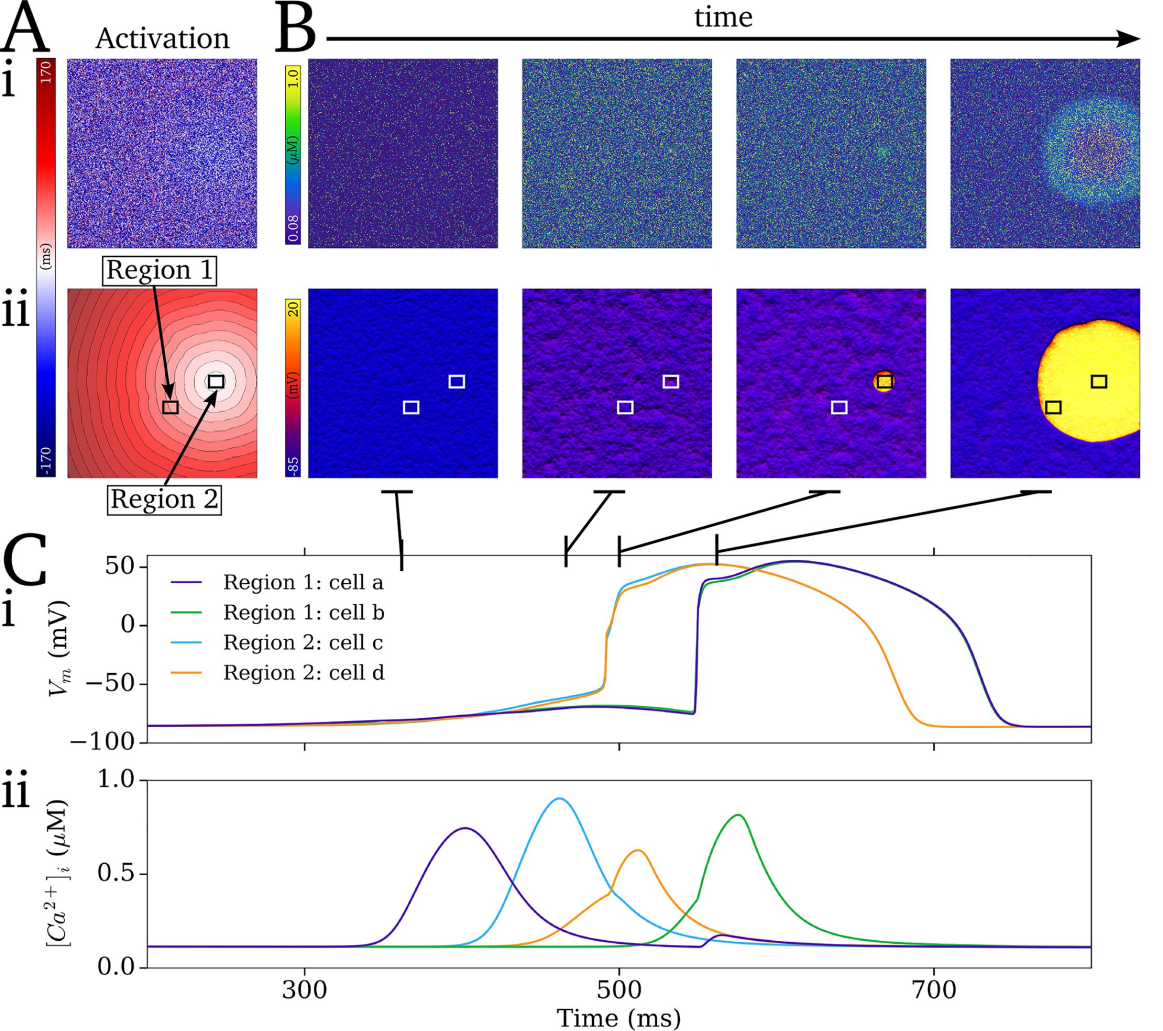
The role of electrotonic coupling in overcoming source-sink mismatch. A–Activation maps for intracellular Ca^2+^ (i) and membrane potential (ii) associated with a spontaneous focal excitation. Note that the time of the initiation of focal excitation (t = 0 ms) corresponds to the halfway point of the colour map. B–Temporal snapshots of Ca^2+^ (i) and *V*_m_ (ii) associated with the onset of focal excitation. C–*V*_m_ (i) and Ca^2+^ (ii) traces from individual cells from two regions with the tissue (labelled in Aii) illustrating the independent (Ca^2+^) and coupled (*V*_m_) cellular behaviour.

Evaluation of the focal activation emerging in the 2D sheet illustrates the mechanism by which these small scale cellular events result in full tissue excitation and the dual role of electrotonic coupling (Fig 8; S2 and S3 Videos): SCRE occurring in independent cells initially causes only a small depolarisation of the cell membrane, repressed by electrotonic coupling (Fig 8C, purple trace); however, this depolarisation is also electrotonically spread to surrounding cells (Fig 8B); due to this depolarised resting potential, SCRE occurring later can act to further depolarise the surrounding tissue (Fig 8C, blue trace); once local tissue is sufficiently depolarised, the probability of DADs manifesting as TA significantly increases and appropriately-timed firing cells can much more easily initiate a focal excitation (Fig 8C, orange trace).

### Inter-cellular variability and the SR-Ca^2+^ dependence of ectopic activity

**Fig 9:**
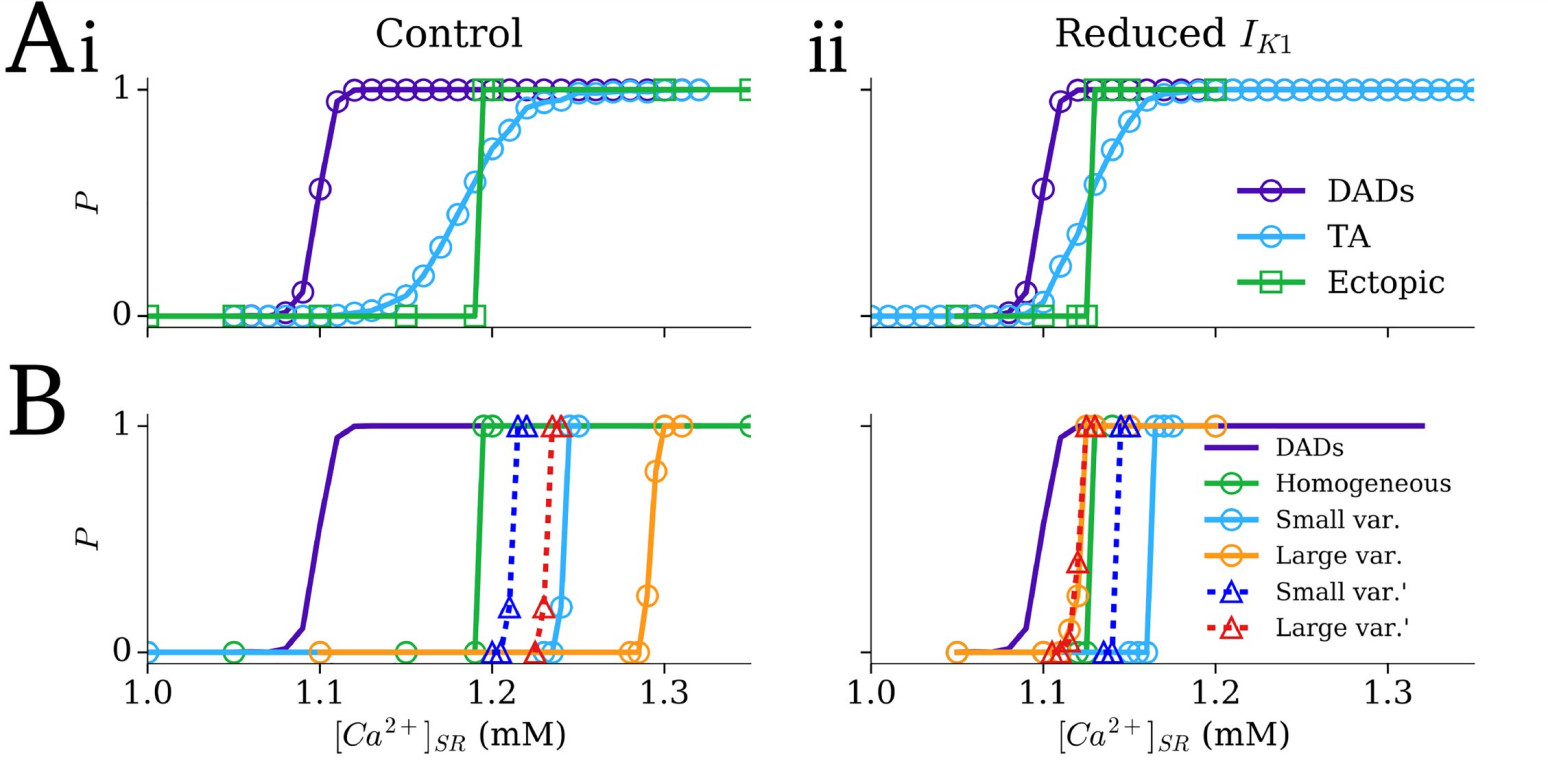
The effect of SCRE heterogeneity on the SR-Ca^2+^-TA relationship. A–Dependence of single cell DADs (purple), single cell TA (blue), and ectopic focal activity in 2D tissue (green; square markers) on the SR-Ca^2+^ concentration in control (i) and reduced *I*_K1_ conditions (ii); homogeneous SCRE dynamics. B–Dependence of ectopic focal activity in tissue on SR-Ca^2+^ in the heterogeneous SRF conditions with small and large variability (blue and orange) and with small and large variability with higher SR-Ca^2+^ threshold cells reassigned to baseline (blue and red; triangular markers) compared to the homogeneous condition (green); single cell DADs in the baseline condition are shown for reference (purple).

In homogeneous tissue, using a baseline General Dynamic SRF model parameter set (see Methods: Simulation Protocols), the probability curve for ectopic activity was substantially steeper than that for the emergence of TA from DADs in single cell (Fig 9Ai). Reduction of the density of *I*_K1_ shifted both the single cell and tissue TA probability curves to the left (lower SR-Ca^2+^), towards the curve describing DADs (Fig 9Aii); however, the steep relationship in tissue remains.

With control *I*_K1_ density, both heterogeneity models (involving either 10% or 20% of cells randomly allocated to each of the additional four General Dynamic SRF models with different SR-Ca^2+^ thresholds) shifted the SR-Ca^2+^ relationship to the right (higher SR-Ca^2+^ concentrations), despite the presence of cells more susceptible to SCRE. With reduced *I*_K1_ density, the small heterogeneity model still shifted the relationship to the right, but the large heterogeneity model now shifted it to the left (Fig 9B). In general, the introduction of heterogeneity reduced the steepness of the probability curve at low probabilities (Fig 9B). Heterogeneity models with the less vulnerable cells removed (higher SR-Ca^2+^ threshold cells reassigned to baseline) produced the same overall features: three cases still shifted to the right despite the presence of more pro-SCRE cells and absence of less vulnerable cells.

### Spontaneous Ca^2+^ release as a mechanism for conduction block

**Fig 10:**
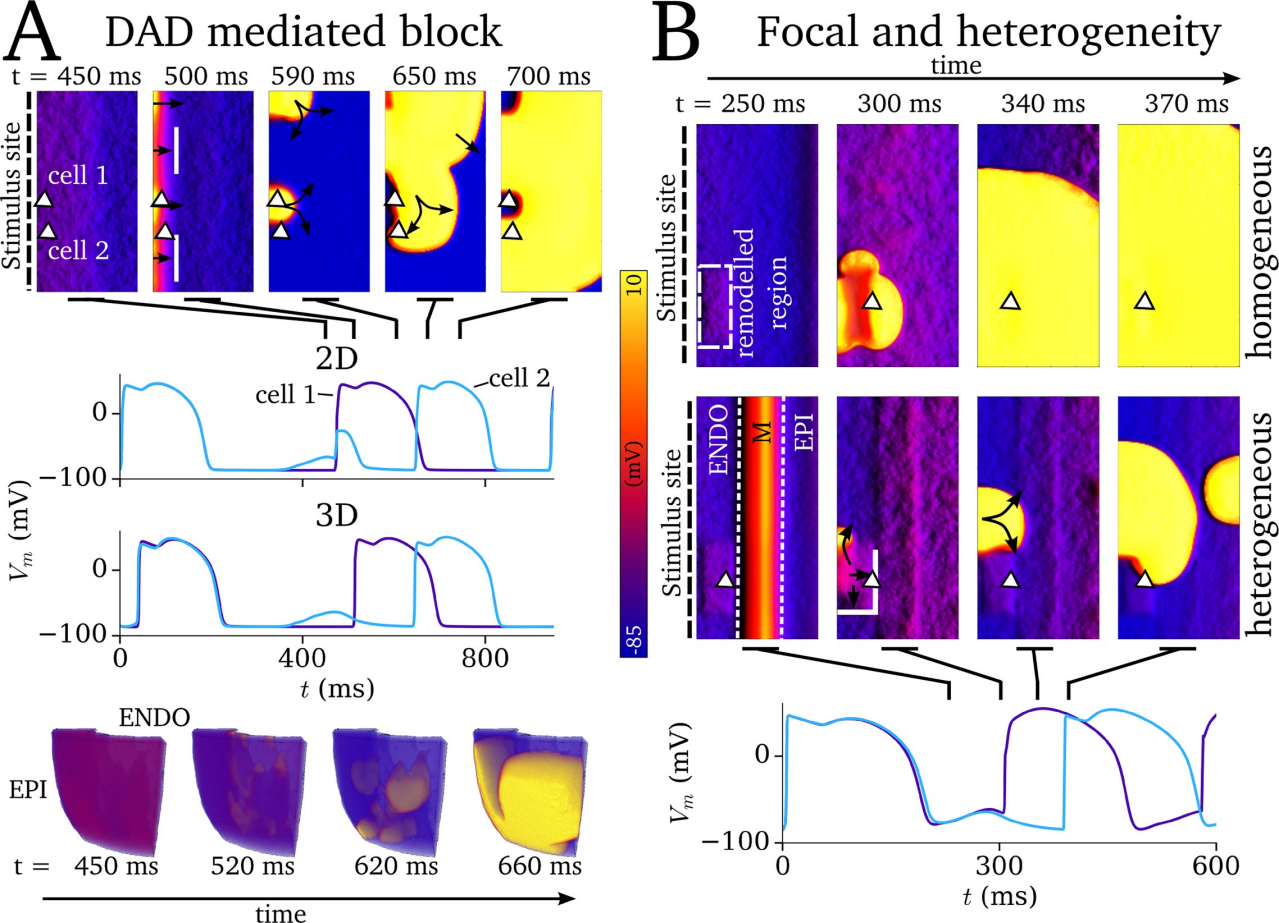
Mechanisms of SCRE mediated conduction block. A–Demonstration of DAD-mediated conduction block in 2D (upper panels) and 3D (lower panels). In both cases, two stimuli were applied to one side of the tissue (left edge of the 2D sheet; ENDO surface in 3D) at a coupling interval of 500 ms, with SCRE induced DADs interrupting the second applied stimulus. Spatial snapshots cover the time just before and during this second stimulus. The locations of the cells from which the AP traces are taken are indicated by the triangular markers in the 2D sheets; for the 3D case, the traces correspond to a region which did (blue) and did not (purple) exhibit conduction block. Solid white lines represent sites of conduction block. B–Demonstration of spontaneous focal excitation leading to different behaviour in electrically homogeneous or heterogeneous tissue. The purple trace corresponds to the homogeneous condition, in which the focal excitation propagates uniformly; the blue trace corresponds to the heterogeneous condition in which focal excitation propagates non-uniformly following conduction block. The triangular marker indicates the site from which the AP traces were extracted, and the region of reduced *I*_K1_ is highlighted by the dashed-white rectangle. The stimulus is applied once to the left edge (ENDO region) of the tissue at t = 0 ms; the second excitation is spontaneously induced.

SCRE leading to non-TA inducing DADs was demonstrated to produce unidirectional conduction block during applied pacing in a homogeneous 2D model of the human ventricular wall (Fig 10A; S4 and S5 Videos) under simulated sodium channelopathy conditions (see Methods: Simulation protocols), wherein the SCRE induced DADs inactivate the sodium channel and result in non-uniform propagation following a stimulus uniformly applied to one edge (Fig 10A).

The potential for SCRE mediated focal excitation to result in unidirectional conduction block due to regional APD heterogeneity was demonstrated in the 2D transmural model of the human ventricular wall. Focal excitations originating in a small temporal window could exhibit a conduction block with the still refractory M-cell region; this was not observed in the homogeneous tissue (Fig 10B; S6 Video). Note that the site of conduction block, unlike in simulations with an applied focal stimulus, is not necessarily clearly at the boundary between the EPI and M cells: in the illustrated case, the focus emerges from the far side of the boundary.

### Re-entry promotes spontaneous Ca^2+^ release and focal excitation

**Fig 11:**
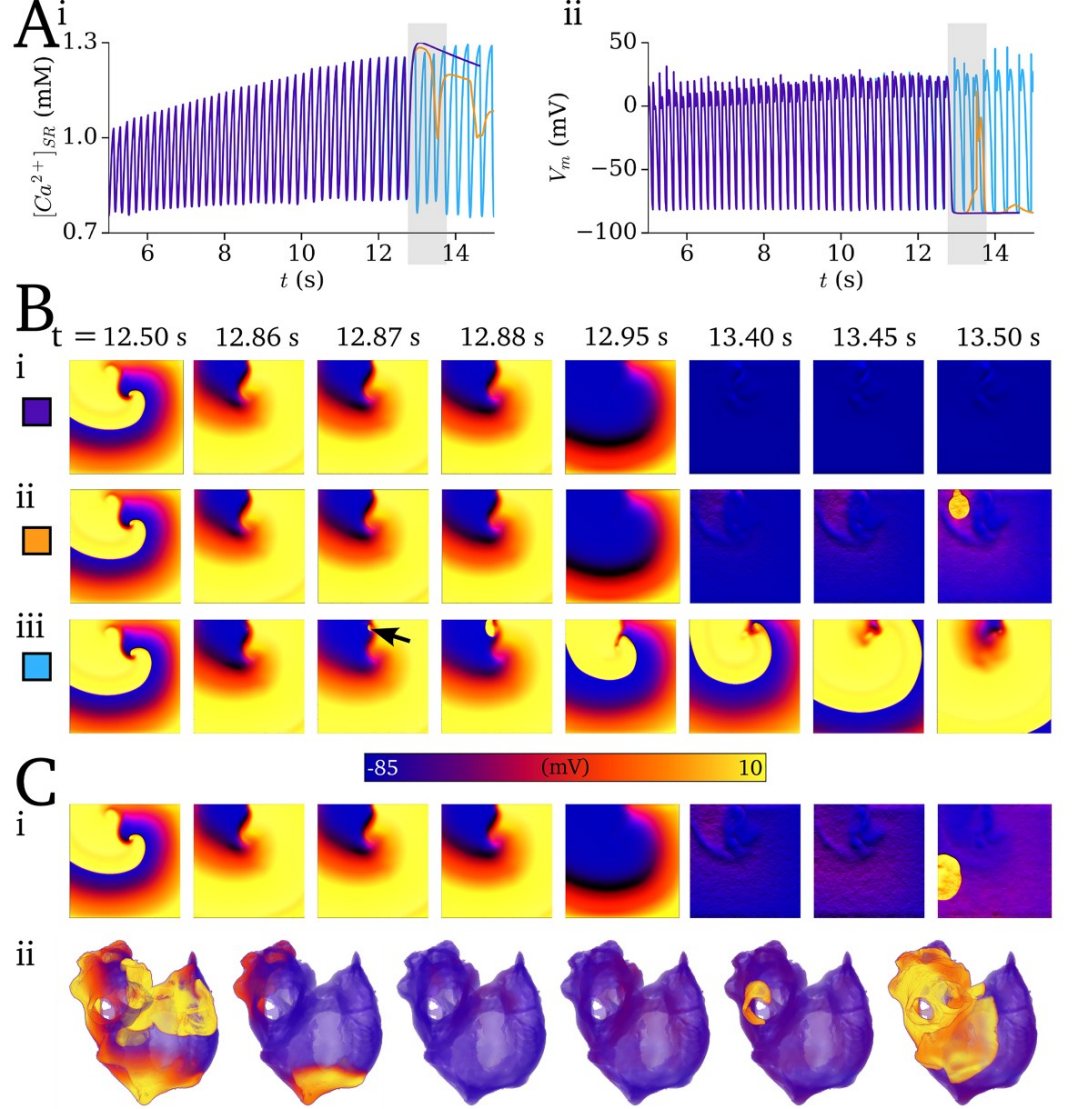
Coupling between re-entry and SCRE. A–SR-Ca^2+^ concentration (i) and *V*_m_ (ii) associated with sustained re-entry followed by self-termination (at around 13 s), simulations without SCRE (purple) and with the General Dynamic SRF model with two different thresholds (orange, 1.125 mM; blue, 1.0 mM). B–Temporal snapshots of voltage in the 2D sheet associated with the traces shown in A, showing self-termination (i) and the emergence of delayed (ii, corresponding to the orange traces in A) and rapid (iii, corresponding to the blue trace in A) focal excitations. C–Examples of non-localised focal excitations emerging in the 2D sheet (i) and 3D whole atria models (ii). Baseline General Dynamic SRF parameters, corresponding to panel A(orange)/Bii: *CaSR*_threshold_ = 1.25 mM; *CaSR*_max_ = 1.525 mM; *CaSR*_P_range_ = 0.05 mM; *t*_i,Sep_^min^ = 300 ms; *t*_i,Sep_^max^ = 870 ms; *t*_i,width_^min^ = 200 ms; *t*_i,width_^max^ = 1000 ms; *MD*^min^ = 150ms; *MD*^max^ = 600 ms; *λ*_width_^min^ = 70 ms; *λ*_width_^max^ = 300 ms; *H*_width_ = 2.5. Parameter differences for panel A(blue)/Biii: *CaSR*_threshold_ = 1.00 mM; *CaSR*_max_ = 1.2 mM; *t*_i,Sep_^min^ = 30 ms; *MD*^min^ = 50ms; *λ*_width_^min^ = 20 ms. For panel Ci: *MD*^min^ = 160ms; *λ*_width_^min^ = 75 ms; *λ*_width_^max^ = 300 ms. For panel Cii: *CaSR*_threshold_ = 0.9 mM; *CaSR*_max_ = 1.3 mM; *t*_i,Sep_^min^ = 30 ms; *t*_i,width_^min^ = 20 ms; *MD*^min^ = 80ms; *MD*^max^ = 800 ms; *λ*_width_^min^ = 20 ms.

Sustained re-entry combined with pro-arrhythmic conditions resulted in significant SR-Ca^2+^ loading, which promoted the onset of SCRE mediated focal excitations following termination. In the illustrated 2D simulations using the minimal RA AP model, re-entry sustained for ~13 s before self-terminating, loading the SR-Ca^2+^ to ~ 1.25 mM (Fig 11A and 11B; S7–S10 Videos). By implementing a General Dynamic SRF model with the SR-Ca^2+^ threshold set below, but close to, the maximal SR-Ca^2+^ observed (*CaSR*_threshold_ = 1.125 mM), a single, delayed focal excitation was observed following termination (Fig 11A and 11Bii). With a lower threshold (*CaSR*_threshold_ = 1.0 mM) multiple and rapid focal excitations were observed which perpetuated the arrhythmic dynamics for the duration of the simulation (Fig 11A and 11Biii). In general, where the tissue was sufficiently vulnerable for focal excitation to emerge, its origin was localised to the region of the scroll wave core on its final excitation pathway (Fig 11B; S8 and S9 Videos); focal excitations emerging away from the scroll wave core were also observed closer to the threshold (Fig 11C).

**Fig 12:**
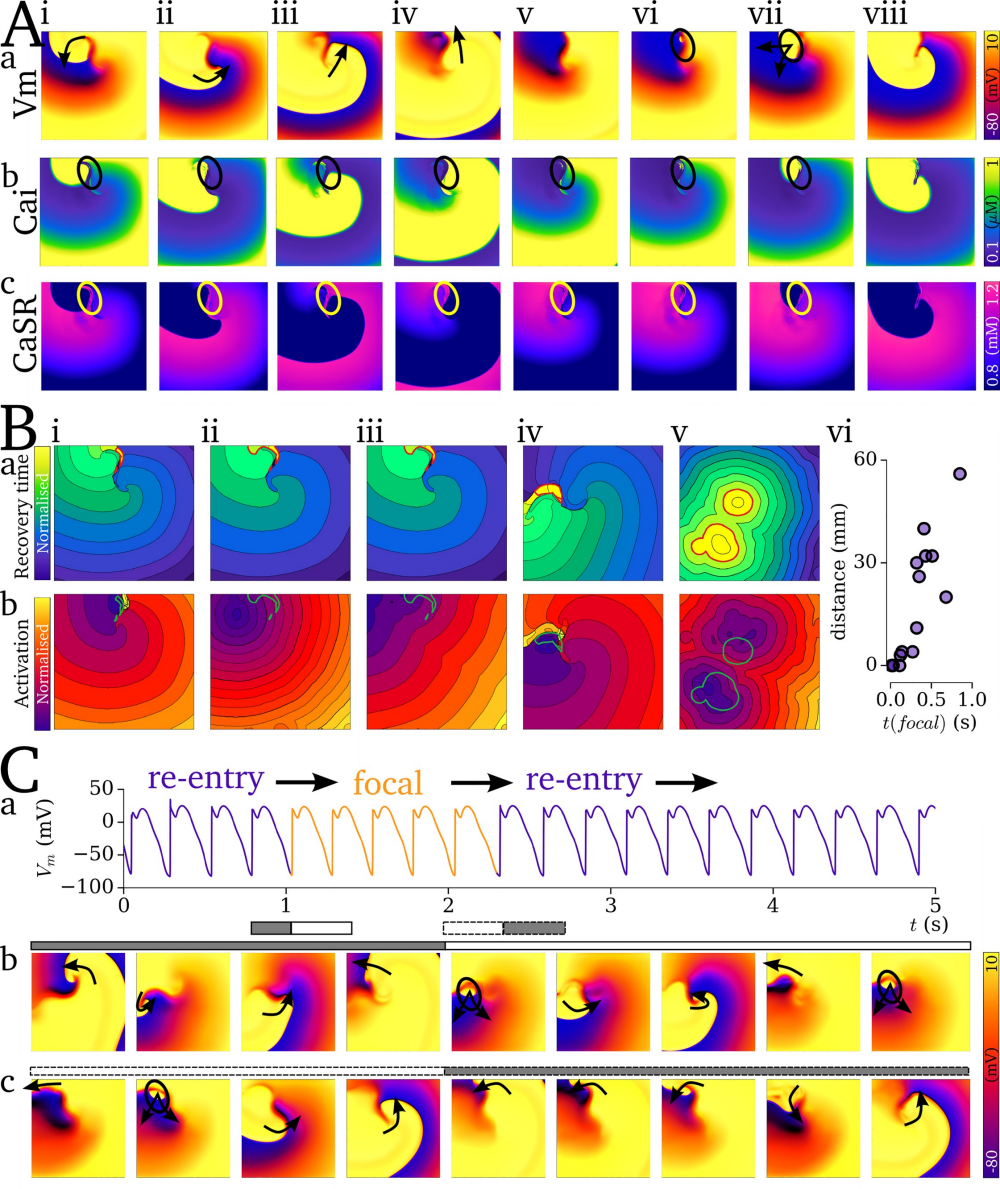
Functional localisation of re-entry and focal activity. A–Temporal snapshots (i-viii) of voltage (a), intracellular Ca^2+^ (b), and SR-Ca^2+^ (c) associated with the final, self-terminating re-entrant cycle and first focal excitation. Arrows in (a) indicate the conduction of the re-entrant and then focal excitations. Highlighted region (circle) illustrates the island of large SR-Ca^2+^ associated with the unexcited scroll wave core (i-iv) and its correlation with the focus of ectopic activation (vi-vii). B–Examples of recovery time maps (a) and focal activation maps (b) for 5 independent simulations (i-v; selected from simulations covering the full range of coupled AP models and tissue parameters which led to transient re-entry which sustained sufficiently to load the SR-Ca^2+^) associated with the self-termination of re-entry followed by ectopic excitation. The contour surrounding the region of longest recovery time (corresponding to the unexcited core illustrated in A) is highlighted in red in the recovery time map and green in the activation maps. (vi)—summary of the correlation between distance between from centre of the focal source to the closest edge of the region of longest recovery and the time of the focal excitation, t(focal), relative to the latest activation of the non-focal excitation. C–Mechanism switching between re-entrant and focal excitation, showing the AP from a randomly selected cell (a) and temporal snapshots associated with the transition from re-entry to focal activity (b) and focal activity to re-entry (c). Snapshots corresponds to the temporal range illustrated by the grey and white bars with solid (re-entry to focal) and dashed (focal to re-entry) borders. Parameters which led to the mechanism switching simulation: *CaSR*_threshold_ = 1.0 mM; *CaSR*_max_ = 1.2 mM; *CaSR*_P_range_ = 0.05 mM; *t*_i,Sep_^min^ = 30 ms; *t*_i,Sep_^max^ = 870 ms; *t*_i,width_^min^ = 20 ms; *t*_i,width_^max^ = 200 ms; *MD*^min^ = 50ms; *MD*^max^ = 800 ms; *λ*_width_^min^ = 20 ms; *λ*_width_^max^ = 300 ms; *H*_width_ = 2.5.

Localisation of focal excitation to the scroll wave core is determined by the dynamics of re-entrant excitation: the scroll wave core remains unexcited associated with each re-entrant cycle (Fig 12A), which leads to an island of high SR-Ca^2+^ in this region (Fig 12Ac). Due to this large SR-Ca^2+^ and longest recovery time, this region thus presents the earliest SCRE which may manifest as TA. This relationship is clearly illustrated by comparing the spatial distribution of recovery time (time since last AP which induced CICR) at the moment before focal excitation occurs with the associated focal activation map: the site of activation can correspond exactly (Fig 12Bi, iv) or approximately (Fig 12Bii-iii, v) to the location of longest recovery time, in both single- (Fig 12Bi-iv) and double- scroll wave (Fig 12Bv) simulations; the earlier the focal excitation occurs relative to the final re-entrant scroll wave, the stronger the correlation between location of longest recovery time and activation source (Fig 12Bvi).

Under pro-SCRE conditions comprising tight synchronisation (small width of *t*_i_ distribution at short-coupled intervals) and short-duration, large-amplitude releases, focal activity can occur at a rate comparable to re-entry; such pacing can transiently drive the excitation, potentially eventually terminating arrhythmia as the SR-Ca^2+^ depletes. Furthermore, the wavefront could degenerate back into a sustained re-entrant excitation (Fig 12C; S11 Video). The latter is promoted by the asymmetric conduction patterns emerging from rapid focal excitation interacting with the tail of the previous scroll wave or asymmetric focal excitation. During this mechanism switching, the underlying driving mechanism (focal or re-entrant) was not clear in AP traces from randomly selected cells in the 2D tissue, and could only be determined by spatio-temporally high-resolution analysis of the excitation patterns (Fig 12C).

**Fig 13:**
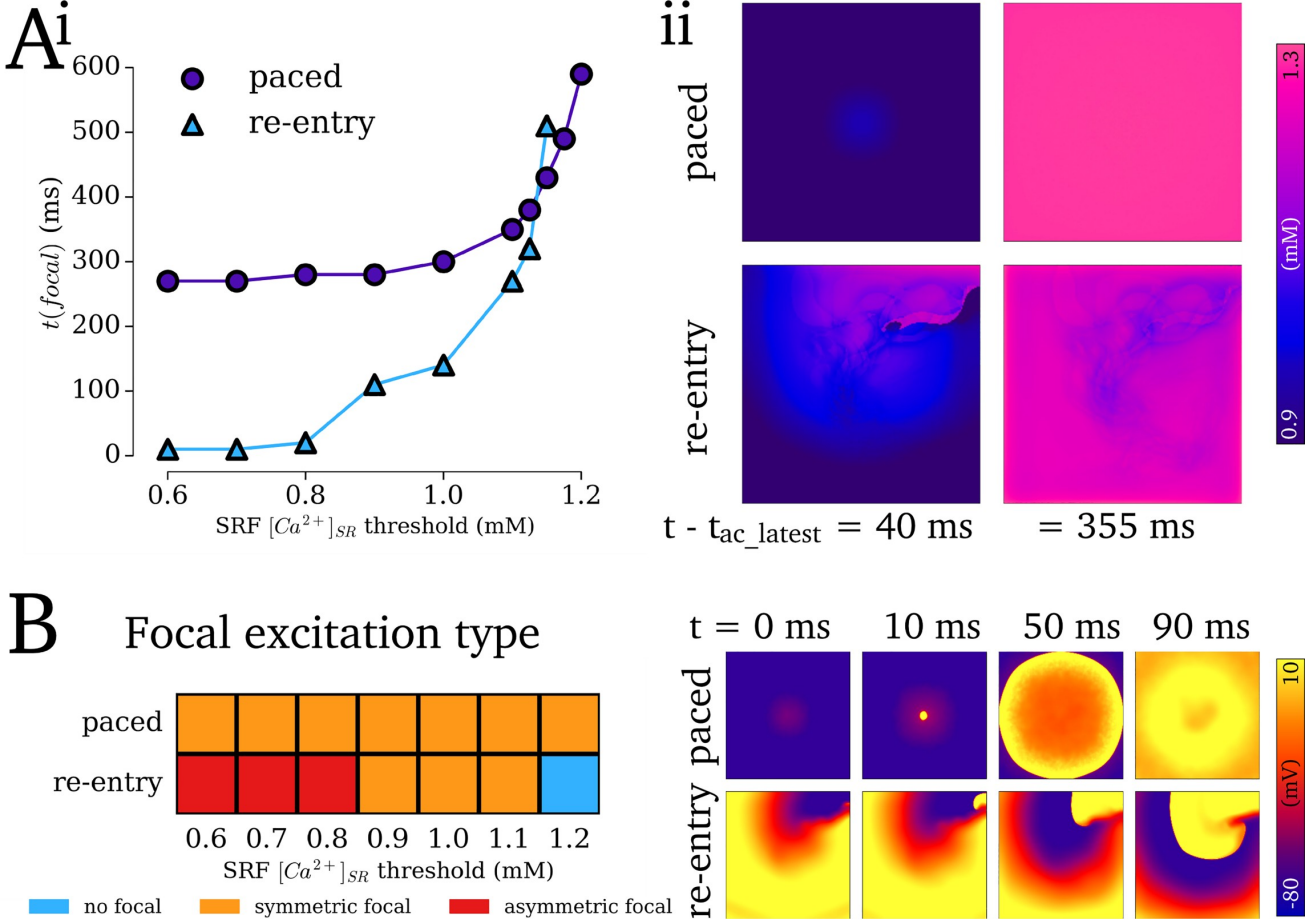
Comparison of focal excitations following re-entry vs regular pacing. Ai–Focal excitation time, t(focal), at different SR-Ca^2+^ thresholds for release under re-entry (purple, circle markers) and matched regular pacing (blue, triangular markers) conditions. t(focal) is calculated relative to the latest activation time of the final paced or re-entrant excitation. (ii)–Temporal snapshots of spatial SR-Ca^2+^ concentration in the 2D sheets, comparing the heterogeneity at equivalent time points. Bi–Categorisation of the outcomes of focal excitation under the two conditions into either non-focal (blue), symmetric focal (orange) or asymmetric focal (red). (ii)–Examples of symmetrical vs asymmetrical conduction patterns emerging following each type of excitation. The times labelled in Bii are relative to the onset of focal excitation.

SR-Ca^2+^ loading as a result of re-entry led to different emergent behaviour than that induced by regular rapid pacing with matched average SR-Ca^2+^ peak concentrations (Fig 13). The impact of the islands of high SR-Ca^2+^ with longer waiting times is clear, as focal excitations in re-entry simulations could emerge almost immediately following termination whereas those following regular rapid pacing exhibited a minimum delay (approximately equal to the sum of SR refilling time and earliest *t*_i_; Fig 13Ai). However, when SR-Ca^2+^ is close to the threshold for release, focal excitations were observed following rapid pacing but not following re-entry. This was due to the heterogeneous SR-Ca^2+^ loading (and subsequent timing of SCRE) associated with re-entry, whereas regular rapid pacing led to almost homogeneous SR-Ca^2+^ loading (offset by activation time; Fig 13Aii). Focal excitations following regular rapid pacing were always plane wave, not occurring early enough (in electrically homogeneous media) to interact with the tail of the previous excitation; those following re-entry, however, exhibited asymmetric excitation patterns when occurring sufficiently early to interact with the tail of the previous excitation (Fig 13B). Note that this is due to both the earlier excitation time and the asymmetry of the previous re-entrant excitation compared to the plane wave of regular pacing.

## Discussion

### Summary

An increased incidence of spontaneous calcium release events (SCRE) is frequently observed in isolated cardiomyocytes from the diseased myocardium [11–15], and their pro-arrhythmic coupling to the membrane potential through activating inward NCX current has led to the hypothesis that these dysfunctional Ca^2+^ handling phenomena play a role in the initiation and dynamics of complex arrhythmia conduction patterns. However, investigating these multi-scale mechanisms presents a significant challenge, both for experimental and simulation approaches, and thus the precise mechanisms and potential importance of these events manifesting as tissue-scale arrhythmia have yet to be fully described.

In this study, a multi-scale computational approach was developed to simulate the dynamics of stochastic SCRE in organ-scale models of cardiac excitation. The computational framework comprises a hierarchy of models (Fig 1) encompassing the microscopic- (3D cell model), mesoscopic- (0D cell model) and macroscopic-scales (tissue models). Spontaneous Release Functions (SRF; Fig 3) were used to reproduce the morphology of SCRE in the 0D cell and tissue models (Figs 6 and 7), directly translating the single-cell modelling to the tissue-scale.

The approaches were then applied to study arrhythmia mechanisms. Firstly, the dual role of electrotonic coupling in the emergence of SCRE as a full tissue focal excitation was illustrated (Fig 8). Secondly, the role of cellular variability in SCRE dynamics on the SR-Ca2+—focal excitation relationship was investigated (Fig 9). Thirdly, two mechanisms of SCRE mediated conduction block were demonstrated (Fig 10), illustrating mechanisms by which re-entry may be initiated. Finally, the long-term interactions with re-entry were investigated (Figs 11 and 12), demonstrating that sustained re-entrant excitation can load the SR-Ca^2+^ and promote SCRE mediated focal excitation, and revealing a purely functional mechanism of localisation. This mechanism also resulted in re-entrant pre-pacing leading to earlier and more asymmetric focal excitations than observed in matched regular pacing scenarios (Fig 13).

### The spontaneous release functions

The present study presents a novel approach to efficiently simulate stochastic sub-cellular Ca^2+^ release dynamics through the implementation of simple and controllable analytical wave functions—the SRF. The use of these simple functions allowed direct control over SCRE dynamics as well as models fit to the behaviour of 3D single cells, facilitating both general mechanistic analysis and translation of single-cell investigations to the organ-scale.

The phenomenological approach considered whole-cell behaviour only, and was not based on capturing underlying detail. Sub-cellular heterogeneity of factors such as t-tubule density can influence the vulnerability to and dynamics of whole-cell SCRE [36,41]. This may be particularly relevant for atrial cells (which, for large mammals, exhibit variable size and t-tubule density, and for small mammals in general exhibit a lack of t-tubules [42,43]) and disease conditions (in which t-tubule density is often observed to decrease [44–46]). This top-down approach facilitates direct incorporation of the dynamics of these heterogeneous conditions, which may be essential for accurately modelling focal excitation in the atria and disease states. In general, adaptations to the SR-Ca^2+^ threshold for SCRE, frequently observed to shift towards lower concentrations in pathophysiological conditions such as heart failure [47,48] (possibly as a result of sub-cellular structural remodelling [36]), can be easily accounted for using this approach.

### Conditions for the emergence of SCRE mediated focal activity

Analysis of the impact of cellular variability in the dynamics of SCRE on the probability of the emergence of an ectopic beat highlighted the complex considerations surrounding the amplitude and timing of SCRE, and its modulation by *I*_K1_: introducing a proportion of cells more vulnerable to SCRE in combination with reduced *I*_K1_ led to a negative shift the SR-Ca^2+^ relationship (i.e., towards lower SR-Ca^2+^ values) as well as the emergence of lower probability events, contrasting to the almost step-function response in homogeneous tissue; the same SCRE variability without reduced *I*_K1_ right-shifted the probability curve, despite the presence of larger amplitude SCRE compared to the homogeneous condition. This important role of reduced *I*_K1_, as previously highlighted [19,20,25,49], is due to a combination of it resulting in (i) resting potentials closer to the *I*_Na_ activation threshold and (ii) smaller repolarising current opposing the DAD, and may be of particular relevance to arrhythmia associated with heart failure remodelling, in which *I*_K1_ is generally reduced.

Preliminary analysis was also performed to assess the criticality of parameter distributions for the emergence of focal excitation (S2 Text (Validation and Results)–section 3, Figure S4). These simulations revealed clear constraints determining thresholds between distributions which did and did not result in focal activity, and the modulation of these constraints by *I*_K1_; however, they also revealed the challenges of predicting emergent behaviour within the threshold region.

### Pro-arrhythmic feedback between re-entry and SCRE

The mechanisms by which a suitably timed focal excitation can result in conduction block and the onset of self-perpetuating re-entrant excitation have been extensively studied previously (e.g. [27]). The present study also demonstrates a novel feedback mechanism by which re-entrant excitation promotes SCRE: Combining simulations of SCRE with those of sustained re-entrant excitation demonstrated that the resulting SR-Ca^2+^ loading can promote the emergence of focal excitation following termination of re-entry, perpetuating the arrhythmic conduction patterns.

The simulations revealed a purely functional mechanism of localised ectopic and re-entrant excitation, without the requirement for a specifically vulnerable region, determined by the additional relaxation time associated with the unexcited core of a scroll wave. This localization combined with rapid focal activity resulted in excitation patterns which were highly asymmetric and almost indistinguishable from the re-entry, due to conduction block with the tail of the previous re-entrant or focal excitation. Furthermore, these asymmetric conduction patterns could result in a complete re-entrant circuit and the re-initiation of sustained re-entry. This mechanism switching may not clearly present in mapping or ECG measurements, but may have significant implications for pharmacological intervention and provide one explanation for variable and limited success.

### Generalisation of the approaches

In the first instance, the Dynamic Fit SRF models are naturally generalised within the range of modifications to the AP model which do not include any differences in the underlying Ca^2+^ handling system, i.e., remodelling and regulation of the sodium and potassium currents and *I*_CaL_: these currents do not directly affect the probability of spontaneous Ca^2+^ sparks or their propagation as whole-cell events in the model, and so the influence of their modulation on SCRE is only through their effect on SR-Ca^2+^ loading and electrotonic load. This can be further generalised if small modifications were made to the Ca^2+^ handling system in relation to *J*_up_, *J*_leak_ and to a lesser extent *I*_NCX_, which have only a small effect on Ca^2+^ spark propagation (in the model) and do not significantly alter the SR-dependence. This was demonstrated in the present study by the ISO model (which had no effect on RyR or NCX, but did affect *J*_up_ and LTCC open channel availability): the SRF did not require an ISO-dependent parameter in order for the 0D model to reproduce the 3D model behaviour under ISO conditions (Fig 6).

Further modifications to the Ca^2+^ handling system, which do significantly alter the SR-dependence, required the SRF models to be rederived, as was demonstrated by the two remodelling models. Whereas time-consuming, such an approach could be used to reproduce different dynamics emerging from any individual or combination of regulation and/or remodelling conditions.

The process and approaches presented in this study can be further generalised to be incorporated into other cell models. Theoretically, any “standard” non-spatial cell model which contains a rigorous model of CICR and includes RyR open state dynamics can directly include the analytical SRF. As an example, the Grandi et al., 2011 human atrial cell model [50] was selected as this includes one of the most physiological descriptions of RyR dynamics in a non-spatial cell model. Incorporation of the SRF required only a few additional lines of code (pertaining to the implementation algorithm) further to the SRF themselves, with the magnitude rescaled to reflect the maximum open-state occupancy observed in that model (S3 Text (Generalisation of approaches)). This demonstrates the potential suitability for direct integration with available contemporary, non-spatial AP models, without the requirement to replace the native intracellular Ca^2+^ handling system or develop a spatial model equivalent.

The General Dynamic approach also facilitates parameterisation to experimental data, even with limited information. To demonstrate this functionality, the data provided by Workman et al., 2012 [34] were selected (S3 Text (Generalisation of approaches)), as these data pertain only to measurement of membrane potential (and not direct measurement of Ca^2+^ dynamics) and are therefore *not* an ideal dataset for parameterisation of SCRE. Even under these limited conditions, it was possible to broadly reproduce the emergence of DADs and TA observed experimentally (S3 Text (Generalisation of approaches)).

### Comparison to other methods for tissue-scale simulation of SCRE

Work from only a few research groups has attempted to simulate stochastic SCRE at the organ-scale [19–25]. These independent studies used alternative phenomenological approaches to overcome the inherent challenges of this multi-scale simulation as presented in this manuscript.

Further to providing an independent approach which forms a complementary tool to the previous models, which is of particular importance for theoretical investigation of systems with many unknowns and highly non-linear behaviour, the present approach differs from those previously namely in: (i) the motivation to reproduce SCRE in tissue models in-line with that observed in specific (and variable) 3D cell models, for direct translation of single cell modelling studies to the tissue-scale; (ii) the ability to directly control waveform parameters and relate observed behaviour to these parameters; (iii) an approach which readily allows direct incorporation of both limited and detailed experimental data; and (iv) the presentation of an open-source computational framework for congruent investigation of SCRE at single cell- and tissue-scales (S1 Code).

### Comparison to previous mechanistic studies

Only a few computational studies have attempted to dissect the multi-scale mechanisms involved in SCRE mediated arrhythmia. Initially, the minimum tissue substrate for the emergence of focal excitations resulting from non-stochastic EADs and DADs was investigated [18], followed by demonstration of independent cellular events emerging as a focal excitation [19–25] and SCRE as a mechanism for both triggered activity and conduction block [25]. Potential interaction with extracellular matrix remodelling was demonstrated [24], as well as the potential non-linear considerations for pharmacological action on both triggers and substrate [22].

Important features observed in these previous studies are in agreement with that of the present study, i.e. in relation to the mechanism of synchronisation overcoming electrotonic load, the steep SR-Ca^2+^ relationship of ectopic activity in homogeneous tissue, the importance of *I*_K1_ in governing the vulnerability to ectopic activity, and the potential role of non-TA inducing DADs to cause conduction abnormalities [19,20,25,49]; this study therefore provides independent validation of these features. The present study also provides novel analyses and mechanistic insight, pertaining to: (i) the analysis of SCRE vulnerability variability on the SR-Ca^2+^-TA relationship, and the quantification of parameter distribution thresholds to predict focal excitation; (ii) investigation of the potentially pro-arrhythmic bi-directional coupling between SCRE and re-entry; and (iii) demonstration of a mechanism of localisation of these phenomena which can also lead to focal-re-entrant mechanism switching.

### Limitations

Due to the substantial components of this paper, full limitations associated with the models and approaches are discussed in detail in the S4 Text (Limitations) and the reader is referred there for full description of the applicability of the models in present form; here, key model limitations and those regarding the novel analysis are discussed.

The method to derive the SRF in order to reproduce behaviour of the single cell model (i.e., not the general implementations) required a large volume of computationally intensive simulations to be performed (~ 5 000 hours of computation time per condition), although these do not need to be repeated once the parameters have been derived. Alternative approaches were also considered which have potential advantages. For example, deriving a similar iterative-map approach to that presented in [51], parameterized to the spatial cell model dynamics, would require significantly less intensive simulations and perhaps provide a more robust underlying dynamic system. A purely mathematical derivation would completely circumvent this computationally intensive requirement, and was considered in the early stages of this research. Such a derivation remains an attractive prospect for a truly rigorous and portable efficient model of SCRE. However, the analytical waveform approach presented also has advantages: based on whole-cell behaviour, parameter sets could be derived to describe underlying spatial models in limitless different conditions including sub-cellular heterogeneity and its variability, which may be significantly more challenging to reproduce with an underlying dynamical system. The General Dynamic implementation also negates the requirement for simulations on which to derive the model, and permits both comprehensive mechanistic analysis and direct parameterization to experimental data.

Simulations of SCRE emerging as tissue-scale arrhythmia in general required model parameters and conditions towards the extreme of physiologically observed behaviour (i.e., corresponding to highly diseased myocardium), and therefore do not provide a complete picture of the role of SCRE across the spectrum of patients presenting arrhythmia. Further analysis and new methodological approaches will be required to practically simulate lower probability events and fully assess the role of SCRE in cardiac arrhythmia.

In the wider context, these simulations represent only initial detailed *in silico* analysis of the impact of SCRE at the organ scale: experimental validation, integration with biophysically detailed species and disease specific models, and parameterisation to experimentally measured SCRE statistics, are essential to translate the approaches and mechanistic insight of the present study to arrhythmia in patients.

### Application of the framework

It is intended that these approaches will be further developed and incorporated with sophisticated biophysically detailed cell models and experimentally validated simulation of cellular SCRE in multiple cardiac conditions, in order to suggest new experiments and contribute to detailed analysis of the role of SCRE in cardiac arrhythmia. Demonstration of the generalisation potential of the approaches is hoped to encourage those interested researchers in the community to integrate the presented framework with their cell models and simulation studies; open-source C++ code of the entire framework and detailed documentation is therefore provided in S1 Code and available on the GitHub repository (https://github.com/michaelcolman/MSCSF).

### Conclusions

The multi-scale cardiac modelling approaches described in this manuscript and accompanying model code present the possibility to model the impact of stochastic, sub-cellular calcium dynamics on organ scale arrhythmic excitation patterns with congruent detailed cellular and tissue simulations or parameterised to specific experimental datasets. Such approaches revealed multi-scale coupling between SCRE and re-entrant excitation and a purely functional mechanism for their localisation. The mechanistic insight gained from the application of these approaches may help to improve understanding and management of cardiac arrhythmia.

## Supporting information

S1 Text Model description. Document containing full model equations and parameters for all of the cell and tissue models and SRF presented in this study.
(PDF)

S2 Text Validation and Results. Document containing additional figures and text for the validation of the methods and additional results.
(PDF)

S3 Text Generalisation of approaches. Document containing additional figures and text for the generalisation of approaches.
(PDF)

S4 Text Limitations. Document containing detailed description of the limitations associated with this study.
(PDF)

S1 Code C/C++ code and documentation containing all implementations presented in the study. (TGZ)

S1 Video Illustration of Ca^2+^ clamp and SCRE dynamics. Video corresponds to snapshots shown in Fig 2. Left panels show proportion of open RyR (upper) and the intracellular (purple/left axis) and SR (blue/right axis) Ca^2+^ concentrations (lower). Right panel shows the intracellular Ca^2+^ concentration in the 3D volume of the idealised cell model. Video covers SR-Ca^2+^ below threshold, just above, and significantly above.
(MP4)

S2 Video Emergence of focal excitation in 2D tissue. Intracellular Ca^2+^ concentration (left) and membrane potential (right) illustrating the emergence of a focal excitation. Corresponds to the condition shown in Fig 8.
(MP4)

S3 Video Emergence of focal excitation in 3D tissue. Intracellular Ca^2+^ concentration (left) and membrane potential (right) illustrating the emergence of a focal excitation in the 3D atrial model.
(MP4)

S4 Video DAD mediated conduction block in 2D. 2D homogeneous sheet, showing the emergence of DADs following regular pacing before an S2 stimulus is applied to the upper edge, resulting in conduction block. Corresponds to Fig 10A—upper.
(MP4)

S5 Video DAD mediated conduction block in 3D. 3D homogeneous ventricular wedge, showing the final S1 excitation and an S2 excitation leading to conduction block; both stimuli applied to the ENDO wall. Corresponds to Fig 10A–lower.
(MP4)

S6 Video Focal mediated conduction block. Simulation in homogeneous (upper) and heterogeneous (lower) 2D sheets, with focal excitation emerging at a similar time, leading to conduction block only in the heterogeneous case. Corresponds to Fig 10B.
(MP4)

S7 Video Focal excitation following re-entry pt 1. Left panels show voltage (upper) and SR-Ca^2+^ (lower) from a randomly selected cell in 2D tissue, right panels show the spatial distribution of voltage (left), intracellular Ca^2+^ (middle) and SR-Ca^2+^ (right). Data show the no SRF case (purple lines; upper 2D panels) and with SRF included (red lines, lower 2D panels), set with the threshold being lower than but close to the SR-Ca^2+^ peak reached during re-entry. This simulation illustrates a single, not-functionally localised focal excitation, corresponding to Fig 11Ci.
(MP4)

S8 Video Focal excitation following re-entry pt 2. Left panels show voltage (upper) and SR-Ca^2+^ (lower) from a randomly selected cell in 2D tissue, right panels show the spatial distribution of voltage (left), intracellular Ca^2+^ (middle) and SR-Ca^2+^ (right). Data show the no SRF case (purple lines; upper 2D panels), the SRF case from S7 Video (middle 2D panels), and an SRF case with the threshold further below the SR-Ca^2+^ than S7 Video (red lines, lower 2D panels). This new simulation illustrates a single, functionally localised focal excitation, corresponding to Fig 11Bii.
(MP4)

S9 Video Focal excitation following re-entry pt 3. Left panels show voltage (upper) and SR-Ca^2+^ (lower) from a randomly selected cell in 2D tissue, right panels show the spatial distribution of voltage (left), intracellular Ca^2+^ (middle) and SR-Ca^2+^ (right). Data show the no SRF case (purple lines; upper 2D panels), the SRF cases from S7 and S8 Videos (middle 2D panels), and an SRF case with the threshold set further below the SR-Ca^2+^ than S7 and S8 Videos (red lines, lower 2D panels). This new simulation illustrates multiple, functionally localised focal excitations, corresponding to Fig 11Biii.
(MP4)

S10 Video Focal excitation following re-entry in 3D. A simulation in the 3D human atrial model in which re-entry self terminates and focal excitations emerge. Left panel is voltage, middle panel is intracellular Ca^2+^ and right panel is SR-Ca^2+^_._ Simulation corresponds to that shown in Fig 11Cii.
(MP4)

S11 Video Mechanism switching. AP traces (upper panels) and snapshots of voltage and intracellular and SR Ca^2+^ (lower panels). A number of re-entrant cycles are shown, followed by rapid focal activity which degenerates back into re-entry. The driving mechanism is indicated by the colour of the AP traces–purple for re-entry and orange for focal.
(MP4)
