# Supplementary material for: Arrhythmia mechanisms and spontaneous calcium release: Bi-directional coupling between re-entrant and focal excitation
Source: PLoS Comput Biol. 2019 Aug 8;15(8):e1007260. doi: 10.1371/journal.pcbi.1007260 (PMC6687119; doi:10.1371/journal.pcbi.1007260)
Supplement: S1 Text — Document containing full model equations and parameters for all of the cell and tissue models and SRF presented in this study. (PDF) [file pcbi.1007260.s001.pdf]

Supplementary Material S1 Text: Model Description

Arrhythmia Mechanisms and Spontaneous Calcium Release: Bi-directional Coupling Between Re-entrant and Focal Excitation

Michael A. Colman

School of Biomedical Sciences, University of Leeds, Leeds, UK

Correspondence:

m.a.colman@leeds.ac.uk

Contents

1. Spatial Ca^{2+} cycling model 3
   Variables 3
   Fundamental equations for Ca^{2+} concentration 4
   Reaction terms 5
      Intracellular Ca^{2+} release, J_{rel} 5
      L-type Ca^{2+} channel flux, J_{CaL} 7
      Intracellular uptake and leak, J_{up} and J_{leak} 9
      Membrane fluxes, J_{NaCa}, J_{pCa} and J_{CaB} 10
   Ca^{2+} Buffering 11
      Instantaneous buffering in the cytoplasm 11
      Instantaneous buffering in the jSR 11
      Troponin buffering and force 11
2. The 0D, deterministic model 13
3. Hybrid-minimal AP model 13
4. Non-specific remodelling and ISO models 14
5. Tissue models 15
6. Spontaneous release functions 16
   RyR waveforms 16
   Parameter distributions 16
   The Direct Control SRF model 18
   The Dynamic Fit SRF model 18
   The General Dynamic SRF model 20
   Inverse functions 21
   Implementation with the 0D model 22
References 23

1. Spatial Ca^{2+} cycling model

This section describes the spatial Ca^{2+} cycling model, a simplified and efficient version of a previously developed model [1].

Variables

Table S1: Ca^{2+} handling model variables

Parameter	Description	Unit
$[Ca^{2+}]_{ds}$	Average Ca^{2+} concentration in the dyadic cleft	μM
$^{m}[Ca^{2+}]_{ds}$	Ca^{2+} concentration in dyad in CRU *m*	μM
$[Ca^{2+}]_{rbSS}$	Average Ca^{2+} concentration in the subspace	μM
$^{m}[Ca^{2+}]_{rbSS}$	Ca^{2+} concentration in subspace in CRU *m*	μM
$[Ca^{2+}]_{cyto}$	Average Ca^{2+} concentration in the bulk cytoplasm	μM
$^{m}[Ca^{2+}]_{cyto}$	Ca^{2+} concentration in cytoplasm in CRU *m*	μM
$[Ca^{2+}]_{nSR}$	Average Ca^{2+} concentration in the network SR	μM
$^{m}[Ca^{2+}]_{nSR}$	Ca^{2+} concentration in network SR in CRU *m*	μM
$[Ca^{2+}]_{jSR}$	Average Ca^{2+} concentration in the junctional SR	μM
$^{m}[Ca^{2+}]_{jSR}$	Ca^{2+} concentration in junctional SR in CRU *m*	μM
$^{m}J_{rel}$ | J_{rel}	Release flux in dyad *m* | Whole cell average release flux	$\mu M.ms^{-1}$
$^{m}J_{CaL}$ | I_{CaL}	LTCC flux in dyad *m* | Whole cell current	$\mu M.ms^{-1}$ | pA/pF
$^{m}J_{up}$ | J_{up}	SR Uptake flux in CRU *m* | Whole cell SR update flux	$\mu M.ms^{-1}$
$^{m}J_{leak}$ | J_{leak}	SR leak flux in CRU *m* | Whole cell SR leak flux	$\mu M.ms^{-1}$
$^{m}J_{ds}$	Flux from dyad *m* to rbSS voxel at *m*	$\mu M.ms^{-1}$
$^{m}J_{ss}$	Flux from rbSS to cytoplasm voxel *m*	$\mu M.ms^{-1}$
$^{m}J_{jsr}$	Flux from network to junctional SR at dyad *m*	$\mu M.ms^{-1}$
$^{m}J_{trpn}$	Trpn buffering flux at CRU *m*	$\mu M.ms^{-1}$
$^{m}J_{NaCa}$ | I_{NaCa}	Sodium-Ca^{2+} exchanger flux in CRU *m* | Whole cell current	$\mu M.ms^{-1}$ | pA/pF
$^{m}J_{pCa}$ | I_{pCa}	PMCA Ca^{2+} pump flux in in CRU *m* | Whole cell current	$\mu M.ms^{-1}$ | pA/pF
$^{m}J_{Cab}$ | I_{Cab}	Background Ca^{2+} current flux in voxel *m* | Whole cell current	$\mu M.ms^{-1}$ | pA/pF
$^{m}\beta_{cyto}$	Instantaneous buffering in the cytoplasm in CRU *m*	-
$^{m}\beta_{jSR}$	Instantaneous buffering in the junctional SR in CRU *m*	-
$^{m}n_{o\_ryr}$	Number of open RyRs in dyad *m*	-
^{m}CA	Number of RyRs in the <u>C</u>losed <u>A</u>ctivated state, dyad *m*	-
^{m}OA	Number of RyRs in the <u>O</u>pen <u>A</u>ctivated state, dyad *m*	-
^{m}CI	Number of RyRs in the <u>C</u>losed <u>I</u>nactivated state, dyad *m*	-
^{m}OI	Number of RyRs in the <u>O</u>pen <u>I</u>nactivated state, dyad *m*	-
csqn	Free calsequestrin concentration	mM
^{m}M	Proportion of csqn in monomer state, dyad *m*	-
$^{m}d_1$	LTCC activation gate state 1, dyad *m*	-
$^{m}d_2$	LTCC activation gate state 2, dyad *m*	-
$^{m}d_3$	LTCC activation gate state 3, dyad *m*	-
$^{m}f_1$	LTCC voltage-dependent inactivation state 1, dyad *m*	-
$^{m}f_2$	LTCC voltage-dependent inactivation state 2, dyad *m*	-
$^{m}fca_1$	LTCC Ca^{2+}-dependent inactivation state 1, dyad *m*	-
$^{m}fca_2$	LTCC Ca^{2+}-dependent inactivation state 2, dyad *m*	-
V_m	Membrane potential	mV

Fundamental equations for Ca^{2+} concentration

$$\frac{d[Ca^{2+}]_i}{dt} = \beta_i \left(\mathbf{D}\nabla^2[Ca^{2+}]_i + \phi_i + \left(v_{ss}/v_i\right) J_{ss}\right) \quad (1)$$

$$\frac{d[Ca^{2+}]_{SS}}{dt} = \beta_{SS}\left(\mathbf{D}\nabla^2[Ca^{2+}]_{SS} + \phi_{SS} - J_{ss} + \left(v_{ds}/v_{ss}\right) J_{ds}\right) \quad (2)$$

$$\frac{d[Ca^{2+}]_{nSR}}{dt} = \mathbf{D}\nabla^2[Ca^{2+}]_{nSR} + f_{nSR} - \left(v_{jsr}/v_{nsr}\right) J_{jSR} \quad (3)$$

$$\frac{d\left[Ca^{2+}\right]_{ds}}{dt} = \phi_{ds} - J_{ds} \quad (4)$$

$$\frac{d\left[Ca^{2+}\right]_{JSR}}{dt} = \beta_{JSR}\left(\phi_{JSR} + J_{jSR}\right) \quad (5)$$

Where transfer between compartments is given by:

$$J_{ss} = \left(\left[Ca^{2+}\right]_{SS} - \left[Ca^{2+}\right]_i\right)\tau_{ss}^{-1} \quad (6)$$

$$J_{ds} = \left(\left[Ca^{2+}\right]_{ds} - \left[Ca^{2+}\right]_{SS}\right)\tau_{ds}^{-1} \quad (7)$$

$$J_{jSR} = \left(\left[Ca^{2+}\right]_{nSR} - \left[Ca^{2+}\right]_{JSR}\right)\tau_{ds}^{-1} \quad (8)$$

And the reaction terms are:

$$\phi_i = J_{NaCa} + J_{pCa} + J_{Cab} - \left(J_{up} - J_{leak}\right) - J_{trpn} \quad (9)$$

$$\phi_{nSR} = \left(J_{up} - J_{leak}\right)\left(v_i/v_{nsr}\right) \quad (10)$$

$$\phi_{ss} = J_{NaCa\_SS} \quad (11)$$

$$\phi_{ds} = J_{rel} + J_{CaL} \quad (12)$$

$$\phi_{JSR} = -J_{rel}\left(v_{ds}/v_{jSR}\right) \quad (13)$$

Spatial diffusion is described by a 6-node nearest neighbours finite difference approximation:

$$\mathbf{D}\nabla^2[Ca^{2+}]_x \approx J_{Ca\_diff\_x} = \sum_{i=1}^{i=3}\left(\frac{{}^{e_i+1}[Ca^{2+}]_x + {}^{e_i-1}[Ca^{2+}]_x - 2\,{}^{e_i}[Ca^{2+}]_x}{\tau_{x,e_i}}\right) \quad (14)$$

Where e_i refers to the three dimensions (x,y,z).

Due to the small volume of the dyadic cleft, an analytical description can be found for the dyadic cleft Ca^{2+} concentration under the approximation that the volume reaches its steady-state concentration within the time-step, Δt. Thus, by setting:

$$\frac{d\left[Ca^{2+}\right]_{ds}}{dt}=0 \tag{15}$$

An approximation for equation (4) can be obtained as [2]:

$$\left[Ca^{2+}\right]_{ds}=\left[Ca^{2+}\right]_{SS}+\frac{\tau_{ds}.\left(k_{rel}.\left[Ca^{2+}\right]_{jSR}+J_{CaL}\right)}{\left(1+\tau_{ds}.k_{rel}\right)} \tag{16}$$

Table S2: Cell structure and diffusion parameters

Parameter	Description	Value
v_i	Cytoplasm volume per CRU	1.0 μm³
v_{nsr}	Network SR volume per CRU	0.05 μm³
v_{SS}	Sub-space volume per CRU	0.0175 μm³
$<v_{ds}>$	Average volume of individual dyad	1.712 x10⁻³ μm³
v_{jSR}	Volume individual jSR	0.015 μm³
τ_{ds}	Time constant diffusion from dyad to SS	0.022 ms
τ_{ss}	Time constant diffusion from SS to cytoplasm	0.1 ms
τ_{jSR}	Time constant diffusion from nSR to jSR	5 ms
$\tau_{i,transverse}$	Time constant of transverse cytoplasm diffusion	2.3 ms
$\tau_{i,longitudinal}$	Time constant of longitudinal cytoplasm diffusion	2.9 ms
$\tau_{ss,transverse}$	Time constant of transverse subspace diffusion	1.35 ms*
$\tau_{ss,longitudinal}$	Time constant of longitudinal subspace diffusion	2.20 ms*
$\tau_{nSR,transverse}$	Time constant of transverse nSR diffusion	7 ms
$\tau_{nSR,longitudinal}$	Time constant of longitudinal nSR diffusion	14 ms

** varied in different conditions. These are control model values*

Reaction terms

The formulations for the reaction terms (Equations (9-13)) are described below:

Intracellular Ca^{2+} release, J_{rel}

$${}^{m}J_{rel}={}^{m}k_{rel}\left({}^{m}\left[Ca^{2+}\right]_{jSR}-{}^{m}\left[Ca^{2+}\right]_{ds}\right) \tag{17}$$

$${}^{m}k_{rel}={}^{m}n_{o\_RyR}.g_{RyR}.{}^{m}v_{ds}^{-1} \tag{18}$$

${}^{m}n_{o\_RyR}$ is the number of open RyR channels in dyad m. RyR dynamics is described by a 4-state Markov Chain model. The model is similar to Stern et al [3] and Restrepo et al [4], with a functional monomer induced inactivation based on the csqn dynamics described by Gaur-Rudy [5].

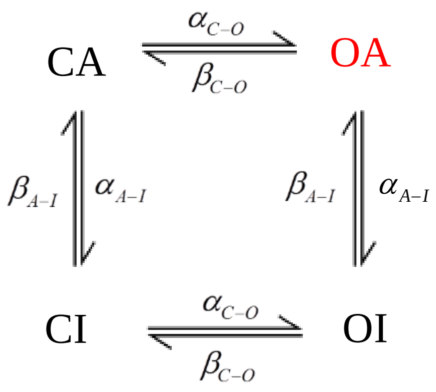

$$\frac{d\,{}^{m}CA}{dt} = {}^{m}OA.\beta_{C-O} + {}^{m}CI.\beta_{A-I} - {}^{m}CA.(\alpha_{C-O} + \alpha_{A-I}) \quad (19)$$

$$\frac{d\,{}^{m}OA}{dt} = {}^{m}CA.\alpha_{C-O} + {}^{m}OI.\beta_{A-I} - {}^{m}OA.(\beta_{C-O} + \alpha_{A-I}) \quad (20)$$

$$\frac{d\,{}^{m}CI}{dt} = {}^{m}OI.\beta_{C-O} + {}^{m}C.\alpha_{A-I} - {}^{m}CI.(\alpha_{C-O} + \beta_{A-I}) \quad (21)$$

$$\frac{d\,{}^{m}OI}{dt} = {}^{m}CI.\alpha_{C-O} + {}^{m}OA.\alpha_{A-I} - {}^{m}OI.(\beta_{C-O} + \beta_{A-I}) \quad (22)$$

Where:

$$\alpha_{C-O} = k_a\left({}^{m}\left[Ca^{2+}\right]_{ds}\right)^{H} \quad (23)$$

$$\beta_{C-O} = k_b \quad (24)$$

$$\alpha_{A-I} = \left(1 - {}^{m}Mi_{ss}\right)/\,\tau_{Mi,1} \quad (25)$$

$$\beta_{A-I} = {}^{m}Mi_{ss}\,/\,\tau_{Mi,2} \quad (26)$$

$${}^{m}Mi_{ss} = 1\Big/\left(1 + e^{({}^{m}M - 0.5)/0.04167}\right) \quad (27)$$

$$\frac{dM}{dt} = \alpha_M(1 - M) + \beta_M M \quad (28)$$

$$\alpha_M = M_{ss}\,/\,\tau_{M,1} \quad (29)$$

$$\beta_M = (1 - M_{ss})\big/\tau_{M,2} \quad (30)$$

$$M_{ss} = 1\big/(1 + e^{(-6.5.(csqn - 6.37))}) \quad (31)$$

$$csqn = B_{csqn}.K_{mcsqn}\Big/\left({}^{m}\left[Ca^{2+}\right]_{jSR} + K_{mcsqn}\right) \quad (32)$$

In this model, OA is the only state in which a flux occurs. Thus, ${}^{m}n_{o\_RyR}$ is equal to the number of channels in dyad m which are in state OA (red text in schematic).

L-type Ca^{2+} channel flux, J_{CaL}

The flux through the L-type Calcium Current is defined as:

$$ {}^{m}J_{CaL} = -{}^{m}n_{o\_LTCC}\,{}^{m}\bar{J}_{CaL} \tag{33}$$

Where ${}^{m}n_{o\_LTCC}$ is the number of open LTCC channels in dyad m (defined below) and $\bar{J}_{CaL}$ is the maximal flux rate per channel [6]:

$$ {}^{m}\bar{J}_{CaL} = 4P_{Ca}zF\,\frac{\frac{1}{2}\gamma_{Ca}\,{}^{m}\left[Ca^{2+}\right]_{ds}e^{2z} - \gamma_{Ca}\left[Ca^{2+}\right]_{o}}{e^{2z}-1} \tag{34}$$

$$ z = \frac{V_m F}{RT} \tag{35}$$

Where $[Ca^{2+}]_o$ is the extracellular Ca^{2+} concentration, P_{Ca} is the maximum permeability of an individual LTCC and F is the Faraday constant.

The LTCCs are described by a Markov Chain construction of a Hodgkin-Huxley model [6,7]

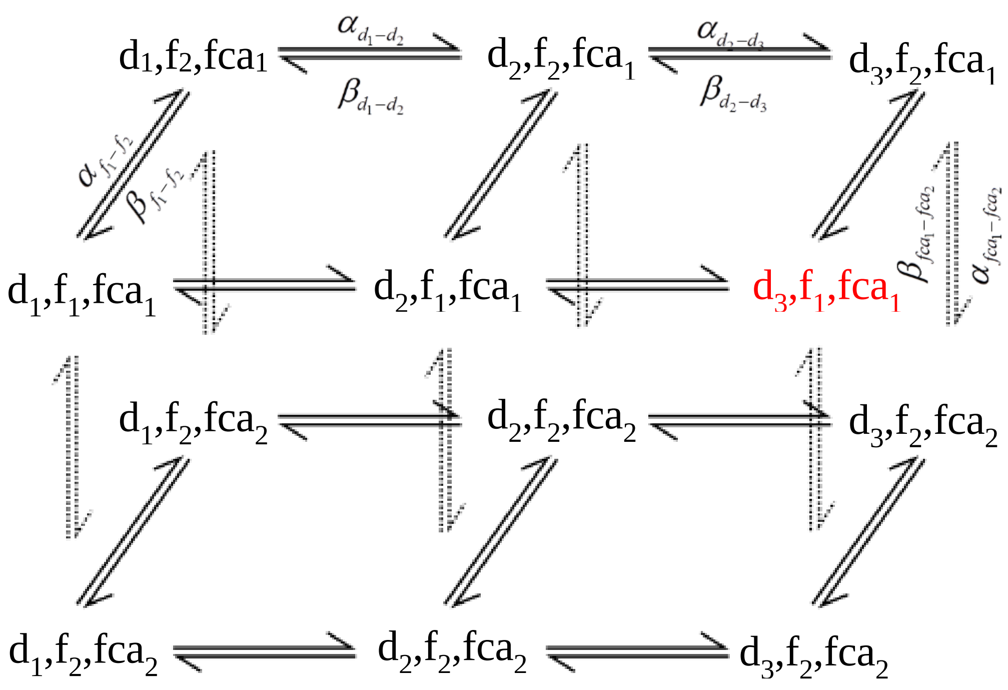

Which is equivalent to the three gate Hodgkin-Huxley model:

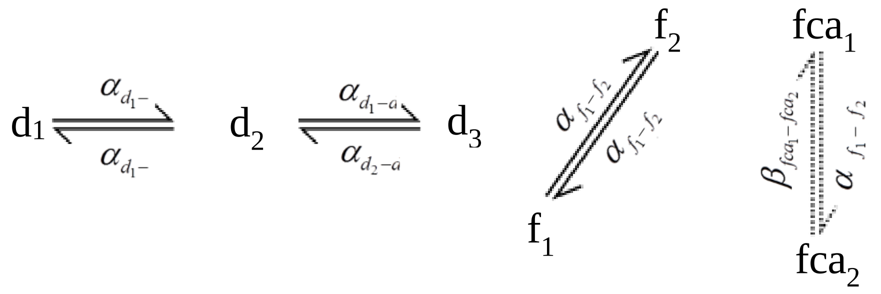

And thus described by:

$$\frac{d(d_1)}{dt} = d_2\beta_{d_1-d_2} - d_1\alpha_{d_1-d_2} \tag{36}$$

$$\frac{d(d_2)}{dt} = d_1\alpha_{d_1-d_2} + d_3\beta_{d_2-d_3} - d_2(\beta_{d_1-d_2} + \alpha_{d_2-d_3}) \tag{37}$$

$$\frac{d(d_3)}{dt} = d_2\alpha_{d_2-d_3} - d_3\beta_{d_2-d_3} \tag{38}$$

$$\frac{d(f_1)}{dt} = f_2\beta_{f_1-f_2} - f_1\alpha_{f_1-f_2} \tag{39}$$

$$\frac{d(fca_1)}{dt} = fca_2\beta_{fca_1-fca_2} - fca_1\alpha_{fca_1-fca_2} \tag{40}$$

Where the transition rates for each variable couplet, (x = d_1-d_2, f_1-f_2, fca_1-fca_2) are defined from the steady-state and time constant in the standard way:

$$\alpha_x = x_{ss} / \tau_x \tag{41}$$

$$\beta_x = (1-x_{ss}) / \tau_x \tag{42}$$

And:

$$\alpha_{d_2-d_3} = k_{d2d3} \tag{43}$$

$$\beta_{d_2-d_3} = k_{d3d2} \tag{44}$$

$$d_{ss} = 1\Big/\left(1+e^{(-(V_m-5)/6.24)}\right) \tag{45}$$

$$f_{ss} = 1-1\Big/\left(1+e^{((V_m+32.06)/8.6)}\right) \tag{46}$$

$$\tau_d = d_{ss}.\left(1-e^{(-(V_m-5)/6.24)}\right)\Big/\left(0.035(V_m-5)\right) \tag{47}$$

$$\tau_f = 2\Big/\left(0.0197e^{-\left([0.0337(V_m+7)]^2+0.02\right)}\right) \tag{48}$$

$$fca_{ss} = 1-1\Big/\left(1+\left({}^{m}\left[Ca^{2+}\right]_{ds}\Big/\bar{C}a\right)^2\right) \tag{49}$$

Note that the steady states of the inactivation gates (*f, fca*) are inverse to those in the standard Hodgkin-Huxley model because in this Markov description f_2 is the inactivated state, equivalent to $(1-f)$ in the standard description (and f_1 is equivalent to f).

Table S3: RyR and LTCC flux parameters

Parameter	Description	Value
g_{ryr}	Maximal flux rate through the RyRs	2.05 x 10^{-4} $\mu m^3\,ms^{-1}$
P_{Ca}	Maximum permeability of LTCC	11.9 $\mu mol\,C^{-1}\,ms^{-1}$
γ_{Ca}	Activity Coefficient LTCC	0.341
N_{RyR}	Number of RyRs per dyad*	100
H	RyR Open rate Ca^{2+} power	2.5
k_a	RyR activation rate constant	1.58 x 10^{-4} $\mu M^{-2.5}\,ms^{-1}$
k_b	RyR deactivation rate constant	1.0 ms^{-1}
$\tau_{M,1}$	Time constant of monomer binding	25 ms
$\tau_{Mi,1}$	Time constant of monomer inactivation	30 ms
$\tau_{M,2}$	Time constant monomer unbinding	156 ms
$\tau_{Mi,2}$	Time constant of de-inactivation	75 ms
N_{LTCC}	Number of L-type Ca^{2+} channels per dyad*	15
k_{d2d3}	Rate constant for transition d_2-d_3	0.3 ms^{-1}
k_{d3d2}	Rate constant for transition d_3-d_2	6.0 ms^{-1}
τ_{fca}	Time constant for Ca^{2+} induced inactivation	15 ms
$\bar{Ca}$	Ca^{2+} constant for Ca^{2+} induced inactivation	6.0 μM
$[Ca^{2+}]_o$	Extracellular Ca^{2+} concentration	1.8 mM

Intracellular uptake and leak, J_{up} and J_{leak}

These equations are based on Restrepo et al [4] and preceding studies [8,9].

$$ {}^{m}J_{up} = g_{up}\frac{\left({}^{m}\left[Ca^{2+}\right]_i / K_{cyto}\right)^2 - \left({}^{m}\left[Ca^{2+}\right]_{nSR} / K_{nSR}\right)^2}{1+\left({}^{m}\left[Ca^{2+}\right]_i / K_{cyto}\right)^2 + \left({}^{m}\left[Ca^{2+}\right]_{nSR} / K_{nSR}\right)^2} \quad (50) $$

$$ {}^{m}J_{leak} = g_{leak}\frac{{}^{m}\left[Ca^{2+}\right]_{nSR}^2}{{}^{m}\left[Ca^{2+}\right]_{nSR}^2 + K_{leak}^2}\left({}^{m}\left[Ca^{2+}\right]_{nSR} - {}^{m}\left[Ca^{2+}\right]_i\right) \quad (51) $$

Table S4: Ca^{2+} uptake and leak parameters

Parameter	Description	Value
g_{up}	Maximal flux rate of J_{up}	0.339 $\mu M.ms^{-1}$
K_{cyto}	Cytoplasm constant for J_{up}	0.15 μM
K_{nSR}	Network SR constant for J_{up}	1700 μM
g_{leak}	Maximal flux rate of J_{leak}	1.412 x10^{-5} ms^{-1}
K_{leak}	J_{leak} constant	450 μM

Membrane fluxes, J_{NaCa}, J_{pCa} and J_{CaB}

These equations are based on Restrepo et al [4] and preceding studies [8,9].

$$ {}^{m}J_{NaCa} = \frac{K_a g_{NaCa} v_{vox}^{-1} \left(e^{\eta z} \left[Na^+\right]_i \left[Ca^{2+}\right]_o - e^{(\eta-1)z} \left[Na^+\right]_o {}^{m}\left[Ca^{2+}\right]_{cyto} \right)}{(t_1 + t_2 + t_3)\left(1 + K_{sat} e^{(\eta-1)z}\right)} \quad (52) $$

$$ {}^{m}J_{pCa} = \left(v_i^{-1} g_{pCa} {}^{m}\left[Ca^{2+}\right]_i \right) \Big/ \left(K_{mpCa} + {}^{m}\left[Ca^{2+}\right]_i \right) \quad (53) $$

$$ {}^{m}J_{Cab} = v_{vox}^{-1} g_{Cab} \left(V_m - E_{r,Ca} \right) \quad (54) $$

Where

$$ t_1 = K_{mCai} \left[Na^+\right]_o^3 \left(1 + \left(\left[Na^+\right]_i \big/ K_{mNai} \right)^3 \right) \quad (55) $$

$$ t_2 = K_{mNao}^3 {}^{m}\left[Ca^{2+}\right]_i \left(1 + \left({}^{m}\left[Ca^{2+}\right]_i \big/ K_{mCai} \right) \right) \quad (56) $$

$$ t_3 = K_{mCao} \left[Na^+\right]_i^3 + \left[Na^+\right]_i^3 \left[Ca^{2+}\right]_o + \left[Na^+\right]_o^3 {}^{m}\left[Ca^{2+}\right]_i \quad (57) $$

$$ K_a = \left[1 + \left(K_{da} \big/ {}^{m}\left[Ca^{2+}\right]_i \right) \right] \quad (58) $$

$$ z = \frac{V_m F}{RT} \quad (59) $$

Table S5: Membrane flux parameters

Parameter	Description	Value
g_{NaCa}	Maximal flux rate of Sodium-Ca^{2+} exchanger	0.3726 $\mu m^3 . \mu M.ms^{-1}$
K_{da}	Ca^{2+} scaling constant	0.11 μM
η	Voltage sensitivity coefficient	0.35
K_{sat}	Saturation constant	0.27
K_{mcai}	Intracellular Ca^{2+} constant	3.59 μM
K_{mcao}	Extracellular Ca^{2+} constant	1.3 mM
K_{mnai}	Intracellular Na^+ constant	12.3 mM
K_{mnao}	Extracellular Na^+ constant	87.5 mM
g_{pca}	Maximal flux rate of PMCA Ca^{2+} pump	1.37 x10^{-3} $\mu m^3 . \mu M.ms^{-1}$
g_{cab}	Maximal flux rate of background Ca^{2+} current	1.82 x10^{-5} $\mu m^3 . \mu M.ms^{-1}.mV^{-1}$
$[Na^+]_i$	Intracellular Na^+ concentration	7.95 mM
$[Na^+]_o$	Extracellular Na^+ concentration	136 mM

Ca^{2+} Buffering

Instantaneous buffering in the cytoplasm

Instantaneous buffering in the cytoplasm follows that of previous models, e.g., Restrepo et al. [4] and Nivala et al. [10], based on [11]. At each voxel, *m*, the buffering term is given by:

$$^{m}\beta_{cyto,SS} = \left[1 + C_{cyto,SS} \sum_{x} \frac{B_x K_x}{\left(^{m}[Ca^{2+}]_{cyto,SS} + K_x \right)^2} \right]^{-1} \quad (60)$$

Where *x* refers to four buffering processes: Calmodulin, SR sites, Myosin (Ca) and Myosin (Mg).

Instantaneous buffering in the jSR

Buffering in the jSR follows that of the previous study Gaur-Rudy [5]:

$$^{m}\beta_{jSR} = \left[1 + \frac{B_{csqn} K_{mcsqn}}{\left(^{m}[Ca^{2+}]_{jSR} + K_{mcsqn} \right)^2} \right]^{-1} \quad (61)$$

Troponin buffering and force

Troponin buffering and force generation is from the Gauthier et al model [12,13]:

$$^{m}J_{trpn} = \frac{dH_{trpn,Ca}}{dt} + \frac{dL_{trpn,Ca}}{dt} \quad (62)$$

$$\frac{d\,^{m}H_{trpn,Ca}}{dt} = k^{+}_{H,trpn}\, ^{m}\left[Ca^{2+}\right]_{cyto} \left(B_{H,trpn} - H_{trpn,Ca} \right) - k^{-}_{H,trpn} H_{trpn,Ca} \quad (63)$$

$$\frac{d\,^{m}L_{trpn,Ca}}{dt} = k^{+}_{L,trpn}\, ^{m}\left[Ca^{2+}\right]_{cyto} \left(B_{L,trpn} - L_{trpn,Ca} \right) - k^{-}_{L,trpn} \left(1 - \frac{2}{3} F_{norm} \right) L_{trpn,Ca} \quad (64)$$

Where F_{norm} is the normalised force:

$$F_{norm} = \left(\frac{P_1 + N_1 + 2P_2 + 3P_3}{P_1^{\max} + 2P_2^{\max} + 3P_3^{\max}} \right) \quad (65)$$

And:

$$\phi = 1 + \frac{2.3 - SL}{(2.3 - 1.7)^{1.6}} \quad (66)$$

$$f_{01} = 3 f_{XB} \quad (67)$$

$$f_{12} = 10 f_{XB} \quad (68)$$

$$f_{23} = 7 f_{XB} \quad (69)$$

$$g_{01} = g_{XB} \quad (70)$$

$$g_{12} = 2 g_{XB} \quad (71)$$

$$g_{23} = 3g_{XB} \tag{72}$$

$$g_{01,SL} = 1\phi g_{XB,\min} \tag{73}$$

$$g_{12,SL} = 2\phi g_{XB,\min} \tag{74}$$

$$g_{23,SL} = 3\phi g_{XB,\min} \tag{75}$$

$$K_{TRPN}^{Ca} = \frac{k_{L,TRPN}^{-}}{k_{L,TRPN}^{+}} \tag{76}$$

$$N_{TRPN} = 3.5SL - 2.0 \tag{77}$$

$$K_{1/2}^{TRPN} = \left[1 + \frac{K_{TRPN}^{Ca}}{1.4\times10^{-3} - 8\times10^{-4}\left((SL-1.7)/0.6\right)}\right]^{-1} \tag{78}$$

$$k_{np,TRPN} = k_{pn,TRPN}\left(\frac{\left[TRPN_{Ca}^{L}\right]}{K_{1/2}TRPN_{tot}^{L}}\right)^{N_{TRPN}} \tag{79}$$

$$\sum Paths = g_{01}g_{12}g_{23} + f_{01}g_{12}g_{23} + f_{01}f_{12}g_{23} + f_{01}f_{12}f_{23} \tag{80}$$

$$P_1^{\max} = \frac{f_{01}g_{12}g_{23}}{\sum Paths} \tag{81}$$

$$P_2^{\max} = \frac{f_{01}f_{12}g_{23}}{\sum Paths} \tag{82}$$

$$P_3^{\max} = \frac{f_{01}f_{12}f_{23}}{\sum Paths} \tag{83}$$

$$\frac{dP_0}{dt} = -\left(k_{pn,TRPN} + f_{01}\right)P_0 + k_{np,TRPN}N_0 + g_{01,SL}P_1 \tag{84}$$

$$\frac{dP_1}{dt} = -\left(k_{pn,TRPN} + f_{12} + g_{01,SL}\right)P_1 + k_{np,TRPN}N_1 + f_{01}P_0 + g_{12,SL}P_1 \tag{85}$$

$$\frac{dP_2}{dt} = -\left(f_{23} + g_{12,SL}\right)P_2 + f_{12}P_1 + g_{23,SL}P_3 \tag{86}$$

$$\frac{dP_3}{dt} = -g_{23,SL}P_3 + f_{23,SL}P_2 \tag{87}$$

$$\frac{dN_1}{dt} = k_{pn,TRPN}P_1 - \left(k_{np,TRPN} + g_{01,SL}\right)N_1 \tag{88}$$

$$\frac{dN_0}{dt} = \frac{dP_0}{dt} - \frac{dP_1}{dt} - \frac{dP_2}{dt} - \frac{dP_3}{dt} - \frac{dN_1}{dt} \tag{89}$$

Table S6: Ca^{2+} buffering parameters

Parameter	Description	Value
K_{CAM}	Dissociation constant for calmodulin	7.0 μM
B_{CAM}	Total concentration buffering sites	24.0 μM
K_{SR}	Dissociation constant for SR sites	0.6/0.9 μM (cyto/SS)
B_{SR}	Total concentration buffering sites	47.0 μM
$K_{M,Ca}$	Dissociation constant for Myosin (Ca)	0.033/0.0615 μM (cyto/SS)
$B_{M,Ca}$	Total concentration buffering sites	140.0 μM
$K_{M,Mg}$	Dissociation constant for Myosin (Mg)	3.64/5.46 μM (cyto/SS)
$B_{M,Mg}$	Total concentration buffering sites	140 μM
$C_{cyto,SS}$	Buffering strength coefficient	1 (cyto), 0.1 (SS)
K_{mcsqn}	Dissociation constant for csqn	0.8 mM
B_{csqn}	Total concentration buffering sites	10 mM
$k^{+}_{H,\ trpn}$	On rate for troponin high affinity sites	100 $mM^{-1}\ ms^{-1}$
$k^{-}_{H,\ trpn}$	Off rate for troponin high affinity sites	1.0 x10^{-3} ms^{-1}
$k^{+}_{L,\ trpn}$	On rate for troponin low affinity sites	100 $mM^{-1}\ ms^{-1}$
$k^{-}_{L,\ trpn}$	Off rate for troponin low affinity sites	4.0 x 10^{-2} ms^{-1}
$B_{H,trpn}$	Total high affinity sites on troponin	0.14 mM
$B_{L,trpn}$	Total low affinity sites on troponin	0.7 mM
f_{XB}	Weak to strong cross bridge transition rate	0.05 ms^{-1}
$g_{XB,min}$	Maximum strong to weak rate	0.1 ms^{-1}
SL	Sarcomere length	2.15 μm
$K_{pn,TRPN}$	Permissive to non-permissive transition rate	0.04 ms^{-1}

2. The 0D, deterministic model

Except for the RyR, the deterministic model comprises the same components as the 3D cell model for one CRU, with the LTCC dynamics solved by the forward-Euler method. However, due to the poor capitulation of CICR by deterministic solutions to the RyR model, adjustments were performed: The dyadic Ca^{2+} concentration seen by the RyRs was solved to be dependent only on I_{CaL}, allowing more continuous behaviour in RyR opening, and k_a set to 2.37×10^{-3} $\mu M^{-2.5}\ ms^{-1}$. The open transition rate is therefore given as:

$$\alpha_{C-O} = k_a \left({}^{m}\left[Ca^{2+}\right]_{SS} + \tau_{ds} . {}^{m}J_{CaL} \right)^{H} \tag{90}$$

3. Hybrid-minimal AP model

The hybrid-minimal model was designed to give a controllable AP while maintaining physiological ion current magnitudes for integration with the calcium dependent currents of the 3D and 0D calcium handling models. In addition to the calcium currents, the model comprises:

Phase-0 depolarising current (I_{p0d}): modelled exactly the same as the LRd I_{Na} formulation [6].

Phase-1 repolarising current (I_{p1r}):

$$I_{p1r} = g_{p1r}.va_{p1r}.vi_{p1r}.(V_m + 88) \tag{91}$$

$$va_{p1r\_ss} = 1/\left(1+e^{-(V_m-1)/11.0}\right) \tag{92}$$

$$vi_{p1r\_ss} = 1/\left(1+e^{(V_m+40)/11.5}\right) \tag{93}$$

$$va_{p1r\_\tau} = 3.5e^{-(V_m/30)^2} + 1.5 \tag{94}$$

$$va_{p1r\_\tau} = 25.6e^{-((V_m+52)/15)^2} + 14 \quad (95)$$

Phase-2 repolarising current (I_{p2r}):

$$I_{p2r} = g_{p2r}.va_{p2r}.vi_{p2r}.(V_m + 88) \quad (96)$$

$$va_{p2r\_ss} = 1/\left(1 + e^{-(V_m+6)/8.6}\right) \quad (97)$$

$$vi_{p2r\_ss} = 1/\left(1 + e^{(V_m+7.5)/10}\right) \quad (98)$$

$$va_{p2r\_\tau} = 9e^{-((V_m+5)/12)} + 0.5 \quad (99)$$

$$va_{p2r\_\tau} = 590e^{-((V_m+60)/10)^2} + 3000 \quad (100)$$

Phase-3 repolarising current (I_{p3r}):

$$I_{p3r} = g_{p3r}.va_{p3r}.vti_{p3r}.(V_m + 88) \quad (101)$$

$$va_{p3r\_ss} = 1/\left(1 + e^{-(V_m+14)/6.5}\right) \quad (102)$$

$$vti_{p3r} = 1/\left(1 + e^{(V_m+15)/22.4}\right) \quad (103)$$

$$va_{p3r\_a} = 0.0003(V_m + 14)\Big/\left(1 + e^{-(V_m+14)/5}\right) \quad (104)$$

$$va_{p3r\_b} = 0.000074(V_m - 3.3)\Big/\left(e^{-(V_m-3.3)/5} - 1\right) \quad (105)$$

$$va_{p3r\_\tau} = 1\Big/\left(va_{p3r\_a} + va_{p3r\_b}\right) \quad (106)$$

Phase-4 repolarising current (I_{p4r}):

$$I_{p4r} = g_{p4r}.\left(1 + 0.07e^{(V_m+80)}\right)^{-1}.(V_m + 88) \quad (107)$$

Table S7: Minimal model heterogeneity parameters

Parameter	Vent EPI	Vent M	Vent ENDO	Atria RA
g_{p0d} **(s/mF)**	16	16	16	16
g_{p1r} **(s/mF)**	0.081	0.081	0.081	0.289
g_{p2r} **(s/mF)**	0.0034	0.0034	0.0034	0.0204
g_{p3r} **(s/mF)**	0.05	0.03	0.045	0.0125
g_{p4r} **(s/mF)**	0.3	0.3	0.3	0.15

4. Non-specific remodelling and ISO models

Representative (but non-specific) models were included for isoprenaline (ISO, sympathetic response which enhances CICR) and two types of pro-SCRE general disease remodelling mimicking features observed in conditions such as AF and HF: (i) SERCA was up-regulated and NCX was down-regulated ($R_{SERCA/NCX}$); (ii) the SR-Ca^{2+} threshold for release was lowered through increased inter-CRU coupling ($R_{CRU\text{-}CRU}$).

Remodelling 1 – "$R_{SERCA/NCX}$":

Table S8: Scaling factors and voltage shifts for remodelling model $R_{SERCA/NCX}$

Parameter	EPI	ENDO	M	RA	ORD	Col
g_{K1}/g_{p4r}	0.5	0.5	0.5	0.25	0.5	0.5
I_{k1} V-shift	10 mV	10 mV	10 mV	10 mV	10 mV	10 mV
g_{CaL}	0.5	0.5	0.5	2	0.5	0.5
g_{Kr}/g_{p3r}	1.5	1.5	1.5	0.5	0.666	1.5
g_{Na}/g_{p0d}	0.5	0.5	0.5	0.5	0.5	0.5
I_{NCX_max}	0.5	0.5	0.5	0.5	0.5	0.5
J_{up_max}	1.5	1.5	1.5	1.5	1.25	1.5

Remodelling 2 – "$R_{CRU-CRU}$" simply involved setting the time constants of sub-space coupling to 1.0 and 1.5 ms.

ISO:

Table S9: Scaling factors ISO model

Parameter	EPI	ENDO	M	RA	ORD	Col
g_{to}/g_{p1r}	1	1	1	1.5	1	1.5
g_{Kur}/g_{p2r}	2	2	2	1.5	1	1.5
g_{k}/g_{p3r}	2	2	2	1	2	1.5
J_{up_max}	2	2	2	1.2	1.75	1.7
I_{CaL} - k_{d2d3}	2	2	2	2	2	2

5. Tissue models

Tissue simulations were performed using the homogeneous, isotropic approximation to the monodomain equation describing spatial coupling of the membrane potential:

$$\mathbf{D}\nabla^2\, {}^{e_i}Vm \approx \frac{D}{\Delta x^2}\sum_{i=1}^{i=3}\left({}^{e_i+1}Vm + {}^{e_i-1}Vm - 2\,{}^{e_i}Vm\right) \tag{108}$$

Where ${}^{ei}V_m$ is the membrane potential at the relevant cell, the subscript i refers to the three spatial-dimensions, $\boldsymbol{D}$ is the isotropic diffusion coefficient, Δx is the spatial step and ∇^2 is the spatial Laplacian operator in 3D.

The basic 2D idealised tissue model, used for analysis of the emergence of focal excitation and the impact of SCRE variability on the focal-SR-Ca^{2+} relationship, comprised a grid of 100 × 100 cells with a spatial resolution of 0.2 mm and an isotropic diffusion coefficient, $\boldsymbol{D}$, of 0.1 mm^2/ms, giving a conduction velocity of 0.56 m/s. Re-entrant (and matched pacing) simulations were performed in a larger tissue model of either 200 × 200 or 400 × 400 cells, at a spatial resolution of 0.25 mm. $\boldsymbol{D}$ ranged from 0.1 mm^2/ms (control) to 0.05 mm^2/ms (most reduced), giving conduction velocities of 0.54-0.34 m/s. The idealised model of the heterogeneous ventricular transmural strand, used in the investigation of SCRE and conduction block, comprised a sheet of 200 × 100 cells, with heterogeneity evenly distributed in the x-direction (ratio of 1:1:1 ENDO:M:EPI); the remaining parameters were identical to the basic sheet described above.

Parameters for the anatomically detailed tissue models are as follows: (1) human ventricular wedge reconstruction – spatial step = 0.3 mm, D = 0.04 mm^2/ms and conduction velocity = 0.26 m/s; (2) whole canine ventricle – spatial step = 0.5 mm, D = 0.1 mm^2/ms and conduction velocity = 0.39 m/s; (3) whole human atria – spatial step = 0.33 mm, D = 0.3 mm^2/ms and conduction velocity = 0.98 m/s. All conduction velocities were calculated using the hybrid minimal cell model.

6. Spontaneous release functions

RyR waveforms

The N_{RyR_O} waveforms can be grouped into two primary types: spike-like associated with short, large-amplitude release, and plateau-like associated with long, small-amplitude release. For the spike-like morphology, the waveform can be well approximated with the simple function:

$$N_{RyR\_O} = N_{RyR\_O}^{peak}\left[\left(1+e^{-(t-t_1)/k_1}\right)\left(1+e^{-(t-t_2)/k_2}\right)\right]^{-1} \quad (109)$$

$$t_1 = t_i + 0.5\left(t_p - t_i\right) \quad (110)$$

$$t_2 = t_p + 0.5\left(t_f - t_p\right) \quad (111)$$

$$k_1 = 0.1689(t_p - t_i) + 0.00255 \quad (112)$$

$$k_2 = 0.1689\left(t_f - t_p\right) + 0.00255 \quad (113)$$

where t_i is the initiation time of the SCRE, t_f is the end time (duration, λ, thus = t_f-t_i), t_p is the time of the peak of the waveform and $N_{RyR\_O}^{peak}$ is the peak of open proportion RyR. The function for the plateau-like waveform (corresponding to durations longer than 300 ms) is derived from the same parameters:

$$N_{RyR\_O} = \begin{matrix} N_{RyR\_O}^{plateau}\left[\left(1+e^{-(t-(t_i+17.5))/5.946}\right)\left(1+e^{(t-(t_f-17.5))/5.946}\right)\right]^{-1} + \\ \left(N_{RyR\_O}^{peak} - N_{RyR\_O}^{plateau}\right)\left[\left(1+e^{-(t-(t_p-25))/5.946}\right)\left(1+e^{(t-(t_p+17.5))/5.946}\right)\right]^{-1} \end{matrix} \quad (114)$$

Where $N_{RyR\_O}^{plateau}$ is the amplitude of the plateau. This equation assumes the same form for the spike occurring within the plateau, with its upstroke time being 50 ms and its decay time 35ms; t_i^{spike} therefore corresponds to t_p-50 (and its half maximal activation time t_p-25).

The waveform is therefore completely described by four-five parameters: (1) initiation time, t_i; (2) duration (λ = t_f-t_i); (3) peak time, t_p; and (4-5) amplitude ($N_{RyR\_O}^{peak}$; $N_{RyR\_O}^{plateau}$). In order to maintain physiological waveforms and randomly sample the parameter values from appropriate distributions, the nature of stochastic variation of these four parameters is discussed below.

Parameter distributions

(1) - t_i: The probability density functions for the initiation time associated with each SR-Ca^{2+} value do not demonstrate a normal distribution, but rather a skewed distribution. The cumulative frequency is well approximated by the use of two simple sigmoidal functions, maintaining the desire for restraint in the number of parameters and allowing simple and intuitive controllability:

$$F(t_i) = \begin{cases} F_1(t_i) = \left(2CF_{t_{i,Sep}}\right)\left(1+e^{-\left(t_i - t_{i,sep}\right)/k_{F1}}\right)^{-1} & t_i < t_{i,Sep} \\ F_2(t_i) = \left(2\left(1-CF_{t_{i,Sep}}\right)\right)\left(1+e^{-\left(t_i - t_{i,sep}\right)/k_{F2}}\right)^{-1} - 1 + 2CF_{t_{i,Sep}} & t_i \geq t_{i,Sep} \end{cases} \quad (115)$$

The distribution for t_i is therefore determined by four parameters: the initiation time corresponding to the point where the functions are separated ($t_{i,Sep}$); the cumulative frequency at this point ($CF_{ti,Sep}$= $F(t_i)\,|_{ti=ti,Sep}$), and the gradient parameter of each function (k_{F1}, k_{F2} - corresponding to the width of the distribution either side of $t_{i,Sep}$.)

(2) – λ: The distributions for the duration are also non-normal, and well approximated by two sigmoidal functions describing the cumulative frequency for half of the data either side of the median duration (*MD*):

$$F(MD) = \left. \begin{matrix} F_{D1}(MD) = \left(1 + e^{-(\lambda - MD)/0.261DW_1}\right)^{-1} \\ F_{D2}(MD) = \left(1 + e^{-(\lambda - MD)/0.261DW_2}\right)^{-1} \end{matrix} \right\} \begin{matrix} \lambda < MD \\ \lambda \geq MD \end{matrix} \tag{116}$$

Where the widths (DW_1, DW_2, in ms) are a function of the *MD*, given by:

$$DW_1 = A_{DW1}\left(1 + e^{-(MD - a_{DW1})/k_{DW1}}\right)^{-1} + DW_1^{\min} \tag{117}$$

$$DW_2 = A_{DW2}\left(1 + e^{-(MD - a_{DW2})/k_{DW2}}\right)^{-1} + DW_2^{\min} \tag{118}$$

Default parameters are given in Table S10. The duration distribution is therefore completely described by the median, *MD*. Note that the widths (DW_1, DW_2) could also be specified directly for complete control over the variability in duration. Note also that, at individual conditions (such as SR-Ca^{2+} concentration) there is no strong correlation between t_i and λ and thus it is reasonable to sample these parameters independently (Figure S1).

(3) - t_p: The timing of the peak varies approximately evenly within the duration of the wavefrom, occurring between 25 ms after the initiation (t_i) and 52 ms before the final time (t_f).

(4) - $N_{RyR\_O}^{peak}$; $N_{RyR\_O}^{plateau}$: The amplitude correlates strongly with duration, λ:

$$\left\langle N_{RyR\_O}^{peak} \right\rangle = 692.99\lambda^{-1.6} + 0.059 \tag{119}$$

$$\left\langle N_{RyR\_O}^{plateau} \right\rangle = 31.09(0.01\lambda)^{-7.39} + 0.034 \quad \Big\} if\, \lambda > 300ms \tag{120}$$

With small variance (± 0.025; ± $0.125N_{RyR\_O}^{plateau}$). Note that it would not be appropriate to define the amplitude independently from the duration, due to the correlation between these two parameters corresponding to the total amount of Ca^{2+} released.

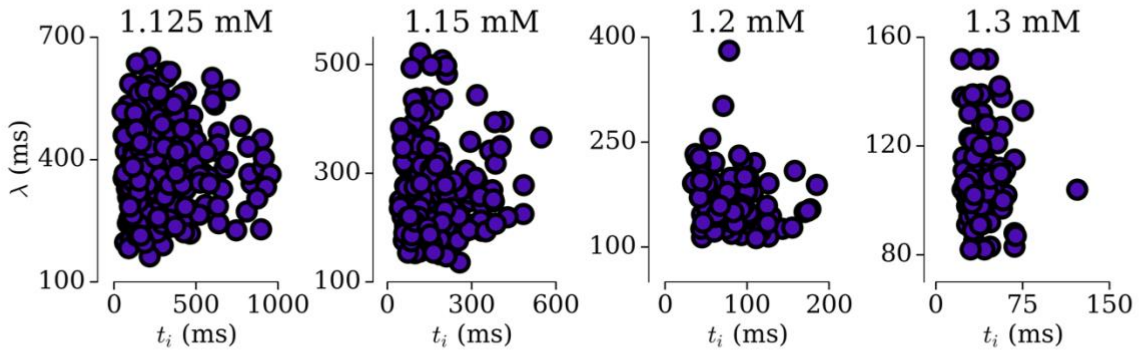

Figure S1: Independence of t_i and λ parameters. t_i and λ plotted against each other for distributions emerging at multiple SR-Ca2+ concentrations (panel titles). Note that while the distributions are not normal, the two parameters exhibit no strong correlation to each other.

The Direct Control SRF model

With this setup, therefore, all parameters of the waveform are derived from two primary waveform properties: the initiation time, t_i, and the duration, λ, which also determine the peak time and amplitude; the distributions describing the variability of these properties is entirely described by 5-7 parameters ($t_i = f(t_{i\_sep}, CF_{ti\_sep}, k_{F1}, k_{F2})$; $\lambda = f(MD, DW_1, DW_2)$ where DW_1, $DW_2 = f(MD)$ or specified). For the simplest implementation of SCRE in 0D models, the user Direct Control model, SRF variability and morphology can therefore be described by simply defining these 5-7 parameters (as well as a probability of SCRE) in order to reproduce single conditions (for example, fit to a single dataset).

The Dynamic Fit SRF model

The Dynamic Fit SRF model was derived through correlation of the parameters defining the t_i and λ distributions with the primary environment variable controlling SCRE: the SR-Ca^{2+} concentration (Figure S2). Relation to this single variable was chosen for practicality and simplicity of the resulting equations, which is in particularly valuable for reproducing variable Ca^{2+} handling system states.

The probability that an SCRE occurs at a given SR-Ca^{2+} is well-approximated by a sigmoidal function:

$$P(SCR) = \left(1 + e^{-\left([Ca^{2+}]_{SR} - SCR_{threshold}\right)/k_{P(SCR)}}\right)^{-1} \qquad (121)$$

The SR-Ca^{2+} dependence of t_{i_sep} and CF_{ti_Sep} can be approximated by the following functions:

$$t_{i,Sep} = A_{t_{i,Sep}} e^{-\left([Ca^{2+}]_{SR} - a_{t_{i,Sep}}\right)/k_{t_{i,Sep}}} + t_{i,Sep}^{\min} \qquad (122)$$

$$CF_{t_{i,Sep}} = A_{CF}\left(1 + e^{-\left([Ca^{2+}]_{SR} - a_{CF}\right)/k_{CF}}\right)^{-1} + 0.05 \qquad (123)$$

The gradient parameters for $F_1(t_i)$ and $F_2(t_i)$, k_{F1} and k_{F2}, are then approximated by the following functions:

$$k_{F_1} = A_{kF1} e^{-\left([Ca^{2+}]_{SR} - a_{kF1}\right)/k_{kF1}} + k_{F_1}^{\min} \qquad (124)$$

$$k_{F_2} = A_{kF2}[Ca^{2+}]_{SR}^{\;H1_{kF2}} + B_{kF2}[Ca^{2+}]_{SR}^{\;H2_{kF2}} + k_{F_2}^{\min} \qquad (125)$$

And finally, the MD also correlates well with the SR-Ca^{2+}:

$$MD = A_{MD} e^{-\left([Ca^{2+}]_{SR} - a_{MD}\right)/k_{MD}} + MD^{\min} \qquad (126)$$

Parameters for the fit achieved for the different model conditions are given in Table S10.

Table S10: Spontaneous release function variables SR-Ca^{2+} dependence parameters, Dynamic Fit model

Symbol	Parameter	Value control	Value $R_{SERCA/NCX}$	Value $R_{CRU\text{-}CRU}$
$\boldsymbol{SCR}_{\mathbf{threshold}}$	SR-Ca^{2+} threshold for SCRE (mM)	1.091	0.993	0.963
$\boldsymbol{k}_{\mathbf{P(SCR)}}$	Gradient parameter for *P(SCR)*	0.0058	0.007	0.0075
$\boldsymbol{A}_{\mathbf{ti,Sep}}$	Coefficient for $t_{i,Sep}$ function	37.78	27.62	65.047
$\boldsymbol{a}_{\mathbf{ti,Sep}}$	$t_{i,Sep}$ exponent $[Ca^{2+}]_{SR}$ reference parameter	1.157	1.096	1.017
$\boldsymbol{k}_{\mathbf{ti,Sep}}$	$t_{i,Sep}$ exponent $[Ca^{2+}]_{SR}$ gradient parameter	0.041	0.0581	0.0674
$\boldsymbol{t}_{\mathbf{i,Sep}}^{\mathbf{min}}$	Minimal value for $t_{i,Sep}$	31.83	18	27
$\boldsymbol{A}_{\mathbf{CF}}$	Coefficient for $CF_{ti,Sep}$ function	0.25	0.25	0.15
$\boldsymbol{a}_{\mathbf{CF}}$	$CF_{ti,Sep}$ exponent $[Ca^{2+}]_{SR}$ reference parameter	1.192	1.124	0.99
$\boldsymbol{k}_{\mathbf{CF}}$	$CF_{ti,Sep}$ exponent $[Ca^{2+}]_{SR}$ gradient parameter	0.0311	0.0481	0.0111
$\boldsymbol{A}_{\mathbf{kF1}}$	Coefficient for k_{F1} function	8.619	3.955	10.93
$\boldsymbol{a}_{\mathbf{kF1}}$	k_{F1} exponent $[Ca^{2+}]_{SR}$ reference parameter	1.136	1.1	1.04
$\boldsymbol{k}_{\mathbf{kF1}}$	k_{F1} exponent $[Ca^{2+}]_{SR}$ gradient parameter	0.0449	0.0704	0.0659
$\boldsymbol{k}_{\mathbf{F1}}^{\mathbf{min}}$	Minimal value for k_{F1}	2.967	1.532	1.529
$\boldsymbol{A}_{\mathbf{kF2}}$	1st coefficient for k_{F2} function	583.6	173.361	181.12
$\boldsymbol{B}_{\mathbf{kF2}}$	2nd coefficient for k_{F2} function	295149	338.742	0
$\boldsymbol{H1}_{\mathbf{kF2}}$	$[Ca^{2+}]_{SR}$ power for 1st term k_{F2} function	-18.979	-21.099	-31.02
$\boldsymbol{H2}_{\mathbf{kF2}}$	$[Ca^{2+}]_{SR}$ power for 2nd term k_{F2} function	-68.306	-118.771	N/A
$\boldsymbol{k}_{\mathbf{F2}}^{\mathbf{min}}$	Minimal value for k_{F2}	5	3.9	6.7
$\boldsymbol{A}_{\mathbf{MD}}$	Coefficient of median duration (MD) function	208.57	801.86	136.86
$\boldsymbol{a}_{\mathbf{MD}}$	*MD* exponent $[Ca^{2+}]_{SR}$ reference parameter	1.136	.953	1.0312
$\boldsymbol{k}_{\mathbf{MD}}$	*MD* exponent $[Ca^{2+}]_{SR}$ gradient parameter	0.0526	0.063	0.0854
$\boldsymbol{MD}^{\mathbf{min}}$	Minimal value of MD	90	90	64.12
$\boldsymbol{A}_{\mathbf{DW1}}$	Coefficient for DW_1 function	276.72	273.53	155.61
$\boldsymbol{a}_{\mathbf{DW1}}$	DW_1 exponent MD reference parameter	324.11	268.012	223.98
$\boldsymbol{k}_{\mathbf{DW1}}$	DW_1 exponent MD gradient parameter	70.67	40.24	57.27
$\boldsymbol{DW_1}^{min}$	Minimal value of DW_1	40	48.26	40
$\boldsymbol{A}_{\mathbf{DW2}}$	Coefficient for DW_2 function	233.26	369.44	109.18
$\boldsymbol{a}_{\mathbf{DW2}}$	DW_2 exponent MD reference parameter	160.74	188.14	140.46
$\boldsymbol{k}_{\mathbf{DW2}}$	DW_2 exponent MD gradient parameter	14.6	18.72	10.7
$\boldsymbol{DW_2}^{min}$	Minimal value of DW_2	53	60.56	70.422

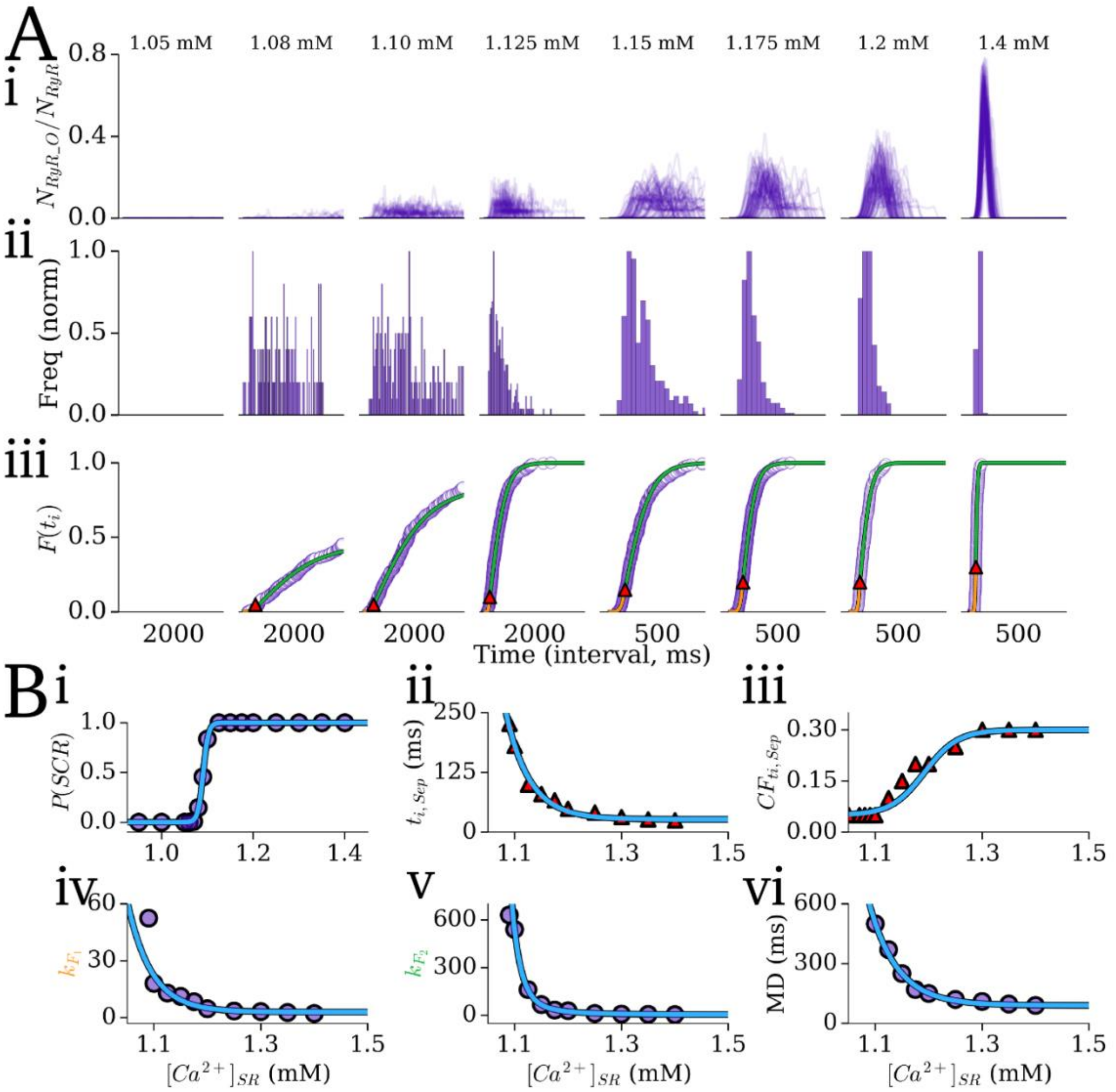

Figure S2: Derivation of the Dynamic SRF parameters in the control model. A – RyR waveforms from 100 simulations of the Ca^{2+} clamp protocol (i) over eight steps of SR-Ca^{2+} (panel titles), and corresponding initiation time distributions (ii) and cumulative frequency plots (iii) from 250 simulations at each SR-Ca^{2+}. The values given on the *x-axis* refer to the total time interval of the plot, not absolute values. The two fitting functions ($F_1(t_i)$ and $F_2(t_i)$, orange and green; see Figure 4) and their separation point (red triangular marker) are shown. B – Summary data (purple dots, red triangles) and the fit from the relevant functions (blue line) against SR-Ca^{2+} for: (i) probability of whole-cell SCRE; (ii) initiation time corresponding to the separation point, $t_{i,Sep}$; (iii) the normalised cumulative frequency at this point, $CF_{ti\_Sep}= F(t_i)\,|_{ti=ti\_Sep}$; (iv) the k parameter for $F_1(t_i)$ and (v) for $F_2(t_i)$; (vi) the median duration, *MD*.

The General Dynamic SRF model

Primary equations for the parameters are:

$$CaSR_{\min} = CaSR_{threshold} - 0.5CaSR_{P\_range} \quad (127)$$

$$P(SCR) = \left[1+\exp\left(-([Ca^{2+}]_{SR} - CaSR_{threshold}) / 0.1CaSR_{P\_range}\right)\right]^{-1} \quad (128)$$

$$t_{i\_Sep} = \left(t_{i,Sep}^{\max} - t_{i,Sep}^{\min}\right)e^{\left(-5\left([Ca^{2+}]_{SR} - CaSR_{\min}\right)/\left(CaSR_{\max} - CaSR_{\min}\right)\right)} + t_{i,Sep}^{\min} \quad (129)$$

$$MD = \left(MD^{\max} - MD^{\min}\right) e^{\left(-5\left([Ca^{2+}]_{SR} - CaSR_{\min}\right)/\left(CaSR_{\max} - CaSR_{\min}\right)\right)} + MD^{\min} \tag{130}$$

$$k_{F_1} = 0.145\left[\left(t_{i,width}^{\max} - t_{i,width}^{\min}\right)\left[\left(t_{t,Sep} - t_{i,Sep}^{\min}\right)/\left(t_{i,Sep}^{\max} - t_{i,Sep}^{\min}\right)\right]^{H_{width}} + t_{i,width}^{\min}\right] \tag{131}$$

$$k_{F2} = 1.5k_{F1} \tag{132}$$

$$DW_1 = \left(\lambda_{width}^{\max} - \lambda_{width}^{\min}\right)\left[\left(MD - MD^{\min}\right)/\left(MD^{\max} - MD^{\min}\right)\right]^{H_{width}} + \lambda_{width}^{\min} \tag{133}$$

$$DW_2 = DW_1 \tag{134}$$

Where the following may be defined by the user: (i) - The threshold for SCRE ($CaSR_{\text{threshold}}$); (ii) - The SR-Ca^{2+} range over which P(SCR) varies from 0 to 1 ($CaSR_{\text{P\_range}}$); (iii) - The maximal SR-Ca^{2+} above which SCRE distributions converge ($CaSR_{\max}$); (iv) - The minimum and maximum $t_{i,\text{Sep}}$ and MD ($t_{i,\text{Sep}}^{\min}$, $t_{i,\text{Sep}}^{\max}$, $MD^{\min}$, $MD^{\max}$); (v) - The t_i and λ distribution widths at these extremes ($t_{i,\text{width}}^{\min}$, $t_{i,\text{width}}^{\max}$, $\lambda_{\text{width}}^{\min}$, $\lambda_{\text{width}}^{\max}$); And (vi) - the non-linearity of width variance (H_{width}); $CF_{\text{ti,Sep}}$ was set to 0.4.

Parameter sets used in the study, for the investigation of variability on SR-Ca^{2+}-TA relationship (General 1-5) and generalisation of the approach by implementation in the Grandi et al. human atrial cell model [14] and parameterisation to the data of Workman et al. 2012 [15] are:

Table S11: SR-Ca^{2+} dependence parameters, General Dynamic model.

Parameter	General_1	2	3	4	5	Grandi	Workman
$CaSR_{\text{threshold}}$	1.1	1.05	1.00	1.15	1.2	0.6	0.72
$CaSR_{\text{P\_range}}$	0.05	0.05	0.05	0.05	0.05	0.01	0.01
$CaSR_{\max}$	1.7	1.65	1.6	1.75	1.8	0.7	1.07
$t_{i,\text{Sep}}^{\min}$	30	30	30	30	30	200	45
$t_{i,\text{Sep}}^{\max}$	870	870	870	870	870	1000	940
$MD^{\min}$	50	50	50	50	50	100	75
$MD^{\max}$	800	800	800	800	800	800	250
$t_{i,\text{width}}^{\min}$	20	20	20	20	20	150	8
$t_{i,\text{width}}^{\max}$	1000	1000	1000	1000	1000	800	262
$\lambda_{\text{width}}^{\min}$	20	20	20	20	20	50	15
$\lambda_{\text{width}}^{\max}$	300	300	300	300	300	300	150

Inverse functions

Initiation time, t_i; inverse function of equation (115):

$$t_i = \begin{cases} -k_{F_1}.\ln\left(\dfrac{2CF_{t_{i,Sep}}}{rand} - 1\right) + t_{i\_sep} & rand < CF_{t_{i,Sep}} \\ -k_{F_2}.\ln\left(\dfrac{2\left(1 - CF_{t_{i,Se[}}\right)}{rand + 1 - 2CF_{t_{i,Sep}}} - 1\right) + t_{i\_sep} & rand \geq CF_{t_{i,Sep}} \end{cases} \tag{135}$$

Duration, λ; inverse function of equation (116):

$$\lambda = \begin{cases} 0.261DW_1.\ln\left(rand^{-1} - 1\right) + MD & rand < 0.5 \\ 0.261DW_2.\ln\left(rand^{-1} - 1\right) + MD & rand \geq 0.5 \end{cases} \tag{136}$$

Peak time, t_p, following a uniform distribution approximation:

$$t_p = 25 + rand(\lambda - 52) + t_i \quad (137)$$

$N_{RyR\_O}^{peak}$, $N_{RyR\_O}^{plateau}$ are determined from λ directly from their definition functions (eqns 119-120) and $\pm rand \times$ simple variance magnitude of 0.05 and $0.25N_{RyR\_O}^{plateau}$, respectively.

Implementation with the 0D model

These SRF were implemented within the 0D cell models using a simple algorithm (Figure 6 MS): the input parameters (P(SCR), $t_{i,Sep}$, $CF_{ti,Sep}$, k_{F1}, k_{F2}, MD; see previous section: Parameter distributions) are defined at a certain time (see below) and then the waveform parameters (t_i, and λ, which in turn define t_p and $N_{RyR\_O}^{peak}$; see previous section: Parameter distributions) are randomly sampled from the associated inverse functions (equations 135-137 above). When the SRF have been initiated (i.e., $t > t_i$ and $N_{RyR\_O}^{SRF}$ is > 0), the N_{RyR_O} in the cell model is set to this value and the model thus evolves as if the equivalent SCRE was occurring in the 3D cell model.

For the simplest implementation of the SRF, the Direct Control model, this calculation is determined at the time of cellular excitation, setting t_i and λ based on the distributions defined by user input parameters (P(SCR), $t_{i,Sep}$, CF_{ti_sep}, k_{F1}, k_{F1}, MD) and five random numbers input into the corresponding inverse functions: first, a random number between 0 and 1 is generated and compared to the probability of release, *P(SCR)*; if *rand* < *P(SCR)*, then the input parameters are used to determine the inverse functions (equations 28-30) and 4 more random numbers determine the actual SRF waveform parameters; if *rand* > *P(SCR)*, then no SRF parameters are set. Note that the t_{i_sep} must be set relative to excitation time. The model will set SRF parameters based on these single distributions with every cellular excitation (note that the parameters may give an SCRE timing later than the next stimulated excitation, in which case it will be reset on the next excitation).

For the Dynamic Fit model, the calculation is performed multiple times, dynamically determined during the simulation: After an initial stimulated AP, when the RyR availability has recovered above a set threshold, the SR-Ca^{2+} is input to first define the probability of release, *P(SCR)*, from equation (22) and the same process is followed as described above, with the remaining input parameters defined from the SR-Ca^{2+} according to the appropriate functions [equations 23-27)]. If the SR-Ca^{2+} concentration changes more than by a predefined value (e.g., 0.01 mM) before the SCRE has been initiated, then the parameters are recalculated based on this new SR-Ca^{2+}. SRF parameters are not calculated during excitation (i.e., when the RyR availability is low) but recalculated upon RyR recovery.

The implementation with the 0D cell model required scaling of I_{NCX} and K_{rel} when SCRE was modelled, due to localised activity differing from proportional global activity:

$$I_{NCX\_mult} = 0.4 / \left(1 + e^{(\lambda - 176)/46}\right) + 0.25 \quad (138)$$

$$K_{rel\_mult} = 0.35 / \left(1 + e^{(\lambda - 113)/18}\right) + 0.4 \quad (139)$$

In the case that a SCRE initiates a triggered action potential, the total proportion of RyRs available to be triggered during excitation is adjusted to account for those which have already undergone release during the event:

$$N_{RyR\_O} = N_{RyR} . N_{RyR\_O\_\det} . \left(1 - 0.75 N_{RyR\_ac\_SRF}\right) \quad (140)$$

Where

$$N_{RyR\_ac\_SRF} = (t - t_i) / \lambda \quad (141)$$

References

1. Colman MA, Pinali C, Trafford AW, Zhang H, Kitmitto A. A computational model of spatio-temporal cardiac intracellular calcium handling with realistic structure and spatial flux distribution from sarcoplasmic reticulum and t-tubule reconstructions. PLOS Comput Biol. 2017 Aug 31;13(8):e1005714.

2. Hinch R. A mathematical analysis of the generation and termination of calcium sparks. Biophys J. 2004 Mar;86(3):1293–307.

3. Stern MD, Song LS, Cheng H, Sham JS, Yang HT, Boheler KR, et al. Local control models of cardiac excitation-contraction coupling. A possible role for allosteric interactions between ryanodine receptors. J Gen Physiol. 1999 Mar;113(3):469–89.

4. Restrepo JG, Weiss JN, Karma A. Calsequestrin-mediated mechanism for cellular calcium transient alternans. Biophys J. 2008 Oct;95(8):3767–89.

5. Gaur N, Rudy Y. Multiscale modeling of calcium cycling in cardiac ventricular myocyte: macroscopic consequences of microscopic dyadic function. Biophys J. 2011 Jun 22;100(12):2904–12.

6. Luo CH, Rudy Y. A dynamic model of the cardiac ventricular action potential. I. Simulations of ionic currents and concentration changes. Circ Res. 1994 Jun;74(6):1071–96.

7. Song Z, Ko CY, Nivala M, Weiss JN, Qu Z. Calcium-voltage coupling in the genesis of early and delayed afterdepolarizations in cardiac myocytes. Biophys J. 2015 Apr 21;108(8):1908–21.

8. Shiferaw Y, Watanabe MA, Garfinkel A, Weiss JN, Karma A. Model of intracellular calcium cycling in ventricular myocytes. Biophys J. 2003 Dec;85(6):3666–86.

9. Shannon TR, Wang F, Puglisi J, Weber C, Bers DM. A mathematical treatment of integrated Ca dynamics within the ventricular myocyte. Biophys J. 2004 Nov;87(5):3351–71.

10. Nivala M, de Lange E, Rovetti R, Qu Z. Computational modeling and numerical methods for spatiotemporal calcium cycling in ventricular myocytes. Front Physiol. 2012;3:114.

11. Wagner J, Keizer J. Effects of rapid buffers on Ca2+ diffusion and Ca2+ oscillations. Biophys J. 1994 Jul;67(1):447–56.

12. Rice JJ, Winslow RL, Hunter WC. Comparison of putative cooperative mechanisms in cardiac muscle: length dependence and dynamic responses. Am J Physiol. 1999 May;276(5 Pt 2):H1734-1754.

13. Gauthier LD, Greenstein JL, Winslow RL. Toward an integrative computational model of the Guinea pig cardiac myocyte. Front Physiol. 2012;3:244.

14. Grandi E, Pandit SV, Voigt N, Workman AJ, Dobrev D, Jalife J, et al. Human Atrial Action Potential and Ca2+ Model: Sinus Rhythm and Chronic Atrial Fibrillation. Circ Res. 2011 Oct 14;109(9):1055–66.

15. Workman AJ, Marshall GE, Rankin AC, Smith GL, Dempster J. Transient outward K+ current reduction prolongs action potentials and promotes afterdepolarisations: a dynamic-clamp study in human and rabbit cardiac atrial myocytes. J Physiol. 2012 Sep 1;590(17):4289–305.
