# Supplementary material for: Arrhythmia mechanisms and spontaneous calcium release: Bi-directional coupling between re-entrant and focal excitation
Source: PLoS Comput Biol. 2019 Aug 8;15(8):e1007260. doi: 10.1371/journal.pcbi.1007260 (PMC6687119; doi:10.1371/journal.pcbi.1007260)
Supplement: S2 Text — Document containing additional figures and text for the validation of the methods and additional results. (PDF) [file pcbi.1007260.s002.pdf]

Supplementary Material S2 Text: Validation and Results

Arrhythmia Mechanisms and Spontaneous Calcium Release: Bi-directional Coupling Between Re-entrant and Focal Excitation

Michael A. Colman

School of Biomedical Sciences, Faculty of Biological Sciences, University of Leeds, UK

Correspondence:

m.a.colman@leeds.ac.uk

1. Validation of approaches by Ca^{2+} clamp

Comparison of 3D and 0D cell model behaviour under Ca^{2+} clamp highlights the strong agreement in both waveform variation (Figure S1A,B), the distributions (Figure S1C-D) and their summary properties (Figure 9E). Similar agreement was also observed for both of the remodelled conditions (Figure S2).

Note that the match is not perfect. However, the largest discrepancies are observed near the threshold (where also P(SCR) < 1 and the distributions are therefore over a smaller sample size). This also corresponds to the most spread-out and lowest amplitude SCRE, which therefore are the least important regarding whole-cell behaviour; so long as the 0D approximation does not introduce larger release than observed in the 3D model, this will not propagate as erroneous results.

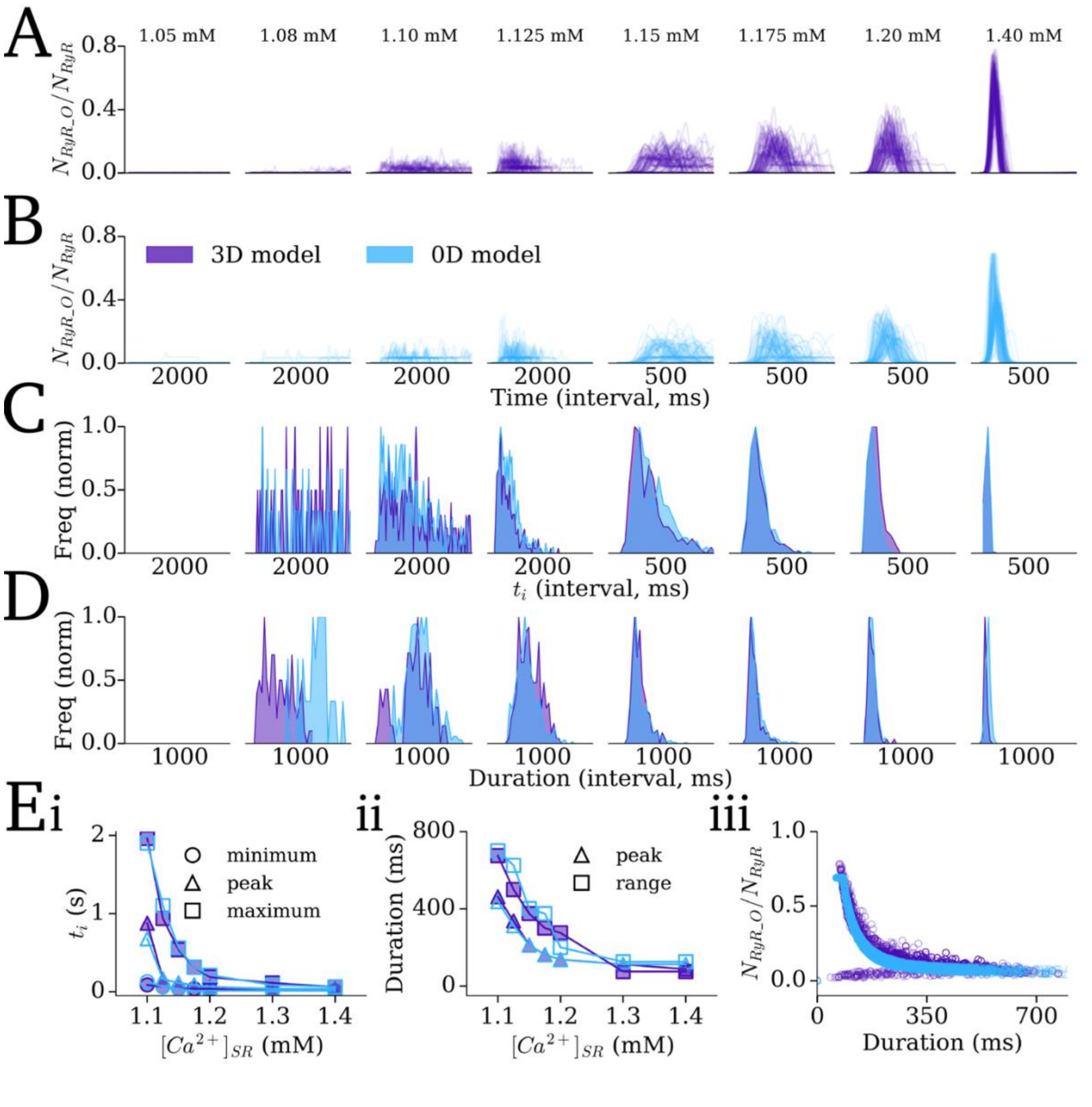

Figure S1: Validation of the control SRF through Ca^{2+} clamp. Results of 250 simulations at different SR-Ca^{2+} concentrations (labels on top of panel A) for the 3D model (purple) and 0D model with control Dynamic Fit SRF implementation (blue), showing: traces of the open RyR from 100 simulations at each SR-Ca^{2+} value (A,B); Histograms of the initiation time (C) and duration of the waveform (D); Summary of measured distribution parameters (Ei,ii), and scatter plot of peak (mode) open RyR against duration (Eiii). The values given on the *x*-axis of panels A-D refer to the total time interval of the plot, not absolute values.

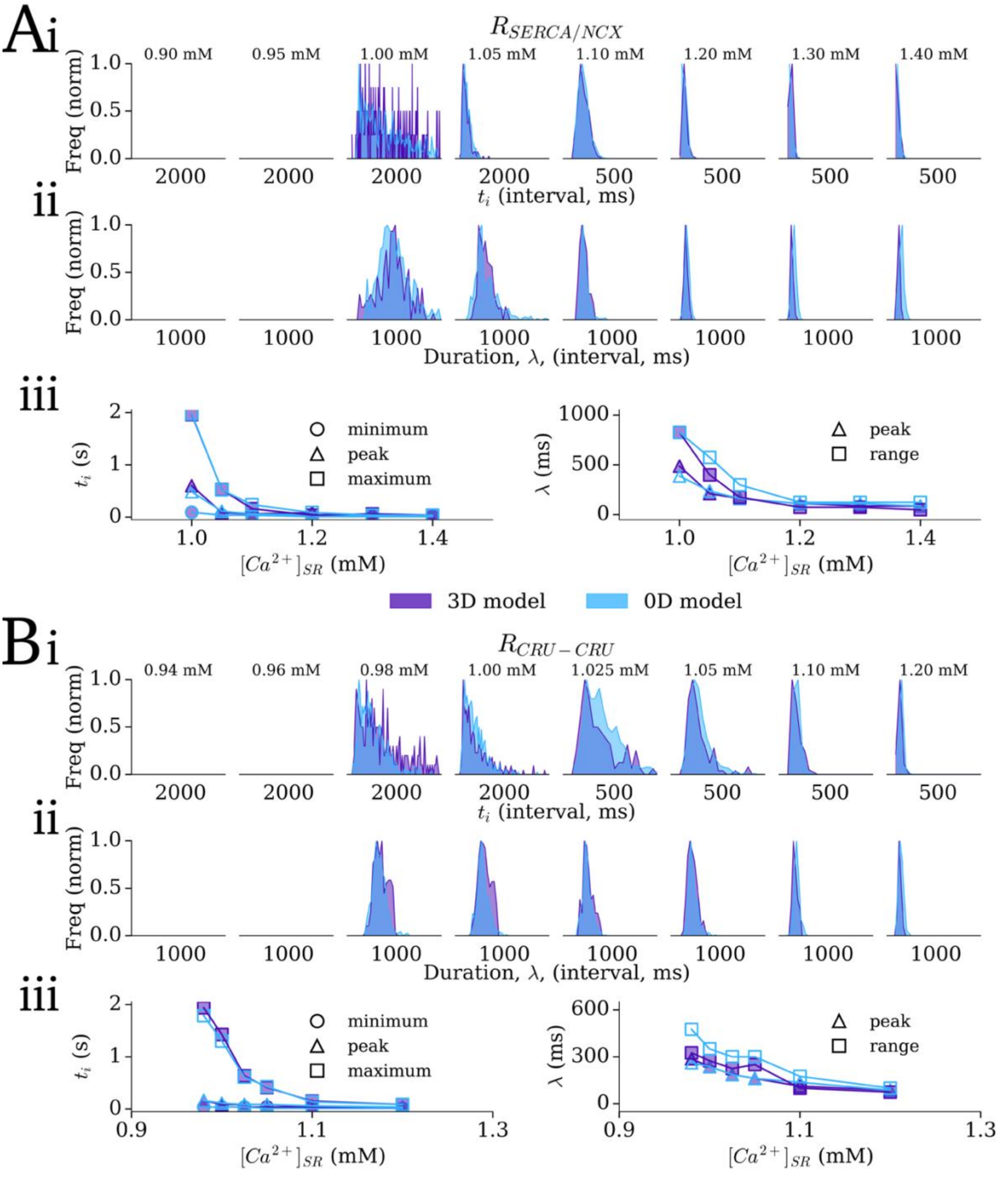

Figure S2: Validation of the remodelling SRF through Ca^{2+} clamp. Results of 250 simulations at different SR-Ca^{2+} concentrations (labels on top of panel A and B) for the 3D cell model (purple) and 0D model (blue) for the SERCA up-regulated/NCX down-regulated remodelling model ($R_{SERCA/NCX}$, A) and increased CRU-CRU coupling remodelling model ($R_{CRU\text{-}CRU}$, B). Histograms of the initiation time (i) and duration of the waveform (ii); Summary of measured distribution parameters (iii). The values given on the x-axis of panels (i-ii) refer to the total time interval of the plot, not absolute values.

Note that both remodelled conditions lower the SR-Ca^{2+} threshold for release; for the R_{SERCA_NCX} condition, this is because the reduced efflux due to lower NCX expression leads to a higher probability of a Ca^{2+} spark propagating as a Ca^{2+} wave.

2. Focal excitations in different tissue models

Focal excitations emerging from a single focus in 2D and 3D tissue models (Figure S3).

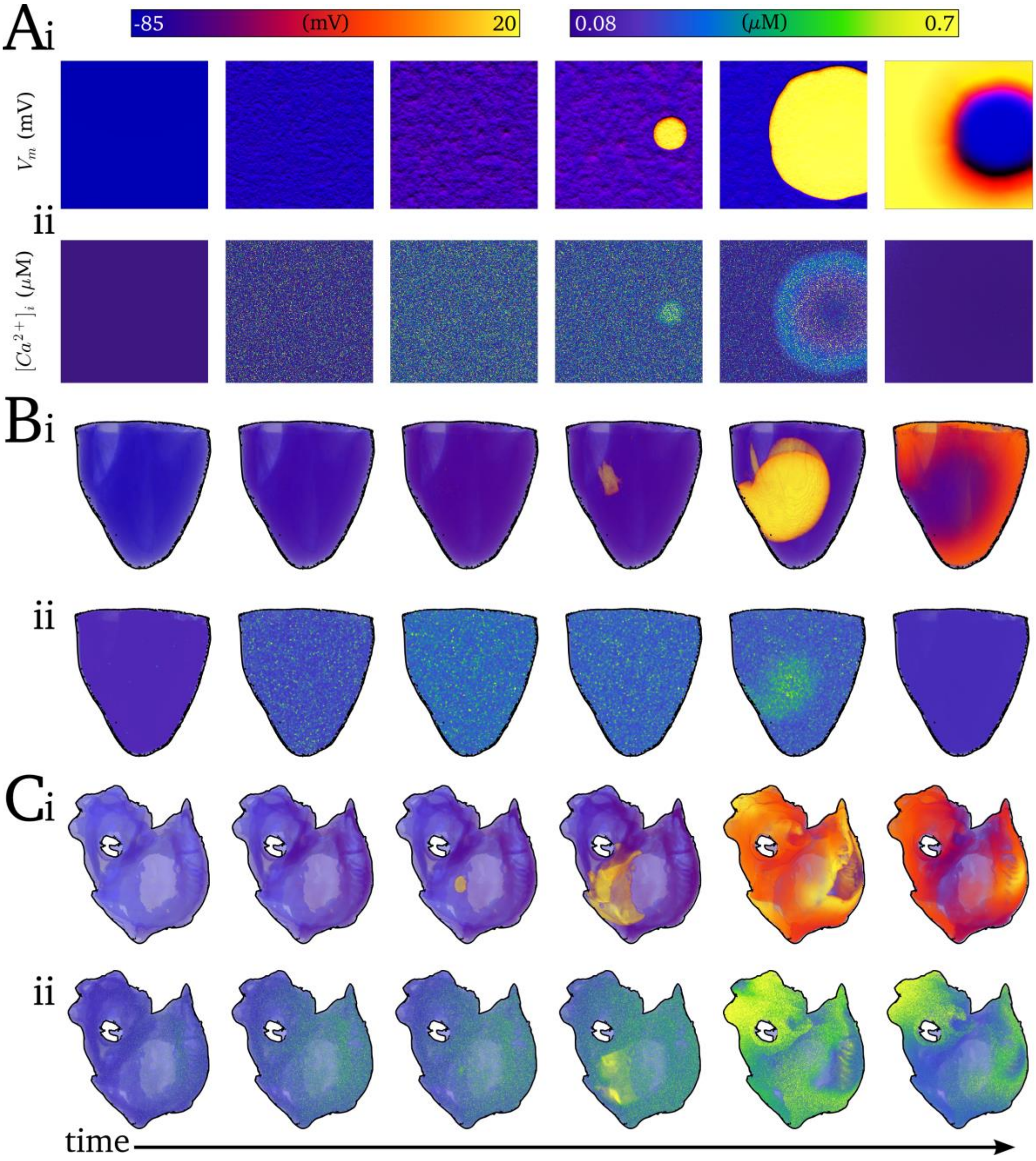

Figure S3: Development of SCRE mediated ectopic focal excitation. Snapshots of SCRE mediated focal excitation in idealised 2D sheet (A), whole-ventricle (B) and whole-atria (C) tissue models, showing the membrane potential (i, mV) and intracellular Ca^{2+} concentration (ii, μM).

3. Parameter distribution criticality for focal excitation

The Direct Control implementation was used to assess the constraints on the initiation time and duration distributions which lead to focal excitation in tissue. The important parameter for the initiation time is the width of the distribution, controlling the level of synchronisation, and it was thus described by a symmetric distribution (i.e. $CF_{ti\_sep} = 0.5$) with t_{i_Sep} set to 1000 ms and width parameter ($k_{F1} = k_{F2} = t_iW$) varied from fully synchronised (= 0 ms) to loosely synchronised (= 350 ms, giving a total spread for 98% of the population of 700 ms; Figure S4Ai). For the duration distribution, the important property is the probability of short-duration, large amplitude release, with a probability of leading to TA in single cells. This property is affected by both the median duration (*MD*) and width of the distribution ($DW_1 = DW_2 = DW$); to facilitate analysis, these were varied together with a maintained reference that the minimum duration, described by the difference between MD and DW, was set to 50 ms (i.e. MD – DW = 50). Thus, the distributions start at the highest probability of TA, where MD = 75 and DW = 25, and the DW was successively increased up to 350 ms (with the corresponding increase in MD), reducing the probability of sampling a short duration parameter (Figure S4Aii).

These distribution variations were then implemented in a 2D sheet tissue model under four conditions, corresponding to low and high SR-Ca^{2+} (~ 0.9 mM and ~1.2 mM; note that here the only effect of SR-Ca^{2+} is on the amplitude of the spontaneous Ca^{2+} transient) and control and reduced I_{K1} (a reduction in conductance of 25% and a 10 mV shift), leading to a more positive resting potential of -75 mV (vs -86 mV in control). 10 simulations were performed per condition and distribution parameter combination, and the results were grouped into three outcomes (Figure S4Bi-iv): those where no focal excitations were observed (P(focal) $\approx$ 0), those where focal excitations emerged in all 10 simulations (P(focal) $\approx$ 1), and those where focal excitations emerged in some but not all of the 10 simulations (0 < P(focal) < 1). Thus, the thresholds for focal excitation in t_i and λ parameter space can be examined (Figure S4Bi-iv). Note that this parameter space covers only the regions where focal excitations can occur – much wider distributions in t_i and distributions where fewer or none of the durations are short enough to initiate TA will commonly emerge but are not shown as they do not lead to any focal excitation.

The constraint on the duration distribution is clear: with tight synchronisation, all conditions exhibit a *DW* threshold below (i.e., shorter than) which focal excitations were observed, which then decreases (i.e., towards shorter distributions) as the t_iW increases. Further simulations were performed with smaller intervals in *DW* (10 ms) around the threshold region for each of the conditions, and the maximum *DW* for which any focal excitations emerged (corresponding to any cases where P(focal) > 0) was calculated (Figure S4Bv). The control I_{K1} condition at low SR-Ca^{2+} has the strongest constrains on both t_iW and *DW*: the shortest durations and tightest synchronisations were required for focal excitations to emerge. The effect of reduced I_{K1} (i.e., more positive RP) was more substantial than the SR-Ca^{2+} (i.e. Ca^{2+} transient amplitude), and the combination of the two led to the least constraints on the emergence of focal excitations.

Only a few conditions – even at *DW* intervals of 10 ms – led to cases where focal excitation emerged in some but not all simulations, further highlighting the threshold nature of TA in tissue – at least, in relation to high enough probabilities to be caught by O(10-100) simulations. Notable parameter combinations for which this occurred include the tightest synchronisation in the control I_{K1} at low SR-Ca^{2+} (Figure S4Bi), where the probability of focal excitation was $\approx$ 1 for t_i widths of 10 and 15, but < 1 for t_i widths of 0 and 5 ms. This highlights the role of SCRE occurring before the trigger point in raising the membrane potential sufficiently to overcome electrotonic load.

The parameter combinations which led to variable outcomes were analysed further, through a comparison of the resultant initiation time and duration distributions between simulations which did and did not lead to focal excitation. Data from one selected example (t_iW = 100 ms; *DW* = 140 ms) are shown in Figure S4C. For comparison, four additional parameter combinations were considered where either t_iW or *DW* were individually varied relative to the selected combination, either increasing the widths (parameter combination 2: t_iW remains = 100 ms, *DW* = 240 ms; parameter combination 3: t_iW = 250ms, *DW* remains

= 140ms) or decreasing them (parameter combination 4: $t_i W$ remains = 100 ms, DW = 100 ms; parameter combination 5: $t_i W$ = 25ms, DW remains = 140ms).

The effect of both $t_i W$ and DW on the emergence of focal excitation is clear: when either is significantly shortened, focal excitation emerges, and when either is widened, it does not (Figure S4Ca, c). However, the distributions associated with simulations which did and did not lead to focal excitation under the same parameter combinations did not exhibit any clear differences or indicators of the outcome (Figure S4Cb) – the parameter distributions sampled from were identical, and the measured outcomes of actual parameters had no statistically significant difference, nor were there clear differences in the correlation of t_i and λ. One possible indicating metric to predict the emergence of focal excitation is to calculate the number of cells which exhibit a duration below various thresholds (Figure S4Civ). This metric did provide a clear indication of the differences between the conditions leading to focal and no focal when the duration distribution was adjusted (Figure S4Civ,a) but did not for either the case where the initiation time distribution was adjusted (Figure S4Civ,c) or the parameter combinations were the same (Figure S4Civ,b – which now compares two simulations leading to each outcome).

Figure S4 (next page): Criticality constraints on focal excitation. A – Illustration of initiation time (i; t_i) and duration (ii; λ) distributions used to assess criticality on the emergence of focal excitation, demonstrating the effect of varying the width of t_i ($t_i W$) and duration (DW). B – Categorised outcomes over 10 simulations for no focal excitation (purple), some focal excitation (green) or all focal excitations (yellow) at various $t_i W$ and DW combinations for low SR-Ca^{2+} (i,ii) and high SR-Ca^{2+} (iii, iv) and control I_{K1} (i, iii) and reduced I_{K1} (ii, iv). (v) summarised maximum DW thresholds at each $t_i W$ over which P(focal) > 0 (v). C – Parameter-space plots (i) and λ (ii) and initiation time (iii) histograms associated with specific simulations: a) corresponds to changing DW, c) to changing $t_i W$ and b) to a single $DW/t_i W$ combination which leads to P(focal) between but not equal to 0 and 1. Note that the initiation time is now the actual time during the simulation the SRF was initiated, not the t_i parameter (which is relative to each cell's excitation time). Blue corresponds to data associated with a simulation in which no focal excitation was observed, and orange to those where focal excitation was observed. (iv) shows the number of cells within the tissue which, for each simulation, were below a set threshold (x-axis). For a and c, the data correspond to the simulations shown in i-iii; for b, the simulations in i-iii are shown as well as a second simulation associated with each outcome.

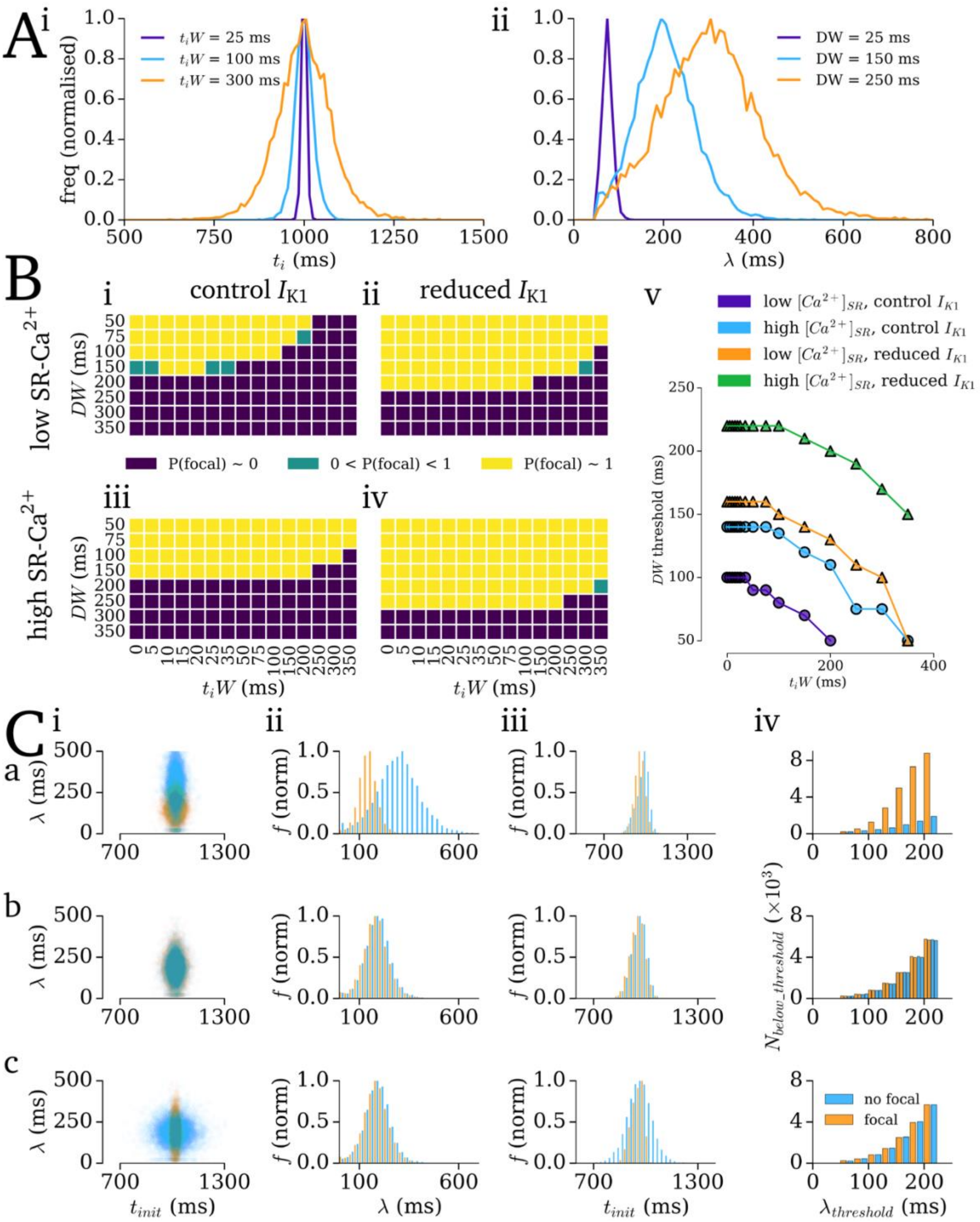

Figure S4 (legend on previous page): Criticality constraints on focal excitation.
