# Supplementary material for: Arrhythmia mechanisms and spontaneous calcium release: Bi-directional coupling between re-entrant and focal excitation
Source: PLoS Comput Biol. 2019 Aug 8;15(8):e1007260. doi: 10.1371/journal.pcbi.1007260 (PMC6687119; doi:10.1371/journal.pcbi.1007260)
Supplement: S3 Text — Document containing additional figures and text for the generalisation of approaches. (PDF) [file pcbi.1007260.s003.pdf]

Supplementary Material S3 Text: Generalisation of approaches

Arrhythmia Mechanisms and Spontaneous Calcium Release: Bi-directional Coupling Between Re-entrant and Focal Excitation

Michael A. Colman

School of Biomedical Sciences, Faculty of Biological Sciences, University of Leeds, UK

Correspondence:

m.a.colman@leeds.ac.uk

To demonstrate generalisation of the developed approaches, the SRF were first integrated with an independent cell model and its native Ca^{2+} handling system (Figure 12A). The human atrial AP model of Grandi et al., 2011 [1] was selected due to its detailed Ca^{2+} handling system containing a physiologically representative model of CICR and RyR model suitable for integration with SCRE waveforms. A General Dynamic implementation was parameterized to the SR-Ca^{2+} observed in that model in order to reproduce, for example, rate dependent susceptibility to SCRE (Figure S1A). The open RyR associated with SRF waveforms was rescaled by 0.05 to bring it in line with the model under normal CICR conditions. This demonstrates the potential suitability for direct integration with available contemporary, non-spatial AP models, without the requirement to replace the native intracellular Ca^{2+} handling system.

Furthermore, approaches to directly incorporate experimental data were demonstrated. Starting with a suitable dataset, for example as found in the analysis of rate-dependence of SCRE in the rabbit atria presented in Workman et al., 2012 [2] (Figure S1Bi-ii), SCRE occurring at each pacing rate could be directly integrated (through the Direct Control implementation) to study the potential for observed SCRE to result in focal excitation. Moreover, by relating pacing rate with SR-Ca^{2+} in the model (i.e., measuring the stable SR-Ca^{2+} concentration observed at different pacing rates in the model, and then correlating the experimental data from those pacing rates with the SR-Ca^{2+} in the model), the measured data could be plotted against model SR-Ca^{2+} and the General Dynamic implementation parameterized to fit these data (Figure S1Biii-iv). Parameterising the duration of SCRE from the data describing AP amplitude is non-trivial, partly due to the indirect experimental measure (i.e., measurement of voltage, not underlying calcium and its underlying open RyR waveform), and partly due to the role of excitation determining this amplitude. Never-the-less, the duration distribution could be approximated, utilising the threshold for TA (dotted lines, Figure S1Bii, iv). Implementation of this General Dynamic model resulted in the expected rate dependence of the timing and amplitude of SCRE (Figure S1).

1. Grandi E, Pandit SV, Voigt N, Workman AJ, Dobrev D, Jalife J, et al. Human Atrial Action Potential and Ca2+ Model: Sinus Rhythm and Chronic Atrial Fibrillation. Circ Res. 2011 Oct 14;109(9):1055–66.

2. Workman AJ, Marshall GE, Rankin AC, Smith GL, Dempster J. Transient outward K+ current reduction prolongs action potentials and promotes afterdepolarisations: a dynamic-clamp study in human and rabbit cardiac atrial myocytes. J Physiol. 2012 Sep 1;590(17):4289–305.

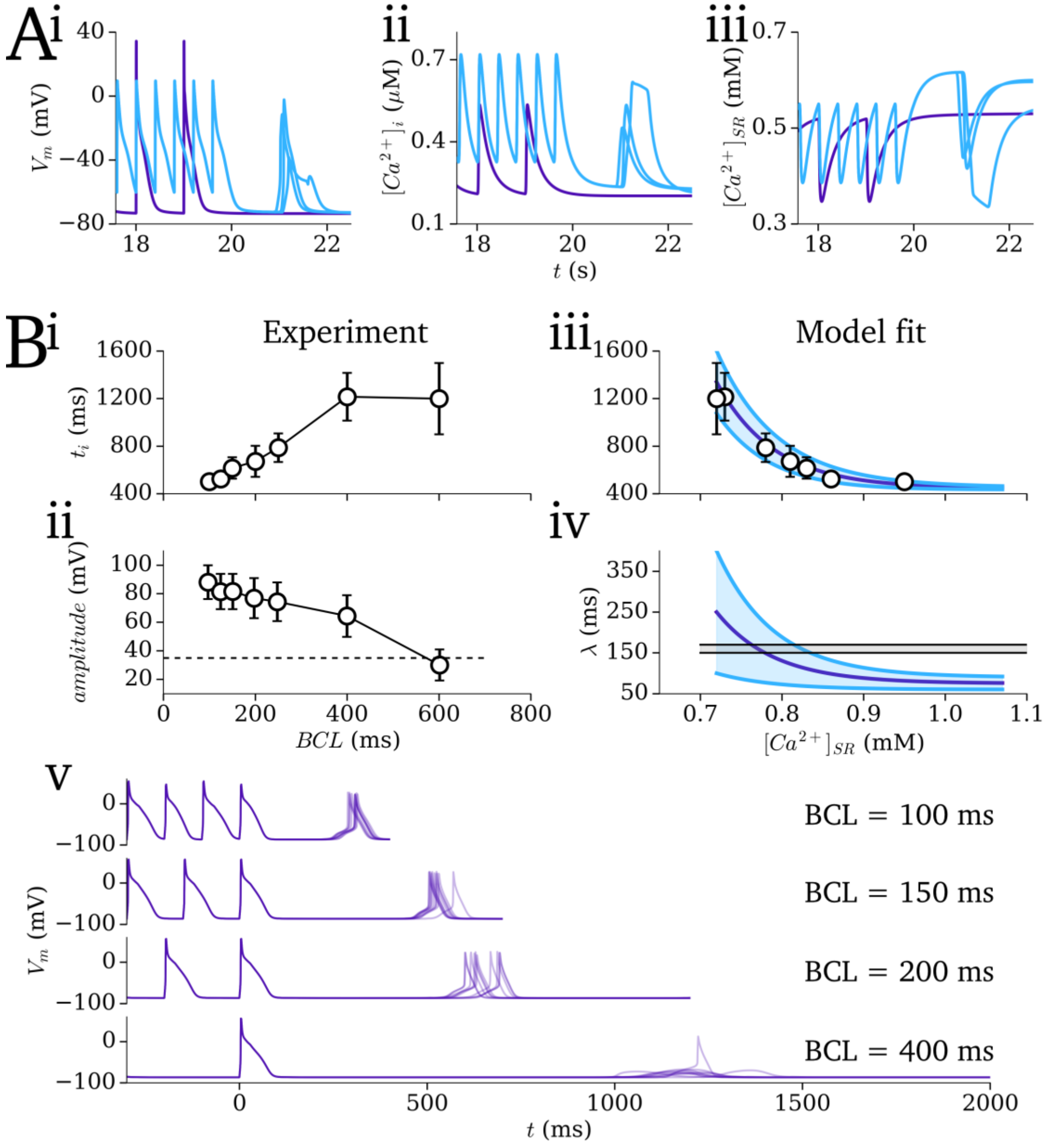

Figure S1: Generalisation of the approaches. A – Illustration of the General Dynamic SRF implementation integrated directly into the Grandi et al. 2011 human atrial model [1], showing membrane potential (i), intracellular Ca^{2+} (ii), and SR-Ca^{2+} associated with slow (BCL = 1000 ms; purple) and 3 simulations of rapid (BCL = 400ms; blue) pacing. The threshold for SCRE was set to 0.6 mM. B – Parameterising the General Dynamic SRF to experimental data of Workman et al. 2012 [2]. (i) Initiation time and (ii) amplitude of SCRE at different cycle lengths in experiment; (iii) Fitting the initiation time and variability to the experimental data plotted against model SR-Ca^2; (iv) approximating the duration of the waveform as a function of SR-Ca^{2+} utilising the durations (amplitudes) corresponding to the threshold for excitation (dotted lines). Parameter values for the General Dynamic SRF model for each case are provided in the Supplementary Material S1 Text (Model Description); (v) example overlays of multiple simulations at different BCLs implementing the derived SRF model, illustrating the difference in DAD/TA behaviour observed (closely reproducing that of the original, experimental study).
