# Supplementary material for: Arrhythmia mechanisms and spontaneous calcium release: Bi-directional coupling between re-entrant and focal excitation
Source: PLoS Comput Biol. 2019 Aug 8;15(8):e1007260. doi: 10.1371/journal.pcbi.1007260 (PMC6687119; doi:10.1371/journal.pcbi.1007260)
Supplement: S4 Text — Document containing detailed description of the limitations associated with this study. (PDF) [file pcbi.1007260.s004.pdf]

Supplementary Material S4 Text: Limitations

Arrhythmia Mechanisms and Spontaneous Calcium Release: Bi-directional Coupling Between Re-entrant and Focal Excitation

Michael A. Colman

School of Biomedical Sciences, Faculty of Biological Sciences, University of Leeds, UK

Correspondence:

m.a.colman@leeds.ac.uk

Baseline, 3D cell model

The microscopic, 3D cell model, on which the computational framework is based, has inherent limitations: It has an idealised structure in which inter-dyad distances are constant throughout the cell volume and implements a compartmentalisation CRU approach, which ignores the complex underlying structures and their heterogeneity. A functional coupling sub-space was implemented to allow the propagation of Ca^{2+} waves in order to maintain this functionality with physiological Ca^{2+} transient magnitudes, as has been used in previous studies [1,2]. This was motivated by the desire to maintain SCRE associated I_{NCX} magnitude (dependent on Ca^{2+} transient amplitude) for accurate investigation of source-sink interactions. Incorporation of direct luminal regulation or sensitisation of the RyRs to drive propagation of Ca^{2+} waves, as has been used in other studies (e.g. [3]), may provide a more physiological solution.

It should also be noted that the SR-Ca^{2+} loads in control pacing and over which spontaneous calcium release events occur do not necessarily match the limited experimental data available [4]. This is a feature of the model parameters, and whereas this does not directly impact the mechanistic analysis performed in this study, a more accurate recapitulation of SR-Ca^{2+} load dependence would be an important improvement to match the correct gain associated with SCRE in species and disease specific applications in future.

However, the primary purpose of the spatial cell model was to reproduce the dynamics, stochastic variability and features of SR-Ca^{2+} dependence of SCRE, and the model is therefore considered suitable for these ambitions. Moreover, inherent limitations in the underlying 3D cell model do not translate to the model reduction approach of using SRF, and the process presented in this study could be applied to any 3D cell model, independent of the details of its Ca^{2+} handling system, to efficiently reproduce matched SCRE.

Phenomenological approach

The phenomenological approach presented provides an approximation to the 3D spatio-temporal dynamics and therefore does not capture the full complexity of the underlying behaviour. For the dynamic models, a single environmental variable was chosen to control SCRE dynamics – the SR-Ca^{2+} concentration – which allowed definitions of the parameter distributions to be simple and intuitive. SCRE dynamics were correlated with SR-Ca^{2+} through the use of a SR-Ca^{2+} clamp protocol, which fixed intracellular Ca^{2+}. This approach therefore assumes that all conditions which directly affect SCRE in the 3D cell model will manifest in the SR-dependence. All non-Ca^{2+} concentration variables, such as the influence of changes to RyR kinetics or other Ca^{2+} handling fluxes, are therefore naturally incorporated into the ladder protocol as these variables will determine SCRE dynamics at each SR-Ca^{2+} and manifest as a different SR dependence; relation to this one variable thus captures the influence of them all.

However, the role of diastolic intracellular Ca^{2+} requires more careful consideration, as this may vary under different pacing conditions (which may be induced by changes to the Ca^{2+} handling system), has a direct influence on SCRE vulnerability and dynamics [5], and would not be captured by the ladder protocol which fixes this variable. The intracellular Ca^{2+} value used in the protocol was chosen by analysing diastolic Ca^{2+} levels under all paced conditions, which demonstrated only a small variation within the conditions considered in this study (~0.08-0.12 μM). This meant that using an intracellular concentration of 0.1 μM was a reasonable approximation for all paced conditions observed. However, this will not necessarily always be the case, as major changes to the Ca^{2+} handling system could result in conditions in which diastolic Ca^{2+} varied by significantly larger amounts, resulting in SCRE analysed at any SR-Ca^{2+} and 0.1 μM intracellular Ca^{2+} no longer being valid for the model under pacing conditions.

One solution would therefore be to derive the model based on both intracellular- and SR-Ca^{2+}. However, this led to more complex equations which resulted in poorer fits overall: the SR-dependence parameters must be fit as a function of intracellular Ca^{2+}, resulting in an additional level of approximation. Another solution would be to first correlate the SR- and intracellular-Ca^{2+} over all paced conditions, and then set the intracellular Ca^{2+} to the appropriate correlated value for each SR-Ca^{2+} concentration in the ladder protocol. This would be suggested as the best solution under conditions where it becomes necessary. A further alternative would be to derive the models as a function of SR-Ca^{2+} based on a pacing protocol rather than Ca^{2+} clamp, therefore naturally capturing any correlation between SR- and diastolic intracellular-Ca^{2+}. However, this presents further limitations in a lack of direct control over SR-Ca^{2+}, having to adjust model conditions to result in the full range of SR-Ca^{2+} sufficient to derive the model, and not providing a clear reference point from which to measure initiation time. The strong match of the 3D and 0D cell models under all pacing conditions which led to SCRE supports the validity of using the single variable, and Ca^{2+} clamp protocol, to derive the SRF dynamics.

Similar considerations apply to the use of RyR recovery and ΔCaSR thresholds for the dynamic calculation of the parameters, which are coarse approximations to the true underlying dynamical system. However, these thresholds were determined empirically to give the most robust and accurate match between 3D and 0D cell models, and, again, the agreement between models under the variety of pacing conditions supports these choices.

References

1. Gaur N, Rudy Y. Multiscale modeling of calcium cycling in cardiac ventricular myocyte: macroscopic consequences of microscopic dyadic function. Biophys J. 2011 Jun 22;100(12):2904–12.

2. Voigt N, Heijman J, Wang Q, Chiang DY, Li N, Karck M, et al. Cellular and molecular mechanisms of atrial arrhythmogenesis in patients with paroxysmal atrial fibrillation. Circulation. 2014 Jan 14;129(2):145–56.

3. Wescott AP, Jafri MS, Lederer WJ, Williams GSB. Ryanodine receptor sensitivity governs the stability and synchrony of local calcium release during cardiac excitation-contraction coupling. J Mol Cell Cardiol. 2016 Mar;92:82–92.

4. Wasserstrom JA, Shiferaw Y, Chen W, Ramakrishna S, Patel H, Kelly JE, et al. Variability in timing of spontaneous calcium release in the intact rat heart is determined by the time course of sarcoplasmic reticulum calcium load. Circ Res. 2010 Oct 29;107(9):1117–26.

5. Egdell RM, De Souza AI, Macleod KT. Relative importance of SR load and cytoplasmic calcium concentration in the genesis of aftercontractions in cardiac myocytes. Cardiovasc Res. 2000 Sep;47(4):769–77.
