# Supplementary material for: Arrhythmia mechanisms and spontaneous calcium release: Bi-directional coupling between re-entrant and focal excitation
Source: PLoS Comput Biol. 2019 Aug 8;15(8):e1007260. doi: 10.1371/journal.pcbi.1007260 (PMC6687119; doi:10.1371/journal.pcbi.1007260)
Supplement: S1 Code — (TGZ) [file pcbi.1007260.s005.tgz › MSCSF/Full_documentation_distribution.pdf]

Version: V1.0 (Release)

Documentation for C/C++ code comprising the Multi-scale Cardiac Simulation Framework (MSCSF)

By Michael A. Colman

This code is associated with multiple publications and contains all implementations therein.

Please visit

https://github.com/michaelcolman/

and

https://physicsoftheheart.com/

for other models, updates, corrections etc.

Read the disclaimer on the next pages carefully before use of this code.

Source code and documentation by Michael A. Colman, 2016-2019.

For major queries, notification of errors etc, please contact:

m.a.colman@leeds.ac.uk

DISCLAIMER

COPYRIGHT MICHAEL A. COLMAN 2016-2019.

THIS PROGRAM IS FREE SOFTWARE: YOU CAN REDISTRIBUTE IT AND/OR MODIFY IT UNDER THE TERMS OF THE GNU GENERAL PUBLIC LICENSE AS PUBLISHED BY THE FREE SOFTWARE FOUNDATION, EITHER VERSION 3 OF THE LICENSE, OR (AT YOUR OPTION) ANY LATER VERSION. THIS PROGRAM IS DISTRIBUTED IN THE HOPE THAT IT WILL BE USEFUL, BUT WITHOUT ANY WARRANTY; WITHOUT EVEN THE IMPLIED WARRANTY OF MERCHANTABILITY OR FITNESS FOR A PARTICULAR PURPOSE. SEE THE GNU GENERAL PUBLIC LICENSE FOR MORE DETAILS. YOU SHOULD HAVE RECEIVED A COPY OF THE GNU GENERAL PUBLIC LICENSE ALONG WITH THIS PROGRAM. IF NOT, SEE https://www.gnu.org/licenses/

THIS SOFTWARE IS PROVIDED OPEN SOURCE AND MAY BE FREELY USED, DISTRIBUTED AND UPDATED, PROVIDED:

(i) THE APPROPRIATE WORK(S) IS(ARE) CITED. THIS PERTAINS TO THE CITATION OF COLMAN 2019 PLOS COMP BIOL (FOR THIS IMPLEMTATION) AND ALL WORKS ASSOCIATED WITH THE SPECIFIC MODELS AND COMPONENTS USED IN PARTICULAR SIMULATIONS. IT IS THE USER'S RESPONSIBILITY TO ENSURE ALL RELEVANT WORKS ARE CITED. PLEASE BELOW AND ON-SCREEN DISCLAIMER OUTPUTS FOR A GUIDE.

(ii) ALL OF THIS TEXT IS RETAINED WITHIN OR ASSOCIATED WITH THE SOURCE CODE AND/OR BINARY FORM OF THE SOFTWARE.

ANY INTENDED COMMERCIAL USE OF THIS SOFTWARE MUST BE BY EXPRESS PERMISSION OF MICHAEL A COLMAN ONLY. IN NO EVENT ARE THE COPYRIGHT HOLDERS LIABLE FOR ANY DIRECT, INDIRECT INCIDENTAL, SPECIAL, EXEMPLARY OR CONSEQUENTIAL DAMAGES ASSOCIATED WITH USE OF THIS SOFTWARE.

THIS SOFTWARE CONTAINS IMPLEMENTATIONS OF MODELS AND COMPONENTS WHICH I (MICHAEL COLMAN) DID NOT DEVELOP. ALL OF THESE COMPONENTS HAVE BEEN CODED FROM PROVIDED SOURCE CODE OR INFORMATION IN THE PUBLICATIONS. I CLAIM NO RIGHTS OR INTELLECTUAL PROPERTY OWNERSHIP FOR THESE MODELS AND COMPONENTS, OTHER THAN THEIR SPECIFIC IMPLEMENTATION IN THIS CODE PACKAGE. FURTHER TO THE ABOVE STATEMENT, ANY INDTENDED COMMERCIAL USE OF THOSE COMPONENTS MUST BE BY EXPRESS PERMISSION OF THE ORIGINAL COPYRIGHT HOLDERS. WHERE IMPLEMENTED FROM PROVIDED CODE, ANY DISCLAIMERS PRESENT IN THE ORIGINAL CODE HAVE BEEN RETAINED IN THE RELEVANT FILE.

Any use of this baseline implementation must cite:

> Colman MA. "Arrhythmia Mechanisms and Spontaneous Calcium Release: Bi-directional coupling between re-entry and focal excitation" PLOS Comp Biol 2019.

This includes any use of the baseline source code even if all components actually used have been added by the user and were not packaged with the code.

This is referred to as the "baseline publication" for this framework throughout the documentation.

Use of other specific components must cite as appropriate and detailed on the next page.

CITATION LIST

Simplified, integrated cell models (used in Colman 2019 baseline publication):

Any use of the O'Hara et al. 2011 human ventricular cell ("**Model hVM_ORD_s**") as simplified for integration with the spatial Ca^{2+} handling system, must also cite:

> O'Hara, Thomas, László Virág, András Varró, and Yoram Rudy. 2011. "Simulation of the Undiseased Human Cardiac Ventricular Action Potential: Model Formulation and Experimental Validation." *PLOS Computational Biology* 7 (5): e1002061. https://doi.org/10.1371/journal.pcbi.1002061

Any use of the Colman et al. 2013 human atrial cell model ("**Model hAM_CAZ_s**") as simplified for integration with the spatial Ca^{2+} handling system, must cite:

> Colman, Michael A., Oleg V. Aslanidi, Sanjay Kharche, Mark R. Boyett, Clifford Garratt, Jules C. Hancox, and Henggui Zhang. 2013. "Pro-Arrhythmogenic Effects of Atrial Fibrillation-Induced Electrical Remodelling: Insights from the Three-Dimensional Virtual Human Atria." *The Journal of Physiology* 591 (17): 4249–72

Human atrial cell models:

Use of any of the Workman-lab based human atrial cell models (**"Model hAM_WL_x; Model hAM_x_mWL**") must cite:

> Colman, MA, Saxena P, Kettlewell S, and Workman AJ. 2018. "Description of the Human Atrial Action Potential Derived from a Single, Congruent Data Source: Novel Computational Models for Integrated Experimental-Numerical Study of Atrial Arrhythmia Mechanisms." *Frontiers in Physiology* 9. https://doi.org/10.3389/fphys.2018.01211.

Use of the original Courtemanche-Ramirez-Nattel 1998 model ("**Model hAM_CRN**"), modified model ("**Model hAM_CRN_mWL**") or Workman-lab model using the CRN Ca^{2+} handling system ("**Model hAM_WL_CRN**") must cite the original paper:

> Courtemanche, M., R. J. Ramirez, and S. Nattel. 1998. "Ionic Mechanisms Underlying Human Atrial Action Potential Properties: Insights from a Mathematical Model." *The American Journal of Physiology* 275 (1 Pt 2): H301-321.

Use of the original Nygren et al. 1998 model ("**Model hAM_NG**") or modified model ("**Model hAM_NG_mWL**") must cite the original paper:

> Nygren, A., C. Fiset, L. Firek, J. W. Clark, D. S. Lindblad, R. B. Clark, and W. R. Giles. 1998. "Mathematical Model of an Adult Human Atrial Cell: The Role of K+ Currents in Repolarization." *Circulation Research* 82 (1): 63–81. https://doi.org/10.1161/01.RES.82.1.63.

Use of the original Grandi et al. 2011 model ("**Model hAM_GB**"), modified model ("**Model hAM_GB_mWL**") or Workman-lab model using the GB Ca^{2+} handling system ("**Model hAM_WL_GB**") must cite the original paper [1] and the updated model [2]:

> [1] Grandi, Eleonora, Sandeep V. Pandit, Niels Voigt, Antony J. Workman, Dobromir Dobrev, Jose Jalife, and Donald M Bers. 2011. "Human Atrial Action Potential and Ca2+ Model: Sinus Rhythm and Chronic Atrial Fibrillation." *Circulation Research* 109 (9): 1055–66. https://doi.org/10.1161/CIRCRESAHA.111.253955.

> [2] Chang, Kelly C., Jason D. Bayer, and Natalia A. Trayanova. 2014. "Disrupted Calcium Release as a Mechanism for Atrial Alternans Associated with Human Atrial Fibrillation." *PLOS Computational Biology* 10 (12): e1004011. https://doi.org/10.1371/journal.pcbi.1004011.

Use of the Maleckar et al. 2009 update of the Nygren et al. 1998 model ("**Model hAM_MT**") must cite the original Nygren paper as well as:

> Maleckar, Mary M., Joseph L. Greenstein, Wayne R. Giles, and Natalia A. Trayanova. 2009. "K+ Current Changes Account for the Rate Dependence of the Action Potential in the Human Atrial Myocyte." *American Journal of Physiology. Heart and Circulatory Physiology* 297 (4): H1398-1410. https://doi.org/10.1152/ajpheart.00411.2009.

Use of the family of human atrial heterogeneity cell-types (“**Celltype RA/PM/CT/BB/RAA/LAA/LA/AVR/AS/PV**”), implemented within any human atrial cell model must cite:

Colman, Michael A., Oleg V. Aslanidi, Sanjay Kharche, Mark R. Boyett, Clifford Garratt, Jules C. Hancox, and Henggui Zhang. 2013. “Pro-Arrhythmogenic Effects of Atrial Fibrillation-Induced Electrical Remodelling: Insights from the Three-Dimensional Virtual Human Atria.” *The Journal of Physiology* 591 (17): 4249–72. https://doi.org/10.1113/jphysiol.2013.254987.

Use of AF remodelling models (“**Remodelling AF_col_1/2/3/4 [1] or AF_GB [2]**”) must cite:

[1] Colman, Michael A., Oleg V. Aslanidi, Sanjay Kharche, Mark R. Boyett, Clifford Garratt, Jules C. Hancox, and Henggui Zhang. 2013. “Pro-Arrhythmogenic Effects of Atrial Fibrillation-Induced Electrical Remodelling: Insights from the Three-Dimensional Virtual Human Atria.” *The Journal of Physiology* 591 (17): 4249–72. https://doi.org/10.1113/jphysiol.2013.254987.

[2] Grandi, Eleonora, Sandeep V. Pandit, Niels Voigt, Antony J. Workman, Dobromir Dobrev, Jose Jalife, and Donald M Bers. 2011. “Human Atrial Action Potential and Ca2+ Model: Sinus Rhythm and Chronic Atrial Fibrillation.” *Circulation Research* 109 (9): 1055–66. https://doi.org/10.1161/CIRCRESAHA.111.253955.

Use of the isoprenaline models (“**ISO [0-1] ISO_model GB [1] /Col [2]**”) must cite:

[1] Grandi, Eleonora, Sandeep V. Pandit, Niels Voigt, Antony J. Workman, Dobromir Dobrev, Jose Jalife, and Donald M Bers. 2011. “Human Atrial Action Potential and Ca2+ Model: Sinus Rhythm and Chronic Atrial Fibrillation.” *Circulation Research* 109 (9): 1055–66. https://doi.org/10.1161/CIRCRESAHA.111.253955

[2] Colman, Michael A. 2013. Mechanisms of Atrial Arrhythmias: Insights from the Development of a Biophysically Detailed Model of the Human Atria. Springer Science & Business Media.

Use of the included mutation models (“**Mutation D322H/Y155C**”) must cite:

Colman, Michael A., Haibo Ni, Bo Liang, Nicole Schmitt, and Henggui Zhang. 2017. “In Silico Assessment of Genetic Variation in KCNA5 Reveals Multiple Mechanisms of Human Atrial Arrhythmogenesis.” *PLOS Computational Biology* 13 (6): e1005587. https://doi.org/10.1371/journal.pcbi.1005587.

Canine atrial cell model:

Use of the canine atrial cell model (“**Model dAM_VA**”) must cite the original paper [1] and original model on which it is based [2]:

[1] Varela, Marta, Michael A. Colman, Jules C. Hancox, and Oleg V. Aslanidi. 2016. “Atrial Heterogeneity Generates Re-Entrant Substrate during Atrial Fibrillation and Anti-Arrhythmic Drug Action: Mechanistic Insights from Canine Atrial Models.” *PLOS Computational Biology* 12 (12): e1005245. https://doi.org/10.1371/journal.pcbi.1005245.

[2] Ramirez, R. J., S. Nattel, and M. Courtemanche. 2000. “Mathematical Analysis of Canine Atrial Action Potentials: Rate, Regional Factors, and Electrical Remodeling.” *American Journal of Physiology. Heart and Circulatory Physiology* 279 (4): H1767-1785. https://doi.org/10.1152/ajpheart.2000.279.4.H1767.

Use of the regional cell models for dog atria must also cite [1], regardless of the model used.

Anatomical tissue models and geometries:

Use of the human ventricular wedge tissue model (“**Tissue_order geo Tissue_model Human_vent_wedge**”) must cite:

Benson, Alan P., Graeme Halley, Pan Li, Wing C. Tong, and Arun V. Holden. “Virtual Cell and Tissue Dynamics of Ectopic Activation of the Ventricles.” *Chaos: An Interdisciplinary Journal of Nonlinear Science* 17, no. 1 (March 1, 2007): 015105. https://doi.org/10.1063/1.2404634.

Use of the whole canine ventricle reconstruction tissue model ("**Tissue_order geo Tissue_model Canine_vent**") must cite:

Benson, Alan P., Oleg V. Aslanidi, Henggui Zhang, and Arun V. Holden. "The Canine Virtual Ventricular Wall: A Platform for Dissecting Pharmacological Effects on Propagation and Arrhythmogenesis." *Progress in Biophysics and Molecular Biology*, Cardiovascular Physiome, 96, no. 1 (January 1, 2008): 187–208. https://doi.org/10.1016/j.pbiomolbio.2007.08.002.

BY USING THIS SOFTWARE, YOU AGREE THAT YOU WILL APPROPRIATELY AND FULLY CITE THE RELEVANT WORKS.

Contents

I - Using the software....8
Forward....8
0. Package contents....9
1. Setup and basic usage....9
2. Basic arguments....10
3. Available cell model list....12
4. Output directories and files....12
4.1. Simulation log....12
4.2. The Outputs Directory....12
4.3. Output directory contents....13
5. Full list of arguments....18
5.1. Simulation settings....18
5.2. Model and Cell conditions....20
5.3 Tissue model options....23
5.4. 3D cell model options....28
5.5. Spontaneous release functions (0D models only)....29
5.6. Direct control parameters (all implementations)....31
5.7. Settings files....32
6. Examples of various protocols....33
6.1. Basic, single-cell examples....33
6.2 Tissue model examples – idealised tissue models....36
6.3 Tissue model examples – anatomically detailed/complex tissue models....46
6.4 3D cell model examples....50
6.5 0D cell model examples – Spontaneous Release Functions....54
6.6 0D tissue model examples – Spontaneous Release Functions at the organ scale....58
II - Modifying the code....59
Forward - general philosophy of the framework....59
1. Introduction....59
2. Variable naming conventions....60
3. Variable structs and contents....60
4. Adding new variables....61
5. Adding a new celltype or modulation model....62
5.1. The modifier variables....62
5.2 Types of modulation....63
5.3. Adding your new modulation; model-specific....63
5.4. Adding your new modulation; common or global....64
5.5. Adding your new modulation; spatial tissue gradients....64

6. Adding a new tissue model 65
7. Adding a new spontaneous release function model 65
8. Adding a full new, or updated, cell model 65
9. Adding a new current 67

I - Using the software

Forward

This source-code forms the Multi-scale Cardiac Simulation Framework (MSCSF), a C/C++ implementation for the simulation of cardiac electrophysiology from the sub-cellular to the organ scales. It is packaged with multiple cell and tissue models and various protocols for simulation, and is designed to be easy to modify. It includes:

"Native" models

- Implementations of multiple published single cell models ("traditional" Hodgkin-Huxley and whole-cell concentration models).
- Tissue model (1D-3D idealised; anatomically detailed) implementations of the native single cell models.

"Integrated" models

- Implementation of 3D spatio-temporal Ca^{2+} handling single cell models associated with my publications.
- Non-spatial reduction of the 3D single cell models, including Spontaneous Release Functions for efficient and controllable simulation of spontaneous calcium release events – termed "**0D**" models.
- Tissue models (1D-3D idealised; anatomically detailed) for the 0D cell models including Spontaneous Release Functions.

The primary novel components of the framework are the stochastic intracellular calcium handling system (associated with 3D and 0D cell models only) and the Spontaneous Release Functions (associated with 0D models only). However, it is also packaged with multiple, previously published and publicly available native cell models for convenient multi-model simulations.

Functionality for multiple protocols, measurements and modulation components has been included, not all of which are associated directly with a previous publication and some of which may be validated for specific models only. Please see Disclaimer for sources of published components; **it is the user's responsibility to sufficiently check and validate any use of these components in models other than originally presented.**

I hope you may find this useful in your own research. Enjoy!

Nomenclature convention: the convention used throughout this code and documentation when referring to gating and computed variables for the currents is as such: $I_{X\_type\_specifier}$. For example, the "voltage-activation" (va) gate for I_{to} is "I_{to_va}"; the "calcium-inactivation" (ci) gate for I_{CaL} is I_{CaL_ci}; the steady state (ss) and time constant (tau) of the "voltage-inactivation" (vi) gate for I_{Kur} are: $I_{Kur\_vi\_ss}$ and $I_{Kur\_vi\_tau}$. I personally find this more convenient and with a greater level of consistency than assigning specific variables for each gate (e.g. *m*, *h* and *j* for I_{Na} variables).

0. Package contents

- CODE – source code
- MSCSF_state_and_geometry_files – state and geometry files
- Simulations – simulations directory
- Example_scripts – contains sub-directories with example scripts for different functionality.

1. Setup and basic usage

***This code is designed to be used within a linux/mac command-line environment, but has not been extensively tested on different operating systems*. It requires g++/clang++ compilers and openMP parallelisation libraries. For installation on windows, refer to the separate documentation.**

1. Build the code: Once unzipped and placed in a sensible location, navigate to the code directory ("**MSCSF/CODE**") in a terminal. Copy "**Makefile_linux"** or "**Makefile_mac**" to a file simply called "**Makefile.**" Type "make" to compile the full package. This will create the following executables:

- "**model_single_native**" – Single cell implementations for traditional cell models
- "**model_tissue_native**" – Tissue implementations using traditional cell models
- "**model_single_3D**" – 3D stochastic spatial calcium handling single cell models
- "**model_single_0D**" – Non-spatial equivalent to the 3D stochastic model
- "**model_tissue_0D**" – Tissue models using the 0D derived calcium system
- "**model_Ca_clamp_3D**" – Ca^{2+} clamp protocol for 3D single cell
- "**model_Ca_clamp_OD**" – Ca^{2+} clamp protocol for 0D single cell
- "**bin_to_vtk_tissue**" – converts binary data to plain text or vtk, tissue models
- "**bin_to_vtk_3Dcell**" – converts binary data to plain text or vtk, 3D cell models

Each of these executables can be compiled individually by typing "make " followed by the identifier – the executable name without the "model_" prefix (or the entire executable name for bin_to_vtk_X).

2. Set your state and geometry file path: The folder "**MSCSF_state_and_geometry_files**" (present in the parent **MSCSF** directory) is the location to/from which all state files will be written/read and all geometry and map files are stored. To avoid having to repeatedly copy files between directories for new projects, place this directory somewhere sensible (such as a Projects parent folder) and add the full path of the location to "**PATH.txt**". Ensure **PATH.txt** is present in every simulation directory.

3. Check everything is setup: Execute the code ("**./model_single_[X]**" in the terminal) with no additional options to run the default model and conditions. The path location, as well as output filenames, will be output to screen – check that this PATH is correct.

3a.Your simulations folder: Rather than running simulations in the same folder as the code, it is suggested to use different (project-specific) folders. Copy ***model_X*** and **PATH.txt** to the appropriate project folder.

4. Executing the code

To run the code with specific settings, pass in command-line arguments at the time of execution. Arguments are passed as argument and value, separated by spaces; any number of argument-value pairs may be passed in any order:

./model_X [ARG1] [VALUE1] [ARG2] [VALUE2]…

The full argument list for each associated implementation can be output to screen by typing any incorrect argument, e.g:

./model_X Display_args

2. Basic arguments

For all implementations:

Argument	Description	Options	Variable default	Notes
Reference	Identifier for the simulation group	string	-	Adds a specific identifier to the Outputs directory name
Results_Reference	Identifier for the specific simulation	string	-	Adds a specific identifier to the Results directories
BCL	Basic cycle length	int [ms]	1000	
Beats	Number of beats	int	10	
Model	Selects which model to run	minimal<br>hAM_WL ….	minimal	Full model list presented in full arguments section
S2	Cycle length of S2 or second pacing rate	int [ms]	0	For single or multiple stimuli applied after the regular train of pacing
Settings_file	Identify a file to read in with all options, rather than passing explicitly in command line	String – filename	N/A	See section on Settings_files for instructions

All tissue models:

Argument	Description	Value	Variable default	Notes
Tissue_order	Determines the spatial dimensional order of tissue model	1D, 2D, 3D, geo	2D	Use geo for anatomically detailed tissue models
Tissue_model	Specifier for specific tissue model and settings	string [basic, …]	basic	Reference for all specific settings
Tissue_type	Electrophysiogically homogeneous or heterogeneous	homogeneous<br>heterogeneous	homogeneous	May be automatically set by Tissue_model; in which case, argument will overwrite automatic settings
Orientation_type	Determines whether isotropic or anisotropic	isotropic<br>anisotropic	isotropic	^^
D_uniformity	Whether the magnitude of the diffusion coefficient varies in space	uniform<br>regional<br>map<br>regiona_map	uniform	regional means will vary according to celltype;<br>map means according to a read-in scaling map;<br>default automatically set by Tissue_model
Spatial_output_interval_{data/vtk}	The period for which spatial data will be output, for either binary arrays (data) or vtk files (vtk)	int [ms]	5 ms (data); 0 ms = off (vtk)	Set to 0 to prevent spatial file outputs

3D cell models:

Argument	Description	Value	Variable default	Notes
Cell_size	Identifier for full cell size	string [standard/thin…]	standard	Use to select predefined dimensions for different cells
Sim_cell_size	Dimensions of the simulated cell	full/testing	full	NOT dimensions of the cell itself, but rather, whether the whole or a proportion is being simulated, the latter for testing efficiency
tau_ss_type	Settings for sub-space diffusion time constants	fast/medium_fast/medium/medium_slow/slow	slow	
Spatial_output_interval_{data/vtk}	The period for which spatial data will be output, for either binary arrays (data) or vtk files (vtk)	int [ms]	5 ms (data); 0 ms (vtk)	Set to 0 to prevent spatial file outputs

3D and 0D cell models, including 0D tissue models and Ca-clamp

Argument	Description	Value	Variable default	Notes
Cai	Specific initial condition of intracellular Calcium	double (μM)	-	Must be specific for Ca_clamp
CaSR	Specific initial condition of SR Calcium	double (μM)		^^^^

0D and 0D tissue models; Spontaneous Release functions

Argument	Description	Value	Variable default	Notes
SRF_mode	Which SRF implementation to use	[Off/Direct_Control/Dynamic]	Off	
SRF_model	Which type of Dynamic implementation to use	3D_cell/General	3D_cell	Only valid for dynamic models; 3D_cell refers to different models derived from the 3D_cell dynamics
SRF_Pset	Specific settings for SRF	[User_control/string]	Control	Different options available depending on model used

3. Available cell model list

A list of all available cell models, and their associated source files. Full model description and options are found in the full argument list; here is just a quick reference.

Identifier	Model	Primary reference	Source file
minimal	Hybrid-minimal model	Colman 2019 PLOS Comp. Biol.	lib/Model_minimal.cpp
hAM_CRN	Human atrial cell model	Courtemanche et al. 1998 Am. J. Physiol.-Heart Circ. Physiol. 275, H301–H321	lib/Model_hAM_CRN.cpp
hAM_NG	Human atrial cell model	Nygren et al. 1998 Circ. Res. 82, 63–81	lib/Model_hAM_NG.cpp
hAM_MT	Human atrial cell model	Maleckar et al. 2009 Am. J. Physiol. Heart Circ. Physiol. 297, H1398-1410	lib/Model_hAM_MT.cpp
hAM_GB	Human atrial cell model	Grandi et al. 2011 Circ. Res. 109, 1055–1066	lib/Model_hAM_GB.cpp
hAM_WL_CRN	Human atrial cell model	Colman et al. 2018 Front. Physiol. 9 1211	lib/Model_hAM_WL.cpp
hAM_WL_GB	Human atrial cell model	Colman et al. 2018 Front. Physiol. 9 1211	lib/Model_hAM_WL.cpp
hAM_CRN_mWL	Human atrial cell model	Colman et al. 2018 Front. Physiol. 9 1211	lib/Model_hAM_WL.cpp
hAM_NG_mWL	Human atrial cell model	Colman et al. 2018 Front. Physiol. 9 1211	lib/Model_hAM_WL.cpp
hAM_GB_mWL	Human atrial cell model	Colman et al. 2018 Front. Physiol. 9 1211	lib/Model_hAM_WL.cpp
hVM_ORD_s	Human ventricular cell model	O'Hara et al. 2011 PLOS Comp. Biol. 7, e1002061	lib/Model_hVM_ORD_simple.cpp
hAM_CAZ_s	Human atrial cell model	Colman et al. 2013 J. Physiol. 591, 4249–4272	lib/Model_hAM_CAZ_simple.cpp
dAM_VA	Dog atrial cell model	Varela et al. 2016 PLOS Comp. Biol. 12, e1005245	lib/Model_dAM_VA.cpp

4. Output directories and files

Running the simulation will create all of the directories and files for output.

4.1. Simulation log

In the parent simulation directory, "**Log.dat**" contains a record of all simulations performed within the parent directory, displaying the time of execution and command-line arguments passed.

4.2. The Outputs Directory

Data associated with the simulation is written to the "**Outputs_X**" directory. The directory name will be defaulted based on the implementation used:

- model_single_native – "**Outputs_single_native**"
- model_tissue_native – "**Outputs_tissue_native**"
- model_single_3D – "**Outputs_3Dcell**"
- model_single_0D – "**Outputs_0Dcell**"
- model_tissue_0D – "**Outputs_0Dtissue**"
- model_single_Ca_clamp_3D – "**Outputs_Ca_clamp_3D**"
- model_single_Ca_clamp_0D – "**Outputs_Ca_clamp_0D**"

If a *reference* has been passed, this folder will be called "**Outputs_X_[*reference*]**".

4.3. Output directory contents

Within the *Outputs* directory are the directories "**Parameters**" and "**Results**" and the files "**Properties_log.dat**" and "**Settings.dat**". For specific tissue models, "**Conduction_velocity_log.dat**" and "**1D_conduction_success_log.dat**" will be also created.

Outputs_X_*reference*/Properties_log.dat (and similar tissue-specific equivalents) records the final beat properties, and is appended with each simulation; it therefore provides a log of the final properties of all simulations performed in **Outputs_X_*reference*** i.e. associated with implementation X and *reference*.

Outputs_X_*reference*/Results/ contains the raw data associated with the simulation (time-courses of AP, currents, concentrations, measured properties etc), in files "**Currents.dat**" and "**Properties.dat**" (and "**CRU.dat"** if using an integrated implementation). If a *results_reference* has been passed, the folder name will be appended with this reference **Outputs_X_*reference*/Results_*results_reference*.** All files within **Results** are overwritten with each simulation associated with implementation X, *reference* and *results_reference*.

Outputs_X_*reference*/Results_*results_reference*/Settings.dat contains a copy of the on-screen settings outputs for the most recent simulation associated with implementation X and *reference*/ *results_reference*.

Outputs_X_*reference*/Parameters/ contains outputs of the model parameters, voltage-dependent ion current functions, and voltage clamp data (if performed) under the exact conditions of the simulation. This directory name will also be appended with *results_reference* and is overwritten in the same way as **Results**.

Thus, use *reference* and *results_reference* to distinguish and save data accordingly; raw data should be saved with *results_reference* if you need to also track final beat properties across multiple simulations associated with *reference* (e.g. for an APDr protocol, use *reference* to label the APDr protocol, model etc, within which Properties_log.dat will contain a list of BCL and APD for all simulations performed with *reference*; save raw data at each cycle length by using *results_reference* to identify each BCL performed).

4.3.1. File contents: Settings and simulation logs

"**Settings.dat**" contains a copy of the screen outputs for settings associated with the most recent simulation passed with *reference* and *results_reference*.

"**Properties_log.dat**" contains an output of the BCL, S2 and measured properties (such as action potential amplitude, action potential duration, and calcium transient properties) associated with the final two beats of the simulation.

	**Variable**		**Variable**		**Variable**		**Variable**
1	BCL (ms)	8	P - APD_{50} (ms)	15	F - V_{min} (mV)	22	P - CaT_{min} (μM)
2	S2 (ms)	9	F - APD_{70} (ms)	16	P - V_{min} (mV)	23	F - CaT_{max} (μM)
3	F - APD_{-70mV} (ms)	10	P - APD_{70} (ms)	17	F - V_{max} (mV)	24	P - CaT_{max} (μM)
4	P - APD_{-70mV} (ms)	11	F - APD_{90} (ms)	18	P - V_{max} (mV)	25	F - $CaSR_{min}$ (mM)
5	F - APD_{30} (ms)	12	P - APD_{90} (ms)	19	F - V_{amp} (mV)	26	P - $CaSR_{min}$ (mM)
6	P - APD_{30} (ms)	13	F - dV/dt_{max} (V/s)	20	P - V_{amp} (mV)	27	F - $CaSR_{max}$ (mM)
7	F - APD_{50} (ms)	14	P - dV/dt_{max} (V/s)	21	F - CaT_{min} (μM)	28	P - $CaSR_{max}$ (mM)

(F - and P - refer to final and penultimate beats, respectively).

"**Conduction_velocity_log.dat**" contains the tissue parameters and the conduction velocities in the principal and diagonal directions; created only when **"Tissue_model conduction_velocity"** is selected.

	Variable		Variable		Variable		Variable
1	Tissue_order	6	OY	11	CV_xy_plus	16	CV_yz_minus
2	D1 (mm/ms)	7	OZ	12	CV_xy_minus	17	CV_xyz_ppp
3	D2 (mm/ms)	8	CV_x	13	CV_xz_plus	18	CV_xyz_ppm
4	dx (mm)	9	CV_y	14	CV_xz_minus	19	CV_xyz_pmp
5	OX	10	CV_z	15	CV_yz_plus	20	CV_xyz_mpp

Where all conduction velocities are in m/s, OX, OY and OZ refer to the X,Y,Z component of the orientation (this tissue model is idealised and all fibres point in the same direction), and xy_plus refers to the symmetrically identical x_plus-y_plus and x_minus-y_minus directions, xy_minus refers to the symmetrically identical x_plus-y_minus and x_minus-y_plus directions, and so on.

"**1D_conduction_success_log.dat**" contains the S2 stimulus information and whether the excitation propagated in both directions in the 1D strand; valid for any 1D tissue model with S2 argument passed. Simply outputs:

1 – S2 x location;
2 – S2 cycle length;
3 – left edge excited?;
4 – right edge excited?;
5 – excitation type.

Where:

for left and right edge excited, 0 = no and 1 = yes; excitation type = left+right = 0 for full block, = 1 for unidirectional conduction block, = 2 for full conduction.

4.3.2. File contents: Results data files

"**Outputs_X_[*reference*]/Results_[*results_reference*]/Currents.dat**"

This contains the time-courses of the membrane potential, ion currents, gating variables, and intracellular concentrations. These files are created, and thus overwritten, with each simulation associated with *reference* and *results_reference*. These files are created for all implementations (native + integrated; single cell + tissue). For tissue models, files are created for 3 specified cells in the tissue, and the name is appended "**/Currents_cell{1-3}.dat**".

	Variable		Variable		Variable		Variable
1	sim_time (ms)	13	I_{CaL_va}	25	$I_{K1\_v\_ti}$	37	Ca_jSR (mM)
2	V_m (mV)	14	I_{CaL_vi}	26	I_{NCX} (A/F)	38	Ca_nSR (mM)
3	I_{stim} (A/F)	15	$I_{CaL\_vi\_slow}$	27	I_{CaP} (A/F)	39	Ca_o (mM)
4	I_{Na} (A/F)	16	I_{Kur} (A/F)	28	I_{Nab} (A/F)	40	RyR_o
5	I_{Na_va}	17	I_{Kur_va}	29	I_{Cab} (A/F)	41	RyR_r
6	$I_{Na\_vi\_1}$	18	I_{Kur_vi}	30	I_{Kb} (A/F)	42	RyR_i
7	$I_{Na\_vi\_2}$	19	I_{Kr} (A/F)	31	I_{NaK} (A/F)	43	Nai (mM)
8	I_{to} (A/F)	20	I_{Kr_va}	32	I_{ClCa} (A/F)	44	Nai_sl (mM)
9	I_{to_va}	21	$I_{Kr\_v\_ti}$	33	I_{Clb} (A/F)	45	Nai_j (mM)
10	I_{to_vi}	22	I_{Ks} (A/F)	34	Cai (μM)	46	Ki (mM)
11	$I_{to\_vi\_s}$	23	I_{Ks_va}	35	Cai_sl (mM)	47	Nao (mM)
12	I_{CaL} (A/F)	24	I_{K1} (A/F)	36	Cai_j (mM)	48	Ko (mM)

"Outputs_X_[*reference*]/Results_[*results_reference*]/Properties.dat(Properties_Cell_{1-3}.dat)"

This contains the beat-by-beat measured properties corresponding to those which are output for the final beat in "**Properties_log.dat**". These files are created, and thus overwritten, with each simulation associated with both *reference* and *results_reference.* These files are also created for all implementations, and in tissue models the output is for the same three cells as **Currents.dat** and the filename is appended accordingly.

	Variable		Variable		Variable		Variable
1	sim_time (ms)	6	V_{min} (mV)	11	APD_{30} (ms)	16	CaT_{max} (μM)
2	V_m (mV)	7	V_{max} (mV)	12	APD_{50} (ms)	17	$CaSR_{min}$ (mM)
3	excitation_state	8	V_{amp} (mV)	13	APD_{70} (ms)	18	$CaSR_{max}$ (mM)
4	dV_m/dt (V/s)	9	APD_switch	14	APD_{90} (ms)		
5	dV_m/dt_max (V/s)	10	APD_{-70mV} (ms)	15	CaT_{min} (μM)		

<u>And for spatial/integrated cell models:</u>

"Outputs_X_[*reference*]/Results_[*results_reference*]/CRU.dat(CRU_cell_{1-3}.dat)"

This contains the time-courses and concentrations related to the **integrated** calcium handling system.

	Variable		Variable		Variable		Variable
1	sim_time (ms)	8	J_{rel} (μM/ms)	15	N_active/N_tot	22	J_{CaP_cyto} (μM/ms)
2	V_m (mV)	9	N_{RyR}_OA / N_{RyR}	16	J_{CaL} (μM/ms)	23	J_{CaP_ss} (μM/ms)
3	$[Ca^{2+}]_{ds}$ (μM)	10	N_{RyR}_OI / N_{RyR}	17	N_{LTCC}_O/N_{LTCC}	24	J_{Cab_cyto} (μM/ms)
4	$[Ca^{2+}]_{ss}$ (μM)	11	N_{RyR}_CA / N_{RyR}	18	J_{SERCA} (μM/ms)	25	J_{Cab_ss} (μM/ms)
5	$[Ca^{2+}]_{cyto}$ (μM)	12	N_{RyR}_CI / N_{RyR}	19	J_{leak} (μM/ms)		
6	$[Ca^{2+}]_{JSR}$ (μM)	13	Monomer_state	20	J_{NCX_cyto} (μM/ms)		
7	$[Ca^{2+}]_{NSR}$ (μM)	14	N_active_CRUs	21	J_{NCX_ss} (μM/ms)		

<u>And for 0D cell models with SRF turned on:</u>

"Outputs_X_[*reference*]/Results_[*results_reference*]/SRF_properties.dat"

This contains the measured properties of initiation time (actual; in simulation time) and duration of actually initiated SRF during either single-cell or tissue models. Column-data: 1 – initiation time; 2 – cell number (for tissue); 3 – peak N_{RyR_O}; 4- duration; 5 - time of peak.

<u>*4.3.3. Spatial maps and results data files (3D cell / Tissue models)*</u>

For tissue models, the geometry, fibres, diffusion coefficient and stimulus and modulation maps are all output as vtks into the **Outputs_X_[*reference*]** directory for verification, monitoring and figure making; these are overwritten for every simulation performed with implementation X and *reference* (files are only written/overwritten if relevant: geometry, fibres, D and stimulus are written every time; modulation maps only if they are applied). Files are (hopefully!) obviously named.

"Outputs_X_[*reference*]/Results_[*results_reference*]/[V]_linescan_{x,y,z}.dat"

Contains linescan data of variable *V* (Vm, Cai, CaSR) in direction *x* (x,y,z). I.e. one line per ms, with the line scanning through the tissue/cell in the x/y/z-direction. (x-direction only for tissue models; all for 3D cell models).

"Outputs_X_[*reference*]/Spatial_Results_[*results_reference*]/[V]_output_[time]{.bin/.vtk}"

Contains full output of spatial data for variable *V* (Vm, Cai, CaSR) at a specific time. VTK data is in 3D ("-100" representing empty space); binary data contains a 1D array of actual data only (no empty space).

Convert binary data to plain text and/or vtk data using the provided **bin_to_vtk_tissue/3D_cell** tools (see below sub-section).

"Outputs_X_[*reference*]/Spatial_Results_[*results_reference*]/Activation_output{.vtk/.dat}"

Contains the activation time of the final excitation (tissue models and voltage only).

Converting binary spatial data to plain text and/or vtk

If running these models, with default settings, the simulation will output only binary data for the spatial outputs (vtk files can also be output directly by the model - see arguments section.)

Use the provided tools to convert the binary data to plain text or vtk data files:

From within the same directory where the simulation was performed, or in a parent directory containing the **Outputs** directory you want to analyse, run: "./bin_to_vtk_{tissue/3Dcell}" with arguments identifying the simulation and conversion settings:

- Any **Reference** and **Results_Reference** passed (so it knows which directory to look in to read binary and write data/vtk files)
- The variable you want to convert (i.e. Vm, Ca, CaSR etc): **Variable [V]**
- The **Tissue_order** and **Tissue_model** (tissue) or **Cell_size** and **Sim_cell_size** (3Dcell) used to perform the simulation (so it knows geometry sizes and files etc).
- Which type of data to write: **Write_data [On/Off]** (plain text) and/or **Write_vtk [On/Off]**
- The time range and time interval over which to convert data: **start_time [n1] end_time [n2] interval [n3]**
- For tissue models, you need to also specify the model type (**Model_type [native/integrated]**)
- For 3Dcell models, you can also write plain text data for specified 2D slides:
  - **Write_slices [On/Off]**
  - **XZ_slice_y [x]** to define the y coordinate of the XZ slice (and similar for all directions).

Examples:

"./bin_to_vtk_tissue start_time 100 end_time 200 interval 10 Model_type integrated Variable Cai Write_vtk On Write_data On Tissue_order 2D Tissue_model basic Reference X Results_Reference Y"

"./bin_to_vtk_3Dcell start_time 100 end_time 200 interval 10 Variable CaSR Write_vtk On Write_data On Write_slices On YZ_slice_x 5 Cell_size standard Sim_cell_size full Reference X Results_Reference Y"

4.3.4. - Simulation model parameters, functions and voltage clamp data

When using single cell models (native or integrated), the model parameters and voltage-dependent functions will also be output to the **Parameters_[*results_reference*]** directory, along with voltage clamp data if the voltage clamp protocol is turned on. These files contain information on the parameters and functions exactly as they were used in the simulation, with any and all modulation incorporated.

"**Outputs_X_[*reference*]/Parameters_[*results_reference*]/Magnitude_parameters.dat**" contains a list of all the ion current densities and Ca^{2+} handling system maximal flux rates as used in implementation X, *reference* and *results_reference*. These pertain to the parameter multiplied by the scale factor.

"**Outputs_X_[*reference*]/Parameters_[*results_reference*]/Modifier_parameters.dat**" contains a list of the final value for all the modifiers (scale factors, voltage shifts etc), after sequential application of all heterogeneity and modulation.

"**Outputs_X_[*reference*]/Parameters_[*results_reference*]/I[x].dat**" contains the voltage-dependent current variables (transition rates, steady-states, time constants etc) as a function of voltage. This includes voltage shifts and time-constant scaling.

File	1	2	3	4	5	6	7	8	9	10	11	12	13
INa.dat	Vm	va_α	**va_β**	va_∞	va_τ	vi_1_ α	vi_1_β	vi_1_∞	vi_1_τ	vi_2_ α	vi_2_β	vi_2_∞	vi_2_τ
INaL.dat	Vm	va_α	**va_β**	va_∞	va_τ	vi_ α	vi_β	vi_∞	vi_τ	-	-	-	-
Ito.dat	Vm	va_∞	va_τ	vi_∞	vi_τ	vi_3_∞	vi_3_τ	vi_Fs	-	-	-	-	-
ICaL.dat	Vm	va_∞	va_τ	vi_∞	vi_τ	vi_s_τ	-	-	-	-	-	-	-
IKur.dat	Vm	va_∞	va_τ	vi_∞	vi_τ	g_{Kur}	-	-	-	-	-	-	-
IKr.dat	Vm	va_∞	va_τ	v_ti	-	-	-	-	-	-	-	-	-
IKs.dat	Vm	va_∞	va_τ	va_2_τ	-	-	-	-	-	-	-	-	-
IKACh.dat	Vm	va_∞	va_τ	v_ti	-	-	-	-	-	-	-	-	-

Voltage-clamp data files

And if Voltage clamp is turned on, a simple Voltage clamp protocol will be applied, and the following files created:

"**Outputs_X_[*reference*]/Parameters_[*results_reference*]/ICaL_IV.dat**" contains the peak current of I_{CaL} (A/F) at each voltage step with column 1 = V_m and 2 = I_{CaL} peak

"**Outputs_X_[*reference*]/Parameters_[*results_reference*]/IK_IV.dat**" contains the peak current of I_{to}, I_{Kur}, I_{Kr} and I_{Ks} (A/F) at each voltage step with column 1 = V_m , 2 = I_{to} peak, 3 = I_{Kur}, 4 = I_{Kr}, 5 = I_{Ks}.

"**Outputs_X_[*reference*]/Parameters_[*results_reference*]/IK1_IV.dat**" contains the value of I_{K1} at each voltage in range -150 mV to 100 mV.

"**Outputs_X_[*reference*]/Parameters_[*results_reference*]/ICaL_Vclamp_trace{s/_Y}.dat**" contains the time courses of the voltage and current for all steps ("..traces.dat") or just step [Y]. 1 = time; 2 = V_m; 3 = I_{CaL} (A/F); 4 = Cai (mM).

"**Outputs_X_[*reference*]/Parameters_[*results_reference*]/IK_Vclamp_trace{s/_Y}.dat**" contains the time courses of the voltage and current for all steps ("..traces.dat") or just step [Y]. 1 = time; 2 = V_m; 3 = I_{to}; 4 = I_{Kur}; 5 = I_{to}+I_{Kur} ; 6 = I_{Kr}; 7 = I_{Ks} (A/F).

4.3.5. Spontaneous release function parameters

When using 0D single cell models with the SRF turned on, the SRF model distributions will also be output to **Parameters_[*results_reference*]/SRF_distributions**.

"**Parameters_[*results_reference*]/SRF_distributions/ti_distribution.dat**" – histogram of ti

"**Parameters_[*results_reference*]/SRF_distributions/duration_distribution.dat**" – histogram of duration

"**Parameters_[*results_reference*]/SRF_distributions/NRyRo_peak_distribution.dat**" – histogram of NRyRo (1 v 2)

If a dynamic mode is selected, these above files will be appended with various CaSR concentrations, and contain the distribution at that CaSR. The additional file "**../CaSR_dependence.dat**" will also be created which tracks how the SRF parameters vary with CaSR (1 – CaSR; 2 – PSCRE; 3 – ti_sep; 4 – ti_width_1; 5 – ti_width_2; 6 – MD; 7 – duration_width_1; 8 – duration width 2.

5. Full list of arguments

Below are listed the full range of arguments (and specific options) which may be used to control and vary the models without having to adjust and re-compile the code. An error message will be produced if an invalid argument has been passed; most options/values also have error checking for validity. However, invalid values may not give an error in all cases. Because this error message prints all valid argument options to screen, you may simply type any incorrect argument (e.g. "Display Arguments") for a full argument list.

Any number of arguments may be passed; where none are passed, defaults will be used. For modulation (ISO, remodelling etc) only one modifier of each type can be passed (i.e. cannot have two remodelling models simultaneously, but can have ISO, remodelling and various direct control modifiers simultaneously).

5.1. Simulation settings

Reference [string]

- **Description:** Adds a specific identifier to the **Outputs** directory name.
- **E.g**. "./model_single_0D Reference ERP" will produce the directory **Outputs_0DCell_ERP**
- **Notes:** *[Reference]* is only appended to the directory name if argument is passed.

Results_Reference [string]

- **Description:** Adds a specific identifier to the **Results**, **Spatial_Results** and **Parameters** directory names.
- **E.g**. "./model_single_0D Reference ERP Results_Reference S2_50" will produce the directory **Outputs_0DCell_ERP/Results_S2_50.**
- **Notes:** *[Results_Reference]* is only appended to the directory names if argument is passed.

Settings_file [string]

- **Description:** Instructs the model to read in argument settings from a settings_file.
- **Notes:**
    - Must pass the full path and filename of the settings file.
    - Must pass this argument first; further arguments can be passed after this one.

BCL [int ms]

- **Description:** Basic pacing cycle length for applied stimuli (S1 stimulus).
- **Default value**: 1000 ms.

Beats [int]

- **Description:** Number of times to apply S1 stimulus.
- **Default value:** 10.

S2 [int ms]

- **Description:** Cycle length for S2 stimulus, applied after the train of S1 stimuli.

NS2 [int]

- **Description:** Number of S2 stimuli to apply; only applies if **S2** argument has been passed.
- **Notes:** Can be used for single or few short-coupled stimuli or to implement two different pacing rates.
- **Default value**: 1.

Total_time [int ms]

- **Description:** Total simulation time.
- **Default value:** The parameter of Total_time is automatically set by BCL, Beats, S2 and NS2. Passing this argument overwrites the calculated default.
- **Notes:** For **native** implementations, the default Total_time is simply the time required for the number of stimuli (including S2 if passed); for **integrated** implementations (**3D** cell and **0D** models), 2 seconds of a quiescent period is added to the end (to observe spontaneous activity). Use this argument to set something different to these.

Paced_time [int ms]

- **Description:** Time during which to apply stimuli.
- **Notes:** Use to set Total_time and Paced_time independently from each-other and Nbeats.

dt [double ms]

- **Description:** Sets the integration time-step.
- **Default value** is model dependent; this will overwrite any defaults set.

Vclamp [On/Off]

- **Valid for single cell** models only.
- **Description:** Will apply voltage clamp protocols for I_{CaL}, I_{to}, I_{Kur}, I_{Kr}, I_{Ks} and I_{K1}. Specifics of the protocols must be viewed and modified in the source code.
- **Default:** Off.

{Read/Write}_state [string]

- **Description:** Define if and how to write the model state to file, or read state files as initial conditions.
- **Options (native and 0D single cell models):** On or Off.
- **Options (3D single cell models): On -** will write/read the whole spatial state of the cell (local concentrations, RyR/LTCC states etc) and **ave -** will write/read the whole-cell averages only. Use **ave** to create and read states using different Sim_cell_size models.
- **Options (tissue models):**
    - **On** – will read/write the whole tissue state. The **Tissue_model, Tissue_type** and **Orientation_type** are also encoded into the state file name; **Celltype** is <u>not</u>.
    - **single_cell** (read only) – will read state files written by the single cell model to all of that cell-type in the tissue model. A state file for every cell-type present in the tissue model must have been written using the single cell models.
    - **ave** – will write the values of an individual coupled cell, and read these into all cells of that cell-type. For write, must be performed on homogeneous tissue for all cell-types required.
    - **phase** – for implementation of the phase-distribution method of initiating re-entry.
- **Default value**: Off

State_Reference_{read/write} [string]

- **Description:** Adds a specific identifier to the state file to be written/read.
- **Notes:** State files are automatically named using the following condition variables: Model, BCL, celltype, ISO, ACh, Remodelling, Agent, Mutation, environment (and Tissue_model, Tissue_order, Tissue_type, Orientation_type). Use state references to distinguish and identify state files which have the same values for the above variables, but differ for others (e.g. direct modulation such as INa_scale, Ito_ac_vshift, or writing the state at different time-points during a simulation.)

5.2. Model and Cell conditions

Model [string]

- **Description:** Selects which baseline cell model to use. Some models may only be appropriate for specific implementation types (native vs integrated; single cell vs tissue).
- **Default:** minimal
- **Options (General):**
  - **minimal**
    - Novel hybrid minimal model for controllable AP in the **integrated** models.
    - **Appropriate implementations**: All
- **Options (human atrial cell models):**
  - **hAM_CRN**
    - **Citation:** Courtemanche et al. 1998 Am. J. Physiol.-Heart Circ. Physiol. *275*, H301–H321.
    - **Appropriate implementations:** Native.
  - **hAM_NG**
    - **Citation:** Nygren et al. 1998 Circ. Res. *82*, 63–81.
    - **Appropriate implementations:** Native.
  - **hAM_MT**
    - **Citation:** Maleckar et al. 2009 Am. J. Physiol. Heart Circ. Physiol. *297*, H1398-1410.
    - **Appropriate implementations:** Native.
  - **hAM_GB**
    - **Citations:**
      - Grandi et al. 2011 Circ. Res. *109*, 1055–1066
      - Chang et al. 2014 PLOS Comp Biol 10 (12): e1004011.
    - **Appropriate implementations:** Native.
  - **hAM_WL_{CRN/GB} / hAM_{CRN/NG/GB}_mWL**
    - Colman-Workman human atrial cell model (hAM_WL_X) or Workman-lab modified cell models (hAM_X_mWL)
    - **Citations:**
      - Colman et al. 2018 Front. Physiol. 9 1211
      - Courtemanche et al. 1998 Am. J. Physiol.-Heart Circ. Physiol. *275*, H301–H321. (any with "CRN")
      - Nygren et al. 1998 Circ. Res. *82*, 63–81. (any with "NG")
      - Grandi et al. 2011 Circ. Res. *109*, 1055–1066. (any with "GB")
    - **Appropriate implementations:** Native
  - **hAM_CAZ_s**
    - Simplified human atrial cell model for integrated implementations.
    - **Citations:**
      - Colman et al. 2013 J. Physiol. *591*, 4249–4272.
      - Colman 2019 PLOS Comp. Biol.
    - **Appropriate implementations:** Integrated.

- **Options (human ventricular cell models):**
  - **hVM_ORD_s**
    - Simplified human ventricular cell model for integrated implementations.
    - **Citations:**
      - O'Hara et al. 2011 PLOS Comp. Biol. *7*, e1002061.
      - Colman 2019 PLOS Comp. Biol.
- **Options (canine atrial cell models):**
  - **dAM_VA**
    - **Citation:** Varela et al. 2016 PLOS Comp. Biol. *12*, e1005245
    - **Appropriate implementations:** native.

Celltype [string]

- **Description:** Specifies the cell-type/region to be used, within a baseline cell model.
- **Notes:** Each individual or group of cell model(s) can have its own set of regional cell models.
- **Default value** is model dependent.
- **Options:**
  - **minimal**:
    - EPI/M/ENDO/RA
  - All **human atrial cell** models **(hAM_X)**:
    - RA/PM/CT/RAA/AVR/BB/LA/AS/LAA/PV
    - **Specific implementation:** Colman et al. 2013 J. Physiol. *591*, 4249–4272.
  - **hVM_ORD_s**:
    - EPI/M/ENDO
  - **dAM_VA**:
    - RA/LA/PM/BB/PV
    - Specific implementation: Varela et al. 2016 PLOS Comp. Biol. *12*, e1005245.

Remodelling [string]

- **Description:** Sets a disease remodelling model to apply.
- **Default value:** "none".
- **Options:**
  - **All models**:
    - RSERCA_NCX/RCRU – "toy" remodelling models to promote spontaneous calcium release, as used to demonstrate the methods in Colman 2019 PLOS Comp. Biol.
  - For **human atrial cell** models **(hAM_X):**
    - Multiple implementations of "AF-remodelling"
    - **AF_GB** as presented in Grandi et al. 2011 Circ. Res. *109*, 1055–1066.
    - **AF_Col_{1-4}** as presented in Colman et al. 2013 J. Physiol. *591*, 4249–4272.
  - **dAM_VA**:
    - **AF_{moderate/severe}** – Varela et al. 2016 PLOS Comp. Biol. *12*, e1005245)

Remodelling_proportion [double 0-1]

- **Description:** Sets the scaling between control and full remodelling.
- **Notes:** Implementations can be linearly or non-linearly scaled. All currently packaged implementations are linear.
- **Default value**: 1.0 (full effect).

ISO [double 0-1]

- **Description:** Concentration (µM) or proportion of saturating concentration of ISO.
- **Notes:** Applies according to **ISO_model**, which will either be default or specified.

ISO_model [string]

- **Description:** Selects a specific implementation of ISO.
- **Notes:** This is only relevant where multiple different implementations of ISO exist for single or a group of cell models.
- **Options:**
    - All **human atrial cell** models (hAM_X):
        - **GB** for implementation presented in Grandi et al. 2011 Circ. Res. *109*, 1055–1066.
        - **Col** for implementation presented in Colman 2013 (New York; Springer).

ACh [double 0-1]

- **Description:** Concentration (µM) or proportion of saturating concentration of ACh.
- **Notes:** Applies according to **ACh_model**, which will either be default (for single, model-specific) or specified (for multiple models and/or implementations).

ACh_model [string]

- **Description:** Selects a specific implementation of ACh. **Currently, only an illustrative test example is presented for assistance in adding a model; <u>not to be used in research</u>.**
- **Notes:** This is only relevant where multiple different implementations of ACh exist or where multiple cell models share the same implementation.

Agent [string]

- **Description:** Pharmacological agent to apply.
- **Default value:** none.
- **Options (all models)**:
    - **MC-II-157c**: compound which affects I_{Kr}
    - **Citations**:
        - Colman et al. 2017 Front. Physiol *8*.
        - Guo et al. 2014 PLOS One *9*, e105553.
    - **Notes:** Only validated in **hVM_ORD_s** for human ventricular cells
- **Options (model-specific):**
    - Currently no further agents have been included in the framework; as with ACh, a testing example has been presented to assist in addition of your own models.

Agent_proportion [double 0-1 (scaling) or x (concentration)]

- **Description:** Scaling between control and full Agent / concentration of Agent.
- **Default value**: 1 (full effect).

Spatial_gradient [string]

- **Description:** Apply a spatial gradient model. In tissue, needs a map; in single cells, used to inspect and plot the effect of the gradient at any point.
- **Default value:** none
- **Options:**
    - **apico_basal_example, SAN_distance_example**
    - Both of these are NOT physiological or real models – simply examples of implementation

Spatial_gradient_prop [double 0-1]

- **Description:** Determines where the single cell model lies within the gradient. Single-cell implementations only (in tissue this must be defined by a spatial gradient map).

Environment [intact/isolated]

- **Description:** Sets environmental conditions to **intact** (default for previously published models) or **isolated** (change of I_{K1} formulation; removal of I_{Kr} and I_{Ks}; addition of I_{hyp}).
- **Notes:** Relevant for **human atrial** cell models only (**hAM_X**).
- **Specific citation:** Colman et al. 2018 Front. Physiol. 9.

Ihyp [double pA/pF]

- **Description:** Determines the magnitude of applied hyperpolarising current, I_{hyp}
- **Notes:** relevant only for human **atrial cell** models in **isolated** environment.

5.3 Tissue model options

Tissue_order [1D/2D/3D/geo]

- **Description:** Defines the dimensional order of the tissue model.
- **1D-3D** refer to idealised models.
- **geo** refers to those where geometry file is to be used.

Tissue_model [string]

- **Description:** Identifier for the tissue model to be used
- **Notes:** This determines tissue dimensions, conduction parameters, heterogeneity and anisotropy automatically from pre-defined settings.
- **Notes:** Specific parameter settings can be passed directly as a command-line argument (see below) which can overwrite individual default settings specified by the Tissue_model. Listed are the defaults for each tissue model.
- **Default value:** basic
- **Options (idealised tissue models; 1D-3D):**
  - **basic**
    - NX=NY=100; NZ = 10.
    - Homogeneous, isotropic, uniform D.
    - dx = 0.2 mm; D1 = 0.2, D2 = 0.05, Diso = 0.1 mm/ms.
    - Stimulus location: edge and shape: cuboid
  - **functional_model_test**
    - Testing example will all functionality included
    - Has settings for: heterogeneity, anisotropy, D non-uniformity, multiple cell models, multiple stimulus shapes, modulation maps.
  - **conduction_velocity**
    - For assessing conduction velocity.
    - 2D or 3D sheets only (NX=NY=201 for 2D; NX=NY=NZ=101 for 3D)
    - Homogeneous, anisotropic, uniform D.
    - dx = 0.25 mm; D1 = 0.2 and D2 = 0.05 mm/ms.
    - Stimulus location: centre and shape: sphere
    - Automatically calculates and outputs longitudinal, transverse and diagonal conduction velocities from the final beat of the simulation.

- **Options (idealised tissue models; 1D-3D - continued):**
  - **re-entry**
    - Settings for re-entry simulations.
    - 2D or 3D sheets only (NX=NY=200; NZ = 10)
    - homogeneous, isotropic, unform_D.
    - dx = 0.25 mm; D1 = 0.2 and D2 = 0.05 mm/ms.
    - S1 stimulus type: edge.
    - S2 stimulus type: cross_field.
  - **Vent_transmural**
    - Heterogeneous transmural strand or sheet of the ventricle.
    - Only valid for models which have ENDO, M and EPI celltypes.
    - Simple heterogeneity ratio 1:1:1.
  - **Heterogeneous_human_atria**
    - Idealised fully segmented strand/slice/3D_slab with all regions of human atria implemented
    - Valid only for human_atrial cell models (**hAM_X**).
    - Celltypes: RA, PM, CT, RAA, AVR, BB, LA, AS, LAA, PV evenly distributed.

- **Options (anatomically detailed tissue models; geo):**
  - **functional_model_test**
    - Testing example will all functionality included
    - Has settings for: heterogeneity, anisotropy, D non-uniformity (regional and map), multiple cell models, multiple stimulus shapes, modulation maps (coords and files), multi-timed stimulus
    - Available map files:
      - Stim: functional_model_test_stim.dat; functional_model_test_S2.dat; functional_model_test_S2_2.dat; functional_model_test_multi_stim.dat
      - Diffusion: functional_model_test_Dscale_base_map.dat; functional_model_test_D_AR_scale_base_map.dat
      - Modulation: functional_model_test_ISO_map.dat; functional_model_test_ACh_map.dat; functional_model_test_remod_map.dat; functional_model_test_SRF_map.dat; functional_model_test_Dscale_mod_map.dat; functional_model_test_D_AR_scale_mod_map.dat; functional_model_test_Direct_modulation_map.dat
      - Phasemap: functional_model_test_phasemap.dat
  - **Human_vent_wedge**
    - Reconstruction of a wedge of the human left ventricle.
    - **Citation:** Benson et al. 2007 Chaos 17(1):015105
    - Heterogeneous (ENDO/M/EPI), uniform_D, isotropic + anisotropic.
    - Available map files:
      - Stim: Human_vent_wedge_stim.dat

- **Canine_vent**
  - Reconstruction of the whole canine ventricle
  - Has functionality for multi-timed stimulus (default)
  - **Citation:** Benson et al. 2008 Progress in Biophysics and Molecular Biology, Cardiovascular Physiome, 96(1): 187-208
  - Heterogeneous (ENDO/M/EPI), uniform_D, isotropic + anisotropic.
  - Available map files:
    - Stim: Canine_vent_multi_stim.dat

Tissue_type [homogeneous/heterogeneous]

- **Description:** Whether the tissue is electrophysiologically homogeneous or heterogeneous.
- **Notes:** If heterogeneous is specified, the cell-types present in the tissue model must already be specified by the **Tissue_model** and must have an entry in the cell **Model** used.
- **Default value** is **Tissue_model** dependent. This will overwrite the default.

Orientation_type [isotropic/anisotropic]

- **Description:** Selects between using the isotropic or anisotropic FDM implementations.
- **Notes:** If isotropic, the parameter **Diso** (set by the **Tissue_model** or overwritten by the specific argument) defines the diffusion tensor; if anisotropic, **D1** and **D2** define the diffusion tensor.
- **Default value** is **Tissue_model** dependent.

D_uniformity [uniform/map/regional/regional_map]

- **Description:** Defines whether **Diso** or **D1 and D2** vary spatially.
- **map** will scale all **D** parameters according to a specified map; map must exist in **Tissue_model.**
- **regional** will set **D** properties according to tissue-region. Specific settings must exist in **Tissue_model**.
- **regional_map** will sequentially apply regional and map non-uniformity.
- **Default value**: uniform (majority of Tissue_models; some may have different defaults.)

Stimulus_location_type / S2_Stimulus_location_type [string]

- **Description:** Selects pre-defined settings for stimulus location and size (across all models).
- **Notes:** Individual or all settings can be manually controlled/overwritten with command-line arguments. These just define some common defaults.
- **Options (S1; idealised tissue models)**:
  - **edge** – stimulates an edge of the tissue; 10 nodes in x-direction, all in y and z.
  - **centre –** stimulates from centre of tissue; 11 nodes in all directions.
  - **whole_tissue** – stimulates the whole tissue simultaneously.
- **Options (S2; idealised tissue models):**
  - **S1** - applies the same settings as the S1 stimulus.
  - **edge/centre** – same settings as described for S1
  - **cross_field** – stimulates half the tissue (in y; all x and z).
- **Options (geo / anatomical reconstruction models):**
  - **file** - read in the stimulus site from a file (as set in tissue_model, or passed as an argument)
  - **coords -** like with idealised, set stimulus from coordinates. Requires the settings to be set in Tissue_model or passed in directly as arguments.
- **Default value:** edge (idealised models); file (geo models)

{S1/S2}_shape [cuboid/sphere]

- **Description:** Defines shape of stimulus.
- **Notes:** If using geo, this will only apply if "coords" is set for Stimulus_location_type; if "file" is selected, the map is read from a file and so shape doesn't have any effect.

{S1/S2}_{x/y/z}_loc [int]

- **Description:** Defines the centre of the stimulus in each dimension.
- **Notes:** Value is array index.

{S1/S2}_{x/y/z}_size [int]

- **Description:** Defines the size of the stimulus in each direction.
- **Notes:** Value is array index either side of centre: a size of 5 in *x* would give a total size of 11 in *x*, 5 either side of the centre-point.

Multi_stim [On/Off]

- **Description:** Sets whether to use a multi-site and timed stimulus. E.g. ventricular stimulation.
- **Notes:** Only valid for geo models; a file with the stimulus information must be supplied.

{D1/D_AR/dx} [double]

- **Description:** Explicitly sets any of these values, overwriting default settings associated with the tissue model.
- **Notes:** If the **Orientation_type** is *isotropic* then using the argument **D1** will set **Diso**, the value of D used for isotropic simulations. If *anisotropic*, D2 = **D1/D_AR.**

{OX/OY/OZ} [double 0-1]

- **Description:** Explicitly sets the global fibre orientation. Valid for idealised models only.
- **Notes:** Will automatically set the final orientation to impose normalisation; in 2D, only OX or OY needs to be defined; in 3D, any two of OX, OY and OZ must be defined.

Global_orientation_direction [X/Y/Z/{XY/XZ/YZ}_{plus/minus}/XYZ_{ppp/ppm/pmp/mpp}]

- **Description:** Automatically sets OX, OY and OZ to point in the direction specified (idealised models only).

{ISO/ACh/Remodelling/Direct_modulation}_map [Off/On]

- **Description:** Defines whether ISO, ACh, Remodelling or Direct_modulation (the direct control parameters listed later) are to be applied homogeneously to the tissue, or according to a map to localise.
- **Notes:** For anatomically detailed models, a map file must also exist and be specified.
- **Notes:** ISO, ACh and Remodelling are continuous maps, where map = 0 means no modulation applied, map = 1 means full modulation (set by the value of ISO, ACh, Remodelling_prop) applies, and between 0 and 1 is linearly scaled between none and maximum modulation. Direct_modulation is a discrete map where it can only be off or on (0 or 1).
- **Default value:** Off.

Dscale/D_AR_scale [double]

- **Description:** Scales the diffusion tensor (**D1** and **D2**) and/or the anisotropy ratio uniformly throughout tissue.
- **Notes: Dscale** applies equally to **D1** and **D2** if anisotropic (preserves anisotropy ratio). **D_AR_scale** scales **D_AR** i.e. D2 = **D1**/(**D_AR** . **D_AR_scale**).

{Dscale/D_AR_scale}_mod_map [Off/On]

- **Description:** Defines whether Dscale and/or D_AR_scale will be applied by a map or homogeneously.
- **Notes:** Similar to ISO/ACh/Remodelling maps – can be continuous, and determines region where and to what extent Dscale or D_AR_scale are applied.

map_in_type [file/coords]

- **Description:** Defines whether the modulation map (ISO/ACh/Remodelling/Direct_modulation /Dscale/D_AR_scale) will be set by coordinates (idealised or anatomically detailed) or from a file (anatomically detailed only).
- **Note:** For coordinates, all modulations must use the same map; for file, all can be different.

map_{x/y/z}_{shape/loc/size} control the modulation map in exactly the same way as the stimulus shape/loc/size arguments above. Applies for map_in_type coords only.

{stim/S2_stim/phase/Dscale_base_map/D_AR_scale_base_map/Dscale_mod_map/D_AR_scale_mod_map/ISO_map/remod_map/ACh_map/Direct_modulation_map/Spatial_gradient_map}_file [string]

- **Description:** Specifies a file to be read in for any of the above; valid for "geo" models only.
- **Note:** {Dscale/D_AR_scale}_base_map refers to the map file used for D_uniformity; {Dscale/D_AR_scale}_mod_map refers to the map file used for Dscale and D_AR_scale.
- **Notes:** This will overwrite the default file set for specific use. E.g. if a default stimulus location is for sinus rhythm, and it is desired to pace from elsewhere, can simply specify the file as an argument rather than having to make new settings.

Multiple_models [Off/On]

- **Description:** Sets whether multiple baseline cell models are to be used for different regions of the tissue model (e.g. a separate atrial and ventricular cell model).
- **Notes:** The specific tissue model must have settings for multiple models enabled.

Tissue_model_2 [string]

- **Description:** Exactly the same as Model argument, where it sets the cell model to be used in the Model_2 region of tissue model, if Multiple_models is On.

Spatial_output_interval_{data/vtk} [int (ms)]

- **Description:** The interval period for which spatial data will be output, for either binary arrays (data) or vtk files (vtk).
- **Notes:** vtk data is 3D, including any empty space (represented by "-100") ; binary data is real nodes only.
- **Notes:** set to "0" to prevent spatial outputs.
- **Default:** vtk – 0 = off; data – 5 ms.

Spatial_output_range_{start/end} [int (ms)]

- **Description:** Specify the time range over which spatial data will be output
- **Default:** Start set to 0 and end set to total_time.

5.4. 3D cell model options

Cell_size [string]

- **Description:** Defines the size of the whole cell being simulated.
- **Options:** Currently, only "standard" and "thin" have been set. Any number of cell sizes may be set in the code.
- **Default value:** standard

Sim_cell_size [full/portion/testing]

- **Description:** Sets the proportion of the cell actually being simulated.
- **Notes:** Use testing for faster simulations; portion for efficient whole-cell behaviour (but not spontaneous) and full for producing real data.

tau_ss_type [fast/medium_fast/medium/medium_slow/slow]

- **Description:** Sets the time-constant of diffusion in the sub-space to predefined.
- **Notes:** Different tau may be used for general investigation, or to represent remodelling etc. Essentially a parameter which is primarily functional and over a range of which produces validated results, where behaviour can be very sensitive to its value.
- **Default value:** slow – gives Ca^{2+} wave velocity of ~90 μm/ms.

Spatial_output_interval_{data/vtk} [int (ms)]

- **Description:** The interval period for which spatial data will be output, for either binary arrays (data) or vtk files (vtk).
- **Notes:** vtk data is 3D, including any empty space (represented by "-100") ; binary data is real nodes only.
- **Notes:** set to "0" to prevent spatial outputs.
- **Default:** vtk – 0 = off; data – 5 ms.

Delayed_CaSR_IC [Off/On]

- **Description:** Sets whether to also impose the initial condition for CaSR at a delayed time.
- **Notes: CaSR** must also be defined; Use to impose specific CaSR at a later time, if this variable needs tight control.

CaSR_IC_delay [int ms]

- **Description:** Sets the time of the delayed imposition of CaSR, if the above is passed.

{volds/RyR/LTCC}_het [Off/random]

- **Descriptions:** Sets the dyadic cleft volume and/or NRyR and/or NLTCC per dyad to either be homogeneous throughout the cell or to vary randomly around the mean for each CRU.

5.5. Spontaneous release functions (0D models only)

SRF_mode [Off/Direct_control/Dynamic]

- **Description:** Sets which SRF implementation to use.
- **Default:** Off
- **Options description:**
  - **Direct_control:** The parameters for the distributions from which specific SRF are derived are defined once. The same distributions will be used on each beat.
  - **Dynamic:** The parameters for the distributions are defined dynamically by the CaSR concentration.

SRF_model [3D_cell/General]

- **Description:** Sets which type of dynamic model to use. **Dynamic** SRF only.
- **Options description:** 3D_cell refers to models which have been derived based on the 3D cell model behaviour; General refers to general, controllable implementations.
- **Default:** 3D_cell

SRF_Pset [string]

- **Description:** Selects the specific settings for the SRF.
- **Options (Direct_control mode):**
  - **General_1** – pre-packaged setttings.
- **Options (Dynamic mode; 3D_cell model):**
  - **Control** – Parameters set to the control model behaviour.
  - **RSERCA_NCX** - Parameters set to the remodelling model RSERCA_NCX.
  - **RCRU –** Parameters set to the model where tau_ss is reduced.
- **Options (Dynamic mode; General model);**
  - **General_{1p00/1p05…1p20}** – Parameter sets used with the same settings other than the CaSR threshold for release and the CaSR max value; as used in SRF heterogeneity models.
  - **hAM_GB_native –** Parameters set to produce SRF in the native Grandi-Bers human atrial cell model.
  - **rbAM_ITO_DC –** Parameters set to reproduce the observations of the data from rabbit atrial myocytes under dynamic clamp and ISO from Workman et al.
- **Options (Direct_control and Dynamic General models):**
  - **User_control** – Sets the parameters directly from command-line arguments.
  - **Notes:** Must pass all specific parameter arguments (below).
  - **Parameter arguments (Direct_control):**
    - **SRF_DC_{**
      - **PSCRE** - probability of release (per beat).
      - **CF** - cumulative frequency of the ti distribution at the separation point
      - **ti_sep** - initiation time of the separation point (~ peak of histogram)
      - **ti_W1** – the width, in ms, of the ti distribution up to ti/CF point
      - **ti_W2** – the width, in ms, of the ti distribution above the ti/CF point
      - **MD** - the median of the duration of the waveforms
      - **duration_W }** - the width of the duration distribution, either side of the MD.

- **Parameter arguments (Dynamic General):**
  - **SRF_Dyn_{**
    - **PSCR_threshold** – the threshold for SCRE (mM)
    - **CaSR_max** – the CaSR above which behaviour converges
    - **CaSR_Prange** – the range of CaSR (mM) over which probability changes from 0 to 0.5 (threshold) / 0.5 to 1.
    - **ti_sep_max/ti_sep_min** – the value of ti_sep at CaSR_min (=CaSR_threshold-CaSR_Prange) or max.
    - **ti_width_max/ti_width_min** – the width of the distribution, left of ti_sep, at CaSR_max and min
    - **MD_max/MD_min** – the median duration at CaSR_max and min
    - **duration_width_max/duration_width_min** – width of the duration distribution at CaSR_max and min
    - **H}** – power dependency of the widths as a function of CaSR.

SRF_het [Off/string]

- **Description:** Sets whether to impose a predefined heterogeneity of SRF / SCRE dynamics.
- **Notes:** Tissue models only.
- **Options: General_1_{small/large}** to distribute 60% randomly to General_1p10 and 10% to _1p00/_1p05/1p15/1p20 (small) or 20% to all five (large).

5.6. Direct control parameters (all implementations).

Many properties of the ion current formulations and calcium dynamics can be directly modified by passing arguments. The following options are available for some or all of the following: Current scaling, voltage dependence, time-constant scaling – I_{Na}, I_{to}, I_{Kur}, I_{CaL}, I_{K1}, I_{Kr}, I_{Ks}, I_{NaL}; Scaling only – I_{NCX}, I_{CaP}, I_{Cab}, I_{Nab}, J_{up}, J_{rel}, J_{leak}. Pass argument "Display Arguments" (or any invalid argument) to receive the error message which will display the full list of the available options and the specific syntax. Modifications will be applied in addition to any other modifications set by model conditions.

NOTE: in 3D/0D models, there are two parameters which can control magnitude of I_{CaL}, Jrel: one is the expression (N per dyad ave – controlled by "**ICaL_scale**", "**Jrel_scale**") and the other is the transition rate to open state (controlled by "**LTCC_Po**", "**RyR_Po**"); these therefore have different effects, and represent different things (e.g. remodelling may change channel expression i.e. N, whereas ISO or a drug may only change channel availability).

Argument	Description	Type/default	Notes
X_scale	Scales the current or flux directly	double/1.0	
X_va_tau_scale	Scales the time constant of the voltage-dependent activation gate of current X.	double/1.0	If multiple gates exist, multiple variables may exist with specific reference.
X_vi_tau_scale	Same as above, for the voltage-dependent inactivation gate(s)	double/1.0	^^
X_shift	Shifts the voltage dependence of all voltage dependent functions associated with current X	double/0	Convention is as if being positively applied to the half-maximal constant ($V_{1/2}$) – thus is subtracted from the voltage "seen" by the current
X_{va/vi}_{ss/tau}_shift	Individually shifts the voltage-dependence of the steady-state (ss) or time-constant (tau) for the activation or inactivation gate(s)	double/0	^^
X_{va/vi}_ss_kscale	Scales the gradient parameter of regular sigmoidal steady-state voltage dependence functions	double/1.0	

5.7. Settings files

You can use settings files to store the options passed in, for quick reproduction, saving a long list of arguments etc.

These files can be anywhere, where the full path of the file must be passed in after "Settings_file" argument.

You can use a settings file AND command line arguments:

"Settings_file" argument must be passed FIRST (this is the only restriction on order of arguments)

Any arguments passed after this will supplement or overwrite the options in the file; command line args supersede settings files if the same argument is in both.

Settings files must have the following format as a plain text document, new line for each argument, spaces or tabs to separate arg and value:

Contents of "Settings_file_X.txt"

```
Number of args
ARG 1   Value 1
ARG 2   Value 2
…
```

Example: An example settings_file could have contents:

```
3
BCL 345
Model hAM_CRN
Results_Reference file_test
```

And performing:

```
./model_single_native Settings_file [filename] Remodelling AF_GB
```

will apply BCL = 345; Model = hAM_CRN; Results_Reference = file_test; Remodelling = AF_GB

Or with the same argument in the file and passed afterwards:

```
./model_single_native Settings_file [filename] Remodelling AF_GB Results_Reference remod
```

will apply BCL = 345; Model = hAM_CRN; Results_Reference = remod; Remodelling = AF_GB

6. Examples of various protocols

Here are listed examples/pseudo-scripts of how to run various models and protocols. The examples given are equivalent or similar to the script files provided in **MSCSF/Example_scripts**.

6.1. Basic, single-cell examples

1. Control pacing

Apply pacing to a specified model at a constant BCL for 200 beats, using references to identify data.

./model_single_native Model hAM_CRN BCL 750 Beats 200 Reference Control_pacing_hAM_CRN Results_Reference BCL_750

This will create an Outputs directory named: "Outputs_single_native_Control_pacing_hAM_CRN", within which results directory for the two BCLs "Results_750".

2. Including multiple modulations applied to multiple cell models

Compare control and remodelling at three ISO concentrations to two cell models.

For *model* in [hAM_CRN, hAM_GB]

For *remodelling* in [none, AF_GB]

For *iso* in [0, 0.5, 1]

./model_single_native Model *model* Beats 50 ISO *iso* Remodelling *remodelling* Reference Control_modulation_*model* Results_Reference ISO_*iso*_Remodelling_*remodelling*

This will create an Outputs directory associated with each model: "Outputs_single_native_Control_modulation_*model*" and Results directories for each condition: "Results_ISO_*iso*_Remodelling_*remodelling*"

3. APD restitution protocol

Apply multiple BCLs to a single model for control and remodelling condition

For *bcl* in sequence 200 to 1000 in steps of 50

./model_single_native Model hAM_CRN Beats 200 BCL *bcl* Reference APDr_CRN_control Results_Reference *bcl*

./model_single_native Model hAM_CRN Beats 200 BCL *bcl* Remodelling AF_Col_1 Reference APDr_CRN_remodelling Results_Reference *bcl*

This will create an Outputs directory each for control and remodelling, within properties and raw data are stored: "Outputs_single_native_APDr_CRN_{control/remodelling}" and Results directory "Results_*bcl*". "Outputs_single_native_APDr_CRN_X/Properties_log.dat" will contain a list of the final 2 beat properties for all simulations under this reference (so here, lines in sequence for each BCL from 200 to 1000 with APD, dvdt_max, amplitude etc) and "Results_*bcl*/Currents.dat" will contain the raw AP data for each pacing rate.

4. State read and write

Apply a long train of stimuli at a rapid rate, until the model reaches stable state, at which point write the state file. Then, read in the state file as initial conditions and apply just a few beats. Compare final properties for stability.

./model_single_native Model hAM_CRN BCL 450 Beats 200 Write_state On Reference State_write_read_example Results_Reference Write

./model_single_native Model hAM_CRN BCL 450 Beats 5 Read_state On Reference State_write_read_example Results_Reference Read

Note that state files are saved independently of the results data (being placed in the PATH directory), and so you need not worry about accidentally deleting state files when deleting redundant or old data.

5. ERP protocol, using state read and write

First, apply pacing at a control BCL until steady state and write the state:

./model_single_native Model hAM_CRN BCL 1000 Beats 200 Write_state On

We don't pass a reference here as we don't need to save the results data associated with the simulation – it was simply used to write the state file.

Now, read the state, apply 5 beats for S1 and then cycle through S2 intervals:

For *s2* in sequence 400 down to 80 in steps of -10

./model_single_native Model hAM_CRN BCL 1000 Beats 5 Read_state On S2 *s2* Reference CRN_ERP Results_Reference *s2*

"Outputs_single_native_CRN_ERP/Properties_log.dat" contains the measured data (relevantly Vmin, Vmax, Vamp) for each S2. Plot: column 2 (S2) vs 15 (Vmin) 17 (Vmax) or 19 (Vamp) for these properties in the final beat (i.e. S2). Can compare to columns 16, 18 and 20 for the same properties on the penultimate beat (i.e. S1 and thus should be a flat line). And/Or inspect / run a script to determine at what S2 the Vamp (column 19) reaches below e.g. 80% of that for S1 (column 20). Use the raw data in "../Results_*s2*/Currents.dat" to inspect and check the AP train and that nothing funny is happening! (e.g. alternans, missed excitations etc).

For efficiency, you probably want to run it using a coarse S2 interval at first (to identify the S2 range for the ERP) and then use a finer interval to find the actual ERP.

6. Applying a train of two different pacing rates

You can use the argument NS2 to apply multiple S2 stimuli, for example to apply a train of one pacing rate followed by another (rather than using S2 for an S1S2 protocol).

./model_single_native Model hAM_CRN BCL 1000 Beats 50 S2 500 NS2 50 Reference two_pacing_rates

Which will apply 50 beats at a cycle length of 1000 ms followed by 50 beats at a cycle length of 500 ms.

7. Multiple and direct modulation

Example where multiple modulation models are applied with and without some direct control modulation.

./model_single_native Model hAM_CRN BCL 1000 Beats 10 Celltype PV ISO 0.5 Reference Direct_modulation Results_Reference Without_direct_modulation

./model_single_native Model hAM_CRN BCL 1000 Beats 10 Celltype PV ISO 0.5 INa_scale 0.5 Jup_scale 0.5 Ito_va_tau_scale 5 ICaL_vi_ss_shift -10 IKur_va_ss_kscale 2 Ito_vi_tau_shift 10 ICaL_scale 0.5 Reference Direct_modulation Results_Reference With_direct_modulation

Further to the results data, compare the parameters list in "../Parameters_Without_direct_modulation" and "../Parameters_With_direct_modulation" and the voltage-dependent steady-state of ICaL_vi, IKur_va and time constants of Ito_va and vi in in "../Parameters_X/Ix.dat" to visualise the effect of the voltage shift, time-constant scaling and kscale:

"../Parameters…Without../ICaL.dat" 1 vs 4 against "../Parameters...With../ICaL.dat" 1 vs 4
"../Parameters…Without../IKur.dat" 1 vs 2 against "../Parameters...With../IKur.dat" 1 vs 2
"../Parameters…Without../Ito.dat" 1 vs 3, 5 against "../Parameters...With../Ito.dat" 1 vs 3, 5

8. Using state references to identify state files

State files are automatically named for a number of parameters. For single cell state files, these are: **Model**, **BCL**, **Celltype**, **ISO**, **ACh**, **Remodelling**, **Agent**, **Mutation** and **Environment**. This means that any other conditions/settings (ISO_model, Remodelling_prop, any direct control modulation) are not encoded automatically - state files with different settings here will overwrite each other. In order to distinguish and identify specific state files which are not differentiated by the automatically encoded conditions, you can use a State_reference (for reading or writing) - this appends the reference to the state files.

So, if we want to write state files for a simulation which includes differences in these additional variables – let's say including a direct control modulation of I_{K1} – we will need a state reference to differentiate the state file with and without I_{K1} modulation:

./model_single_native Model hAM_CRN Beats 100 ISO 1 Write_state On Reference State_reference_example Results_Reference hAM_CRN_ISO_control_write

./model_single_native Model hAM_CRN Beats 100 ISO 1 IK1_scale 1.5 Write_state On **State_Reference_write IK1_scale_1p5** Reference State_reference_example Results_Reference hAM_CRN_ISO_IK1_write

Now, if we are reading in the state, we need to ensure we read in the correct one. Notice that:

./model_single_native Model hAM_CRN Beats 5 ISO 1 IK1_scale 1.5 Read_state On Reference State_reference_example Results_Reference hAM_CRN_ISO_control_read

reads in the state file written without the I_{K1} modulation (i.e. the resting potential at the start of the simulation does not match the end of the simulation performed with IK1_scale 1.5). Whereas:

./model_single_native Model hAM_CRN Beats 5 ISO 1 IK1_scale 1.5 Read_state On **State_Reference_read IK1_scale_1p5** Reference State_reference_example Results_Reference hAM_CRN_ISO_IK1_read

does read in the correct state file. Note the reference you pass is completely up to you and doesn't have to link directly to the additional modulation – use sensible references to identify and distinguish your simulations.

Another reason you may need to use state references would be to write the state at different time points during a simulation. As the simulation conditions are identical, you need state references to identify specifically the time point you wrote the state file.

9. Settings file example

Example 7 above, "Multiple and direct modulation", required the application of multiple modulation run-time arguments. In order to tidy this up a little, and not require so many arguments, we can use a settings file to encode most or all of the arguments we want to pass. Settings files can be used in combination with further command-line arguments, as long as the **settings file argument is passed first**. Any command-line arguments passed after then supplements or overwrite the arguments in the settings file.

So, for example, to repeat example 7 but this time for multiple cell models and varying the ICaL_scale amount (but not the others), we could first create a settings file called "SF_example_modulation.txt" with the following contents:

11
BCL 1000
Beats 10

Celltype PV
ISO 0.5
INa_scale 0.5
Jup_scale 0.5
Ito_va_tau_scale 5
ICaL_vi_ss_shift -10
IKur_va_ss_kscale 2
Ito_vi_tau_shift 10
Reference Direct_modulation_settings_file

And then call this file in a loop over different cell models can ICaL scales:

For *model* in hAM_CRN hAM_GB hAM_NG
For *ical* in 0 0.5 1 1.5 2
./model_single_native Settings_file SF_example_modulation.txt Model *model* ICaL_scale *ical* Results_Reference *model_ical*

Which will now create specific Results directories for each model and ICaL_scale.

10. Applying a voltage clamp

We can also apply simple voltage clamp protocols (the details of which have been hard-coded into the source code; you need to change the code in order to include different or more complex voltage clamp protocols in **lib/Model.c**). Passing Vclamp On will apply three simple voltage clamp protocols (I_{CaL}, I_K and I_{K1}) before the simulation is performed (or set Beats to 0 to perform the voltage clamp only).

./model_single_native Model hAM_CRN Beats 0 Vclamp On Reference Voltage_clamp_example

And now the data for the voltage clamp is found in:

"Outputs_single_native_Voltage_clamp_example/Parameters/I{CaL/K}_Vclamp_trace{s/_Y.dat}" and

"Outputs_single_native_Voltage_clamp_example/Parameters/I{CaL/K/K1}_IV.dat"

6.2 Tissue model examples – idealised tissue models

1. Basic pacing in an idealised tissue model at different dimensional orders

We will use the idealised tissue model "basic" and apply control pacing in a 1D strand, 2D sheet and 3D cuboid:

./model_tissue_native Model minimal BCL 500 Beats 20 Tissue_model basic Tissue_order 1D Reference Basic_model_pacing_1D

./model_tissue_native Model minimal BCL 500 Beats 2 Tissue_model basic Tissue_order 2D Reference Basic_model_pacing_2D

./model_tissue_native Model minimal BCL 500 Beats 1 Tissue_model basic Tissue_order 3D Reference Basic_model_pacing_3D

This will create an Outputs directory named for the model: "Outputs_tissue_native_Basic_model_pacing_{1D/2D/3D}". In the Outputs directories are the files "Geometry_idealised.vtk" and the stimulus and diffusion map files: "Map_{S1/S2}.vtk" and "Map_{D1/D2}.vtk". Notice for the 1D models, these contain just one line of data, with either the value of D1 or, for the stim map, 1s for stimulus regions and 0s for non-stimulus regions. For the 2D or 3D models, we can visualise this using visualisation software (such as the free Paraview (tm) program).

Our results are now in "Results", which contains files "Currents_cell{1-3}.dat" and "Properties_cell{1-3}.dat" - simply the single-cell outputs, as produced using model_single_native, for three different cells in the tissue. "Results/Vm_linescan_x" contains a linescan of voltage in the x-direction, each line for 1 ms intervals. "Spatial_Results" then contains outputs of the voltage in all space at specific time points (by default, binary data with an interval of 5 ms). The next example gives an example of how to convert these binary data into plain text and/or vtk.

2. Convert binary data to plain text and/or vtk files

As, by default, the model outputs spatial data only in binary form, we need to use the tool "**bin_to_vtk_tissue**" to convert the binary data into plain text and/or vtk. This must be executed in the same directory as the simulations were performed, or a parent directory which contains the "Outputs_" directories required.

In order to access the data correctly and with the correct settings, we must pass arguments:

1. Any Reference and/or Results_Reference passed (to look in correct Outputs and Spatial_Results directories)
2. The variable we wish to convert (Vm only for native tissue models by default)
3. The start, end and interval time over which convert data (binaries written at interval of 5 ms as default)
4. The Tissue_order and Tissue_model used to perform the simulation (necessary for array sizes etc)
5. The data you want to write: Write_vtk, Write_data, On or Off
6. The model type (integrated or native)

So, for the 2D and 3D simulations performed in the previous example, we will run: (note – we did not pass a Results_Reference for the simulations, so don't pass one here!)

Convert the 2D data into vtk and plain text, for the second beat (t = 500 ms to 1000 ms in 20 ms intervals):

```
./bin_to_vtk_tissue Reference Basic_model_pacing_2D Tissue_model basic Tissue_order 2D start_time 500 end_time 1000 interval 20 Variable Vm Write_vtk On Write_data On Model_type native
```

Convert the 3D data into vtk only, for the first beat (t = 500 ms to 1000 ms in 10 ms intervals):

```
./bin_to_vtk_tissue Reference Basic_model_pacing_3D Tissue_model basic Tissue_order 3D start_time 0 end_time 500 interval 10 Variable Vm Write_vtk On Write_data Off Model_type native
```

And now you'll see in the Spatial_Results directories are also .dat and .vtk files.

3. Controlling spatial outputs at run-time

We may want to change the interval over which binary data are written, or to output vtk files directly from the simulation for fast inspection of results, or output no spatial data at all if the excitation data is sufficient and we want to save disk space. To do this, we use "Spatial_output_interval_{data/vtk} [x]", where "0" will prevent data being output.

We will run the same simulation as example 1, for the 2D case, except now we will output binary spatial data every ms, and a vtk for inspection every 10 ms.

```
./model_tissue_native Model minimal BCL 500 Beats 2 Tissue_model basic Tissue_order 2D Reference Basic_model_pacing_2D Results_Reference Controlling_output_intervals Spatial_output_interval_data 1 Spatial_output_interval_vtk 10
```

Or output no spatial data:

./model_tissue_native Model minimal BCL 500 Beats 2 Tissue_model basic Tissue_order 2D Reference Basic_model_pacing_2D Results_Reference Preventing_spatial_data_output Spatial_output_interval_data 0 Spatial_output_interval_vtk 0

Notice now, in "Outputs_tissue_native_Basic_model_pacing_2D", we have new Results and Spatial_Results directories, with the Results_References appended. Note that in "../Spatial_Results_Controlling_output_intervals", there are binary data at intervals of 1 ms rather than 5 ms, and a vtk at intervals of 10 ms, while in "../Spatial_Results/Preventing_spatial_data_output" there are no spatial data. Also note that the Currents data in "../Results_X/" are the same for all simulations, as the conditions were identical.

We may now want to convert the 1ms interval binary data into vtk files, for which we use the "bin_to_vtk_tissue" tool, as before, but now need to also pass the Results Reference:

./bin_to_vtk_tissue Reference Basic_model_pacing_2D Tissue_model basic Tissue_order 2D start_time 500 end_time 700 interval 1 Variable Vm Write_vtk On Write_data On Model_type native Results_Reference Controlling_output_intervals

Note how now, vtk files before 500 ms have intervals of 10 ms (as output by the simulation), whereas between 500 and 700 the vtk interval is 1ms.

4. Controlling stimulus spatial properties

There are different ways we can control the stimulus spatial properties from the command-line (i.e. to differ from any default settings). For the Tissue_model basic, which we used for the above examples, the default settings for both S1 and S2 stimulus "edge" with a shape "cuboid" – which simply means stimulate from the small-x end of the tissue, using a cuboid stimulus shape.

The first way we can change the stimulus spatial settings is to pass in some of the other pre-defined types, and change the shape from cuboid to sphere. These pre-defined types set the location and sizes of the stimulus in all directions, based on the tissue dimensions.

First, let's make the S1 stimulus from the centre and a sphere/circular shape (we limit total time as this is sufficient to inspect the stimulus):

./model_tissue_native Tissue_model basic Tissue_order 2D Stimulus_location_type centre S1_shape sphere Reference Stimulus_S1_centre_sphere Total_time 20 Spatial_output_interval_vtk 5

We can now inspect the stimulus site using "Outputs_tissue_native_Stimulus_S1_centre_sphere/Map_S1.vtk" and can check the vtk files for voltage evolve as we would expect.

We can set the S1 stimulus to be "edge", "centre" or "whole-tissue" and shape to be "cuboid" or "sphere". Using "S2_Stimulus_location_type", we can also do the same for the S2 stimulus, where now our options are: "S1" – to be identical to the S1 stimulus; "edge", "centre" or "cross_field".

Let's try setting an S2 of 300 ms and set the stimulus to be "cross_field":

./model_tissue_native Tissue_model basic Tissue_order 2D Reference Stimulus_S2_cross_field Total_time 500 Beats 1 S2 300 S2_Stimulus_location_type cross_field Spatial_output_interval_vtk 5

And we can inspect the S2 stimulus settings in "Outputs_tissue_native_Stimulus_S2_cross_field/Map_S2.vtk".

Finally, we can also control explicitly the location (centre-points) and sizes (distance from the centre to the edge) of both stimuli. We can define every single value ourselves, or start from a pre-defined setting and overwrite just some parameters.

Let's start with Stimulus_location_type edge, but move it in the x-direction; let's also set the S2 stimulus to be centre, move its centre in y and adjust its size in x and y:

./model_tissue_native Tissue_model basic Tissue_order 2D Reference Explicit_stimulus_settings Stimulus_location_type edge S1_shape cuboid S1_x_loc 50 S2_Stimulus_location_type centre S2_shape cuboid S2_y_loc 25 S2_x_size 15 S2_y_size 5 S2 300 Total_time 500

Note: if we set the shape to "sphere", then the only size parameter is the radius (as elliptical stimulus functionality has not been included yet) – the only size argument we can use is "..x_size", to define the radius; y_size and z_size will have no affect if shape is sphere.

5. Heterogeneity, anisotropy and diffusion tensor spatial variation

We will now show examples of turning on or off heterogeneity, anisotropy and D_uniformity. To turn any of these on, first, the tissue model must have appropriate settings (in **lib/Tissue.cpp** – see the available functionality of each tissue model in the full arguments section or AVAILABLE_MODEL_LIST.txt) – we will use the Tissue model "functional_model_test" now, as this has settings for all of these functionalities, with the defaults set to off (homogeneous, isotropic and uniform).

First, let's run the model with defaults (so we can make comparisons):

./model_tissue_native Tissue_order 2D Tissue_model functional_model_test Reference functional_test_defaults Total_time 500

Note that the on-screen settings show: Tissue type = homogeneous, Orientation type = isotropic and D_uniformity = uniform.

Now, let's make the model heterogeneous, anisotropic, and vary D according to region:

./model_tissue_native Tissue_order 2D Tissue_model functional_model_test Reference functional_test_het_aniso_regional_D Total_time 500 Tissue_type heterogeneous Orientation_type anisotropic D_uniformity regional

First, we can compare "Outputs…Defaults/Geometry_idealised.vtk" with the file in "Outputs…het_aniso_regional_D/" to inspect the geometry and the celltype segmentation.

We can also compare "Outputs…Defaults/Map_{D1/D2}.vtk" with those in "Outputs…_het_aniso_regional_D" and confirm that they both do, indeed, vary in space. Moreover, "Outputs…het_aniso_regional_D" also contains a file "Orientation.vtk" of the fibre orientation (note this is global with them all pointing in the same direction).

Let's also inspect the evolution of voltage in the tissue:

./bin_to_vtk_tissue Tissue_model functional_model_test Tissue_order 2D start_time 0 end_time 500 interval 10 Reference functional_test_defaults

./bin_to_vtk_tissue Tissue_model functional_model_test Tissue_order 2D start_time 0 end_time 500 interval 10 Reference functional_test_het_aniso_regional_D

And we can clearly see the influence of the myocyte orientation, regionally dependent D1 and D2, and the heterogeneity (and thus repolarisation time) on the activation and repolarisation patterns.

Note that we need heterogeneity settings in order to apply regional D uniformity (as it depends on cell number in the tissue geometry), but we can set the Tissue_type to homogeneous to apply homogeneous electrophysiology.

6. Setting the diffusion parameters at run-time

You can explicitly set the values of the diffusion properties (D1 and D_AR) and the spatial integration step (dx) through command-line arguments. Note: If D_uniformity is not uniform, then D1 and D_AR set the baseline values, from which regional variation is scaled. Let's run the heterogeneous, anisotropic and regional D simulation from the previous example, except with a larger D1, larger anisotropy ratio, and larger space step. You may have noticed the on-screen outputs for the previous simulation (also in "Outputs_tissue_native_functional_test_het_aniso_regional_D/Settings.dat") that this model has default settings: D1 = 0.2 mm/ms; D_AR = 4; dx = 0.2 mm.

```
./model_tissue_native Tissue_order 2D Tissue_model functional_model_test Reference functional_Diffusion_params Total_time 500 Tissue_type heterogeneous Orientation_type anisotropic D_uniformity regional D1 0.3 D_AR 10 dx 0.25
```

Now, inspecting the D1 and D2 maps compared to the previous simulation should clearly show the larger D1 and anisotropy ratio (smaller D2 relative to D1). And if we analyse the voltage evolution data, we can see the impact this has (also reflected in the Activation_output.vtk data):

```
./bin_to_vtk_tissue Tissue_model functional_model_test Tissue_order 2D start_time 0 end_time 500 interval 10 Reference functional_Diffusion_params
```

7. Setting the global orientation at run-time

Idealised tissue models can only have global myocyte orientation (i.e. the same orientation for every cell in the tissue). You can directly control what this orientation is, through either explicitly setting each component (**OX**, **OY**, **OZ)** or by selecting a pre-set global orientation direction using the **Global_orientation_direction** argument. If setting directly, you only need to specify N-1 components, as the code will automatically set the final component from the normalisation constraint. Obviously, Orientation_type anisotropic must be set in order for the orientation to have any effect.

Let's set the stimulus to be centre and sphere, and change the default components explicitly, in both 2D and 3D:

```
./model_tissue_native Tissue_order 2D Tissue_model functional_model_test Stimulus_location_type centre S1_shape sphere Total_time 200 Orientation_type anisotropic OX 1 Reference 2D_OX_1 Spatial_output_interval_vtk 10
```

```
./model_tissue_native Tissue_order 2D Tissue_model functional_model_test Stimulus_location_type centre S1_shape sphere Total_time 200 Orientation_type anisotropic OY 0.2656 Reference 2D_OY_0p2656 Spatial_output_interval_vtk 10
```

```
./model_tissue_native Tissue_order 3D Tissue_model functional_model_test Stimulus_location_type centre S1_shape sphere Total_time 200 Orientation_type anisotropic OX 0.7 OY 0.1 Reference 3D_OX_0p7_OY_0p1 Spatial_output_interval_vtk 10
```

```
./model_tissue_native Tissue_order 3D Tissue_model functional_model_test Stimulus_location_type centre S1_shape sphere Total_time 200 Orientation_type anisotropic OX 0.2 OZ 0.5 Reference 3D_OX_0p2_OZ_0p5 Spatial_output_interval_vtk 10
```

Note that the on-screen settings (and in Settings.dat) prints the values of all the orientation components. Visualise the orientations using "Outputs_X/Orientation.vtk" and check they align with what is expected from the settings, and do indeed control the preferential direction of propagation correctly (which should be apparent from the voltage spatial outputs and the activation map).

We can also use Global_orientation_direction to set the fibres pointing in any of the principal or diagonal directions. Let's set Total_time to zero so we can quickly output the Orientation for different pre-set directions.

For *orientation* in X Y Z XY_plus XY_minus XZ_plus XZ_minus YZ_plus YZ_minus XYZ_ppp XYZ_ppm XYZ_pmp XYZ_mpp

> ./model_tissue_native Tissue_order 3D Tissue_model functional_model_test Total_time 0 Orientation_type anisotropic Global_orientation_direction *orientation* Reference *orientation*

And again, we can inspect the orientation in "Outputs_X/Orientation.vtk"

8. Using model "conduction_velocity" to parameterise a tissue model

The **Tissue_model conduction_velocity** has been provided to calculate and parameterise conduction velocities relative to the myocyte orientation. For this specific model only, certain cells are set for the conduction velocity to be automatically calculated in all principal and diagonal directions (rather than requiring post-processing of the results data to make these calculations). Combining this tissue model with direct settings of **D1**, **D_AR** and **dx** allows other tissue model properties to be matched, to provide a clean method to calculate and parameterise these parameters to match experimentally measured conduction velocities. The model must be run using homogeneous, where the influence of celltype on conduction velocity can be investigated by passing different celltypes.

By default, the model is 2D and sets the global orientation in the x-direction, and applies a spherical stimulus to the centre; the conduction velocity in the x-direction thus gives the longitudinal velocity, and in the y-direction gives the transverse conduction velocity. The velocities and diffusion parameters are stored in the file "Outputs_tissue_native_X/Conduction_velocity_log.dat" and output to screen.

Let's run the model with default parameters, then set a different dx (to correspond to the space step of an anatomical reconstruction) and cycle through a couple of diffusion parameter combinations to see the effect on the conduction velocity. We'll use just 1 beat here to save time; ensure you are at stable state for real parameterisation. We'll use Results_Reference to distinguish individual simulations, such that a list of our conduction velocity data is stored in Conduction_velocity_log.dat.

./model_tissue_native Tissue_model conduction_velocity Beats 1 BCL 500 Reference Conduction_velocity Results_Reference default_params

./model_tissue_native Tissue_model conduction_velocity Beats 1 BCL 500 dx 0.3 Reference Conduction_velocity Results_Reference dx

./model_tissue_native Tissue_model conduction_velocity Beats 1 BCL 500 dx 0.3 D1 0.25 D_AR 8 Reference Conduction_velocity Results_Reference dx_D1_D_AR_1

./model_tissue_native Tissue_model conduction_velocity Beats 1 BCL 500 dx 0.3 D1 0.35 D_AR 10 Reference Conduction_velocity Results_Reference dx_D1_D_AR_2

We are then interested in Conduction_velocity_log.dat which lists D1 (column 2), D2 (3), dx (4) and the conduction velocities in the x-direction (longitudinal; column 8), y-direction (transverse; column 9) and the xy-diagonal-directions (11 and 12).

We can also set the model to 3D for a more accurate representation of a 3D tissue model, or change the global orientation to point in any direction and calculate the conduction velocity in all the principal and diagonal directions.

9. Applying cellular and tissue modulation and modulation maps

Cellular modulation (ISO, Remodelling, ICaL_scale etc) can be applied to tissue models exactly as with single-cell models. By default, they apply homogeneously to the tissue, on top of any local heterogeneity due to celltype. We can also apply tissue modulation – i.e. Dscale and/or D_AR_scale, to multiply the values of D1 and D2 or the anisotropy ratio, relative to the set values.

Let's create a control and modulation simulation:

./model_tissue_native Tissue_order 2D Tissue_model functional_model_test Total_time 200 Reference Tissue_control

./model_tissue_native Tissue_order 2D Tissue_model functional_model_test Total_time 200 ISO 1 ICaL_scale 0.5 Dscale 2 D_AR_scale 4 Reference Tissue_modulation

We can also define whether to apply the modulation homogeneously, or by a spatial map. Whereas we can individually turn the modulation map on or off for all different modulations (Dscale_mod_map, D_AR_mod_map, Remodelling_map, ISO_map, ACh_map, Direct_modulation_map [Off/On]), we can only use a single map itself for idealised models. If you require different maps for different modulations, you must use an anatomically detailed model and provide map files. Note: "Direction_modulation_map" will control where any direct_modulations are applied (ICaL_scale, INa_va_shift, IKur_vi_tau_scale etc).

Let's apply Dscale and ISO by a spatial map, but leave D_AR_scale and ICaL_scale to apply homogeneously. Maps are defaulted to off, but we will include explicit settings for each map here just for clarity.

./model_tissue_native Tissue_order 2D Tissue_model functional_model_test Total_time 200 ISO 1 ICaL_scale 0.5 Dscale 2 D_AR_scale 4 Dscale_mod_map On D_AR_scale_mod_map Off ISO_map On Direct_modulation_map Off Reference Tissue_modulation_map

Note that in "Outputs_tissue_native_Tissue_modulation_map" there are now two additional files: Map_Dmod .vtk and Map_ISO.vtk, each with a region of 1s and a region of 0s. This number is what determines the magnitude of the modulation – so where it is 1, ISO will be set to the value of ISO and Dscale_mod will be set to the value of Dscale. Where it is 0, ISO = 0 and Dscale = 0 and thus parameters = control. You can also visualise D1 (D1_map.vtk) to see if the modulation map has the intended effect.

We can control the shape, size and location of the map at run-time in an exactly analogous way to setting the stimulus spatial properties, by using map_shape, map_x_loc, map_x_size etc. As with stimulus, this sets the centre and size from centre to edge; spherical maps can only have an x_size which determines radius.

./model_tissue_native Tissue_order 2D Tissue_model functional_model_test Total_time 200 ISO 1 ICaL_scale 0.5 Dscale 2 D_AR_scale 4 Dscale_mod_map On D_AR_scale_mod_map Off ISO_map On Direct_modulation_map Off Reference Tissue_modulation_map_controlled map_shape sphere map_x_loc 90 map_x_size 5 map_y_loc 7

Finally, let's also turn D_uniformity to regional to demonstrate how regional D and Dscale with a map can combine:

./model_tissue_native Tissue_order 2D Tissue_model functional_model_test Total_time 0 ISO 1 ICaL_scale 0.5 Dscale 2 D_AR_scale 4 Dscale_mod_map On D_AR_scale_mod_map Off ISO_map On Direct_modulation_map Off Reference Tissue_modulation_map_and_regional_D map_shape sphere map_x_loc 90 map_x_size 35 map_y_loc 7 D_uniformity regional

And you may notice now that D1_map.vtk has clear regional variation, and an additional area where the modulation map is applied (we made the map larger so we can see it affecting different regions).

10. Reading and writing state in tissue models; efficient pre-pacing protocols

There are multiple ways to read and write state files using tissue models.

Setting **Write_state** to **On** will write the state of the whole tissue, which can then be read in with **Read_state On**. Setting **Write_state** to **ave** will write the state of the centre cell in the tissue, which can then be read into the whole tissue with **Read_state ave** (you will need to create a state file for every celltype and modulation condition used in your tissue model). Finally, you can also read in state files created by the single cell models using **Read_state single cell**.

Read/Write_state On can be used to save whole tissue states for more efficient simulations (e.g. pace to stable state, then test S2 intervals and locations, or pharma action, or similar). The **single cell** and **ave** functionality can be used sequentially to to get to stable state on a large tissue model more efficiently.

Let's explore that, using Tissue_model functional_model_test at a rapid rate, with heterogeneity turned on, and ISO applied according to a map (reproducing a complex situation).

First, we'll create state files using the single cell models paced to stable states. We need a state file for each celltye in the model (EPI, M and RA) and with and without ISO.

For *celltpye* in ENDO, M, RA

For *iso* in 0 1

./model_single_native Model minimal BCL 400 Celltype *celltype* ISO *iso* Beats 200 Write_state On Reference single_cell_pre_pace Results_Reference *celltype_iso*

Now we'll use these state files as a starting point, and pace a 1D homogeneous tissue for each condition to output the coupled cell properties. We'll use fewer beats as we already paced the single cell models to stable state over 200 beats. Note: we need to set some stimulus properties, as the default for this tissue model (centre and sphere) does not create a stimulus region in 1D (due to the sphere; we also move region to edge so that we are not writing the state of a cell in the stimulus region).

For *celltpye* in ENDO, M, RA

For *iso* in 0 1

./model_tissue_native Tissue_model functional_model_test Tissue_order 1D Model minimal BCL 400 Stimulus_location_type edge S1_shape cuboid Tissue_type homogeneous Celltype *celltype* ISO *iso* Beats 20 Read_state single_cell Write_state ave Reference Tissue_state_read Results_Reference ave_coupled_write_celltype_iso

And finally, we'll read the average coupled state files into our full 2D or 3D tissue model, pace for five beats, and write the whole tissue state:

./model_tissue_native Tissue_model functional_model_test Tissue_order 2D Tissue_type heterogeneous Model minimal BCL 400 ISO 1 ISO_map On Beats 5 Read_state ave Write_state On Reference Tissue_state_read Results_Reference ave_coupled_read_whole_tissue_write

Which can now be read in to start a new simulation from that point:

./model_tissue_native Tissue_model functional_model_test Tissue_order 2D Tissue_type heterogeneous Model minimal BCL 400 ISO 1 ISO_map On Beats 2 Read_state On Reference Tissue_state_read Results_Reference whole_tissue_read

This process is limited if we are using complex tissue models with continuous maps, as creating state files for every individual condition becomes infeasible or impossible. The ISO and ACh concentrations are written to two decimal places in the state files, so if a continuous ISO or ACh map is used, we would need

to create a state file for every celltype and every concentration in two decimal place intervals between 0 and max concentration; other modulation proportions or maps (Remodelling_prop, Direct_modulation map) are not encoded into state files and so tissue models which contain these cannot read from single_cell or ave coupled cell state files; only full state reading and writing will work. For heterogeneity and discrete ISO, ACh, mutation and Remodelling maps, we can feasibly use the above process to create state files for every celltype and modulation condition in the tissue model.

11. Using multiple baseline cell models for different regional celltypes

Functionality to use two different cell models for different regions has been included. For example, to use a ventricular and atrial cell model for different parts of a whole-heart model. The tissue model must have the appropriate settings, and the celltypes must exist for each region which that cell model is applied to.

We'll again use functional_model_test, where the heterogeneity celltypes are set to ENDO, M and RA. We'll turn Multiple_models On, use the minimal model for the ventricular cell types (model 1) and set the second model (applied for region RA) to an atrial cell model.

./model_tissue_native Tissue_model functional_model_test Tissue_type heterogeneous Multiple_models On Model minimal Tissue_model_2 hAM_GB Beats 2 Reference Multiple_model_example

12. Calculating a 1D Vulnerability window

You can use any 1D tissue model to calculate the vulnerability window for conduction block at different S2 locations and coupling intervals. We'll use the model "Vent_transmural" which has celltypes EPI, M and ENDO evenly spaced. In this example, we'll just consider a couple of S2 locations and a coarse coupling interval, but you could cycle through S2 locations at any interval to provide a picture over the whole tissue. You should also pre-pace the model to stable state at the desired BCL and write the state file to read in for the vulnerability window calculations, for actual investigations.

For *s2_location* in 25 75

    For *s2* in sequence 200 to 150 in steps of -5

    ./model_tissue_native Tissue_model Vent_transmural Tissue_order 1D Beats 1 S2 *s2* S2_x_loc *s2_location* Reference Vulnerability_window Results_Reference location_*s2_location*_S2_*s2*

And now the file "Outputs_tissue_native_Vulnerability_window/1D_conduction_success_log.dat" contains a list of the S2 locations (column 1), coupling intervals (column 2), conduction success at either edge of the tissue (columns 3 and 4, where 0 = failure and 1 = success) and the conduction type (column 5, where 0 = failure, 1 = unidirectional conduction and 2 = full conduction). The raw data can be inspected using "Outputs_tissue_native_Vulnerability_window /Results_location_x_S2_y/Vm_linescan.dat." For a quick inspection (rather than plotting the full colour-map linescan in 2D), you can simply plot columns 10 and 90, representing the voltage in cells 10 and 90 with the x-axis intervals representing the 1 ms increments of linescan output.

In this example, we can see that for the location at x = 25, the S2 interval 155 resulted in uni-directional conduction and for x = 75 it occurred for 185 and 180. We could then cycle within those coupling intervals in 1 ms increments to determine the full vulnerability window to an accuracy of 1ms.

13. Inducing re-entry example using cross-field stimulation

Tissue_model re-entry has been designed for simulations of re-entry in idealised 2D and 3D sheets. It is a relatively large tissue, with S1 stimulus from the x=0 edge and the default S2_Stimulus_location_type set to "cross_field". Thus, passing an S2 argument will apply cross-field stimulation in an attempt to induce re-entry. By default, the model is isotropic, but anisotropy can be included by setting Orientation_type to anisotropic and setting the orientation directions as in example 7. You may also want to define the diffusion parameters at run-time (example 6) as the defaults may not be desired for your investigation.

In this example, we'll set D1 and dx directly, and set Dscale to 0.75 to promote sustained re-entry. We'll use the state write and read functionality to pace to stable state, and save the stable re-entry.

First, we'll pace a 1D model with matched D1 and dx to stable state at a relative rapid pacing rate. We'll write the state as an ave coupled cell, and pass a state reference to identify the specific tissue simulation.

./model_tissue_native Tissue_model re-entry Tissue_order 1D D1 0.25 dx 0.3 Dscale 0.75 BCL 300 Beats 100 Write_state ave State_Reference_write Re_entry_1D_pre_pace Reference Re_entry_example Results_Reference 1D_pre_pace

We'll then pace the 2D re-entry tissue model for a couple beats to settle it into a stable state (you should ensure this is actually the case for any real investigations) and write the whole-tissue state file (with another reference – this is now very important as we will also perform a whole-tissue state write after inducing re-entry, and we want to preserve the separate state files):

./model_tissue_native Tissue_model re-entry D1 0.25 dx 0.3 Dscale 0.75 BCL 300 Beats 2 Read_state ave State_Reference_read Re_entry_1D_pre_pace Write_state On State_Reference_write Re_entry_2D_pre_pace Reference Re_entry_example Results_Reference 2D_pre_pace

And finally, apply just one S1 beat, an S2 of 155 (or cycle through different intervals – which is why we wrote the tissue state at stable S1 pacing). These parameters don't actually lead to sustained re-entry – it self-terminates within one second – so we will set the Total_time to 450, where a re-entrant circuit still persists, to illustrate the state-read protocol next.

./model_tissue_native Tissue_model re-entry D1 0.25 dx 0.3 Dscale 0.75 BCL 300 Beats 1 Total_time 450 S2 155 Read_state On State_Reference_read Re_entry_2D_pre_pace Write_state On State_Reference_write Re_entry_2D_stable_at_3secs Reference Re_entry_example Results_Reference S2_155

We can visualise the spatial data with:

./bin_to_vtk_tissue Reference Re_entry_example Results_Reference S2_155 Tissue_model re-entry start_time 0 end_time 450 interval 10

We can then read this (semi) stable re-entrant state to start any number of simulations from a stable re-entry point (for example to apply some Direct_modulation to test potential intervention to terminate the re-entry). Set beats to 0 as we don't want to apply a stimulus (we must still specify a BCL in order to read in the correct state file, which has the BCL in its filename).

./model_tissue_native Tissue_model re-entry D1 0.25 dx 0.3 Dscale 0.75 BCL 300 Beats 0 Total_time 500 Read_state On State_Reference_read Re_entry_2D_stable_at_3secs IKr_scale 0.25 IKs_scale 0.25 Reference Re_entry_example Results_Reference Termination_attempt

And create vtk files of the data:

./bin_to_vtk_tissue Reference Re_entry_example Results_Reference Termination_attempt Tissue_model re-entry start_time 0 end_time 500 interval 10

14. Inducing re-entry using the phase-distribution method

An alternative method to induce re-entry is to use the phase-distribution approach – where state files for a coupled cell are written throughout the duration of an AP (representing 0 - 2π) and then read in as initial-conditions applied to a phase map.

First, we must create the phase state files. This must be performed in 1D, with a BCL of 400 ms or longer. This is because the state files are output over an interval of 400 ms, hard coded in the model; you may change this in the appropriate main tissue file.

./model_tissue_native Tissue_order 1D Tissue_model re-entry Beats 2 BCL 600 Write_state phase Reference Phase_re-entry Results_Reference phase_write

And now we read in this phase file to a 2D or 3D tissue model (we are using Tissue_mdoel re-entry, but this is not required), and ensure we set beats to 0.

./model_tissue_native Tissue_order 3D Tissue_model re-entry Beats 0 BCL 600 Read_state phase Total_time 500 Reference Phase_re-entry Results_Reference phase_read Spatial_output_interval_vtk 20

We can visualise the phase map itself in "Outputs_tissue_native_Phase_re-entry/Phase_Map_3D.vtk".

Note: we can impose the phase method on a heterogeneous tissue model; doing so requires the phase files to be created for every celltype in the tissue model (the code then automatically reads the correct files for each region).

6.3 Tissue model examples – anatomically detailed/complex tissue models

Functionality for anatomically detailed models is largely the same as idealised models, where now a geometry file must also be provided. A few different, previously published tissue models are available, as well as "**functional_model_test**" which has example implementations of all functionality. The primary difference here is that we now have the ability to read in maps (stimulus, diffusion tensor spatial non-uniformity, modulation and phase) from file, allowing more complex and continuous implementations than the simple maps created in the code for idealised models. Thus, you could also use these types of models to create semi-idealised models which need to implement complex gradient maps. This section outlines the primary differences and new functionality of these models – you can also follow idealised examples above using an anatomically detailed model for anything not directly listed here.

1. Basic pacing with an anatomically detailed model

Simple example using an anatomically detailed model (**Tissue_order geo**). We'll use **Human_vent_wedge** for this first illustration.

./model_tissue_native Tissue_order geo Tissue_model Human_vent_wedge Reference Human_vent_control Beats 1 BCL 500

We can view the geometry in "Outputs_tissue_native/Human_vent_control/Geometry_anatomy.vtk", and visualise results spatial data (note now it is important PATH.txt is in the directory from which "**./bin_to_vtk_tissue**" is being performed, as it now needs to be able to load the correct tissue geometry file):

./bin_to_vtk_tissue Tissue_order geo Tissue_model Human_vent_wedge Reference Human_vent_control start_time 0 end_time 500 interval 20

2. Spatial gradient map and model

Unlike idealised models, we can now also impose a spatial-gradient model. This may be apico-basal ventricular heterogeneity, or a gradual shortening of the AP with distance from SAN, or similar. i.e. a spatial gradient imposed on top of possible underlying cellular heterogeneity.

All we need is 1) a map file containing the spatial gradient (continuous 0 – 1; must be in PATH -> Tissue_geometries) and 2) an electrophysiology gradient model to apply (we have two testing examples implemented, "apico_basal_example" and "SAN_distance_example", neither of which are based on real data or observations). **Functional model test** has an associated spatial-gradient file. Even though this

filename is set in the tissue model settings (in **lib/Tissue.cpp**), we will specify it at run-time as an example of how to do this (we could select between multiple available files this way) – note we just specify the file name, which is stored in PATH. In general, we do not need to pass in the filename directly; any default filenames set in the tissue model will be used, unless overwritten.

./model_tissue_native Tissue_order geo Tissue_model functional_model_test Spatial_gradient apico_basal_example Spatial_gradient_map_file functional_model_test_spatial_gradient.dat BCL 500 Beats 1 Reference Spatial_gradient_example

We can then have a look at the spatial gradient map, as well as the geometry itself, in "Outputs../Map_Spatial_gradient.vtk" – note that you may have to rescale the colourmap to 0-1 (rather than scaling for empty space of -100). Note also that the geometry is heterogeneous, and the spatial gradient applies on top of that

./bin_to_vtk_tissue Tissue_order geo Tissue_model functional_model_test Reference Spatial_gradient_example start_time 0 end_time 350 interval 10

And now we can see the effect of the spatial gradient and heterogeneity on repolarisation; the default D_uniformity is also regional, so there is variation in conduction velocity also.

3. Specifying stimulus map files or coordinates

We can specify different files for stimulus (S1 or S2), or set the stimulus using coordinates (just as with the idealised tissue models).

Let's first see what happens under default settings for this model – we'll set total time to 0 as we are only interested in the stimulus locations, which we can see from the map files produced.

./model_tissue_native Tissue_order geo Tissue_model functional_model_test Total_time 0 Reference Default_stimulus

You may notice the on-screen output during setup: "File loaded /…/MSCSF_state_and_geometry_files/Tissue_geometries/functional_model_test_stim.dat", which is the default file set in **lib/Tissue.cpp**, and we can inspect what this stimulus location will be in "Outputs../Default_stimulus/Map_S1.vtk". We can also pass an S2 argument to see what the default S2 stimulus file is and its location.

./model_tissue_native Tissue_order geo Tissue_model functional_model_test Total_time 0 S2 50 Reference Default_stimulus_S2

And we can see that it loads file "functional_model_test_S2.dat", which is output in "../Map_S2.vtk". There is also another S2 stimulus file available to us – "functional_model_test_S2_2.dat". Let's now set the S1 stimulus file to being the default S2 stimulus file, and the S2 file to be this alternative (there is nothing specific about S1 or S2 stimulus files – we can just name them as is most useful for us).

./model_tissue_native Tissue_order geo Tissue_model functional_model_test Total_time 0 S2 50 Reference Different_stimulus_files stim_file functional_model_test_S2.dat S2_stim_file functional_model_test_S2_2.dat

And we now notice that "Map_S1.vtk" looks like the previous S2 (as we would expect), while "Map_S2.vtk" is clearly a new file.

We can also set either or both stimuli by coordinates by passing "**{S2_}Stimulus_loc_type coords**". There are default coordinate settings in the tissue model, so in the first instance we don't need to speficy any coordinate parameters directly:

./model_tissue_native Tissue_order geo Tissue_model functional_model_test Total_time 0 Stimulus_location_type coords S2_Stimulus_location_type coords S2 50 Reference Default_coords_stimulus

And we can see that the S2 stimulus is perfectly setup for cross-field stimulation. We can also control any of the stimulus location parameters directly, just as with example 4 for idealised models (referring to the direct location and size settings; we cannot call the pre-set **Stimulus_location_type** settings in anatomically detailed models). Let's adjust some S2 parameters:

./model_tissue_native Tissue_order geo Tissue_model functional_model_test Total_time 0 Stimulus_location_type coords S2_Stimulus_location_type coords S2 50 S2_shape cuboid S2_y_loc 25 S2_x_size 15 S2_y_size 5 S2_z_size 1 Reference run_time_S2_coords_stimulus

Note: if the tissue model does not have default settings for the stimulus coordinates, all parameters must be set at run-time if "coords" set.

4. Setting D_uniformity from map or regional_map

The default setting for the **functional_mode_test** is for D to vary according to region/celltype – the same functionality as is available with idealised cell models (so long as the heterogeneity settings exist). A difference here, of course, is that our heterogeneity settings (tissue segmentation) is absorbed into the tissue geometry file – it either contains 1s and 0s only, or further numbers for segmentation – rather than having to set the segmentation properties as with an idealised model. So long as our tissue geometry file has multiple regions, we can impose electrical heterogeneity and/or D_uniformity by region. However, with anatomically detailed tissue models, we can also impose D_uniformity by a map, or by both a region and map (applied sequentially). The map multiples D1 and/or D_AR by the map value for every cell (so can be used to impose a D spatial variability which does not depend on celltype and/or which varies continuously, or checkerboard etc – anything you can make with a map!). Remember, this is the baseline D1 and D_AR associated with the model – not a remodelled region in which we want to change the diffusion parameters (which is controlled by Dscale, D_AR_scale, and Dscale_mod_map, D_AR_scale_mod_map – see next example) – and so is to correspond to different diffusion parameters around the tissue model as part of its control setup. When using a map file, we must provide a map file for both D1 and D_AR – if we want only one to vary spatially, just create a map of all 1s for the one we don't want varied.

So let's compare our D1 and D2 maps under uniform, regional, map and regional_map D_uniformity. We'll again set Total_time to 0 as we just need the maps for this illustration:

./model_tissue_native Tissue_order geo Tissue_model functional_model_test Total_time 0 D_uniformity uniform Reference D_uniformity_uniform

./model_tissue_native Tissue_order geo Tissue_model functional_model_test Total_time 0 D_uniformity regional Reference D_uniformity_regional

./model_tissue_native Tissue_order geo Tissue_model functional_model_test Total_time 0 D_uniformity map Reference D_uniformity_map

./model_tissue_native Tissue_order geo Tissue_model functional_model_test Total_time 0 D_uniformity regional_map Reference D_uniformity_regional_map

We notice that "../Map_{D1/D2}.vtk" for the uniform simulation are homogeneous (as we would hope!), whereas for the regional case, they clearly vary according to celltype (compare with the segmentation in "Geometry_anatomy.vtk"). In the map simulation, we can see there are now two map files: "Map_Duniformity.vtk" and "Map_D_AR_uniformity.vtk", and we can easily see how these maps correlate with the actual values of D1 and D2: remember, D2 depends on both D1 map and D_AR map, as D_AR is relative to D1. Finally, we can see how applying the map and regional variation combine

sequentially in the regional_map simulation example (seeing the smooth gradients in D1 in this last example may require rescaling between the ranges for each celltype, as the smooth gradient is imposed within discrete celltype variation.)

5. Modulation and modulation map files

As with idealised tissue models, we can apply modulation (ISO, ACh, Remodelling, Mutation, Direct_modulation, D_scale_mod, D_AR_scale_mod) either homogeneous or by a map. We can set the map using coordinates, exactly as with the idealised model example 9, using **map_in_type coords** (at least one map has to be turned on for it to do anything!):

./model_tissue_native Tissue_order geo Tissue_model functional_model_test Total_time 0 ISO 1 ISO_map On map_in_type coords Reference ISO_map_coords

And we can explicitly set the map coordinates and sizes as in that previous example. However, this is limiting in two major ways:

1) we are limited to discrete maps (0 or 1 for off or on, multiplied by total amount of ISO);

2) all modulation must be subject to the same map (if map is set to on for that modulation – we can still combine some by maps and others homogeneously).

To overcome these limitations, we can use map files – which we can create to contain any shape and continuity we like (note: Direct_modulation maps must still be discrete, as continuous imposition of these modulations is not implemented). We can either apply all modulations by coordinates or files – we cannot combine coordinates and files for different modulation maps.

Map files can be named to indicate the modulation they are designed for, but, as with stimulus maps, the actual name of the map file does not limit where it can it can be used. The **functional_model_test** has example maps files for all modulations, and are defaulted in the tissue mode settings. So, let's first apply all modulations, set the modulation map to "file" and set all modulations to be applied by a map. We won't pass any map files (so it reads the default settings) and will set D_uniformity to uniform (so we can easily interpret the D1 and D2 maps from the modulation). We need to pass some direct modulation in order for that map to have an effect. Note: the maps simply define the regions and proportion of maximum effect to which the modulation applies, so for ISO, ACh and Remodelling the maximum value of the map should be = 1, which will be the region where ISO, ACh or remodelling are applied according to their concentration/proportion variable (i.e. if ISO = 0.5, then the map region of 1 will apply ISO at a concentration of 0.5, and the map region 0.5 will apply concentration 0.25). So, passing different values of ISO, ACh and Remodelling_prop will affect your simulation, but will not be reflected in the maps. Similarly, the D_scale and D_AR_scale maps must also be between 0 and 1, where map=1 applies the full value of Dscale or D_AR_scale (which may be larger or smaller than 1). This is different to the baseline D1 and D_AR maps (example 4), which are absolute scale factors to multiply the parameters D1 and D_AR.

./model_tissue_native Tissue_order geo Tissue_model functional_model_test Total_time 0 D_uniformity uniform ISO 1 ISO_map On ACh 0.5 ACh_map On Remodelling test Remodelling_map On Remodelling_proportion 0.75 ICaL_scale 0.5 IK1_scale 2 Direct_modulation_map On Dscale 2 Dscale_mod_map On D_AR_scale 3 D_AR_scale_mod_map On map_in_type file Reference All_mod_maps_by_file

We can see on the run-time outputs that different map files are loaded for each modulation. We can now check all the map files, and that they are all different (note the Direct_modulation map is not continuous) and that the D1 and D2 maps correlate with "Map_Duniformity.vtk" and "Map_D_AR_uniformity.vtk", as in the previous example (again, remember D2 depends on D1 map and D_AR map).

Sometimes, we may want the same map to apply to multiple modulations – for example a remodelled region could be associated with electrical and diffusion dysfunction. We will use the functionality to set the map file at run-time in order to impose the same map on remodelling and Dscale:

./model_tissue_native Tissue_order geo Tissue_model functional_model_test Total_time 0 D_uniformity uniform Remodelling test Remodelling_map On remod_map_file functional_model_test_Dscale_mod_map.dat Dscale 0.5 Dscale_mod_map On Dscale_mod_map_file functional_model_test_Dscale_mod_map.dat Reference Remodelling_and_Dscale_same_map_file

We can of course combine D_uniformity (by region or maps) with Dscale and D_AR_scale maps, in order to apply baseline variation in D, with a remodelled variation imposed on top.

6. Multi-timed and site stimulus protocols

Using files rather than creating idealised stimulus regions easily allows for the stimulus to apply to multiple different locations. We can also apply a stimulus protocol where different regions are stimulated at different times (for example, simulating the Purkinje-fibre breakthrough sites in the ventricle during sinus rhythm). To do this, we need to ensure we have the right stimulus file and settings in **lib/Tissue.cpp** for this functionality (which just requires a stimulus file with different regions numbered sequentially in the order to apply the stimuli, and settings as to what the relative delay of each region is – see the functional_model_test setup for an example) and then to pass "**Multi_stim On**". As multi_stim is not the default for this tissue model, we also need to tell it to read the multi_stim stimulus file rather than the standard one. In general, if a model requires multi-stim functionality, it will likely be for control and so set the default stimulus file to the multi-stim file.

./model_tissue_native Tissue_order geo Tissue_model functional_model_test Total_time 100 Multi_stim On stim_file functional_model_test_multi_stim.dat Reference Multi_stim

First, we can inspect "Map_S1.vtk" and notice it now has 5 different regions (rather than just one), and applying the post-processing:

./bin_to_vtk_tissue Tissue_order geo Tissue_model functional_model_test Reference Multi_stim start_time 0 end_time 100 interval

We can clearly see the effect of the delayed stimulus points.

6.4 3D cell model examples

1. Basic, control pacing using the 3D cell model and testing and full simulation cell sizes

First, we'll use the **Sim_cell_size testing** model to perform a fast test 3D cell simulation

./model_single_3D Model minimal Beats 20 Sim_cell_size testing Reference Control_pacing_minimal Results_Reference testing

And now an example simulation using the full simulation cell size:

./model_single_3D Model minimal Beats 1 Total_time 1000 Sim_cell_size full Reference Control_pacing_minimal Results_Reference full

This will create an Outputs directory named for the model: "Outputs_3Dcell_Control_pacing_model". Within which results directory for the two model sizes "Results_{testing/full}". Further to "Results_{testing/full}/Currents.dat", which has time courses for the AP and ion currents, "Results_{testing/full}/CRU.dat" contains the intracellular Ca^{2+} concentrations and RyR, LTCC and SERCA dynamics. Linescan data (cross section through centre of cell) "Results_{testing/full}/Ca_linescan_{X/Y_Z}.dat". Spatial data for calicum concentrations in the 3D cell (Ca, CaSR, Cads) is "Spatial_Results_{testing/full}". Under the default settings, these spatial data will be output in binary format every 5 ms . See next example for how to convert binary spatial data to plain text and vtk (analogouos to the tissue models).

2. Convert binary data to plain text and/or vtk files

As, by default, the model outputs spatial data only in binary form, we need to use the tool "**bin_to_vtk_3Dcell**" to convert the binary data into plain text and/or vtk. This must be executed in the same directory as the simulations were performed, or a parent directory which contains the "Outputs_" directories required.

In order to access the data correctly and with the correct settings, we must pass arguments:

1. Any Reference and/or Results_Reference passed (to look in correct Outputs and Spatial_Results directories)
2. The variable we wish to convert (Ca, CaSR, CaDS)
3. The start, end and interval time over which convert data (binaries written at interval of 5 ms as default)
4. The Cell_size and Sim_cell_size
5. The data you want to write: Write_vtk, Write_data, Write_slices, On or Off
6. If writing slices, you can also specify the coordinate of the normal to slice at which the slice is taken (XY_slice_z [n], XZ_slice_y [n], YZ_slice_x [n]).

First, let's run an additional simulation to assist this illustration:

```
./model_single_3D Model minimal Beats 1 Total_time 100 Cell_size thin Sim_cell_size full Reference Thin_cell_pacing_minimal
```

Visualise intracellular calcium at 10 ms intervals in testing cell control simulation:

```
./bin_to_vtk_3Dcell Reference Control_pacing_minimal Results_Reference testing Sim_cell_size testing start_time 0 end_time 50 interval 10 Variable Ca
```

Visualise dyadic calicum at 5 ms intervals; write vtk and text data; full cell control simulation:

```
./bin_to_vtk_3Dcell Reference Control_pacing_minimal Results_Reference full Sim_cell_size full start_time 0 end_time 50 interval 5 Variable Ca Write_data On
```

Visualise intracellular calcium slices (plain text only) for the thin cell full size simulation (ran above); output xz slices at surface and centre. Don't produce vtk files or 3D text data for this case.

```
./bin_to_vtk_3Dcell Reference Thin_cell_pacing_minimal Sim_cell_size full start_time 0 end_time 50 interval 5 Variable Ca Write_data Off Write_vtk Off Write_slices On XZ_slice_y 0
```

```
./bin_to_vtk_3Dcell Reference Thin_cell_pacing_minimal Sim_cell_size full start_time 0 end_time 50 interval 5 Variable Ca Write_data Off Write_vtk Off Write_slices On XZ_slice_y 7
```

```
./bin_to_vtk_3Dcell Reference Thin_cell_pacing_minimal Sim_cell_size full start_time 0 end_time 50 interval 5 Variable Ca Write_data Off Write_vtk Off Write_slices On XZ_slice_y 15
```

We can also directly control the spatial outputs at run-time, exactly as in the idealised tissue model example 3.

3. Modulation with 3D cell models

We can apply modulation to the 3D cell models in exactly the same way as native cell models. One key different aspect here is the different ways to control RyR and LTCC activity:

- Change in channel expression: ICaL_scale and Jrel_scale will scale NRyR and NLTCC (ave per dyad)
- Change in channel activity: LTCC_Po and RyR_Po will scale the transition rate to the open, active channel state (for LTCC this is a voltage independent transition from the voltage activation gate; for RyRs, it scales the transition rate from C to O i.e. sensitivity to CaDS). These control the model variables GLTCC_kva1_va2 and GRyR_kCO.

We can apply both types of modification simultaneously (e.g. remodelling reduces channel numbers, but increases RyR sensitivity). As an example, let's compare what happens when we increase NLTCC and the I_{CaL} open rate - both increase channel activity, different ways. Check the screen settings outputs for NLTCC to see how it changes according to GCaL. We'll turn voltage clamp on (which for the 3D cell model will clamp I_{CaL} using the stochastic spatial CICR) so we can compare peak current (Note that with the testing Sim_cell_size, the I_{CaL} trace will be very noisy).

./model_single_3D Model minimal Beats 10 Sim_cell_size testing Vclamp On Reference Modulation_example Results_Reference Control_ICAL

./model_single_3D Model minimal Beats 10 Sim_cell_size testing Vclamp On ICaL_scale 2 Reference Modulation_example Results_Reference NLTCC

./model_single_3D Model minimal Beats 10 Sim_cell_size testing Vclamp On LTCC_Po 2 Reference Modulation_example Results_Reference LTCC_Po

In "Outputs_Modulation_example/Results_{Control_ICAL/NLTCC/LTCC_Po}/Parameters" are now data for I_{CaL} voltage clamp. To plot/analyse the raw current data, use ICaL_Vclamp_traces.dat - plot time (column 1) against voltage (2), current (3) or Cai (4). To plot IV relationships, use ICaL_IV.dat (column 1 vs 2 for voltage and current).

Look at "Outputs_Modulation_example/Results_{Control_ICAL/NLTCC/LTCC_Po}/CRU.dat" using columns: 1 - time; 1 - AP; 5 - Cai; 16 – JCaL and "../Currents.dat" using 1 (time) and 12 (ICaL). Note that a scale factor of 2 for ICaL_scale and LTCC_Po do not have identical effects; if you are matching data based on current measurements, you will need to set the scale to give the correct magnitude.

Apply multiple, simultaneously: Increase SERCA (Jup_scale), increase activity of ICaL but not channel numbers (LTCC_Po), reduce numbers of RyRs (Jrel_scale) but increase open rate (RyR_Po)

./model_single_3D Model minimal Beats 10 Sim_cell_size testing Jup_scale 2 LTCC_Po 1.5 Jrel_scale 0.5 RyR_Po 2.5 Reference Modulation_example Results_Reference Multiple

5. Producing a spontaneous release dataset

This protocol will apply rapid pacing protocol to load the CaSR, then run multiple simulations to produce a dataset on which spontaneous release can be analysed. This will also explore and use the **State_write ave/On** functionality (similar to tissue models). In this example, we are not rigorously testing to ensure we have reached a stable-state, but you should do this for real investigation!

Rapid pace testing cell and write state as whole-cell averages; apply ISO and further enhance Jup in order to promote CaSR loading we'll also set **tau_ss_type** to **medium_fast** to promote spontaneous release, for this example. Do this to the testing cell size in order to produce state file more quickly (or full size if too noisy). Note: The spatial and 0D models, by default, apply pacing and then leave a 2 second quiescent period to analyse spontaneous release; in order to correctly write the state file, we need to explicitly set the

Total_time to 17500 (BCL × Beats). State write also set to "ave," as we want to read the state into the full sized model later - and clearly cannot do this with a spatial state file

./model_single_3D Model minimal Beats 50 BCL 350 ISO 1 Jup_scale 2 tau_ss_type medium_fast Sim_cell_size testing Total_time 17500 Write_state ave Reference Spontaneous_release Results_Reference Pre_pace

We can check in "Outputs_3Dcell_Spontaneous_release/Results_Pre_pace/CRU.dat" using columns 1 and 7 to plot CaSR against time; in this example, it loads to ~1100 mM by the end of the simulation (just enough to induce some spontaneous release!).

Now read in that state (which is written as whole-cell averages) into the full-size cell model. Pace this model for a couple of beats, and write the whole state file which includes the spatial state (local concentrations and RyR/LTCC state). Here you'd probably want more than 2 beats for real work.

./model_single_3D Model minimal Beats 2 BCL 350 Read_state On ISO 1 Jup_scale 2 tau_ss_type medium_fast Sim_cell_size full Read_state ave Write_state On Total_time 700 Reference Spontaneous_release Results_Reference Pre_pace_full

Then run multiple simulations to produce a dataset which can be analysed statistically. Apply just 1 beat, as this is why we wrote the state file. Produce spatial data outputs for the first 2 simulations (to visualise spontaneous calcium activity). We now want the **Total_time** to include a 2 second quiescent period, so no need to pass the argument.

For *run* in 0 1

> ./model_single_3D Model minimal Beats 1 BCL 350 Read_state On ISO 1 Jup_scale 2 tau_ss_type medium_fast Sim_cell_size full Reference Spontaneous_release Results_Reference Run_*run*_spatial_vis

For *run* in sequence 2 to 9 in steps of 1

> ./model_single_3D Model minimal Beats 1 BCL 350 Read_state On ISO 1 Jup_scale 2 tau_ss_type medium_fast Sim_cell_size full Spatial_output_interval_data 0 Reference Spontaneous_release Results_Reference Run_*run*

We have a look at "Outputs_3Dcell_Spontaneous_release/Results_Run_0000_spatial_vis/CRU.dat" looking at columns 5 (Cai) and 9 (open RyR state occupancy) and indeed see there is spontaneous activity after the 5th applied beat (likely for at least one of the two visualisation simulations under these example conditions). So, we may want to convert the binary spatial data into vtks for visualisation.

./bin_to_vtk_3Dcell Sim_cell_size portion Reference Spontaneous_release Results_Reference Run_0000_spatial_vis start_time 300 end_time 2350 interval 10 Variable Ca

And use open RyR state (CRU.dat column 9) to analyse spontaneous release timing statistics, using some threshold of open RyR occupancy occurring after 300 ms to determine if a spontaneous release has occurred and its timing, or simply plot the overlays of all simulations' open RyR or Cai (CRU.dat columns 9 or 5) to inspect spontaneous release variability.

6. The effect of the "toy" remodelling models on CaSR and spontaneous calcium release

This example illustrates the effect of the two "toy" remodelling models on SR-loading spontaneous release. We are essentially going to repeat the previous example, with the additional modulations:

For *rem* in none RSERCA_NCX RCRU

./model_single_3D Model minimal Beats 50 BCL 350 ISO 1 Jup_scale 1.5 Sim_cell_size testing Remodelling *rem* Total_time 17500 Write_state ave Reference Spontaneous_release_remodelling Results_Reference Pre_pace_*rem* State_Reference_write pre_pace_*rem*_ave Spatial_output_interval_data 0 Spatial_output_interval_vtk 0

Notice that RSERCA_NCX has a significant effect on SR Ca2+ loading, whereas RCRU does not.

Now read in state and pace full cell for a couple of beats

For *rem* in none RSERCA_NCX RCRU

./model_single_3D Model minimal Beats 2 BCL 350 ISO 1 Jup_scale 1.5 Sim_cell_size full Remodelling *rem* Read_state ave State_Reference_read pre_pace_*rem*_ave Write_state On State_Reference_write pre_pace_*rem*_full Total_time 700 Reference Spontaneous_release_remodelling Results_Reference Pre_pace_*rem*_full Spatial_output_interval_data 0 Spatial_output_interval_vtk 0

And finally, a couple of sims to look at spontaneous release:

For *rem* in none RSERCA_NCX RCRU

for *run* in 0 1

./model_single_3D Model minimal Beats 1 BCL 350 Remodelling *rem* Read_state On State_Reference_read pre_pace_*rem*_full ISO 1 Jup_scale 1.5 Sim_cell_size full Reference Spontaneous_release_remodelling Results_Reference Spont_*rem*

6.5 0D cell model examples – Spontaneous Release Functions

1. Control pacing

Control pacing using the 0D model, which should be roughly equivalent to the 3D cell mode.

./model_single_0D Model minimal BCL 500 Beats 200 Reference Control_pacing_minimal

Output data will be in "Outputs_0Dcell". Further to "Results/Currents.dat", which has time courses for the AP and ion currents, "Results/CRU.dat" contains the intracellular Ca2+ concentrations and RyR, LTCC and SERCA dynamics (exactly as with 3D cell models).

2. Spontaneous release functions – Preset direct control

Simplest example of the spontaneous release functions, using direct control presets. "Direct_Control" simply means the SRF parameters will be sampled from a single, defined distribution for each beat. We will use the preset model "General_1" for this first example

First, just one beat so see some SRF

./model_single_0D Model minimal BCL 0500 NBeats 1 SRF_mode Direct_Control SRF_Pset General_1 reference Direct_control results_reference 1_beat

And if we look at "Outputs_0Dcell_Direct_control/Results_1_beat/CRU.dat", we can see:

- A DAD (columns 1 vs 2 - voltage)

- The spontaneous calcium transient underlying the DAD (columns 1 vs 5 - Cai)
- And the open RyR SRF waveform (columns 1 vs 9 - NRyR_O)

This implementation can be used with multiple beats - the distribution is the same each time. Let's apply a few beats, one at a BCL faster than the likley fastest ti, and one slower:

./model_single_0D Model minimal BCL 0350 NBeats 3 SRF_mode Direct_Control SRF_Pset General_1 reference Direct_control results_reference 3_beats_BCL_0350

./model_single_0D Model minimal BCL 0350 NBeats 3 SRF_mode Direct_Control SRF_Pset General_1 reference Direct_control results_reference 3_beats_BCL_0350

And we can see that when the BCL is slower, we get SCRE on each beat (and it has variability, even with the same distribution).

3. Controlling the Direct_Control SRF parameters at run-time

We will still use the Direct_Control mode, but this time we will apply the User_Control Pset, which requires all the distribution parameters to be set at run-time.

Settings which need to be passed:

- SRF_DC_PSCRE - probability of release (per excitation)
- SRF_DC_CF - Cumulative frequency at ti separation point
- SRF_DC_ti_sep - ti at this point
- SRF_DC_ti_W1 - width of ti disitritbion to left of ti_sep
- SRF_DC_ti_W2 - width of ti distribution to right of ti_sep
- SRF_DC_MD - Median distribution
- SRF_DC_duration_W - width of duration distribution (*optional; we set from MD is not passed)SRF_DC_PSCRE

Let's run a couple of very different distributions to see their effect.

./model_single_0D Model minimal BCL 1000 NBeats 1 SRF_mode Direct_Control SRF_Pset User_control SRF_DC_PSCRE 0.5 SRF_DC_CF 0.4 SRF_DC_ti_sep 1000 SRF_DC_ti_W1 400 SRF_DC_ti_W2 700 SRF_DC_MD 500 SRF_DC_duration_W 300 reference DC_User_control results_reference low_SCRE

./model_single_0D Model minimal BCL 1000 NBeats 1 SRF_mode Direct_Control SRF_Pset User_control SRF_DC_PSCRE 1 SRF_DC_CF 0.4 SRF_DC_ti_sep 500 SRF_DC_ti_W1 100 SRF_DC_ti_W2 200 SRF_DC_MD 150 SRF_DC_duration_W 50 reference DC_User_control results_reference high_SCRE

The effect of these settings is clear from the results: dist 2 leads to much larger spontaneous calcium transients, with some eliciting a triggered AP (run a few simulations - append Results_Reference with a run number if you like).

We can also view/plot the distributions directly:

plot: "…/Parameters_low_SCRE/SRF_distributions/ti_distribution.dat" using 1 and 2,

and compare it to "…/Parameters_high_SCRE/SRF_distributions/ti_distribution.dat"

And similarly for "duration_distribution" and "NRyRo_peak_distribution".

4. Using the dynamic SRF models, fit to 3D cell statistics

Now using a dynamic model, fit to the 3D cell model behaviour. As this model depends on CaSR, we will prepace the model to 3 different CaSRs so we can see the effect. We will apply ISO, and maybe some

Jup_scale, to help load the CaSR . We need to pass "SRF_model 3D_cell" to select from models fit to the 3D cell model (rather than general).

Recall that we need to set total time direclty to avoid leaving a 2sec quiescent period

Below settings load CaSR to ~ 800, 1000 and 1200 mM

./model_single_0D Model minimal BCL 400 ISO 1 NBeats 5 Total_time 2000 Write_state On state_reference_write 5beats Reference Dynamic_3D_cell Results_Reference Pre_pace_5

./model_single_0D Model minimal BCL 400 ISO 1 NBeats 50 Total_time 20000 Write_state On state_reference_write 50beats Reference Dynamic_3D_cell Results_Reference Pre_pace_50

./model_single_0D Model minimal BCL 400 ISO 1 NBeats 50 Total_time 20000 Jup_scale 2 Write_state On state_reference_write 50beats_Jup Reference Dynamic_3D_cell Results_Reference Pre_pace_50_Jup

Now, read in each and apply 1 beat, then apply the Dynamic SRF mode and different Psets.

For *pset* in Control RSERCA_NCX RCRU

> ./model_single_0D Model minimal BCL 400 ISO 1 Beats 1 Read_state On state_reference_read 5beats SRF_mode Dynamic SRF_model 3D_cell SRF_Pset *pset* Reference Dynamic_3D_cell Results_Reference pset_800
>
> ./model_single_0D Model minimal BCL 400 ISO 1 Beats 1 Read_state On state_reference_read 50beats SRF_mode Dynamic SRF_model 3D_cell SRF_Pset *pset* Reference Dynamic_3D_cell Results_Reference *pse}*_1000
>
> ./model_single_0D Model minimal BCL 400 ISO 1 Beats 1 Jup_scale 2 Read_state On state_reference_read 50beats_Jup SRF_mode Dynamic SRF_model 3D_cell SRF_Pset *pset* Reference Dynamic_3D_cell Results_Reference *pset*_1200

Notice how the SRF models depend on CaSR differently.

If we also passed "Remodelling RSERCA_NCX" into that case, we would also see different CaSR loading (would have to create new state files). Notice therefore that the SRF model can be set independent of cell model conditions.

We can also see what the SRF distributions at each CaSR for each model are in "../SRF_distributions/X_distribution_YmM.dat" and how the parameter vary with CaSR in "../CaSR_dependence.dat".

5. Using the dynamic, controllable SRF models

We can also use a controllable dynamic SRF model. Some presets Psets have been included, so we will first run one of these (set in lib/Spontaneous_release_functions.cpp), and then demonstrate how to set the CaSR dependence using the User_control approach.

We will use the CaSR loaded state files from the previous example.

./model_single_0D Model minimal BCL 400 ISO 1 Beats 1 Read_state On state_reference_read 5beats SRF_mode Dynamic SRF_model General SRF_Pset General_1p10 Reference Dynamic_controllable Results_Reference General_800

./model_single_0D Model minimal BCL 400 ISO 1 Beats 1 Read_state On state_reference_read 50beats SRF_mode Dynamic SRF_model General SRF_Pset General_1p10 Reference Dynamic_controllable Results_Reference General_1000

./model_single_0D Model minimal BCL 400 ISO 1 Beats 1 Jup_scale 2 Read_state On state_reference_read 50beats_Jup SRF_mode Dynamic SRF_model General SRF_Pset General_1p10 Reference Dynamic_controllable Results_Reference General_1200

Now we will control the CaSR dependence directly at run-time.

Again, we will use a settings file to make this tidier

We must pass:

- SRF_Dyn_PSCR_threshold CaSR threhsold above which SCRE occurs
- SRF_Dyn_CaSR_max CaSR value at which SCRE dynamcis converge
- SRF_Dyn_CaSR_Prange Range of CaSR for which probability goes from 0 to 1
- SRF_Dyn_ti_sep_max ti_sep at CaSR_max
- SRF_Dyn_ti_sep_min ti_sep at CaSR_threshold
- SRF_Dyn_ti_width_max ti dist width at CaSR_max
- SRF_Dyn_ti_width_min ti dist width at CaSR_threshold
- SRF_Dyn_MD_max MD at CaSR_max
- SRF_Dyn_MD_min MD at CaSR_threshold
- SRF_Dyn_duration_width_max duration dist width at CaSR_max
- SRF_Dyn_duration_width_min duration dist width at CaSR_threhold
- SRF_Dyn_H power of CaSR dependent functions

./model_single_0D Model minimal BCL 400 ISO 1 Beats 1 Read_state On state_reference_read 5beats SRF_mode Dynamic SRF_model General SRF_Pset User_control SRF_Dyn_PSCR_threshold 0.9 SRF_Dyn_CaSR_max 1.2 SRF_Dyn_CaSR_Prange 0.1 SRF_Dyn_ti_sep_max 1000 SRF_Dyn_ti_sep_min 200 SRF_Dyn_ti_width_max 800 SRF_Dyn_ti_width_min 200 SRF_Dyn_MD_max 700 SRF_Dyn_MD_min 100 SRF_Dyn_duration_width_max 500 SRF_Dyn_duration_width_min 50 SRF_Dyn_H 2.5 Reference Dynamic_controllable Results_Reference User_control_800

./model_single_0D Model minimal BCL 400 ISO 1 Beats 1 Read_state On state_reference_read 50beats SRF_mode Dynamic SRF_model General SRF_Pset User_control SRF_Dyn_PSCR_threshold 0.9 SRF_Dyn_CaSR_max 1.2 SRF_Dyn_CaSR_Prange 0.1 SRF_Dyn_ti_sep_max 1000 SRF_Dyn_ti_sep_min 200 SRF_Dyn_ti_width_max 800 SRF_Dyn_ti_width_min 200 SRF_Dyn_MD_max 700 SRF_Dyn_MD_min 100 SRF_Dyn_duration_width_max 500 SRF_Dyn_duration_width_min 50 SRF_Dyn_H 2.5 Reference Dynamic_controllable Results_Reference User_control_1000

./model_single_0D Model minimal BCL 400 ISO 1 Beats 1 Jup_scale 1 Read_state On state_reference_read 50beats_Jup SRF_mode Dynamic SRF_model General SRF_Pset User_control SRF_Dyn_PSCR_threshold 0.9 SRF_Dyn_CaSR_max 1.2 SRF_Dyn_CaSR_Prange 0.1 SRF_Dyn_ti_sep_max 1000 SRF_Dyn_ti_sep_min 200 SRF_Dyn_ti_width_max 800 SRF_Dyn_ti_width_min 200 SRF_Dyn_MD_max 700 SRF_Dyn_MD_min 100 SRF_Dyn_duration_width_max 500 SRF_Dyn_duration_width_min 50 SRF_Dyn_H 2.5 Reference Dynamic_controllable Results_Reference User_control_1200

And we can inspect the results and distributions exactly as before. Have a play with changing the settings and seeing what happens at different CaSRs etc!

6.6 0D tissue model examples – Spontaneous Release Functions at the organ scale

We can use all the same commands as for native tissue models and the SRF commands from 0D tissue models. So we'll just go through one protocol to load the CaSR in a tissue model and apply some SRF. We'll also increase dt as this is a simple case and it will speed it up.

We'll start by reading in the state files from the high CaSR load single cell 0D example, and writing the whole tissue state:

./model_tissue_0D Tissue_order 2D Tissue_model basic dt 0.025 BCL 400 ISO 1 Jup_scale 2 Read_state single_cell state_reference_read 50beats_Jup Write_state On State_Reference_write 2D_tissue_CaSR_load Beats 2 Total_time 800 Reference Tissue_SRF_pre_pace

Now we'll read that state file and apply the RCRU SRF:

./model_tissue_0D Tissue_order 2D Tissue_model basic dt 0.025 Beats 1 BCL 400 ISO 1 Jup_scale 2 Read_state On State_Reference_read 2D_tissue_CaSR_load SRF_mode Dynamic SRF_model 3D_cell SRF_Pset RCRU Reference Tissue_SRF

And convert Vm and Cai binary spatial data to vtk. Note the "Moidel_type integrated" argument

./bin_to_vtk_tissue Tissue_order 2D Tissue_model basic Model_type integrated Reference Tissue_SRF start_time 0 end_time 2000 interval 10 Variable Vm

./bin_to_vtk_tissue Tissue_order 2D Tissue_model basic Model_type integrated Reference Tissue_SRF start_time 0 end_time 2000 interval 10 Variable Ca

II - Modifying the code

Forward - general philosophy of the framework

Firstly, it should be clarified this is not a library, tool-set, scripting language or similar for cardiac cell and tissue modelling. Rather, this is a single implementation in C/C++ which contains all of the functionality for single cell (spatial and non-spatial) and tissue (idealised and anatomically detailed) simulations of cardiac electrical excitation which I use in my research[1].

I personally prefer to develop my models in C/C++ with all functionality hard-coded and easily readable/modifiable. Over the course of my research so far, this has led to uncountable different project codes, implementing different cell and tissue models and with different conditions, functionality and protocols. Whereas this led to clean and efficient implementation for specific purposes, it became rather tedious to copy sections of code when implementing functionality to be applied to multiple models or projects; In particular, between single-cell and tissue implementations of the same model, or introducing new components to multiple models for model-independent studies.

This software represents a significant effort to contain everything I use in my research in one, functional and readable implementation such that once a new modification or cell model, tissue geometry and so on, has been developed, one has access to the full functionality without the need to double-up on code or use multiple projects etc. The motivation was firstly to facilitate my own research, and secondly to make the code and models easily accessible for my students and other researchers. Following this, and with the principals of openness, transparency and community support, there was also motivation to make the code as easy to interpret and modify as possible such that others may use and adapt the code with the same ease as myself. This documentation is designed to facilitate this purpose. I hope that this will be of assistance in the reader's own research.

1. Introduction

Because this code has been designed maintain significant functionality for running multiple different cell models and conditions, it is necessarily relatively large and consists of multiple files and variable structs; the structure and contents of the code are therefore detailed below and in the appendix, to assist in using and modifying the software. However, it should be easy to follow the code through using the algorithm schematic and reading the appropriate Main file (wherein the relevant files containing all called functions have been commented). The instructions below should be sufficient for most modifications; the appendix containing full file and function lists should only be required for specific reference or significant modifications.

Instructions on how to add new modifiers, such as celltype, remodelling, mutation, pharmacological agent, or isoprenaline, as well as a template file and instructions on how to add a full new model to the code, are given below. Whereas some tasks may be more involved than implementations in simple code, it (I hope!) will be more than worth it for long-term functionality.

The general philosophy of the code is simplicity to the extent of practicality. The code is written primarily in C to facilitate readability and following the inherently sequential nature of the simulations required. However, some additional functionality from C++ was used; Thus, whereas the code is compiled as C++, it retains the sequential nature of C and does not (primarily) utilise classes. Furthermore, the requirement for additional libraries (such as higher-order solvers) was avoided in order to maintain simplicity and facilitate portability and ease of installation.

---

[1] Not all functionality may be present with specific code versions; all models are included in the global, parent code which does contain all functionality.

This software may be modified (see disclaimer at the start of this document). If you would like any modifications and additions to be shared as part of the framework (with appropriate credit given and citations added to the disclaimer) please feel free to send them to me. If deemed appropriate, the additions will be incorporated into the framework for future downloads.

For any new component you may add, please also add the relevant citations to the disclaimer output. In **lib/Outputs.cpp** go to output_disclaimer_citations(), simply add an entry here which prints the citation to screen if your new model/component is used.

2. Variable naming conventions

Where possible, variables have been named using a consistent convention.

For currents, gating variables, and variables which determine these, the convention used throughout is as such: "$I_{X\_type\_specifier}$". For example, the "voltage-activation" (va) gate for I_{to} is "**Ito_va**"; the "calcium-inactivation" (ci) gate for I_{CaL} is "**ICaL_ci**"; the steady state (ss), time constant (tau) or alpha-transition rate (al) of the "voltage-inactivation" (vi) gate for I_{Kur} are: "**IKur_vi_ss**", "**IKur_vi_tau**" or "**IKur_vi_al**". For maximal flux rates, for example for intracellular dynamics: "**I/J_x_bar/max**" – for example, "**J_rel_max**", "**INCX_bar**". For parameters and constants, in general denoted with "**I_x_kref**" – example "**INCX_kCai**" would be the intracellular calcium constant associated with INCX whereas "**J_SERCA_kCaSR**" is the SR-calcium constant associated with SERCA uptake.

3. Variable structs and contents

All model variables and parameters are contained within structs in order to allow access from multiple files and functions. All structs are defined in "**lib/Structs.h**", and consist of:

Struct	Contents
Argument_parameters	Variables which are used to control/modify model settings from the command line arguments (i.e. a variable associated with each option listed in the arguments). Argument variables are used for settings only.
Simulation_parameters	Global simulation settings: *BCL, Total_time, Paced_time, NBeats, S2, NS2, dt, Vclamp, Read/Write_state.*
Cell_parameters	Parameters which are not dynamically updated during simulation. Includes **model conditions** (*Model, Celltype, Remodelling, ISO etc*), **constants** (*R, F, T*), **cell structure** (C_m, volumes, etc), **stimulus parameters** (*stimduration, stimmag*), **ion-current parameters** (conductance, maximal flux rate, saturation constant etc), **ionic concentrations** (initial or constant values), **native calcium handling parameters** (CICR model, buffering etc), **modifier variables** (scale factors, voltage shifts etc).
State_variables	Time-dependent variables which are integrated numerically. Includes **V_m, gating variables, ionic concentrations** etc
Model_variables	Variables calculated dynamically during the simulation. Includes **stimulus variables** (*Istim, stimflag* etc), **reversal potentials** (E_x), **ionic currents** (I_{tot}, I_x), **intracellular fluxes** (J_x), **transition rates and steady-states** (a, β, ∞, τ etc), **excitation properties** (APD, dv/dt, $[Ca^{2+}]_i$_min/max etc).
Tissue_parameters	Parameters associated with setting up and running tissue models. **Tissue model conditions** (*Tissue_model, Tissue_order, Tissue_type, Orientation_type* etc), **Stimulus location parameters**, **Tissue dimensions and diffusion parameters**, **map files and flags**, **Heterogeneity and anisotropy settings**, **Conduction velocity measurement variables**
SC_variables	All variables and parameters for spatial diffusion and coupling (tissue and 3D cell). **Dimensions, space steps, geometry and map arrays, Diffusion coefficient and differential arrays, neighbour arrays and maps, local orientations, spatial Laplacian.**

And for integrated (3D cell and 0D) models:

Struct	Contents
Ca_variables	Variables for calcium concentrations. Includes **global** and **local concentrations** and **buffering variables.**
CRU_variables	Variables and parameters for calcium handling system dynamics. **Cell size settings**, **dimensions**, **whole-cell averages**,
Dyad_variables	Variables for computing dyad fluxes – RyR and LTCCs.
SR_fluxes	Variables for computing Jup and Jleak
Membrane_fluxes	Variables for computing membrane fluxes
RAND	Random number arrays
Spontaneous_release_functions	Variables and parameters for the SRF implementations.

4. Adding new variables

You may need to add new variables to the code as part of any modification you are making; variables for most model components already exist, so first please to check **lib/Structs.h** for matching or suitably similar variable. If your variable does not already exist, then you will need to add it. This procedure depends on the type of variable you are adding:

- **Local variables:** if the variable is computed for the calculation of a current/flux/concentration, and is not required for use outside of its used function, create it locally. Otherwise:
- **Parameters:** Add the parameter to the appropriate location of Cell_parameters struct. It is now ready to be used - simply give it a value in the relevant set_parameters_native_X() function in **lib/Model_X.cpp**, or in set_default_parameters() in **lib/Initialisation.c**, if it applies to multiple models.
- **Computed variables** (e.g. steady state, transition rates, currents, fluxes): Simply add to the appropriate location of the Model_variables struct. It is now ready to be accessed from any function which reads in Model_variables.
- **State variables (gating variables, concentrations and other time-dependent variables):**
    - Add to struct State_variables.
    - Set the initial condition in initial_conditions_native_X() in the relevant **lib/Model_X.cpp** file.
    - Finally, add it to read/write state functionality. In **lib/Read_write_state.c**, navigate to the first two functions {Write/Read}_state_variables_native() and follow the instructions at the bottom of each function to add a new variable.
- **Modifier variables** (current/flux scaling; voltage shifts etc):
    - Add to the appropriate location of Cell_parameters struct (search "MODIFIERS").
    - Set the default value in set_modification_defaults_native() in **lib/Initialisation.c** (1 for scale; 0 for shift).
    - Note: shift applies by adding to V1/2 and thus subtracting from V.
- **Tissue parameters**: Add to Tissue_parameters struct and default (if necessary) in set_tissue_model_conditions() in **lib/Tissue.cpp**. Set either here or in relevant set tissue settings functions (set_tissue_settings_idealised() or set_tissue_settings_anatomical()).
- **Variable/parameter to be controlled from command line arguments:**
    - First, add the variable itself as above (e.g. a current scale modifier); the following describes how to set and modify this variable from command line/settings_file arguments; the variable itself must already be included and do what you want it to!
    - In the Argument_parameters struct (last one in the Structs.h file), add:
        - A copy of the variable you want to modify (e.g. a new current scale)

        - A boolean flag variable with the same name with "_arg" appended.
  - Default the ".._arg" boolean to **false** in **lib/Arguments.c** -> set_argument_defaults().
  - Add a condition to the IF statements in function set_arguments() in **lib/Arguments.c**, following the examples already present. Ensure to set the flag and increment arg_counter within this conditional.
  - Add your new argument and its options to screen outputs at the bottom of that function.
  - In **lib/Initialisation.c**, navigate to assign_modification_from_arguments() and add a line which sets the model variable to the argument variable if the _arg flag has been set to true (follow examples).
  - This will ensure the argument value will be applied, but only if argument is passed.
  - If it is a **Tissue parameter**, then you may also need to navigate to overwrite_tissue_properties_from_args() in **lib/Tissue.cpp** and add a clause there to overwrite/set the tissue parameter from your argument parameter.
  - Finally, you can add it to screen settings outputs in output_settings() in **lib/Outputs.cpp**.

5. Adding a new celltype or modulation model

The code has been presented with extensive variables to modify the equations of the ion currents (and basic variables to modify calcium handling properties – the **modifier variables** in the Cell_parameters struct), and all included models have been implemented with these variables. Therefore, for the majority of modifications it is simple to implement a new modifier which affects all, some, or a specific cell model. Instructions are embedded in the relevant parts of the code; here, we outline what needs to be done and where. These instructions are intended to be read while the user is updating the code.

5.1. The modifier variables

Modifier variables, elements of Cell_parameters, are grouped into different types:

1. Scale factors – denoted **Gx** where x refers to the current or flux. These simply multiply the maximum conductance or flux rate. E.g. **GNa**, **Grel**. These are defaulted to 1.0.
2. Voltage-shifts – denoted **Ix_vy_ss_shift** and **Ix_vy_tau_shift**, where x denotes the current and y denotes the gating variable (activation, inactivation). These are subtracted from the voltage as used in the steady-state or time constant equations, i.e. equivalent to adding to the $V_{1/2}$. E.g. **Ito_va_ss_shift**, **ICaL_vi_tau_shift**. Defaulted to 0.0.
3. Time constant scaling – denoted **Ix_vy_tau_scale** simply multiples the calculated time-constant of current x gating variable y. Defauled to 1.0.
4. Gradient scaling – denoted **Ix_vy_ss_kscale** – scales the gradient parameter of regular sigmoidal functions for the steady state of current x and gate y. Defaulted to 1.0.

These can be accessed in all functions which read in Cell_parameters, and should in general be used instead of setting parameters explicitly to apply modulation. In **lib/Structs.h**, search "MODIFIERS" for a full list.

Note on I_{CaL} and J_{rel} modifiers: There are two ways to scale these fluxes in the integrated cell models, but only one way in the native cell models. In the integrated cell models, the fluxes can be scaled by either changing the expression (N per dyad, corresponding to Cell_parameters.{GCaL/Grel}) or by scaling the open transition rates (Cell_parameters.{GLTCC_kva1_va2/GRyR_kCO}). Both of these have the same effect in native models (Cell_parameters.{GCaL/Grel} is simply set to GLTCC_kva1_va2/GRyR_kCO if using native models) but do have different effects in the integrated models, which may not linearly correspond. So please think about what your modulation represents (change in channel expression or availability?) and use the appropriate variable for the correct integrated implementation, bearing in mind they both do the same for native models so either can be used.

5.2 Types of modulation

Modulation models are grouped as: **Celltype** – modifications for different celltypes within the same model i.e. regional heterogeneity; **Remodelling** – modifications for different remodelling conditions e.g. associated with atrial fibrillation or heat failure; **ISO/ACh** – models for sympathetic stimuluation/isoprenaline and parasympathetic stimulation/acetylcholine, passed as 0-1 representing up to a saturating concentration; **Agent** – Name/specifier for pharmagological agents; **Mutation** – specific channel mutation; **Spatial_gradient** – for continuous variation of parameters by a spatial map in tissue models.

Each of these types has its own specific function in which to place your new modifier. If your modifier fits within one of these categories, please follow the instructions below; adding a new modifier type is slightly more involved and requires more detailed understanding of the code (instructions may be added to this documentation in future). The code is set up such that each modifier can only have one option at a time (i.e. you cannot have two **Mutation** modifiers simultaneously), but may simultaneously run an option for each of the different modifiers (i.e. you can have **Celltype**, **Remodelling, ISO, Agent** and **Mutation** simultaneously) – the modifications will be applied sequentially, and so it is important to modify rather than set parameters unless explicitly intended. If you want to include models for multiple of the same type of modifier (e.g. AF and HF in **Remodelling**), then you must specify the combination as its own option (e.g. AF-HF).

An illustration of how to code modifiers is given in each relevant function as either "test" or "test_global"; some real modifiers may also be already present in the relevant function. You can use **lib/Model_TEMPLATE.cpp** as a guide if the function you need does not already exist.

The method of introducing new modifiers is the same whether they are model-specific, common to many models, or global – only the location and function in which the modifier is placed is determined by specificity.

5.3. Adding your new modulation; model-specific

Open the file associated with the model to which your modifier applies ("**lib/Model_X.cpp**").

Navigate to the appropriate function:

- set_celltype_native_X()
- update_celltype_integrated_X()
- set_modulation_ISO_native_X()
- set_modulation_Agent_native_X()
- set_modulation_Remodelling_native_X()
- set_modulation_Mutation_native_X()
- set_modulation_ACh_X()

And add a new condition of the IF statements with your new modulation identifier. If the function is not present in your selected cell model, copy the template function from **lib/Model_TEMPLATE.cpp** and add the function call to set_het_mod_X() and the function to **lib/Model.h.**

Now follow the in-code instructions for incorporating your model, using the modifier parameters described above. A few notes:

- Remember to modify (multiply/add) your modifier variables rather than explicitly set.
- For all modulation other than celltype and mutation, you can apply an amount to apply the modulation. For ISO and ACh models, this variable is Cell_parameters.{ISO/ACh}; for Remodelling and Agent, this variable is Cell_parameters.{Remodelling/Agent}_prop. The variable

will always be in the range 0-1 to specify the strength of the modulation. The convention in the code, to allow easy readability of the maximum effect of the modulation, is to write it as:

- Cell_parameters.[MODIFIER] *= (1.0 + proportion * (max_effect – 1)) [scale factor]
- Cell_parameters.[MODIFIER] += proportion * max_effect [shift]

- For example, if the maximum effect of **ISO** on $\boldsymbol{I_{CaL}}$ is to **increase the current by a factor 3** and to **shift the voltage dependence of the activation gate by -5 mV**, we would add:
  - Cell_parameters.GCaL *= (1.0 + ISO * (3.0 – 1.0))
  - Cell_parameters.ICaL_va_ss_shift += ISO * -5.

5.4. Adding your new modulation; common or global

You may want to add a new modifier which applies to multiple cell models. This can be done by placing the modifier in **lib/Model.c**, without the requirement to repeat or copy code for multiple different models. In general, a modifier may be common or global if: (1) It is a pharmacological agent which affects different celltypes or species homogeneously; (2) There are multiple, independent models of the same cell (e.g. human atria, rabbit ventricle) to which heterogeneity, ISO etc apply; (3) It is a mutation which affects multiple celltypes or species homogeneously; or similar case.

First, in **lib/Model.c**, navigate to set_heterogeneity_and_modulation_native().

If a global/common function already exists which covers the new modulation you need to add, you simply need to place your modifier within this function following the same procedure as detailed above for model-specific. Global/common functions already present in the code: set_global_Agents() – for any drug which affects all models homogeneously; set_celltype_hAM() – regional celltypes for all human atrial cell models; set_ISO_hAM() - ISO models for human atrial cell models; set_global_remodelling() – remodelling models which apply to every cell model; set_remodelling_hAM() - AF remodelling (or others if added!) models for human atrial cell models; set_mutation_hAM() - I_{Kur} mutations (or others if added!) models for human atrial cell models; set_ACh_global() – any global model of parasympathetic activity.

If a global/common function does not already exist, you will need to create it. (1) define the function in "**lib/Model.h**" where the other global/common modifier functions are (near the start of the file) – a template function has been placed there called set_MODIFIER_X_Y() so copy this and rename as appropriate (if global, call it "set_global_MODIFIER()", if species specific call it "set_MODIFIER_species()" and so on). Now, in "**lib/Model.c**" navigate to below the set_heterogeneity_and_modulatioin_native() function, where the global/common modifier functions are listed. An entry for the template is placed here, so you may also copy this and use it as a guide. [**Note:** ensure that the relevant tracking variable (*p->MODIFIER_set_ref*) is appropriately set to 1 and 0 as indicated in the template for proper error checking and selection]. Now, simply add your function call before the model specific selection IF statements in set_heterogeneity_and_modulatioin_native() in the relevant modifier type section. If your function applies to some but not all models, include an IF statement before the function call with a list of the suitable models.

5.5. Adding your new modulation; spatial tissue gradients

Adding a spatial gradient model, such as apico-basal ventricular heterogeneity gradients, is unique in that it is only implemented globally (as it will depend on geometry and species, but not specific cell models). Simply navigate to set_spatial_gradient() in **lib/Model.c** and add your model there, giving it an appropriate specific identifier. In tissue models, implementation will also require a spatial gradient map; in single cells, the gradient parameter can be controlled through the argument "**Spatial_gradient_proportion [0-1]**".

6. Adding a new tissue model

New idealised or anatomically detailed tissue models can be easily added in **lib/Tissue.cpp.**

Simply navigate to: set_tissue_settings_idealised() for an idealised model or set_tissue_settings_anatomical() for an anatomically detailed model. Create a new ELSE IF condition at the bottom of the list, following the examples given in "**functional_model_test**", which contains examples of all currently implemented functionality and instructions on file format etc. If adding an anatomical model, you will need to create all geo, stim and map files and put them in your PATH location in the directory "Tissue_geometries", following file format instructions in the code.

At minimum, you must specify the spatial step (dx), diffusion parameters (D1, D_AR and Diso) and spatial dimensions (NX, NY, NZ) for an idealised model, and the geometry and stimulus files for an anatomically detailed model. All other settings, such as heterogeneity, anisotropy and modulation maps are optional. You can also default whether these properties are turned on or off for your tissue model (which can be overwritten by run-time arguments). For example, you can provide settings for heterogeneity but default the tissue model to be homogeneous; you must provide settings for any of these options, independent of the default, if you want the functionality to be available when passing run-time arguments.

7. Adding a new spontaneous release function model

Spontaneous release function parameters are controlled in **lib/Spontaneous_release_functions.cpp.** You can add a new model for Direct_control (static probability distribution settings) or Dynamic, either based on analysis of the 3D cell or general and controllable. Simply navigate to the appropriate function (below) and add an ELSE IF condition following the examples already present.

- set_SRF_Direct_Control_parameters() - single distributions from which to sample parameters
- set_SRF_dynamic_parameters_3D_cell() - CaSR dependent distributions, fit to 3D cell model behaviour
- set_SRF_dynamic_parameters_controllable() - CaSR dependent distributions, general and controllable

8. Adding a full new, or updated, cell model

Users are welcomed to incorporate further cell models into the framework (e.g. previously published or new work). A template file for a new cell model has been provided to assist in this task (**lib/Model_TEMPLATE.cpp**) while automatically maintaining all functionality in your new model. Instructions have been embedded in that file – so you can just follow those; below is a summary of what must be done.

New models may be either entirely independent from the already implemented models, or may be an update of an already-included model and inherit functions from the baseline model, in which case, only those new components need to be added to the code (see **lib/Model_hAM_MT.cpp** as an example). These instructions are intended for use while the user is updating the code.

First, select a suitable **model identifier** (let's call it **X**) – this will be used for both file naming and for the conditional statements which select model-dependent functions: the convention implemented is "**speciesCELL_MODEL**", e.g. **H**uman **A**trial **M**yocyte **G**randi-**B**ers model is **hAM_GB**. Now, copy the template to a new file with the name **lib/Model_X.cpp** and search and replace all "**_speciesCELL_MODEL**" with "**_X**"; similarly, copy all the "**_speciesCELL_MODEL**" functions in the header file (**lib/Model.h**) and rename them as appropriate. Add any new functions required following this convention.

The overall structure is that all model-specific functions are in **lib/Model_X.cpp**, with calls to the model-specific functions placed in **lib/Model.c**. Process for adding the actual model formulations:

1. In all main files for which your model is relevant (single cell/tissue, native/integrated), navigate to the "Check Model is appropriate for this code version" section and add a line with your model identifier, so that it will be allowed to run for those implementations.
2. In **lib/Model_X.cpp**, define all parameters used in the model in set_parameters_native_X(), OR, if your model inherits from an already existing model (i.e. is an update of one already included), then you simply need to define all updated parameters in update_parameters_native_X(). Add function calls to **lib/Model.c** -> **set_parameters_native()**. **NOTE:** All ionic concentrations must first be defined as a parameter here.
3. In **lib/Model_X.cpp**, set an initial value for all state variables, including concentrations, in initial_conditions_native_X() and add the function call to **lib/Model.c** -> **initial_conditions_native**(). **NOTE:** Ensure you set the initial value for all the concentration state variables from the equivalent parameter variable set in step 2. This must be performed even for concentration variables which are not updated dynamically in your model.
4. Now go through each current in turn and add the equations – available parameters and variables have been listed in each function to help, and the format to include modulation has been demonstrated. Try to include the modifier parameters properly and fully wherever possible, so that modulation works properly!
   - Ion-channel currents controlled by gating variables have three associated functions: set_Iy_X_rates(); update_gates_Iy_X(); compute_IY_X(). Currents which do not depend on gating variables (i.e. time-independent) only have this last function (or possibly also a version of set_Iy_X_rates() which sets a time-independent but voltage-dependent variable).
   - Ensure you have added function calls for each current to the whole-model call functions (in **lib/Model_X.cpp):**
     - set_gate_rates_X_native() (if the current has a set rates function)
     - update_gating_variables_X_native() (if the current has an update gates function)
     - compute_Itot_X_native() (all currents).
     - And ensure that the current itself is added to *Itot* in compute_Itot_X_native().
   - These three functions are called in compute_model_X_native() and so you must add a call to this function in **lib/Model.c -> compute_model_native()**.
5. Add a call to set_gate_rates_X_native() to the function **lib/Model.c -> compute_and_output_current_functions().** This allows the Parameters outputs to work for your model.
6. Add the homeostasis calculations, including intracellular Ca^{2+} handling, to the function comp_homeostasis_X().
7. Finally, add heterogeneity and modulation for all available/desired model-specific implementations. Follow the instructions above (and in the template) on how to do this, ensuring the specific functions are called in set_het_mod_X(), and that this function is also called in **lib/Model.c -> set_heterogeneity_and_modulation_native()**.
8. If you are introducing a new integrated model (i.e. using the spatial and 0D Ca^{2+} handling systems), you will also need to fill in the function compute_model_X_integrated() (and possibly also update_parameters_integrated_X(), update_het_and_mod_X_integrated(), if relevant) and add calls to these functions in **lib/Model.c** where relevant. You will also need to add the I_{CaL} function calls to **lib/CRU.cpp** function set_LTCC_rates(). Note: combining models is rarely trivial!
9. Don't forget to add the associated citation(s) to **lib/Outputs.cpp -** output_disclaimer_citations().

Re-compile the code, and your model is now ready to be selected by the run-time argument "**Model X**", where X is your model identifier.

Suggestion: When adding a new model, I tend to go through current by current (and then homeostasis), adding the formulations, parameters and initial conditions of state variables for each component in turn

(rather than adding all parameters at once, then all state variables initial conditions at once, then all current formulations etc).

9. Adding a new current

If you need to add a new current to an existing model (or to a model you are adding), and the variables for the current don't already exist (i.e. the current isn't implemented in any of the packaged models), you simply need to add all the related variables (following the directions above; add the current itself and all computed variables to Model_variables; the gating state variables to State_variables, and related parameters to Cell_Parameters (including the **modifier** variables) and ensure the parameters and state variable initial conditions are set in your model function) and then add your functions to the appropriate model, ensuring you add the current to the calculation of Itot and the function calls in the relevant compute and set functions. You do not need to update any settings for models which do not include the current. Finally, you may want to add the current (and its gating variables) to the Results outputs function in **lib/Outputs.cpp**.
